# Comparative transcription profiling of mRNA and lncRNA in pulmonary arterial hypertension after C75 treatment

**DOI:** 10.1186/s12890-023-02334-6

**Published:** 2023-01-31

**Authors:** Cuilan Hou, Lijian Xie, Tingxia Wang, Junmin Zheng, Yuqi Zhao, Qingzhu Qiu, Yi Yang, Tingting Xiao

**Affiliations:** 1 Department of Cardiology, Shanghai Children’s Hospital, School of Medicine, Shanghai Jiao Tong University, No. 355 Luding Road, Shanghai, 200062 China; 2 NHC Key Laboratory of Medical Embryogenesis and Developmental Molecular Biology, Shanghai Key Laboratory of Embryo and Reproduction Engineering, Shanghai, 200062 China; 3 The First Affiliated Hospital of Anhui Medical University, No. 218 Ji-Xi Road, Hefei, 230022 Anhui China; 4 The Children’s Hospital, Zhejiang University School of Medicine, No. 3333 Binsheng Road, Binjiang District, Hangzhou, 310052 China; 5 Department of Pediatrics, JinShan Hospital, Fudan University, Shanghai, China

**Keywords:** C75, Pulmonary arterial hypertension, High-throughput sequencing, Bioinformatic analyses, Cell cycle, Inflammatory

## Abstract

**Objectives:**

To investigate mRNA and long non-coding RNA (lncRNA) expression profiles in monocrotaline (MCT)- mice.

**Materials and methods:**

Lung tissues (Control-Vehicle, MCT-Vehicle, and MCT-C75) were examined by high-throughput sequencing (HTS). Aberrantly expressed mRNAs and lncRNAs were analyzed by bioinformatics. Cell proliferation and cell cycle analysis were performed to detect the potential protective effects of C75, an inhibitor of fatty acid synthase. The signaling pathways associated with inflammatory responses were verified by real time-PCR.

**Results:**

RNA sequencing data reveals 285 differentially expressed genes (DEGs) and 147 lncRNAs in the MCT-Vehicle group compared to the control. After five-week of C75 treatment, 514 DEGs and 84 lncRNAs are aberrant compared to the MCT-Vehicle group. Analysis of DEGs and lncRNA target genes reveals that they were enriched in pathways related to cell cycle, cell division, and vascular smooth muscle contraction that contributes to the PAH pathological process. Subsequently, the expression of eight DEGs and three lncRNAs is verified using RT-PCR. Differentially expressed lncRNAs (ENSMUSG00000110393.2, Gm38850, ENSMUSG00000100465.1, ENSMUSG00000110399.1) may associate in PAH pathogenesis as suggested by co-expression network analysis. C75 can protect against MCT-induced PAH through its anti-inflammatory and anti-proliferation.

**Conclusions:**

These DEGs and lncRNAs can be considered as novel candidate regulators of PAH pathogenesis. We propose that C75 treatment can partially reverse PAH pathogenesis through modulating cell cycle, cell proliferation, and anti-inflammatory.

**Supplementary Information:**

The online version contains supplementary material available at 10.1186/s12890-023-02334-6.

## Introduction

Pulmonary arterial hypertension (PAH) is a progressive and fatal disease characterized by increased pulmonary vascular resistance, then leads to right ventricular failure and ultimately death [1, 2]. The prevalence of PAH is about 1%, rising to 10% of people over 65 years old [3]. It is well known that many targeted therapeutics such as endothelin receptor antagonists, phosphodiesterase 5 inhibitors, and prostacyclin analogs, can improve the life quality of PAH patients [4, 5]. Despite the remarkable progress, the prognosis with PAH patients is still poor, and the molecular pathways underlying the PAH pathogenesis are still largely unknown, thus, it is urgent to discover novel therapeutic targets.

Over the past two decades, metabolic theory has become to be one of the most influential theories in PAH. For example, Gopinath’s group reported that metabolic remodeling occurred in the pulmonary arterial wall in PAH patients [6]. Paulin et al*.* proposed a metabolic theory that integrated cancer-like signals upstream and downstream of mitochondria, which could explain many characteristics of PAH vascular phenotypes, including proliferation and apoptosis resistance [7]. The PAH metabolic theory still needs further exploration. Singh et al. reported that the expression of fatty acid synthase (Fas) was increased in the pulmonary artery smooth muscle cells (PASMCs) and lung tissues in PAH rats model [8]. They also pointed that Fas inhibition played a protective role in regulating of PAH [8]. Our previous study also showed that Fas inhibition played a key role in shielding PAH mice, and partially through the activation of PI3K/Akt signaling [9]. However, there was no comparative transcription profiling of Fas inhibition in PAH model.

Non-coding RNAs are emerging as important regulatory molecules in the development of cardiopulmonary diseases including PAH. In 2010, the first report of investigating the global microRNA (miRNA) profiles of rat lungs during hypoxia and MCT-induced PAH showed that some miRNAs were specific and important in regulating the disease development [10]. Previously, we showed that several miRNAs (miRNA125-3p, miR-125-3p, miR-193, and miR-148-3p) were associated with PAH [11], our further data indicated that miR-29b targeted myeloid cell leukemia 1 and Cyclin D2 to regulate apoptosis and proliferation in PASMCs [12]. Interestingly, various lncRNAs such as Paxip1-as1 and Hoxaas3 are also associated with the regulation of PASMCs proliferation, migration, and apoptosis [13, 14]. Although some miRNAs or linRNAs were reported [11–14], but there still lack comprehensive understand of miRNA/lincRNA–mRNA in PAH. Thus, we aimed to investigate potential mRNA and lncRNA expression profiles in the PAH lung tissues using high-throughput sequencing (HTS) and explore the potential regulatory network in the pathogenesis of PAH.

## Materials and methods

### Animals and experimental design

The animals were raised in the same way as our previously methods [9]. Twenty-four C57BL/6 mice (eight-week-old) were purchased from Shanghai Laboratory Animal Center (Shanghai, China), and grown under controlled conditions (45–55% relative humidity, 22 ± 2°C and 12 h dark–light cycles), with unrestricted access to food and water. The health and weight of these mice were continuously monitored throughout the experimental period. All of the mice were randomly divided into three groups: the disease group (n = 8), in which mice received the MCT (60 mg/kg/week, Sigma, Germany, intraperitoneal injection) and vehicle (0.5%DMSO, Sigma, Germany, intraperitoneal injection) for five weeks to induce PAH [15–18]; the treatment group (n = 8), in which MCT as described in the disease group, followed by C75 intraperitoneal injection (2 mg/kg/week, Sigma, Germany, dissolved in 0.5% DMSO) for five weeks [18]; and the control group (n = 8), which received an equivalent amount of vehicle (0.5%DMSO, Sigma, Germany, intraperitoneal injection) each week.

### Ethics approval

All methods (animal experiments) were carried out in accordance with Shanghai Jiao Tong University Institutional Animal Care and Use Committee guidelines and regulations.

All methods in this manuscript are reported in accordance with ARRIVE guidelines (https://arriveguidelines.org) for the reporting of animal experiments.

### Morphological and histological analyses

Mice lung slices, Hematoxylin–eosin (HE), and Masson staining were in the guide of our previously methods [19]. Mice lung tissues were excised, fixed at 10% formalin, and embedded in paraffin. According to the manufacturer’s instructions, tissue sections (4 μm) were subjected to HE and Masson staining. HE staining was used for mice lung tissue pathological changes, while Masson staining was performed to evaluate the medial wall thickness in small pulmonary arteries. To assess the medial wall thickness [8], 20–25 muscular arteries, categorized as being 20–50 µm and 50–100 µm in diameter, from each lung were randomly outlined by an observer blinded to pharmacological treatment. The degree of medial wall thickness, presented as a ratio of medial area to cross-sectional area (media/CSA), was analyzed by using Image J.

### Analysis of RVSP and right heart hypertrophy (RV/LV + S)

Animals were anesthetized by intraperitoneal injection of pentobarbital (100 mg/kg). A left parasternal incision was made after mice were anesthetized. Then the ribs were partially resected, a 1.4-F microtip pressure transducer catheter (Millar Instruments) was carefully inserted into the right ventricular (RV), and right ventricular systolic pressure (RVSP) was continuously monitored for 5 min using a PowerLab data acquisition system (AD iInstruments) [20]. RV hypertrophy was evaluated in Fulton index measurements (weight of RV/LV + S), and determined according to the method described previously [21, 22].

### RNA extraction and high-throughput sequencing

RNA extraction and high-throughput sequencing were performed according previously methods [23]. RNAiso (Takara, Beijing, China) was utilized to extract total RNA from mice lung tissues. RNA integrity was evaluated by the Bio-analyzer 2100 system (Agilent Technology, CA, USA). Ribosome RNA was isolated from 3 μg of RNA using a commercially available RNA Removal Kit (Epicentre, WI, USA). Thereafter, the sequencing library was constructed. PCR products were purified and library qualification was detected. The library was sequenced using the illumina Hiseq 3500 platform to generate 150 bps long paired-end reads. Raw and clean data were obtained after filtering for quality control. Reading counts for every sample were analyzed using HTSeq v6.0. RPKM (reads per kilo base million mapped reads) and computed to estimate gene expression levels. The datasets generated and analyzed during the current study are available in the [GEO data, Series GSE128358] repository.

### Gene annotation and pathway identification

Gene Ontology (GO) was performed to determine the main functions of genes, lncRNAs, and their target genes. Biological pathways related to aberrantly expressed genes were analyzed based on KEGG database (http://www.genome.jp/kegg/) [24–26]. Benjamini-corrected *p* < 0.05 was used as the cut-off for significantly enriched biological processes.

### Co-expression analysis of lncRNA and mRNA

Cis and trans assays were performed to reveal the relationship between the predicted targets of DEGs and lncRNAs. The interaction network of lncRNA-mRNA co-expression pairs (COR ≥ 0.7 and *p* < 0.05) was then constructed using Cytoscape 3.0. A lncRNA-miRNA regulatory network was established by Star Base v2.0 to determine the functions of candidate lncRNAs after C75 treatment. Six lncRNA sequences were obtained from NCBI (Additional file 1: Table S1). The MiRanda software (https://www.miranda.software/contact) was then used to predict the possible binding events between lncRNAs and miRNAs by accepting predicted scores that were above 140 and energy below -20.

### CCK-8 and cell cycle assay

In vitro pulmonary hypertension was induced in pulmonary arterial smooth muscle cells (PASMCs) by incubating cells with hypoxia (3% O_2_, 5% CO_2_, 92% N_2_) for 24 h. PASMCs were purchased from the Chinese Academy of Sciences Cell Bank (Shanghai) and cultured with Smooth Muscle Cell Medium (SMCM) (ScienCell, California, USA). Cell Counting Kit 8 (CCK-8) was used to detect PASMCs proliferation. And cells at passages 3–6 were used in experiments. Briefly, PASMCs were grown in a 96-well plate under hypoxia for 24 h and treated with or without C75 (50 μg/mL). Culture medium (SMCM) was deleted, 100 μL of SMCM and 10 μL CCK-8 detection solutions were added to each well of the 96-well plate. Background control composed of 100 μL SMCM and 10 μL CCK-8 was added. After incubating in a 37 °C cell incubator for 2 h, the optical density of each well was read using a Thermo Scientific Microplate Reader (Thermo Fisher Scientific, USA) at 450 nm. The experiment was repeated at least three times. For cell cycle assay, Briefly, PASMCs were grown in a 6-well plate under hypoxia for 24 h and treated with or without C75 (50 μg/mL) for another 24 h. Subsequently, the supernatant was removed, the cells were washed with PBS twice, and then fixed with 1 mL precooled ethanol (70%) at 4 ℃ overnight. The cells were centrifuged at 1000 rpm for 5 min, incubated with 10 μL RNase (50×), 25 μL propidium iodide (20×) solution, and 500 mL buffer solution, stained in dark at 4 ℃ for 30 min, and then detected by flow cytometry. Each experiment was replicated at least three times.

### Quantitative real-time polymerase chain (RT-PCR) reaction

RT-PCR was carried out to validate the HTS results using SYBR Green assays. Assays were performed with 2 μL of cDNA in 20 μL reactions. The cycling conditions were: 95 °C for 10 min for initial denaturation and enzyme activation, followed by 40 cycles at 95 °C for 15 s and 60 °C for 1 min (Table 1). All primers used in this study are shown in Table 2.

**Table 1:** The animal model method was showed in the following table

Groups	Week 1	Week 2	Week 3	Week 4	Week 5
Control-vehicle	Vehicle + Vehicle	Vehicle	Vehicle	Vehicle	Vehicle
MCT-vehicle	MCT + Vehicle	Vehicle	Vehicle	Vehicle	Vehicle
MCT-C75	MCT + C75	C75	C75	C75	C75

**Table 2:** All primers used in this study are shown in the following table

Primer	Forward sequence	Reverse sequence
Hsd17b2	CAAGGCGTTTCTGCCTCTAC	GGTTAGAGCTGCCTTTGTGG
Retnlg	TGTCACTGGTTGTGCTTGTG	CCCAGTCCATTGTTGAGCAC
Mmp8	ACGCACCCTATGAGGACAAA	TGGCTGGGAATGCCAGATTA
S100a9	GCCAACAAAGCACCTTCTCA	TGTCAGGGTGTCCTTCCTTC
Ll1r2	TGGTGCGGACAATGTTCATC	ACGCACCCTATGAGGACAAA
S100a8	TTCGAGGAGTTCCTTGCGAT	AGCTCTGCTACTCCTTGTGG
Slfn4	AAGAGCTGGGCTTTGGATCT	GCGCCTAGTTTCCCAAGAAG
Ntrk2	ACACGAAACAAGCTGACGAG	CGGATTACCCGTCAGGATCA
Ckap2	TACACCTCGGCTGCAAAGTA	GGCAGTCGTGAAGTCTTGTC
Gm38850	CTTCCTGTATCGCCCAGGAT	CATAAATTGGGCGTGGCTGA
Gm41235	TGGATGTCACACCTGATGCT	TTGTGTGATGCCCAAACCTG
Mirt2	TGCGCTACCATCTTTGAACG	AACAGTGAGGGAGGAATGGG
GAPDH	GTCGGTGTGAACGGATTTGG	TGATGGGCTTCCCGTTGATG

### Statistical analyses

Differential expression analysis for any two groups was performed using the DESeq2 R package (1.26.0). A *P* value < 0.05 and fold Change ≥ 2 was set as the threshold for significantly differential expression. Student’s *t*-test, one-way ANOVA, and Pearson’s correlation were performed using SPSS (version 22). *P*-values less than 0.05 were considered statistically significant.

## Results

### Morphological and pathological studies

**Fig. 1:**
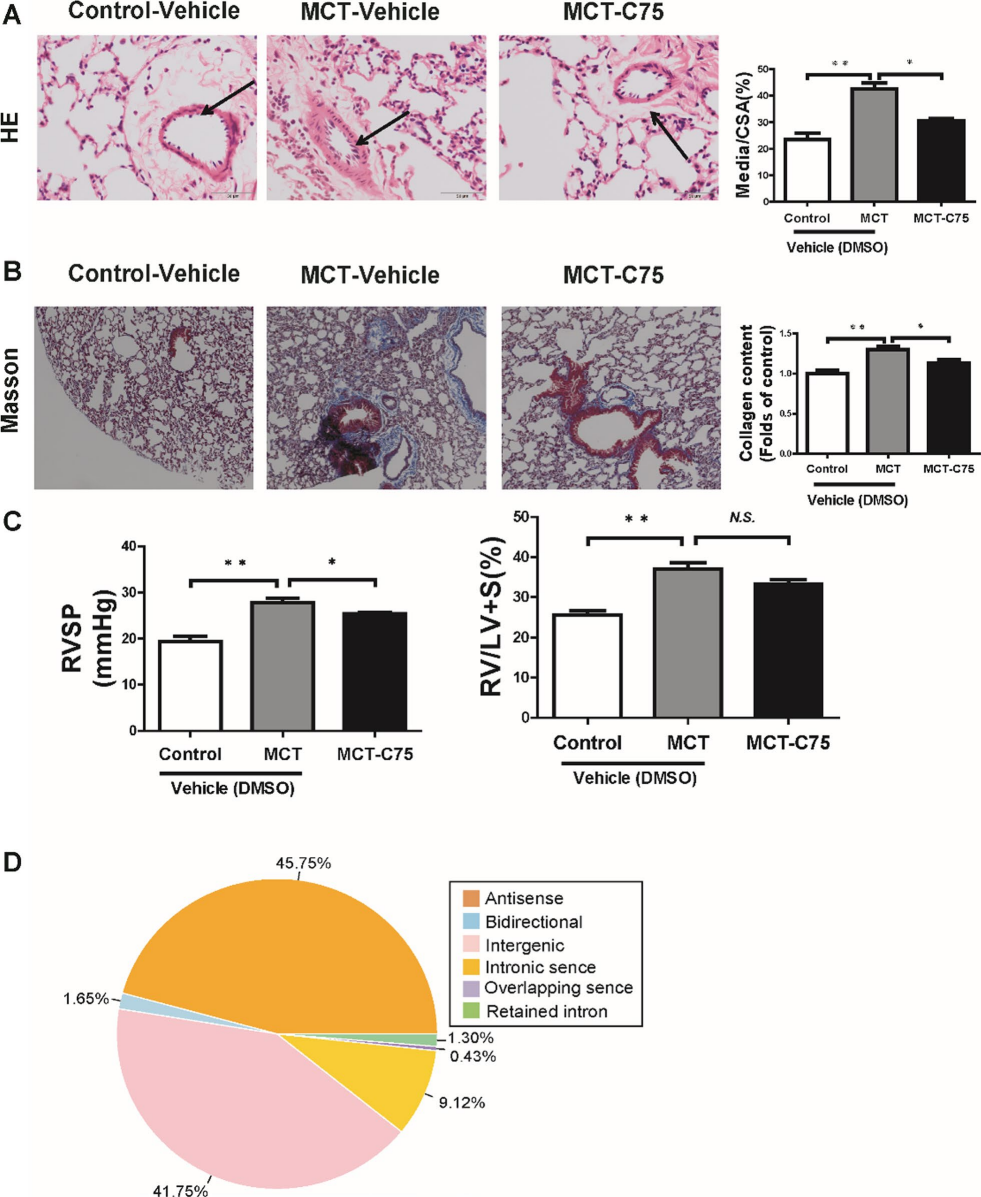
HE and Masson staining of lung tissues of MCT -induced PAH mice. Scale bars = 50 μm. **A** Representative graphs of HE staining and quantification of the ratio of vascular medial thickness of total vessel size (Media/cross-sectional area [CSA]) for the PAH model (n = 6). **B** Representative graphs of Masson staining and collagen contents statistics (n = 6). **C** RVSP and ratio of RV/LV + S in mice after exposure to monocrotaline (n = 7). **D** These lncRNAs were classified into antisense lncRNAs, bidirectional lncRNAs, intergenic lncRNAs, intrinsic sence lncRNAs, overlapping sence lncRNAs, and retained intron lncRNAs. Right ventricular systolic pressure (RVSP); Hematoxylin–eosin (HE); Monocrotaline (MCT)

The animal model dosing strategy is shown in Table 1. MCT injection induced pulmonary artery remodeling, while C75 (50 μg/kg/week) administration partially inhibited the ratio of vascular medial thickness of total vessel size (Fig. 1A). Likewise, mouse collagen content was significantly increased after MCT treatment, and partially reduced after C75 administration (Fig. 1B). In line with the morphological change, we also observed a significant increasing of the right ventricular systolic pressure (RVSP) and the ratio of right ventricular wall weight to left ventricular wall plus septum (RV/LV + S) following MCT injection. C75 reduced the increase of RVSP due to the MCT effect, but such effect was not significant in RV/LV + S (Fig. 1C).

### Characteristics of mRNAs and lncRNAs

**Fig. 2:**
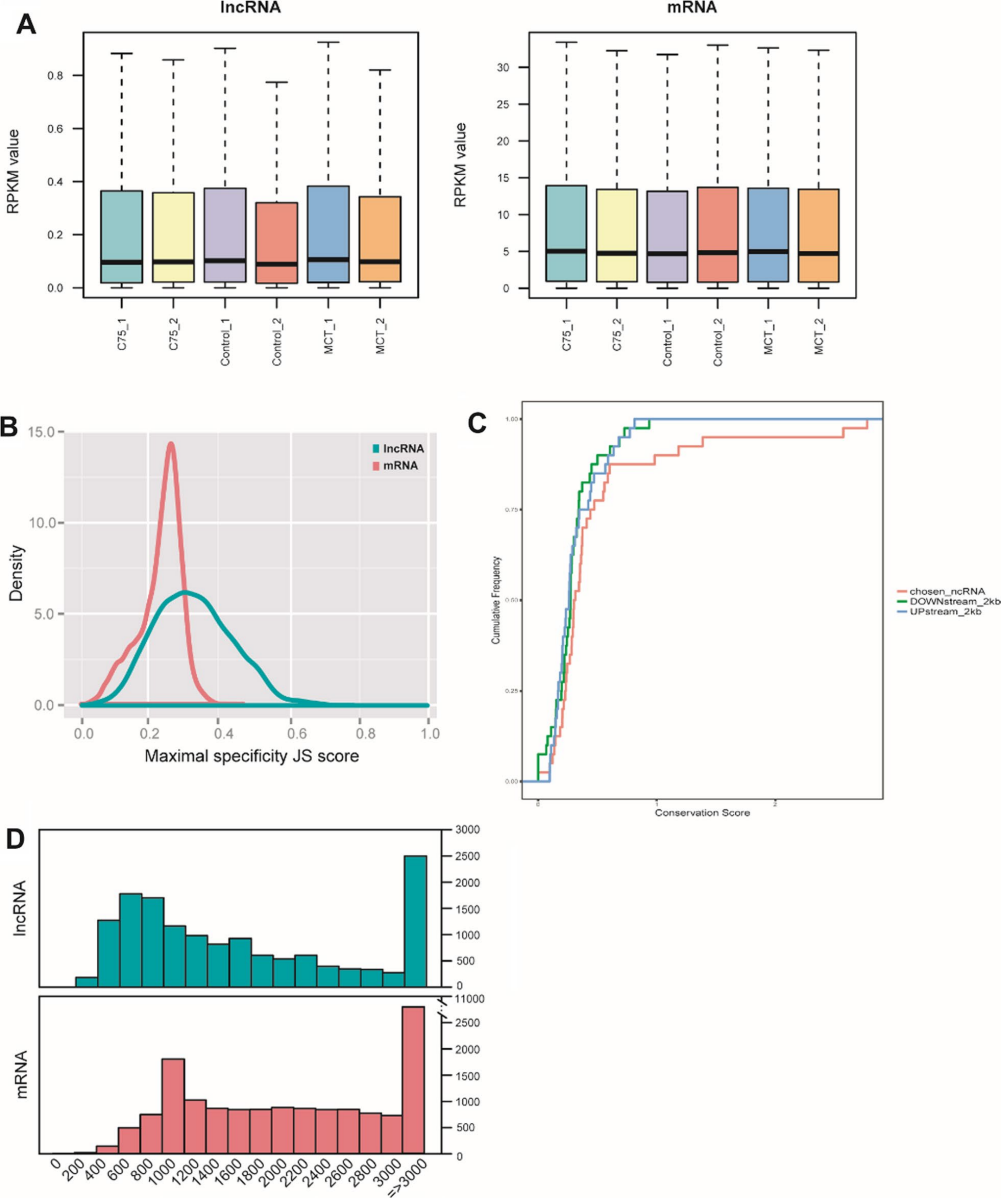
Comparison of mRNA and lncRNA characteristics. **A** RPKM distribution of mRNAs and lncRNAs. **B** JS score distribution of mRNAs and lncRNAs. **C** Conservation scores for two individual subtypes of mRNAs and lncRNAs. **D** Transcript lengths of mRNAs and lncRNAs

The raw and clean data were submitted to the GEO repository (series record GSE128358). A total of 9,082 lncRNA transcripts were selected by intersecting coding potential calculator, coding non-coding index, and protein family database. These lncRNAs were classified into antisense lncRNAs (45.75%), bidirectional lncRNAs (1.65%), intergenic lncRNAs (41.75%), intrinsic sence lncRNAs (9.12%), overlapping sence lncRNAs (0.43%), and retained intron lncRNAs (1.3%) (Fig. 1D). Compared to mRNAs, lncRNAs exhibited much lower transcript abundance (Fig. 2A), higher tissue specificity (Fig. 2B), and less conservative (Fig. 2C). The lncRNAs identified are over 200 bps, containing more than 2 exomes (Fig. 2D). These properties are consistent with lncRNA traits.

### Profiles of mRNAs and lncRNAs

**Fig. 3:**
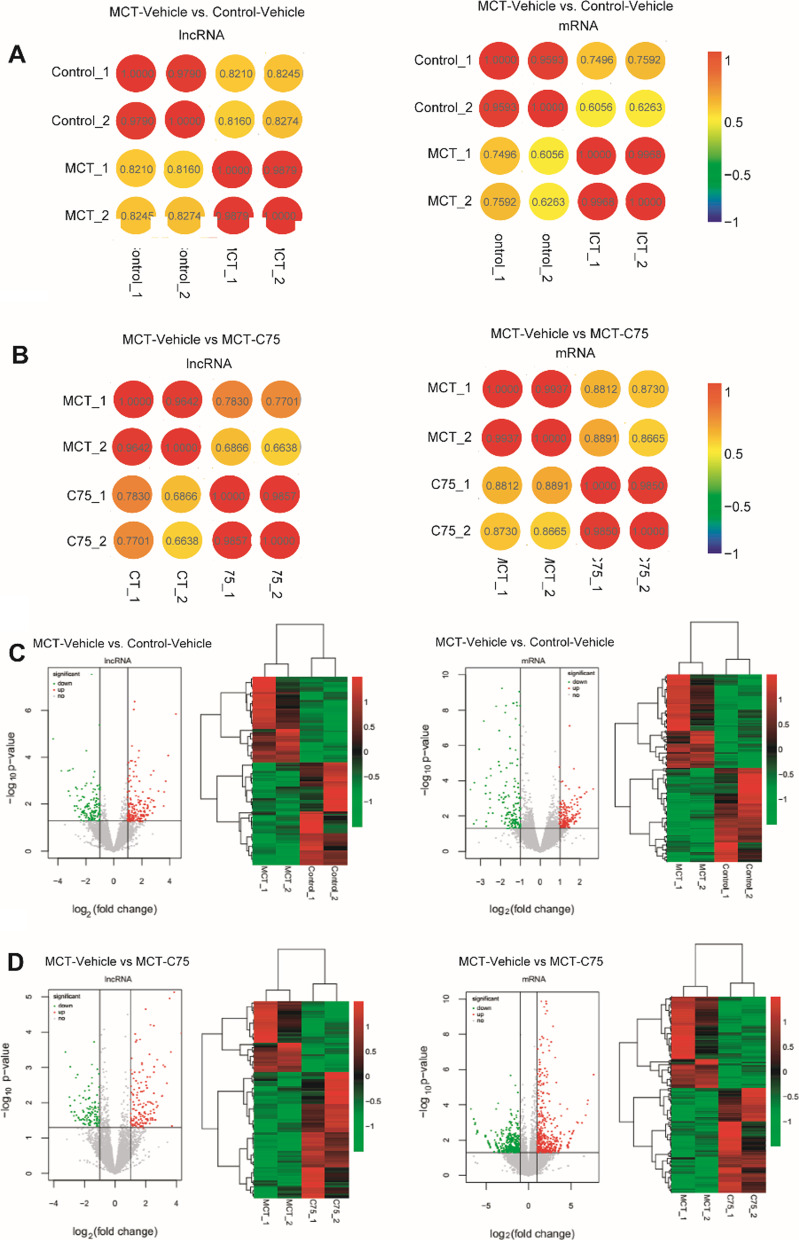
Aberrantly expressed genes in two-two comparison samples. **A** The possible correlation between aberrantly expressed lncRNA profiles among the three groups: MCT-Vehicle Vs Control-Vehicle, MCT-Vehicle Vs MCT-C75. **B** The possible correlation between aberrantly expressed mRNA profiles among the three groups: MCT-Vehicle Vs Control-Vehicle, MCT-Vehicle Vs MCT-C75. **C**, **D** Volcano plot and hierarchically clustered heat map illustrating differentially expressed lncRNAs and miRNAs among the three groups: MCT-Vehicle Vs Control-Vehicle, MCT-Vehicle Vs MCT-C75. Upregulated mRNAs are shown in red while downregulated mRNAs are shown in green (fold change ≥ 2 and *p* ≤ 0.05)

**Fig. 4:**
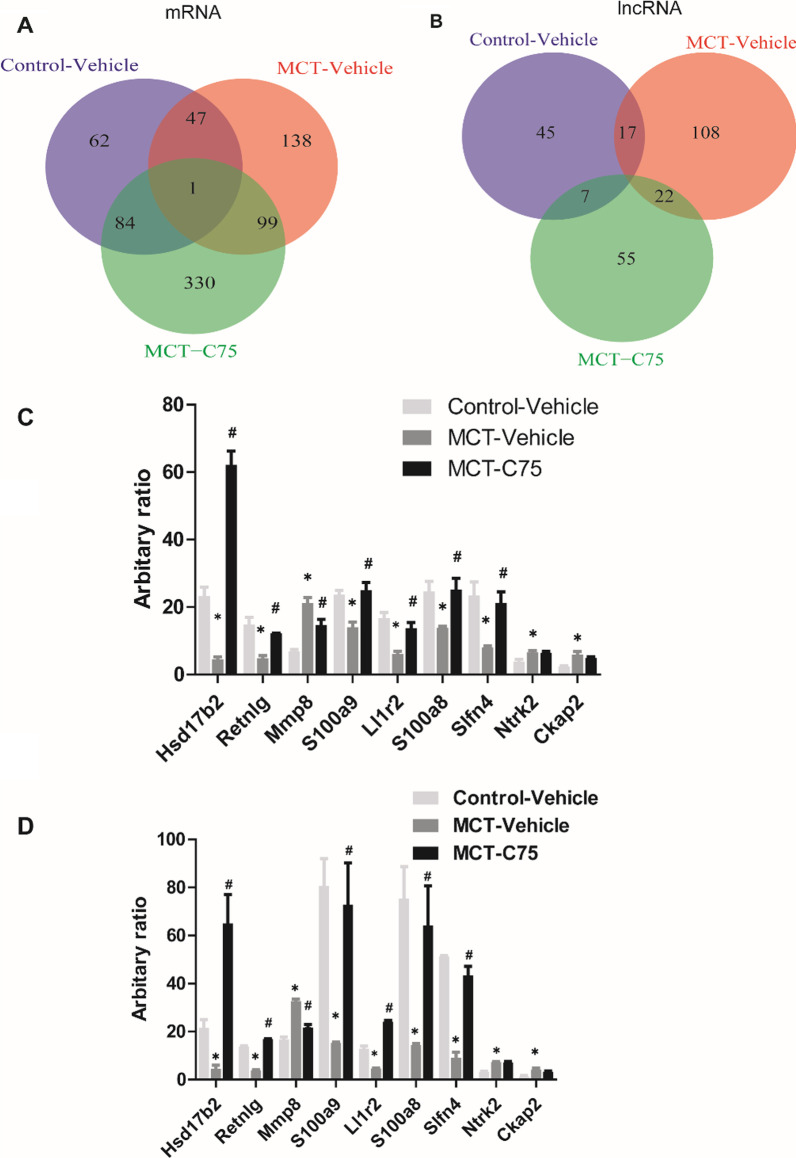
Venn diagram analysis and RT-PCR validation of these dysregulated mRNAs. **A** Venn diagram of mRNAs among the three groups and only one of the mRNAs Hsd17b2 was decreased in the MCT-Vehicle group, but return to normal level after C75 intervention. **B** Venn diagram of lncRNAs among the three groups and not one lncRNA altered among them. **C** RT-PCR validation of dysregulated mRNAs. **D** Dysregulated mRNAs after the HTS analysis. N = 6, *p < 0.05

Volcano plots were plotted using gglot2 package to demonstrate gene/lncRNA expression based on RPKM among the three groups. Conservation analysis was performed and JS score was calculated. Correlations between mRNA and lncRNA profiles were significantly higher within groups than inter*-*group correlations (Fig. 3A, B), suggesting that the results are highly reliable. Compared to the control group, 285 mRNAs (113 upregulated and 172 downregulated) and 147 lncRNAs (82 upregulated and 65 downregulated) were differentially expressed in the MCT-Vehicle group. Top 10 deregulated mRNAs and lncRNAs are presented in Tables 3 and 4, respectively. After five weeks of C75 treatment, 514 mRNAs (401 upregulated and 113 downregulated) and 84 lncRNAs (35 upregulated and 49 downregulated) were aberrantly expressed in the MCT-C75 group compared to the MCT-Vehicle group. Top 10 disordered mRNAs and lncRNAs are shown in Tables 5 and 6, respectively. Heat maps and volcano plots illustrated the expression profiles of mRNAs and lncRNAs after MCT and C75 treatment (Fig. 3C, D). Venn diagram showed that only one mRNA, Hsd17b2, was decreased in the MCT-Vehicle group, but returned to normal level after C75 intervention (Fig. 4A); not one lncRNA altered among them (Fig. 4B). The numbers in the non-overlapping sections of the diagram represent the number of genes unique to each group (Fig. 4A, B). Aberrantly expressed mRNAs (Hsd17b2, Retnlg, Mmp8, S100a9, Ll1r2, S100a8, Slfn4, Ntrk2, and Ckap2) were selected and validated using RT-PCR assay (Fig. 4C). Based on the RT-PCR results, six genes exhibited lower expression, while three genes exhibited higher expression in the MCT-Vehicle group. These effects were partially reversed by C75 treatment. The RT-PCR results were consistent with the HTS (Fig. 4D).

**Table 3:** Top 10 up-regulated and down-regulated lncRNAs (MCT-vehicle vs. control-vehicle)

Symbol	Chromosomal position	Control-vehicle normalize	MCT-vehicle normalize	log_2_ FC	*p*-value	Up/down
ENSMUSG00000112441.1	chr12:8,291,024–8,293,694	3	32	3.2915026	0.0030079	Up
ENSMUSG00000107605.1	chr6:90,459,711–90,461,468	4	27.5	2.7232757	0.0040755	Up
ENSMUSG00000112647.1	chr10:98,653,799–98,699,710	7	37.5	2.3810823	0.0192184	Up
ENSMUSG00000109957.1	chr8:122,339,171–122,343,100	3	26.5	2.9411917	0.0240806	Up
ENSMUSG00000114105.1	chr12:25,300,071–25,304,613	4	21.5	2.3530836	0.0246912	Up
ENSMUSG00000110290.1	chr8:92,465,767–92,469,665	4.5	27	2.6516029	0.0311117	Up
ENSMUSG00000097219.1	chr11:51,949,882–52,269,514	40	127.5	1.4952014	0.035351	Up
ENSMUSG00000098001.1	chr8:80,933,534–80,938,667	8	34	2.0443405	0.0353627	Up
Vaultrc5	chr18:36,801,763–36,802,107	248.5	683.5	1.4588887	1.41E−06	Up
LOC105246895	chr11:120,235,184–120,239,123	10.5	66	2.6002823	5.38E−05	Up
Gm40799	chr10:127,107,362–127,113,573	2.5	41.5	3.9511874	7.10E−05	Up
ENSMUSG00000097554.1	chr5:5,781,530–5,783,636	225.5	79	−1.4568222	0.0072437	Down
ENSMUSG00000111250.1	chr9:77,836,030–77,848,111	33	9.5	−1.8350256	0.016356	Down
ENSMUSG00000107736.1	chr6:121,255,203–121,272,685	69	30	−1.2331487	0.0164294	Down
ENSMUSG00000097576.1	chr16:38,451,982–38,453,493	42	7.5	−2.5782671	0.0192785	Down
ENSMUSG00000087340.1	chrX:167,164,097–167,171,278	18.5	3	−2.7450978	0.0355979	Down
ENSMUSG00000103170.1	chr3:52,198,248–52,200,489	28.5	7.5	−1.8897878	0.0411948	Down
Gm41235	chr14:103,043,478–103,048,082	348.5	53	−2.8783353	3.65E−07	Down
1600010M07Rik	chr7:109,998,377–110,006,646	100.5	29	−1.8322912	7.38E−05	Down
Gm30286	chr2:131,014,185–131,025,161	379.5	84.5	−2.105982	0.0002835	Down
Gm13605	chr2:35,223,695–35,254,166	31	2	−3.9802018	0.0003077	Down

**Table 4:** Top 10 up-regulated and down-regulated lncRNAs (MCT-C75 vs. MCT-Vehicle)

Symbol	Chromosomal position	MCT-vehicle normalize	MCT-C75 normalize	log_2_ FC	*p*-value	Up/down
ENSMUSG00000102196.1	chr2:11,315,372–11,319,874	21	103	2.3662741	3.45E−06	Up
ENSMUSG00000100465.1	chr5:149,234,816–149,247,682	1.5	47.5	5.0271267	4.48E−06	Up
ENSMUSG00000087340.1	chrX:167,164,097–167,171,278	3	34	3.5396281	0.0011068	Up
ENSMUSG00000108197.1	chr6:86,687,823–86,688,428	0.5	18.5	5.2597749	0.0031102	Up
ENSMUSG00000085532.1	chr11:63,173,914–63,188,454	12	43.5	1.9194104	0.0040397	Up
ENSMUSG00000108308.1	chr6:91,599,232–91,605,514	4.5	25	2.5651125	0.0165033	Up
ENSMUSG00000105560.1	chr5:4,784,753–4,799,094	11.5	36	1.7164354	0.019711	Up
ENSMUSG00000105699.1	chr5:140,605,789–140,606,551	3.5	19	2.4695539	0.0491302	Up
Gm41235	chr14:103,043,478–103,048,082	53	359	2.8386814	1.35E−12	Up
Gm38850	chr6:86,653,954–86,663,064	888	2212.5	1.3850536	1.47E−12	Up
ENSMUSG00000110399.1	chr8:25,443,668–25,454,107	21.5	2	−3.3533301	0.0058612	Down
ENSMUSG00000107689.1	chr6:86,513,891–86,516,273	19	2	−3.1682999	0.0127772	Down
ENSMUSG00000110393.2	chr13:65,241,753–65,250,154	142	62	−1.1271112	0.0132598	Down
ENSMUSG00000109028.1	chr7:70,548,869–70,554,924	40.5	10	−1.9304727	0.0185882	Down
ENSMUSG00000098061.1	chr12:109,640,341–109,642,351	47.5	12.5	−1.9088353	0.0276344	Down
ENSMUSG00000096983.1	chr11:88,039,578–88,047,360	32	9	−1.7770616	0.0306435	Down
ENSMUSG00000096984.1	chr1:163,301,790–163,303,620	22.5	3	−2.8080874	0.0323113	Down
ENSMUSG00000112433.1	chr10:78,248,880–78,256,111	20.5	3	−2.6735213	0.0334734	Down
ENSMUSG00000111868.1	chr10:44,851,393–44,866,786	20	4.5	−2.0949938	0.0376827	Down
ENSMUSG00000085411.1	chr2:167,858,585–167,862,542	28	6	−2.173601	0.0388288	Down

**Table 5:** Top 10 up-regulated and down-regulated mRNAs (MCT-Vehicle vs. Control-Vehicle)

Symbol	Chromosomal position	Control-vehicle normalize	MCT-vehicle normalize	log_2_ FC	*p*-value	Up/down
Ttn	chr2:76,703,980–76,982,557	8935	22,213.5	1.2427283	1.89E−10	Up
Ntrk2	chr13:58,806,569–59,133,970	390	860.5	1.0885439	1.31E−08	Up
Kcnc3	chr7:44,590,661–44,604,751	435	916.5	1.0262368	1.32E−08	Up
Ckap2	chr8:22,168,149–22,185,819	147.5	484.5	1.7217981	4.14E−07	Up
Iqgap3	chr3:88,082,051–88,121,048	215	581	1.4181361	1.39E−06	Up
Gtse1	chr15:85,859,690–85,876,573	30.5	150.5	2.3038095	8.95E−06	Up
Npas2	chr1:39,193,715–39,363,240	365	808	1.0730423	4.26E−05	Up
Gm9780	chr14:26,027,782–26,042,963	66.5	214.5	1.6404044	5.19E−05	Up
Chsy3	chr18:59,175,340–59,411,336	160.5	356	1.1008269	7.13E−05	Up
Wisp1	chr15:66,891,325–66,923,205	86.5	239	1.3969618	0.0001027	Up
S100a9	chr3:90,692,630–90,695,721	9514.5	1805	−2.4365459	2.38E−95	Down
S100a8	chr3:90,669,071–90,670,034	8895	1695.5	−2.4247112	4.61E−85	Down
Slfn4	chr11:83,175,172–83,190,216	6051.5	1077.5	−2.5706601	5.37E−30	Down
Mmp8	chr9:7,558,429–7,568,486	3691.5	850.5	−2.1926784	1.38E−28	Down
Retnlg	chr16:48,872,608–48,874,498	1614.5	460.5	−1.8714284	3.38E−25	Down
Dhrs9	chr2:69,380,462–69,403,086	981.5	177.5	−2.5533365	9.77E−21	Down
Ccr1	chr9:123,962,126–123,968,692	2904	1032.5	−1.5605265	9.39E−18	Down
Slc2a3	chr6:122,727,809–122,802,274	2285	947	−1.3191384	9.23E−17	Down
Sirpb1b	chr3:15,495,754–15,575,067	811.5	233.5	−1.8636476	9.84E−16	Down
Clec4e	chr6:123,281,789–123,289,871	1141.5	209.5	−2.5408385	1.18E−15	Down

**Table 6:** Top 10 up-regulated and down-regulated mRNAs (MCT-C75 vs. MCT-Vehicle)

Symbol	Chromosomal position	MCT-vehicle normalize	MCT-C75 normalize	log_2_ FC	*p*-value	Up/down
Il1r2	chr1:40,083,308–40,125,230	545	2848.5	2.4452348	3.97E−84	Up
Mmp8	chr9:7,558,429–7,568,486	850.5	4327.5	2.4055314	2.67E−74	Up
Retnlg	chr16:48,872,608–48,874,498	460.5	1990	2.1717867	3.98E−56	Up
Clec4d	chr6:123,262,107–123,275,268	355	1639.5	2.262501	1.62E−37	Up
Slfn4	chr11:83,175,172–83,190,216	1077.5	5131.5	2.3228931	8.67E−34	Up
Fgg	chr3:83,007,724–83,015,056	911.5	2932.5	1.7434494	2.83E−27	Up
H2-Q10	chr17:35,470,089–35,474,563	303	1125.5	1.9528828	1.04E−26	Up
Serpina1e	chr12:103,946,931–103,956,897	1536	3686	1.317478	8.71E−26	Up
Fgb	chr3:83,042,246–83,049,863	759	2608	1.8304191	3.37E−24	Up
Clec4e	chr6:123,281,789–123,289,871	209.5	992	2.2964289	5.01E−24	Up
Slc7a10	chr7:35,186,352–35,201,116	1140	512	−1.089346	6.39E−09	Down
Npas2	chr1:39,193,715–39,363,240	808	296.5	−1.3756141	1.05E−08	Down
Hcn1	chr13:117,602,320–117,981,028	67.5	15	−2.1064787	0.0002332	Down
Sbspon	chr1:15,853,862–15,892,722	258	103.5	−1.2465895	0.0004178	Down
A730017C20Rik	chr18:59,062,181–59,076,962	27.5	1	−4.7317615	0.0005319	Down
M1ap	chr6:82,946,908–83,030,309	100	33.5	−1.5235042	0.0005978	Down
Kctd19	chr8:105,382,807–105,413,502	53	12.5	−2.0154139	0.0016237	Down
Zfp239	chr6:117,863,077–117,872,766	67	19	−1.7555554	0.0017625	Down
Trpm3	chr19:22,137,798–22,989,897	75.5	25	−1.5348774	0.0021623	Down
Sycp1	chr3:102,818,499–102,936,100	70	20.5	−1.7157192	0.0021941	Down

### GO and KEGG analyses

**Fig. 5:**
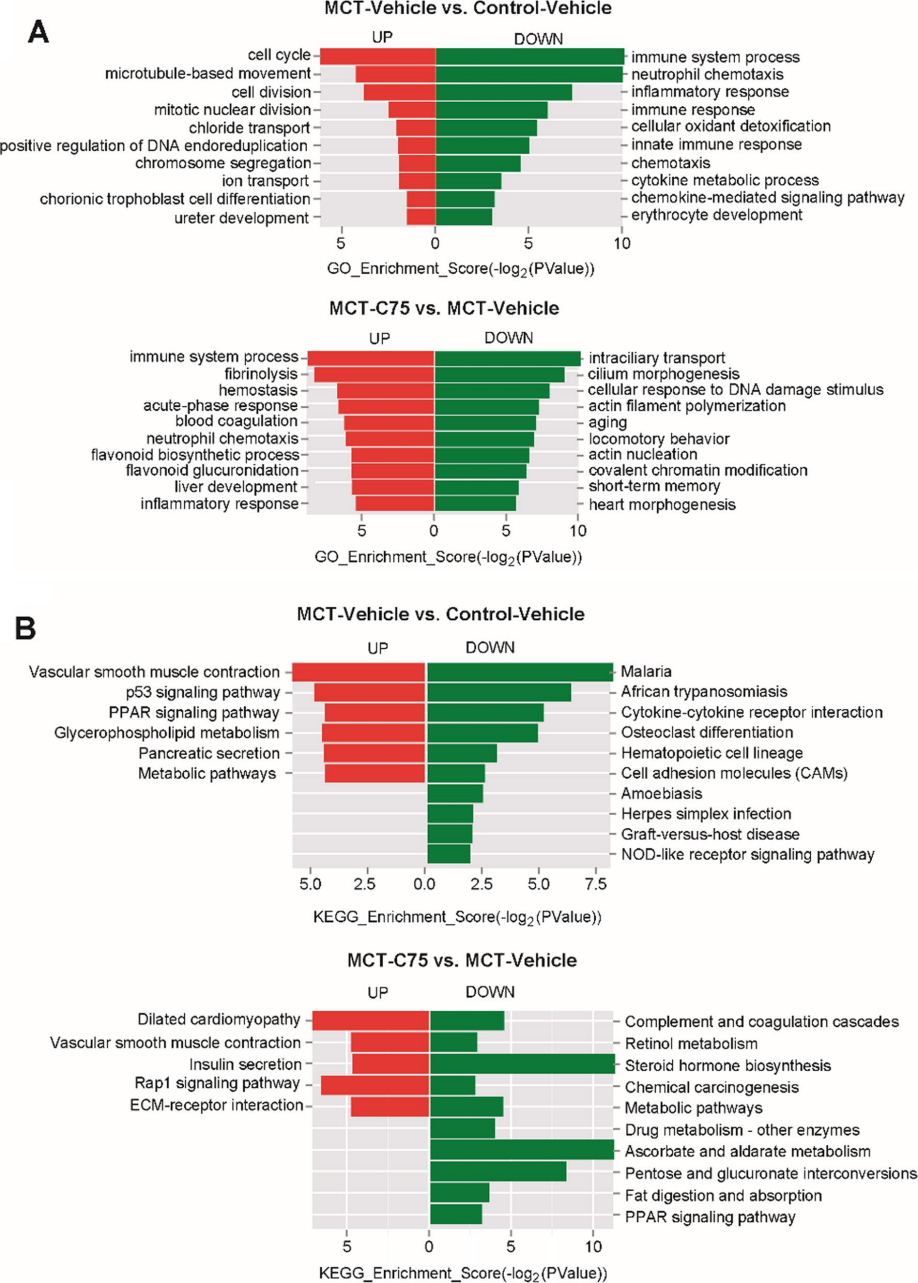
GO and KEGG analyses for mRNAs. **A** GO analyses for mRNAs (Top 10 if enriched terms were greater than 10). **B** KEGG analyses for mRNAs (Top 10 if enriched terms were greater than 10)

**Fig. 6:**
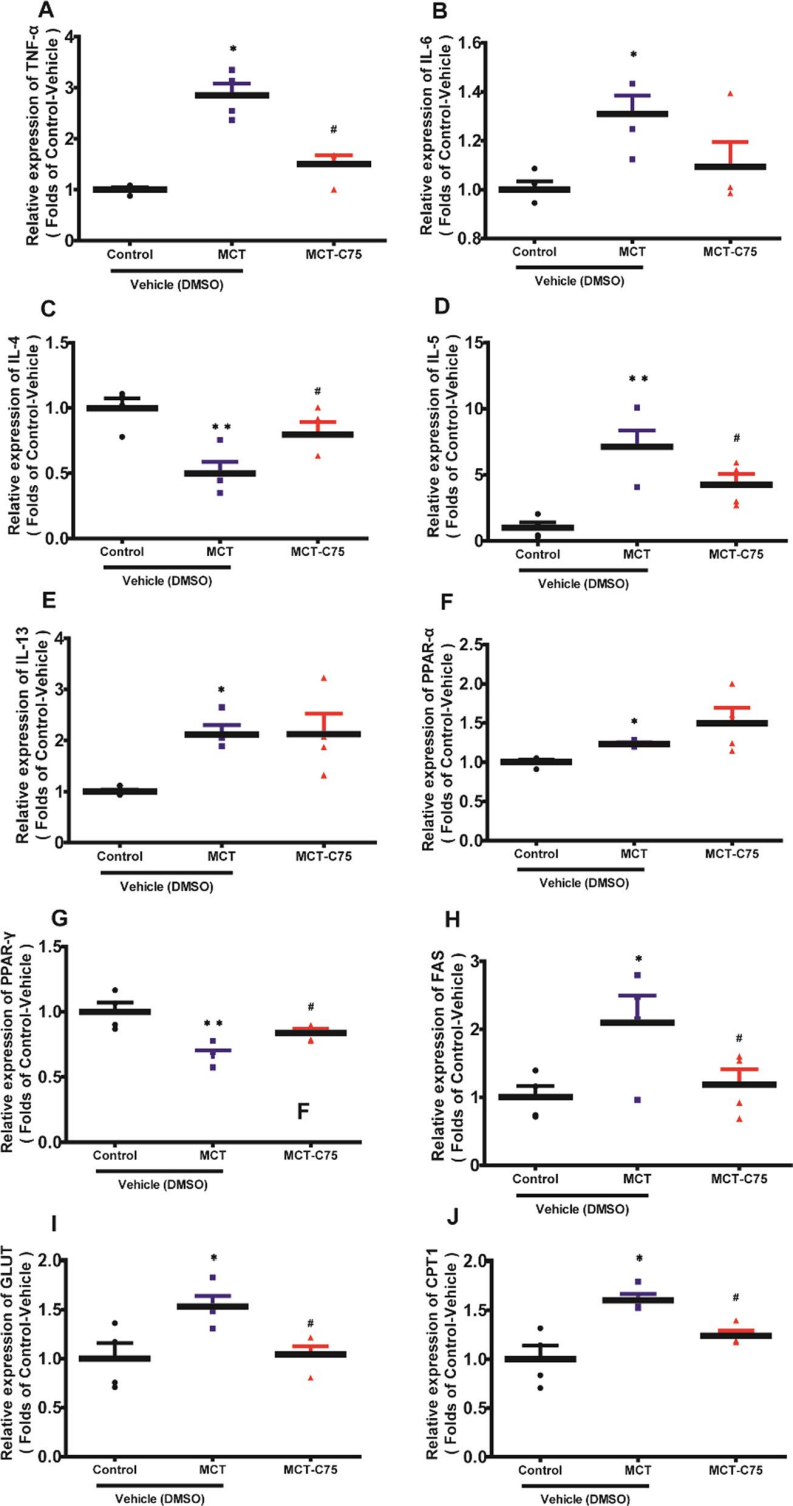
The protective effects of C75 on the PAH mice. **A**–**J** The relative mRNA levels of TNF-α, IL-6, IL-4, IL-5, IL-13, PPAR-α, PPAR-γ, FAS, GLUT, and CPT1in the PAH mice. *P < 0.05

Both GO and KEGG analyses were performed to explore the functions of aberrantly expressed mRNAs. Top 10 GO terms are illustrated in Fig. 5. GO analysis revealed that up-regulated mRNAs between the MCT-Vehicle and control group were primarily associated with the cell cycle, microtubule-based movement, cell division, and mitotic nuclear division. Down-regulated mRNAs were mainly associated with neutrophil chemotaxis, inflammatory response, and immune response (Fig. 5A). Up-regulated mRNAs between the MCT-C75 and MCT-Vehicle were centered on fibrinolysis, hemostasis, and acute-phase response. Down-regulated mRNAs were involved in intracellular transport, cilium morphogenesis, cellular response to DNA damage stimulus, and actin filament polymerization (Fig. 5A). KEGG analysis revealed that up-regulated mRNAs were mainly concentrated in p53 signaling pathway, PPAR signaling pathway, glycerophospholipid metabolism, pancreatic secretion, and metabolic pathways. Down-regulated mRNAs were involved with malaria, African trypanosomiasis, cytokine-cytokine receptor interaction, osteoclast differentiation, hematopoietic cell lineage, and cell adhesion molecules (Fig. 5B). GO and KEGG analysis indicated that the inflammatory response may play crucial roles in the PAH, we used RT-PCR assay to detect the pro-inflammation, anti-inflammation signaling and metabolic pathways. Compared to the control group, mRNA levels of TNF-α, IL-5, IL6, and IL-13 were increased. And C75 treatment can partially decrease TNF-α, IL-5 (Fig. 6A-E). PPAR-α mRNA level was increased in the MCT-treated mouse, C75 treatment has little effect on its expression (Fig. 6F). While IL-4 and PPAR-γ mRNA levels were decreased in the MCT-treated mouse, C75 treatment can partially increase their expression (Fig. 6C, G). We also detected FAS, CPT1, and GLUT mRNA levels, and found that C75 treatment can partially reverse their increase induced by MCT (Fig. 6H–J). The RT-PCR results indicated that inflammatory was activated in the PAH model, while C75 treatment can reverse the inflammatory partially.

### Functional prediction of mRNAs regulated by aberrantly expressed cis- and trans-acting lncRNAs

**Fig. 7:**
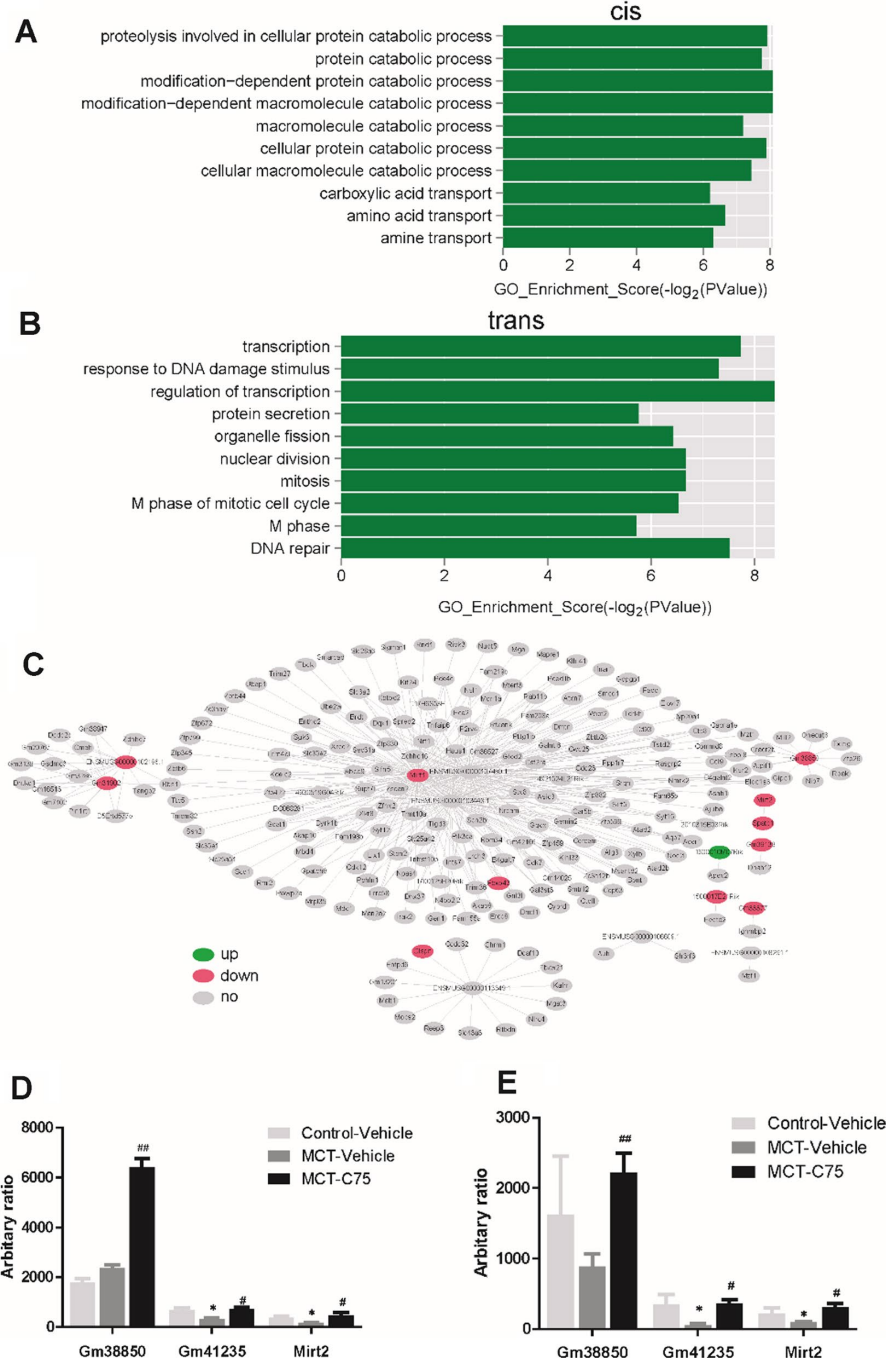
Target genes regulated by aberrantly expressed lncRNAs (in cis and trans) in C75-treated mice, compared with the MCT-Vehicle group. **A** GO analyses of mRNAs regulated by lncRNAs in cis was forecast. **B** GO analyses mRNAs regulated by lncRNAs in trans was predicted. **C** LncRNA-mRNA regulatory network. **D** RT-PCR validation of dysregulated lncRNAs which were identified by the HTS. **E** Dysregulated lncRNAs after the HTS analysis. N = 6, *P < 0.05

GO analysis was performed to examine the function of mRNAs regulated by lncRNAs in cis and trans. Compared to the MCT-Vehicle group, aberrantly expressed lncRNA in C75-treated mice are shown in Fig. 7. The mRNAs targeted in cis by aberrantly expressed lncRNAs are involved in multiple biological processes, such as cellular protein catabolic processes, modification-dependent protein catabolic processes, and macromolecule catabolic processes (Fig. 7A). Disordered mRNAs targeted in trans by aberrantly expressed lncRNAs are associated with various biological processes, such as regulation of transcription, response to DNA damage stimulus, protein secretion, organelle fission, mitosis, cell cycle, and DNA repair (Fig. 7B). Moreover, lncRNA-mRNA network analysis exhibited the possible relationship in trans (Fig. 7C). Based on RT-PCR results, lncRNAs Gm41235 and Mirt2 exhibited lower expression in the MCT-Vehicle group compared to the control group. It was observed that the expression of lncRNAs Gm41235 and Mirt2 was partially rescued after C75 treatment (Fig. 7D). And these RT-PCR results were consistent with the HTS (Fig. 7E). The Gm38850 level was a bit increased in the MCT-Vehicle (there was no significance between the Control-Vehicle and MCT-Vehicle), and further increased after C75 treatment (Fig. 7D). As the RT-PCR results showed the Gm38850 mRNA level was decreased in the MCT-Vehicle (there was no significance between the Control-Vehicle and MCT-Vehicle), after C75 treatment (Fig. 7D), and was partially rescued after C75 treatment (Fig. 7E).

### Co-expression networks

A comprehensive analysis of lncRNAs in lung tissues was carried out to understand the possible impacts of lncRNAs on PAH. A number of lncRNAs were aberrantly expressed after C75 treatment. A co-expression network (protein-coding genes and lncRNAs) was constructed to identify the potential functions and regulatory mechanisms of lncRNAs (Additional file 4: Figure S1).

### Network of lncRNAs and miRNAs

**Fig. 8:**
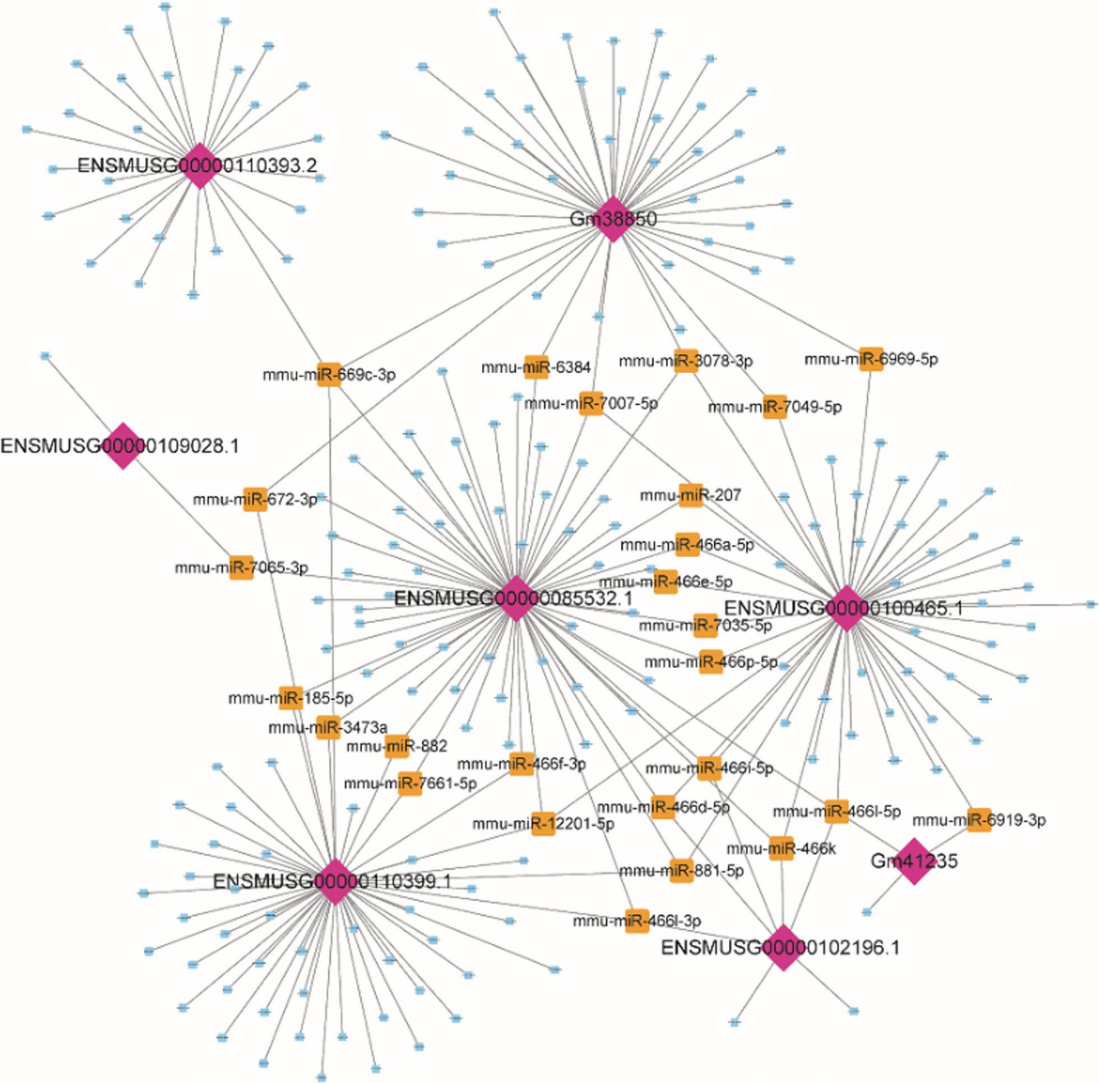
Regulation network of miRNA-lncRNA. Purple diamonds represent the six lncRNAs. A lncRNA with two or more than two regulation networks overlapping with those of miRNAs are marked with yellow. A lncRNA with only one regulation network overlapping with that of miRNA is indicated by blue

It is known that lncRNAs and mRNAs have similar sequences and can be linked to a common miRNA. When lncRNAs bind to miRNA, upregulated lncRNAs act as competing endogenous RNAs, which prevent miRNAs from binding to untreated mRNA targets, thereby increasing their expression at post-transcriptional levels [27, 28]. According to the predicted score of > 140, and energy ≤ 20, six lncRNAs and 1623 miRNAs that met these criteria were selected (Additional file 2: Table S2). Thereafter, 259 lncRNA-miRNA relationship pairs (at least five miRNA binding events), including six lncRNAs and 221miRNA (Additional file 3: Table S3) were filtered. The constructed lncRNA-miRNA network revealed the relationships between six abnormally expressed lncRNAs and 221 potential target miRNAs (Fig. 8).

### PASMC cell proliferation and cell cycle following C75 treatment

**Fig. 9:**
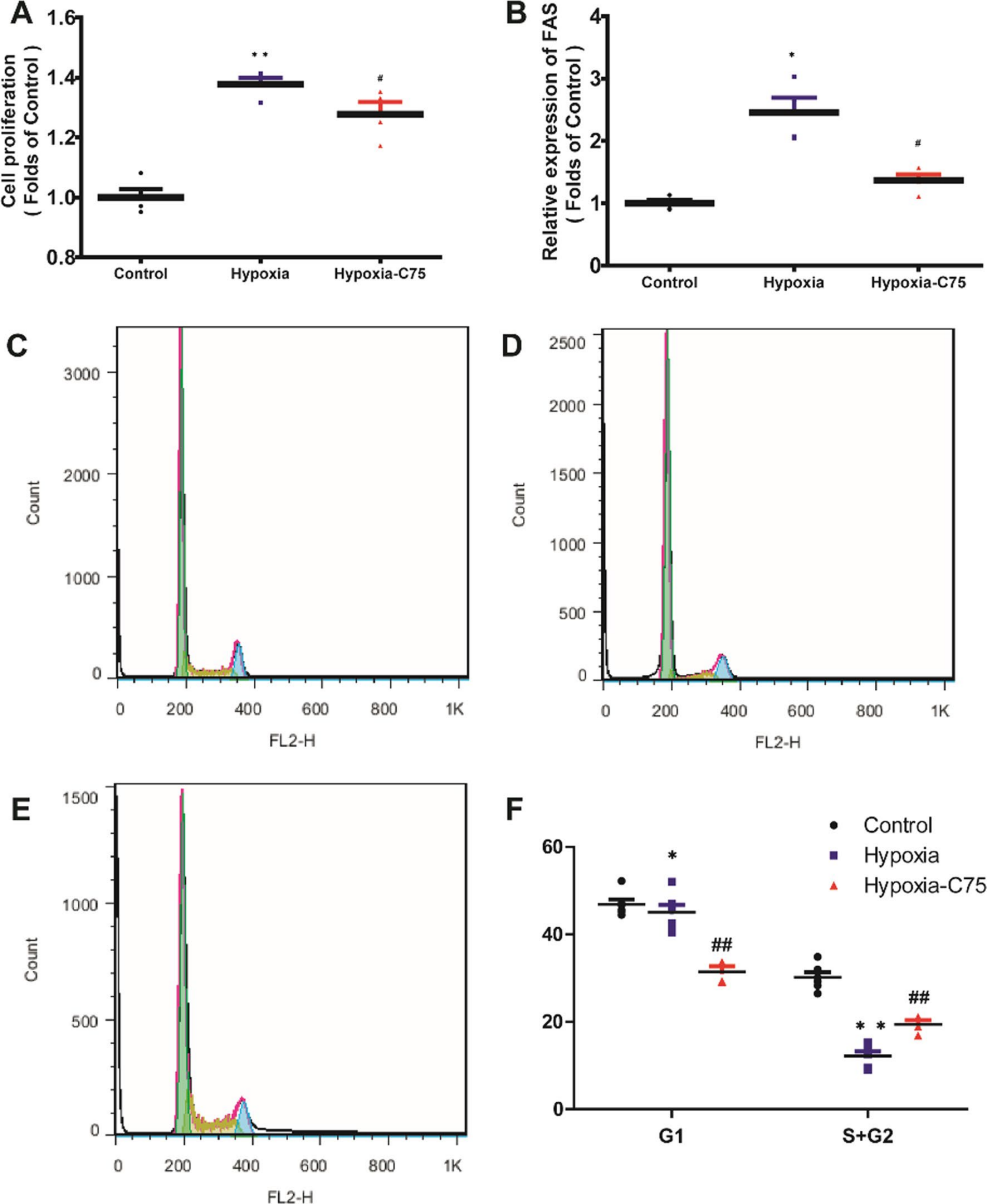
PASMCs cell proliferation and cell cycle after hypoxia and treatment. **A** CCK-8 results of the PAMSCs. **B** The relative mRNA level of FAS in the PAMSCs. **C** Representative PASMCs cell cycle illustrations of the Control group. **D** Representative PASMCs cell cycle illustrations of the hypoxia group. **E** Representative PASMC cell cycle illustrations of the hypoxia-C75 group. **F** Statistical graph of the PASMCs cell cycle. G1 means the proportion of all the PAMSCs that are in the G1 phase. S + G2 means the proportion of all the PAMSCs that are in the S and G2 phase. *P < 0.05

The PAMSCs cell proliferation ability was significantly increased under hypoxia condition, and its proliferation ability was decreased after incubation with C75 (50 μg/mL, 24 h) (Fig. 9A). FAS mRNA expression was increased in hypoxia-induced, and C75 incubation could inhibit hypoxia-induced FAS increase (Fig. 9B). Based on the flow cytometry assay, G1 phase duration was marginally reduced in hypoxia compared to that of control (Fig. 9C–F). The ratio of S and G2 phase (S + G2) was also decreased in hypoxia, and was partially reversed after incubation with 50 μg/mL of C75 for 24 h (Fig. 9C–F).

**Fig. 10:**
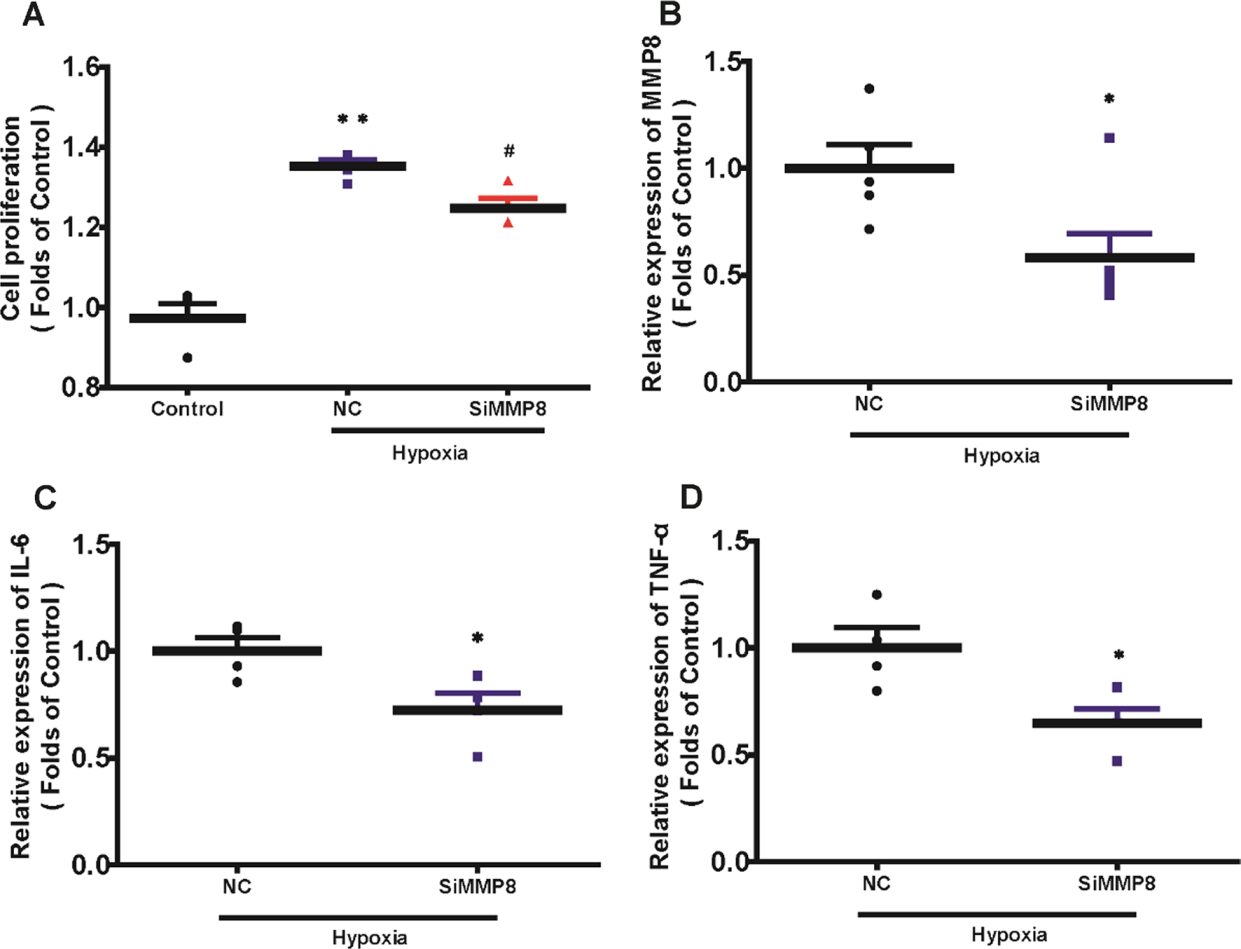
The protective effects of MMP8 on the PAMSCs cell proliferation and inflammation. **A** The effects of MMP8 on the PAMSCs cell proliferation were detected by CCK-8 assay. **B** The relative mRNA level of MMP8 in the PAMSCs cell. **C**, **D** The relative mRNA levels of IL-6, TNF-α after MMP8 knockdown in the PAMSCs. *P < 0.05

As MMP8 plays a crucial role in the PAH, and is widely expression in PAMSCs, we knockdown MMP8 in order to seek its role in vitro. We found that when the MMP8 gene was knockdown, it could inhibit hypoxia-induced PAMSCs cell proliferation (Fig. 10A). We also found that MMP8 knockdown could inhibit hypoxia-induced inflammation, such as IL-6 and TNF-α expression (Fig. 10B–D).

## Discussion

There are increasing evidences show that metabolic dysfunction underlies the PAH pathogenesis [6, 29], particularly altering lipid metabolism in PAH. However, there have been no studies on Fas transcription profiling in PAH. Utilizing HTS, we systematically demonstrated that both mRNAs and lncRNAs were abnormally expressed and altered in PAH after C75 treatment, and these DEGs and lncRNAs may provide novel candidate regulators for future molecular studies. Secondly, we found that C75 treatment could inhibit PASMCs cell cycle. We propose that C75 treatment can reverse PAH pathogenesis through regulating collagen contents, cell proliferation, cell cycle, and anti-inflammatory, thus inhibition of FAS may serve as a potential means for reversing PAH.

Singh et al*.* first demonstrated that C75 treatment can reduce right ventricular pressure, pulmonary vascular remodeling, hypertrophy, and endothelial dysfunction in the lungs [8]. However, the expression patterns of DEGs and lncRNAs after C75 treatment in PAH animal model have not been comprehensively studied. HTS technology allows us to discover previously inaccessible complexities of transcription, such as novel promoters and isoforms. Thus, this study takes the initiative to describe DEGs and lncRNAs to further our understanding of mRNAs and lncRNAs that are associated with PAH pathogenesis. It was reported that dysfunction of collagen digestion enzymes (MMP2 and MMP8), and TIMP1 (a collagenase inhibitor) were associated with pulmonary fibrosis [30]. Loss of the peroxisome proliferator-activated receptor γ (PPARγ) was associated with PASMC proliferation and pulmonary arterial remodeling [31]. In this study, we found that C75 treatment could partially decrease TNF-α, IL-5 mRNA levels (Fig. 6A–E). We also found that C75 had an effect on the PPAR-α and PPAR-γ mRNA levels (Fig. 6F–G). The RT-PCR results indicated that inflammatory is activated in this PAH model, while C75 treatment can partially reverse the inflammatory response.

LncRNAs are a part of endogenous RNAs that act as gene expression regulators and are involved in various developmental and physiological processes and diseases [32, 33]. Analyzing the expression profiles of mRNAs and lncRNA offers new insights into PAH pathogenesis and pathophysiology, and the possible effects of C75 treatment. Pulmonary vascular remodeling including pulmonary vasculature thickening, is a major characteristic of PAH [22]. Previous studies exhibited that pulmonary vascular remodeling coupled with an increase in RVP leads to RVH, and right ventricular heart failure [34]. In this study, we found that C75 can partially attended MCT-induced PAH mice pulmonary vascular remodeling (Fig. 1), and our previous study also showed that C75 has a protective in right ventricular function in hypoxia-induced PAH mice [9], suggesting that inhibition of Fas plays a protective role in PAH.

Cis and trans methods were employed to detect the potential functions of lncRNAs. The cis-acting lncRNA acts on its neighboring genes on the same allele. GO and KEGG analyses of these protein-coding genes showed that these genes mainly belonged to cellular protein catabolic processes, modification-dependent protein catabolic processes, and macromolecule catabolic processes (Fig. 7A). The mRNAs targeted in trans by abnormally expressed lncRNAs were involved in various biological processes, such as transcription, regulation of transcription, response to DNA damage stimulus, protein secretion, organelle fission, mitosis, cell cycle, and DNA repair (Fig. 7B). As for the switch of glycolytic phenotype, we detected GLUT mRNA levels, and C75 treatment could partially reverse increased GLUT mRNA induced by MCT (Fig. 6I). Paula Mera et al*.* reported that C75 is a potent inhibitor of CPT1, the rate-limiting step in fatty-acid oxidation both in vitro and in vivo [35]. We also detected FAS and CPT1 mRNA levels, we found that C75 treatment can partially reverse the FAS, and CPT1 increase (Fig. 6H–J). As C75 can alter glycolytic phenotype and fatty-acid oxidation, we propose that these findings fit into the current paradigm of metabolic theory of PAH. Expression of six lncRNAs and 1623 miRNAs is altered significantly after C75 treatment, suggesting the existence of relationships between the six aberrantly expressed lncRNAs and its 221 potential target miRNAs within the lncRNA-miRNA network.

## Conclusions

Altogether, we performed a comprehensive study of miRNA/lncRNA-mRNA in PAH lung tissues after C75 treatment. We identified some dysregulated mRNAs and lncRNAs which may be potential drivers as well as diagnostic and therapeutic biomarkers of PAH (the dysregulated mRNAs and lncRNAs sample sizes are still needed to expand for validation). GO and KEGG pathway analysis reveals these targets are related to cell cycle, cell division, and vascular smooth muscle contraction that contributes to the pathological process. Differentially expressed lncRNAs such as ENSMUSG00000110393.2, Gm38850, ENSMUSG00000085532.1, ENSMUSG00000100465.1, ENSMUSG00000110399.1, may be associate with the relieve of PAH. We propose that these lncRNA and DEGs may be promising candidates for molecular regulators of PAH pathogenesis.

## Supplementary Information

**Additional file 1. Table S1.** The sequences of the six lncRNAs (in the attachment of supporting information).

**Additional file 2. Table S2.** Lists of six lncRNAs and 1623 miRNAs (in the attachment of supporting information).

**Additional file 3. Table S3.** List of filtered 259 lncRNA-miRNA relationship pairs (in the attachment of supporting information).

**Additional file 4. Figure S1.** Co-expression networks of lncRNAs and protein-coding genes.

## Data Availability

The datasets generated and analysed during the current study are available in the [GEO data, Series GSE128358] repository (https://www.ncbi.nlm.nih.gov/geo/query/acc.cgi?acc=GSE128358). The data used to support the findings in this study are available from the corresponding author upon request.
