# Supplementary material for: Comparative transcription profiling of mRNA and lncRNA in pulmonary arterial hypertension after C75 treatment
Source: BMC Pulm Med. 2023 Jan 31;23:46. doi: 10.1186/s12890-023-02334-6 (PMC9887911; doi:10.1186/s12890-023-02334-6)
Supplement: Supplementary file 1 — Additional file 1. Table S1. The sequences of the six lncRNAs (in the attachment of supporting information). [file 12890_2023_2334_MOESM1_ESM.docx]

**ENSMUSG00000085532.1** GACAGCGTCCTCGTGACAGCATCCTGACTCTTGCCTGTCCAGATGTGTATGAGTCAAGCCCTTGCTCTTCTGCTCCTCACATTGCAGGGGGTGAGTACCATGAGTTTGGTTGTTTGGGTGTGAGCTTGAACTCAACAGATAACAAAAATCTTAGCTTTCAGCCATCCTTTCCTTGCCTCGCCTTCCTATTAACTTGTTCTGAATTCTGTGTGTGTTTTTATTATTGAGTTTTAGTGAGAGGTGAGAAGGCCCCATCCACAGTATTTGGAGAAGGGAAGTGGGACAGGCCATGCAGGGGACAGATGACAGGAGTAGGCACAGAATCTCATTTTAGTTGTGAGGAAAATGTCTTAGGAAGCAGATTCCATGAAGATTCCCCTGTGATGCAGGCAGAAAATCTGGACCCAGTAAAAGTGCTCATCCTGTAGAGCATTGGTGCCCTGCCTTTCCATTTTAAGGAAAGGAAAGAAAGAGCCACAGGAAGGATGAGAGACAATGCTTACCTCATCTGCTTGTCACATACAAACTGAAGAAATGAGTAACTCGGCATAACCGACTCCAAGCTTTGCCCCACCTGATCTGGGCTGAATCAGTGACCATGAGCTTCTAACTGCTGGATCTCTCTGGGATGCCCCTCTTGCTTCCACTCCAATCTACCCTTGGGTGGGAATGCATTCCATGGGGCCGCAGTGGAGAGAGCAAAGCACAGTCTACATAAGGAGGTGTTGAGGCCTGGGGCAATCTTAGCTTGTAAAATTTTTTTCTTTTTTAGTCTATGATAAAATAGATCATATTAGATATTATCAATACCCATCACCTATTTTCTCTCTCCAATTCTCTCCATATCTCCCTAACCACACCCCCTTTCCAGAACTCCTATCTTTGTTTTGTTTTTGTTTGTCTGTTGATGCTCTTATAACCCACAGACCTGGTTAAATATATAATGCATTTTGAGTTGCATGTTGGATACATGGATATAATTATGACTCGGTTATTGTTTCTGTTTGGAAATTAAACATGAGGTAAGGATATATGATTATGTGTTAAGTTGACAAGGGGTGGATTTGTGCTAGCTATTCTTGGAACAGCTGGACTTAACTAAAACCCAAGCAGTTACACCTGTGAGAGAGTTTTCTTGAGGTGTTTTCATTTGAGAGTTTTCATTTGAGGTGGGAAGACTCACCCTAAATCTAGACCATCTGTAGTGTGAAAAGCCTCAAATCGTCCACACCTTCTGGAACAGGCATGGAACAAGGAAGCACTTGCTCTCTGCATGTTGGCAAGTCCATTTGTTCACTGGCATTAGAGCCTCCTTCAGGATTCTGGGATATACTGAGGATCACCTGAGACATCCAACCTTGTGGACCAAATAAAGCTTACATTCTTATACTTCCCATCAGGAGACAGCATTGTTGGAATAGTTAGACCACAGCCTGTAAGCCATTCTAATAAATCACACACACACACACACACACACACACACACACACACACACACACACACACACACACACACTCACACACACACCCCTGACTAATACACCCACAAGATCCTGCTAGTGCTGCCCACATGTACAGAGGTGTGGCGCCACCCACTGGAGCATAGGTAACTTACCGAATTCTCAAACAAGAATGATGCTCATTCCTTCCCTCAGCAACTTTCCACTGCTAATGGCTCAGCTGTAGGGGCAGGGCCTGGAAATCATTTTACTGCTAATGTCAGGATTTTGGCTGCCTTGATCTTGTGCAGGGCTGTGAGTTTATGCGTTTGACAAGCATATCATATCTATAAGACAGCATTTACTGCATTCTTCCCCACCTTCGGAGCTATCATTCTTTCTGCCTTCTCATCTGCTGCACTCCCTAAGCCTTGGTGTGTGGGGGTGGTGCAGATGTACTGGTTAGGGCTGGGTACTCTGTATCTTGGCACTTTAACCAGCTATGTGTCTCTCCATTGACGACTGCCAACAGCATAAAGAACCTTCTCTGACCAAGGCTGAGAGCAACCCAGACCTATGTGTATAAACGTATGTGTAGTTAGAAGGCATTTTGACAGCATGACCATTTAGCAAAAATAACAATAGCAGGTTGTTGACCAAAATTACAGTACCAGGAATGAAATTCTCTTCTGTGGAACAGGCTTAAAATCCAATCAGAAAGTAGTTAGTTATCCCTTAAGAGTCATGCCACTATTGTGAGTGAGGGGAAGAGCCAGGATAGTGGGTGGGTGGATTAGCCTAAACTAAGGGGTGTGGAAAGCCTTGTAGAAACCACTAGTCAGTTGTATAATTTCAAAACACAATAATAAAAAGAGCTCCAAGGGGGTAGCTTGTCCTGTAATAACTTTTCTGTAGCGGGAGATGGATGAAAAGCCTCTGCCGGAGTGTAACACCCCCCCCCCGAAGCACGGGGACTTTGCTTTAGACTGAACTGGGGGACCCCTAACTCACAGAAATAAATGAGCATTGCCAGGGAGCTTGGAGGTGTTCAAATGGTGGCGGTGGTGGGTCTAAAGGGCCAGGACTGGATGACCACATCCAGCTGCTCCCCAAATGCTCACTTCAATCTCTTTCTAAAAATGAGGTGTTAAGACATGGAGAGGAGAAAAGATGTAGTTCTCTCACTTTTTGCTGCTATGTTTGGAAGCCTCAGTCATCTGTGGTGGGTGGTGAAGGAACTGGCTGGGCCTTGTTGTCACATCAATACGGATGGTGCCGTGACTCCGGAGTGTTCTCTCGTGGCTTAGATTTGCATTCTCTCACACAGTCGCCAGGCACTGCTACTGTGTGAACTGTTGGGGGGCGTGGATCGCATGGCTGAGGAGCAGGTGCTGAGCTTTAGTCAGAACTAATTCTTGTTCTGCTTAGTATTATATGTAGTTTGTGTACTTGTTGTATGTAAAACGAAAGCTTCTCAGATATATAGGGCTGAGTATAGTTTTTGAAATCAATGTTACGTGCTTTATTGTAGATCCTTGAAAGTGCATTTTTGAGTTTGTCAGTAGTTCAGTCTGTTTAGTTACTGAGTGGTATTCCATCCTGATAGCCACACATTGTGTCACTCCTTCCTCTGTAAGTGGGAGTTGCAGTGTGGGGTTACTATAGTAAAGCTGCCGAGAACATTCTAGATCAGCCTCTGGAGGTGTACCAGAGTGCAAGACGACCGATCTGTATGCTAGCACACTGATTCTCAATCTGTGGGTCCTGACCACATTGGGCTCACATATCTGATATCCTGCACATCAGATATTTACCTTTTGATTCATAACAGTAGCAAAATTACAGTTATGAAGTAGCAAACAAAAATAATTTTATGGTTGGGGTCAGCACCACACGAGGAACTATATTAAACTATATTAATGTCCCAGCATAAGGAAGGTTGAGAACCACTGGTCTAGCAAGTATAAGCTTGACTTTGGCCGAGGTGGGGGGCCGAGGTGGGAGGGGATTGTCAGGCTATTCCTAAAGTAGTTAGTTGTAGCATGTAGTTCCCCATGTGTGGTCTCAGTATAACTCTTTTGAACTTGCAATCATTTGCTTGTCTTTAACTCTAGCACCTAACAGTCTACAGTAGTAAATCATCATGGTTTTCATCCATGCAGTTGGGTGACTAATAGTATTGAACATTTAAAATCTTTAAAGATTTACTTTTTATTTATGTGTATGTGTGGGGCAAGTGGACCATGCCACCAGACAGAAGAACTGGTAGGTCGAGTGACCCTGAACCCTATGAAACTCAGAAATGAGGTAGGTGTGAGAGCCAGCTCCCTGCCAAACTACCTGCTCTGGTGTCATAACCATGACACCCCACTGAAAGGACCATGGCAATCAGTCACTGTAACATAAAAACCTGAAGTTCTCCATGCTAATGAGGTATCTAGATAGGTTTCGAGTGTTCAGCCAATGAGCTTCCTTCTTGGATATTCCTTCCCATAAAAGGTATTTAATCTGTGGTTTACCCTAAGTAAGGTGTATGAACTTACATTTGCCATCAAATAAAGCATTGGACAAGCAAGGACCATCTCTTCATCAAGAATCACCTGGCCATCATAAAGTTCCCCACCCAAATCTCCCCAAGAAGGCCTTGTCTCTCCCAGTCTCTCTGTACTTCCGTGGAACCAAACCCTCCCTCCTTTCCTGGGCTCTGCCTTCTTCCCCTTCCTGCCTGGCATGTGGATGTCTTGAGGGGAAGGTCTGACCTGGAACCCCTGCTCCCAACTCCAAGTGGTACCCCTGCAGTGTTTAGCATACAGGAGAAGGGGGACCACAGCAAACATTCATGTTTCTGCTGTTTTCCATCCAGAGAAACATGAAGACCTCCATTATGGGCTGCGTTCTGCCTCCCCTGAGCTGCGTGCCAGCAGTCCAGGCATCCACGCAGTGAGGCAGACATGCAGCCCAGCATGTATGTATGTGTTTCTGTTCATTAACGTGTGTGTGTCCGTGTATGTGTGTGTGTGTGTGTGTGTGTGTGTGTGTGTGTGTGTGTGTGCAAGTAGGAGCCCACAGAAACCAGATGAAGATGTTGGACGAATATTTTATATTATGTTATTGATCTCTTTCAGCTGTCTTCTTGCTCTTGCTAAGACTTCAAGTATATATTGAATAAGCTTGGAGAGAGAAGACAGCCTTGTCATTTTCCTGATTTTAGTGAAGCTGCTTTGAGTTTCTTTCCATTTAAGTTGATATTGGTCCTGGGCTTGCTGTAAACTGCCTTTATTATTGTTTAGGTAGGGCCTTTGTATCCCTAATCTCTCTAGAATTTTTCTCATGAAGGGGAATTTGTCAAAGGCCTTTTCTGCATCTAATGAGATGATCATGTGGGTTTTTTTTCTTTCAGTTTGTTTATATGGTGTTTATGTTTATCGATTTTTGTGTATTGAGCCATCTCTGTATCTCTGGGATGAAGCTTACTTGGTTATGGTGGCTGATCTTTTAGATGTGTTCTTGCAGTCAGTTTGCAATTATTTTATTGAGTATTTTTGCATGTTCATAAGGAAAATTGTATGTAATTCTCTTTCTTTGTTGAGTCTTTTATGTGGTTTGAGTATCAGGGTAACTGTACCTCCATAAAATGAATTGGGCAATTTTTCTTCTGTTTCTATTTTGTGGAGTATTGGCATTTTCTCTTCTTCAAAGGTCTAGTAGAATTCTGCACTAAAACCATCTTTATTTTGTTTTTTTGTTTGTTTTTGTTTTGTTTTCCTTTGGTTGGGAGACTTTTAAAGACTGCTTCTATTTATTTCACTAGGAGTTGTAAGTCTGTTTAAATTGCTTACCTGAACTCAATTTAACTTCAATGAGTGGTATATCTATATATCTATATGTCTGTATCTATATGTCTATATCTACATCTATTTCTACACCTACATCTATCTCTCCATCTCTCTATCTCTATCCATCTATATAGATACAGATACAGATACAGATACACATACACATACACATATACATATACATATACAGATACATATAAAATTATCTATTCCTTTTAGATTTTGATGGGGTGCAGGTTTTTAAAGTACGTCCTTATGACTCTCTGGATTTCTGCAGTGTCTGTTGTTATGTCCCCCTTTGCGTTTCTGATTTTGTTAATTTGGATATTCTCTCTCTTTACTGGCAAATATATCCACTGACTGAGAGTCTAAGGGTAGGAGTCAATCGATGATGATGATGATGATAACATTACCTTTGAAGAACTTATCTGTGTAGGTATGTGCACATGCATGTGCTATTGTGCATATATGGAGGTCGTGGAACAACTTATGAGAGCTAGTTCTCTCCTTCTAACATGTAGGTCCCAGGGATCTAACTCGGACCGTTAGGGATGCTACTCCCACTGATCCTATTTCACATGGCTTTCCCATGTTCCCAGCTTTATTCTGCTGCATGAATAGTATTTGATACTGATATTTTATTGAGCCCTATACTCAAGTGCACATGTACTTGTCTCCACTCTCCTTGAAGAAAGGCAAGTGCCTTGTGCAGTGCACCCTTGCCTCTATAATGGCTGTGGTTTCCCAGTGCACAATGATAATTGTAGGAACTGGTTGTGTAAACAAACAAACAAACAAAGATGATAAGCCTAATTGCAGCACTTTGGCAGCTTCCCGAGGTGAAGAACCCAATTTAAGGCAGCCATGTGGACCACAAAGTCAAAGAACTATAAAGCATGCTAGGCCCTGGAGAGATTGCAGGCTGAAGGGAAAAGGAAGGAGAGGGAGACTCAATCCCAGAATGGCTGGGCAAGCATTACAAGAGATCTGAGTTCAAAATAACGTGGGTGCACCTCGAATAAAGAAATAGTAGTGTCACCTACTGTTTATTTAGTGCTTACTGTTTACATATCAAGTACACATTTCCACACATTGTTGCTTTGATAATATATGTGAAAGCTGAAATAGAAATCTGAGTTTACCAAACAGTTCAACTAAAGGGCATCTGTGTCCATTACAAAAGGGTTCTTTGCAGTACAAAAGGATTAGCAGTGGGGTTCCTTTAAAAAGCATCTCCCTTAGTGCCCTAAAGTGTTTATTTATAGATCATTAACTGCTTGACCAACCTGCCTCAGGGTACAACAGCTTAAAGACAACCATCAGGAAGCTGCATCCACAGAGGATCAATGAAACAGCAAAACAAAGTGTCTTCAAAATAGTACATTGTGTCAATGGGAGGGGACTCATGAACCCACATTGGCTTTATCTCAGCGGGGAGCGCATATGTTGGATGGACAGCACACCTGTGTATTCTCATTCGTGCCCAACCAAGGCGGATGTGGTCTCCAATCTATATGGCTAAACTGTGATATGTCCAGGGTCATGGCTATGTTTAGCTTCCCTTTGCCTGTGCTTTAATTAAGAGGTAAAATGATTAAAGCAGCAGATGTTTCTAGCCCGAGGTCCTGGGCCCCAGCACATCCAGCAGATGGTCCCAAGCAAGGCAGCATCCTTTAATGATGCGGAATTTGCAATTCATGAGCACTCATGGTGTCCTTTCCTTTCACCATCCGATGTTCTGCTTGTCAGGGGTCCCATTGTGTGGCCTGGGAGCATGATGAATTAGGCAAAACCCTTCTGGTTTGGGACACAGTCTTGGGTGGATGTAAATTAAAAGCATCATCTCCCAGAAGATCAAGAGATTAGAGAGTGGAGACTTCTGCCCCCTTCCCACCAGCCTGGTAATCTGTCTAAAAGTCATCTCCCATGTCACACATTCTACGCAGCACTGACGGAAGACTGGGAAGTAAGAAGGAACTAGCCTGCTTCTCCATTTGATCCCTTAACGAGTCACCTTGGGTTCATTTTGGTCTTTTGCAGGACAATCAGCAGCTCCCAGCATGCATACTGTCTTTGGGCAAGACATTCTCTCCAGATACCAGAAAACCAATTCTATTTTATTGCCAAGAGTGACCCAGGACAACACATACACATACAACTGCAGGCATGCACACACATAATCTCTGAATCTCAGGGTCTTCCATTTCCTCTAGGTGGTGAATAGTCAGAGGTTCCAAATAATTGAACTGAGAAAGGAAAGCAATCAAAACTTGTTCCTTTCATAGAAAAAAAAAACAAAAACCAAACACACAAAACCAGGGAGAATCAAGTGAGATAAATCAACACTACGATGTAAAGCAATGCTAATGCTAAAGTGGGTTAATATAAGAGTACTTGGTGGGGCCAAGACATTTAACCATGGCATGTGCCCAGGAAAACATATTTGGCTGAAAGTCAGAGAGCAGCAAAAATGATGGACTTGACCCCTTGATGTTCTGGTTTAATATTTTATCCCCTTTCAGAGTCTGACTCTATAATGACAGTAACATTTCCTGTGGAAGATGTTAAGTAAGTACAAGGTCTCTCAGGCCTTTTACTCAGGGCAAATGGGGTGGGATGGGGACGAAAGCAAATAGTGCACAGCTGGCCTGGAGCCATAGCGCTGGAGTAAGAAGCTTTCTATCCGTGCTTCTGCTCCTAGAACCTCGGCCAACCTCCCAGAGGCCTGAGATTCCCTCCCTCTTCTGAGCTCTCAGTATCCCTGCATTTTCTGAGAACCCCGGTTGGCATATTGCAGCAAGCCTGGAGATTTGATTTCTTTTGGCGATGTCCCAGCTAGGAGATGTGTGGCTCTCTGTCATTCTTGCAGACTCCTGTGCCCAGCCTCAGTGGACTGTGAGCAGGAAGCTGCCAGGAGAACTAACTCACTCTGAGGACTGAGCAGGCAGACCACAGAATGTGTAGAGGGCGCCATCACTTTGTTTTCTTGACTTGTGTGGCTCTCAGATGACGTACTGCTGACTTAAGAATTTCCCAGAAAAGGAAATTTGTTCATTAAGGGTATGATAAATTATATGTGCATTTCAGTTTTAAAACTGTTAAGATGCAAAAGTATGAGTATTTTGAAGGAGAAATGGTATTTTAAAAAAAGAATTTACACACGCCTGAGAGTGGATGACACAGTATTCTGTACTTTCATATTAAATCAACACTGGGAAACGTGGATGCGCTCTTGGGCCAGACGCGTCTCTTGTACTTTAATGAGTCTTATTAGCTACCCTTAGTGGATAGGCCAACTTCCAAATACAAGCTTGGGTTTCAGATGTTCCACTGACTGCATTGGAGCCCAGTGTCCTATTTCCTGAGATCAGTGTGTACCAAGTATCTACTCATGCCTGTGCTACATCAAAATTCCCAGCGGACAGGAAAGCCATATGGCCTCACGTGGGGAAAACAGGTGCAAAGCCACTGGTTGGAGATTACCTTTAACCCCATTCCTGCAACTTGTACCAACTTAACGGTCCCTGTCCCCAGTGTCTTCCTACCCTCCAAGTAGTAGTTGACTTAGTTCCAGAGTGTTCTGTTAATAGCTTATGCTAAAGTATATGCAAAATAACACTAGAAATTTCACTTTGCTTTTTAAAATAAAGCAAGATTTTACCCATTTTAAAGGAATTACAGCAAAGATGAAGTATCAAAATATATCCCAGTCTAAAGCATAGATCCTAAAATGAAAGACATATCAAGGTAGGAACCTTAAGATCTGGAACAGTAATTCCTCCTCCAGCACTGTTCTTGTTGTTCAGGATTGTTTGGGCTACCCAGGGTCTCTTGTGGTTCAATATGAATTCTAGGATAGTTATTTTCTATTCATGTGAAGAATGAGATGGAGATTTTGATTGGGGTTACATTGAGTCTGTGAATAGCTTTTGGCAAAATGGCCATGTTTTTCATAATATTAATTCTACTAGTCTTCCCATTTTCTAGTCTCTCTCTCTCTCCCTCTCTCCCTCCCTCCCTCCCTCCCTCCCTCCCTCCCTCCCTCCCTCCCTCCCTCCCTCCCTCCCTCCCAGAGGTTTAAAGTTTTTAATGTAGAGGTCCTCTGCTTCTTTGCTTAGGGTTATTCATAGATATTCTATTTGAAGCTATCATAAATGAGAGTATTTTCATGATCTCTTTCTCTGTATGTTTGTTGCTAATACATAGAAAAGCTATGATTTGTGCAAGCTGATTCTGTATCCTGCTACATTGCTGAATTTATTGTTTCTATAAGCTTTCTGGTAGAATTTTCAGGATCTCTAATGGAAACTATATTATCATCTGGACATAGGAACAGTTTGACTTCTTCTCCTATTTGTAGCCTTTTAACTTCCTCCTGTTGCCTTACTGCTCATCAGTATTTCAAGCATAATATTGAAAAAGTACATGAGGGGGAGATATTTGATGTCTCGGCTTTGTTCCTGACTTCAGGGGATTTGCCACAAGCTTTTCTTCATTTAGGATGATGTTGGCTGTGGGTTTTTCAGGCGTAGCTTTTACATATTGATGTATGTTTCCTCTAGCCCTACATCCGATAGGACTTTTATCATGATTGCATGTTGGATTTTGTTAAAAGTTTTTATTTAGCATCTATGATCACGTGAGGTTTTTTTGTTTGTTTGTTTCGTTTTGTCTTCAAGTACATTTATGTGGCATATTGCATTTACTGAATTGTGTATGTTCTATGTTCAACTATCCCGGCATTTCATGGATAAAGTTAATTTGGTCCTGATGGATAATCTTTTCAATGTCTGTCTAAATTCTATTTGCAAATATTTTTGAACACTTTTTAGACCATGTTCATCAGGGATATTTCTTTCGCTTGTTCTTAAACAAGTTTAAATTCTGTGGGGTTTTGTTTTGTTTTTTGTGTTTTGTTTTTTGCTAGAGCGACACTGGCTTCTTTTCCTCTTTCCTTGCTTCTGACTTTCTCTCCTCCTTCCCTTTTGGATACTTTAAGACAGATTGGCTTTTACAATCAGGTAGAATTCTGTTGTGAATTTATCTAGTCCTGGACAATTTTTTAGTTGAAGGGTTTTTATTACTATTTCAACCTTCTTGGTTGTTACGGGTCTGTTTGCATTGTTGACCTCTTGGTTTAGTTTTTAGTGGTTTAGCCCGATCTAGAAATTCATTTATTTATTTTAGGTTTTCTAGTTTAATTGAGGTTTTAAAAAGTGGTCTGTTCTAGATTTCTTTGGTGTCTCTTATGTTTTCCTGTTCATTTCCTAATTACTTTGGGTTGGGTTCTATCCTCCTTTCTTTTGGCTATCAAGATCTGTCATTCTTGTTTATCATAGAGCCAGCTCTTAGATTCATAGATTCTTAGTATTGCTTCCAACCTCACTCTCCCCCAACCCCCAACCAGTTGTTCTTGTTGTTCAATGTTTTCAGCTCTAATTTTCACTTTTTCTTTTTCTTTCTTTTCTTCTCCCTTCCTTCTTTCTTTCCTTCCTTCCTTCCTTCCTTCCTTCCTTCCTTCCTTCCTTCCTTCCTTCTTCCCTTCTTTCTTTTTCTGTCTTAGTTAGGGTTTTACTGCTGTGAACAGACACCATGACCAAGGCAACTCTTATAAAAACAACATTTAATTGGGGCTGGCTTACAGGTTCAGAGGTTCAGTCCATTATCATCAAGGTGGGAGCATGGCAATATCCAGGAGGCATGGTGCAGGCAGAGCTGAGAGTTCTACATCTTCATCAAAAGGCTGCTAGTGGAAGACTGACTTCCAGGCAACTAGGGTGAGGATCTTAAGCCCACACCCACAGTGACACACCTACTTCAACCAGGTCACACCTATTCCAACAAGGCCACACCTCAAAATGGAACCACTCCCTGGTCCAAGAATATACATACCATCACAACTGGGTTTAGATTTGGTCTGTTCTTGTTTTTCTAATTTTTTGAATTGCATCATTAAGTCATTTATTTGTGTTCTCTGATTTTTTTTTCATGTGACACTTAGAGCTATCTATTTCCCTTGTAGGACTGCTTTCAATGTGTCCCAGAGGTTTTGTTGTGTTGTGTTTTTATTTTATTTAGTTCCATGACATTTTAAAAAATTCTTTCTTGATTTATTCTTTGACCCATTCATCATTCAGTAATGGGTTGTTGAACCTCCATGAGTTTATATAATTACTAGAGATTTGTTGGATGTCAACTTTAAGTTTTGTTACATTGTGGTCAAATAGGATACAAGAATTGATTTCAATCTTTTTGCATTCATAGAGATTTGTCTTGTGTCCTGGGATGTGGCCTGTTTTAACAAAGCATCCATGTGATGCTGAGTAGAGTACATATTCTTTGGTGTTTGAACAAAATATTCTGTAAATATGATTTAAGTCCATATGATGTCAATTATTCCTCTATTTATTTTTTGTTCAGATAATCTGTCTACTGGAGAAAGTAGATTGACTATGTTAGTGTGTCAATTGGGCACCTGAGAGTTTAGTGCATATATGTTTAGGATTGTATTGTTTTCTTGGCTGTTTCCTTGATTAGAATGAAGTGCCCCCCCCTTTTTAATCTCTTCTGATTAGTTTTAGTTTGAAGTCTATTTTGTCAGATATTTGTAATATATCTAGTAATGCCTGCTTGCTTCATGGCCCATTTGATTGGAGTTCATTTACTCATCCTTTTATTTGAAGGCAATGCCTATCTTTAAAGGTGTGTTTCTTGACCCATTCAGCCAGCCATTATTTACCATTGAAATGCATATGTTAATTGGAATCATTGAGTGGTGGATTTTTGGTGTTGTTTGTGTTTTCAGTGGTATTTCATATTTCTTTCCACAGTCTCTCTGCTATATTCATTCTTCTTTTCAGCCAGAAATATTGATTCCTATATTTTCTTTAGGTTTGATTTGCGGAACATAAATTTTTTTAGGTGTTTAATGTCTTAAAAAGTTTTTCTTTCTCCTTCAATCATGGTGGATAGTTGTGCTGGGTATATATTTCTAGGTTGGACATCATAGCCTTTTAGGACTTGGAATGCATTGCTCTCGATTCTTATGGTTTCCAAAGTTTCCTGAGAAATCAGCTGTCCTTCTGATGTTCATATGAAGACCATAAGGATCTTTTTTTCTAGTCCTTTCTACTTAGTGTTCTATGTGCTTCTTATATTTATATGGGTGTGTCTTTTCTTAGTTTGGGGAAGTTTTCTTCTATGATCTTGTTGAAGATCTAGTCTATACCACTGACTGGGGATTCTTCTCCCTTATCTTCACATATACTTTAAATGTTTAATTTTTTCATGATGTCCCATATTTCCTATATGTTTCTTACTGTGTTTTAGTTTTTTTCATATTTCTTGCTTATTTGGTCTAGGTCTTCTGCTTTATCTGAAAGTCCTGATCTTTCATCTTCTGTTTGACTTGTTCAATTTATAAGGGTTTCCTTTGAATTTTCTAGTTACATTATTGTGTTTTCAACTCCTTCATTGTTTCAGCTTGAGTCCTCTTCACTGTTTATATCTCCTTATTAAATTCTGTCTTCCAGTCCTGGATTTTGTCATTTTCATAAACCTTGTGTGTGTGTGTGTGTGTGTGTGTGTGTGTGTGTGTGTTTTTGAGCATCACTCAGACATTTATTCTCCTTTCTTTCTTTGTCCTTTATTTTATTGAACTTCTTCTCTTTGCCTTTCTTAAATTCCTTGACTTCTTTGATAAAGTTTCTAGTAATTCTGTTAAATTCTAGAAGTGTCTGATAGTTTTTTTAAGTTCCATGTCTTGGCGTTGATCTAGGAAATTCTCTTTGGCAAACATTTCTATAGAAGTAGTAGGTTTGGGAGAGCAAGTATTAGCTTGATCTTTCATTGTGTTGTTGGTTTTTGTTTTGTTTTATTTTATTTTGTTTTGTTTTGTTTTTGTTTTTGTTTTTCGAGACAGGGTTTCTCTGTGTAGCTCTGGCTGTCCTGGAACTCACTTTGTAGACCAGGCTGGCCTTGAACTCAGAAATCCACCTGACTCTGCCTCCCAAGTGCTGGGATCAAAGGTGTGCACCACCACCGCCTGGCTCATTGTGGTATTTTTGTAATGAGATTTAAGCATGTGGACACCTTTTCTTAGGTCTATATCTAATATAGATACTTTGTACTATGGTTGTATGTAGGCTAGAAGGGGCGTGTGAGTTGGGGGTAGGTAGGGTGGGCCTTGGCTAAACCTAGGGATTGGTTGTAACACAAGGAAAGTTTAATCTGGGCCACTGGAGGGAGGATCCTCCTCTAGATCTGGTGGTCTGGGGAGAAGGGATGGGTGAGAGGGTCAGGGAAACTTAGCATGCTAGTCTCTAGTGAAGGATGTGGGATATCTAGCTGAGTAAAGAAGTTAAATGCTTCATGAGCAAAACCATGAAGCATTTAACTTGTCAAGGTCTATCAAGTTTTTCCACTTTTGATCTCTTTCTAGTGGTTTTAAGTGGCTTTATTTAGGTATTCACTTATGTCCCCGGTTTATATCTATAATCTGCAAGAAGGTAAATATAGTAGAAATGCAGCCATATCAATCAGGAAAGCTTTTCATCTATCACAGAAAGTGCAATATTGTGAGGTTTTTTGTTTTGTTTGTTTGTTTTTTATTTGAACCACAAAGTATGGCCTAGCTGCTGACCCAGAAGCATATAGACAAGGGCCTAGCATGGCTGTCCACTGAGAGGATCTACCCAGCAACTGACTCCGACAGATGAAGAGGCTCACAGCCAAGCATTGGATGGAACTTGAGGACTCTCATGGAAGAGTTGGGGGAAGGACTGAGGGCCCGAAGCAAATTGGAACTCTACAGGAAGATCAACAGAGCCAACTAACCTGGACCCTTGAGGACTCTCAGAAACTAAACCACCAACCAAAGAACACACACTGGCTGAACCTAGGTACCACCCCCCCACACACACACACACATGTAGCACATGTGGGTCCTGAACAACTCAAATGGGGACTATTCTTAAAGCTGTTGTCTGTCTGTGAAATCCATTACCCTAACTTAGCTCTCTTGTCTGGCCTTGGTGGGAGAAGAAGTGCCTAGTCCTGCAGAGATTTGATGTGCCAGGGTGTGGGAATACCCAGGAAGTGCCTCTACTCTCTTGGAGGAGAAGGAGAGACAGTATGGAGGAAGGAACTGGGGGAGGGATGGGGAAGGTGGACAGCAATAAGGTTGTAAAGTGAATTTTTTAAAAAGTGTGTTTTAGCAAATTCATGCAGGAAACAGTATATGGGATATATTTTCTATCTAGGATTATC

**ENSMUSG00000100465.1**

CTCTTACGTCTCTGCCTCCTCGACCTTGCACTAGAGAAGCCCCACTATGAGGAGACCGAGAGCTGCCCTCCTCTGACCGCGCATCCTTTTCAAGAGAGAGCAGAAAGCGTTGTCCGGCTTTCCCCATTAAACAGACGGAGAGATGTTCTAAGCTGGCCAGGATTCTGAACGCCCTGCCAACGGTCTTTTCCCAGCAGGTTAGCAGCGTATCTGTCACCGCACCTCAGCCTGTGTGCTCTGCAAAGTCAAGGAGGGGAGCTAACAACACGGGGGGGGGGGGGGGGGGGCGGGGGTTGCTCAGTGAGATCTGTCACTTCCTTCAAGACAGAGAGGAATCGGTCAGCTGTTTACTAGGAGTTTGACCCCAGAATTCAGGAACCTGGCTCAGAAAGAGATAAAAGTTGGAAACTGGTTTGCTAGCGTTTTGGCTGCCAAAATCTGACGCTGGGAGTGGCTGCTCTTAGAGCCATTGTATGTGGCCTTGGAACTGGATTGTGTAAGAGACATAGTAGAATATGTATACACATAAGCACACACAGTTCCCACATGTGGAACAGACACACTCGTAACATCTATATACACACATGCATCCACTCATAGCATCCCTGATGGCATATGCATAACCAAGGCCCCTCAGTGTAAGACGCCCTCAGTTCAGAACTTTCCGGTCGGGCATGGTGGCACATGCTTACAATTCCAGCATGCCCAGCAGGTAGGACAATCATGAGCTGGAGGCCTGGACTATAGCAAGACTCTGTCTCAAAAACAGGGAGATGTAGCCTAGAATAAATCAACTACACTCCAGTGGAAGGCCACACATCCAAGAATTTTTGGGCATCGAAAATTGGTCTTGATGGAGGGAAGGGCACAAAGTGAGGAGTGAGGAGGGCAGGGGGTGGGGGTCTGGGGAGAGTGGGGGAGGGGTGGATGTGATTGAAGCACAGTGAGTGGAATCCCCAGAGAACTAATGAAAAAGGAGGAGGGGAAGGAGGAGGAGGGGGAGGAGCAGGGAGAAGAGGGAGAGGTGCGGAGGAGGGAGAGAAAGAAGAAAAACTCAAGTGATGATGGTGGTGCTGATGGGGGTTGGGTGGTGATGGTGATGGTGGGGTGGGGTAGAGGTGGTGGTGGTGGTGATAATGATTGTTGTGATGGGGGTGGGGATGGTGGGGGTTGGGTGGGGTGGGATGGTGGTGGTGAGGGTGGGGTTGAAAGGAGGGGCTACAGAACTACTCCATACCCAGAACCCTCCGCTCCCACAGCCTGACCTGTGCGGGAGCTCTACCACCTGCCTGTTCTGCATGCAGATGAGAGCACCATTGCCTGCCCGTTTCTGACTGTGCTGTCTCTCCCCAGCCTTCCCCACCCCTGCTCCCCACGGGGGGGTGGGGGGTGGGGTAAACACCCTTATCTCCCTGCAGAGCCAGGGCCGTCGCCGCGGTTGTGGCCTCACTTCCGTGTACCATGCGGGAGGAAGCCCTGTTGTTGGGTCTGTAGGACAGTGAGCTGTGTTGACACTTCCTCTTTCTCAATGCCTGTGCCACTCGCTCAGCTTCTCCTACCACAGTCCCCTCCCTCAAGTTCCTGGGGCCTAGGATCCTAGTCCCTTCCCCCTCCACCACCGATAATACCCCATCTCCAGATGTCCCGTCCTTCCTGCATCTTGGGAATCCTCTCGGGCTCGGCTCAGAGGCACACGTAGCCTCTCCTGTTGCCAGACATCTGCCGGAGAAACAGAGTGAGAGGAAGTTCTGGGGGGCTGACATAGCCACAGCTGGAGGGAAACCGGCAGCAGAATGACGAGGAACTCATTTTGTCTGATCACCATACTTAAAGACGTTTCAAAAAAAAAAAAGAAAAGAAAAAAATTTCCTGCTATCTTGGTCCCGGCTCCTTACAAATCTAACACATGCCCTGTTGTCTGTGGGTCCTACAGTCGTAACACAAGCCTAGCACATGGGCTGGTATCAATTCAATTTTGTCTCCAAAATTTGACAGGGAACTTGAGAGTACACATTACTTGTGATCAAGGTTGTTGCTGATACAATTCATCATACCGGGGCTCAGAAGATGACTAATGCAACCACGGAGACAGCTGGACGGATAAGAGCTGCTCTTGTGGAGTGCCTGGGTTTGGTTCCCAGCATCTGCTTGATAACTGCAGCTCTGTGGGATCTAGTGCCTTGTCCACCTCTGTGGGTACCAGGCACATCCATGATGATACACAGGCATGATGAAACATAGACATATATACAGGCAAAACATCCATATACCTAAAAAGCAAATAAATTAAACTACAAGAAAGAGGGCCTTCATCCAATACGAGCAACATTCCTATAAGACCCTGCAATAGACACACTGGGGTGGTGGGGGGTGGGCAGGGCACAGCATGTAATGATGAAGTCAAAAAACGGGAGCTTTCTGTCTACAAGTTCAGGAATGATGACGGTCACTAGCAACACCAAAAGCTGAGAGAAAGGCATGAAGGCTTCCTCTCAGAGCTCTTAGAGAGAGAGCACAGCCCTGCCCTGCTGACACCTTGACACTAGACTTCCATACTCTATGTGAATCTATCCTCTGTCGTATGCCGCTATAAGAACAATAAAGAGCTCAGATAAATCGCCCGGCGCAGAACGCCTCCTTCTGATGCCCCCTCCCAGTGGAAGACACGTGCGTTTCAATCCATCACACCGAAATGAAAAGTTTCTGGTTGAAGCCTGCCGTGCTCTCACCTGCTTACTGTCACCTATCAACAGAGATTCCAGGAACAGAGACAGGATACCGCTGAAGAAAGACAAGTGGCCACAGCAAGCAGGGTACATGGGTTGACAAATCCTGCACGCAAAAAAAATGACATAATCGCTCATCTAGAAAACTTGAGCAAACACTTGCTGGAAGGAAGACACGGCAGGATTCCCAGACCAGTCAGTCAACCTGTGCACCCTTCACTCTGTCATTAAGCAATAAAAGGCCGGACCCAGAACCGTATGCTGAGCTCACAGGCATCCCTTGGACCCAATATCTGTCAACATGTCGTCTCTGGCTTTGACATTGATTTATTGAAGGTTTTAAGGAGGTGGGTATTATTCAAGGATTCAGAAATTTCAAACGCTATCAGTATTCTGAAAAAGAAATTGCTTTCAGTGTTGTAAATAGAGTTGTCCAGTAAAATATAGGAGACTCAATTAAATTTTAATTTTGGACAATGAATAATATTCTGGTATAAAAGCACGTGCTTTTGTGTGTTTGAGGGGTTTTCACATCTTTTGTTAATCCTGTGAAAGTTTCCTATGTGCTTGTAATGTATTTTGATCATAGTCATTGTCTTGCCCCACTCCGAACCTGAACCTTTCTTCTTCTGAAAGTTCAGCCTCTACTTTCATATAATGTGTGTGTGTGTGTGTGTGTGTGTGTGTGTACTGTAAGTGTATCTCAAAATACGCATTCATACCTCACGCATGTAGTGGTGTAGAGATACACACACATACACACACACATGTTTATCTTGTATATGTGCCTGAACATACCTATCCACACCATATGCATGCAGTGCCTACAGATGCCAGAAGGGGACATCAGATCTCCTGGAACTGGAGTTACAGGTAGTTGTGAGCCATTATGTAGGTGCTGGGAACTGAACCCAGGTCCTTGGCAAGAGCAGTAAGTGTTCTTTAACCAGTGGGCCATCTATTTTGTACCTGGGATGGTTTTAAATAGCCAGATTATATTTGCCAAAATTCTCTGAACTGTGCATTTAAAATGCGCAAACAACATCTCTAAAAATAAATGCCCTGTTTGCCTAAGCTTGGGGAGTCGGGTTTCTGTGAAAGTGGCTGACCGTAAGCCCTGGCTAATTAGAAGATGAGAGCGGTGAACTGAAAGCAAGTGGAACCTGGGATTCTCAGCCCACTGGGTTCTCAAGCCTTCAGAGGGAGTGGTACTGGTTGCGCTAAGCTCGTTCTTCGGGCTTCTGGTTCGTTTGGGTGATAAAAACTACCCAAAAGACTAAGATTTGGGACAAATGCGGTTGTGTGAAAATGAGACCATATATGCTTATTCCAAGGGGAGACTGAAGGGCCAGGAGAGTCAAACTTCCGAACTTCCGGTATGTGCAGGCAACTTGGGAAAGCTTTAGAAACTCGAAGCCATGAAATCAGGTGTTTGAACCAGACATACTTAAGTTTTTGACACACAGAAGGGCCCAAAATGGCACCTCAGTTAGATGGGGGGGGGGGGGGGGGGAGACGTTACACCTGGTGCTCTTGTTCCCACCAGCAGCCGGCAGGCGTCTCTGTCCCTGCCCTTTCTGGACTCCTCCTGAGTGGTTTTAGGTCAAAGCAGCTTTTGCAGCCACACAGGACCCTCCTGTGGCAGAGCCTTCCCTTTGCTCACCATTCTACCCAAATCCACTGTCCACAGGGGGGCAACCGCTGTCCACAGGGGGCAAAGAGTCTGCTCTGCAGGTCCCTAGCCTTGCTGGTAAGAGAAGTTTAAAAATAACCTAGGAAGGGAACAGGAGGGAGTCAGTGAGCTAAAGAGCAACATGCCAAGCAACAGGGAGATGCCAGCAGCTGCCGAGAGGATATGAAAAGAGGGAGAAACAGAAACTCAAGGTCCTCCCAGAGACAACTAAAAGACACTCTGTGGAGGACAAGGTGGGAAAAATCAGGAAAGAGAAAGAGAAAGAGCCCGGTGCTCAGAGCCCTTGCTTCTTGCTTCTTCCGTGTGTGAAGGAACACAATGTTGCTTTCTTCTCCGGGTAATGTGGATCTTGAGCACAAACCCAGCAACATCTTCCTTTCTGATCAATAGAGGAGGAAGTATTTAACCTTAATATTATTTAGGATAGTCTGGCATCCAGCTAAAACCTTGCTGGGGTAGCCTGAGACTCAGGACAGGTCTAAGAGAGGAAGGCATCTTCCTGCTGTCCATCTTTTCTGCTGTCTACTTTGCTGGTGAGCCGTAGCTCTGGGCCATGAGAGAATCACTAAAAGCAGTGATGGGCTGTTGGCTTCCCTTCAAGTTTTGTCAGCTTTGAGTCACTCTAAGGCCAATGTCCTTGGTTCCTCTTCAGTGCTGCAGTTCGTTTCAAGCTTTTGTGGCTCTTCACCGCCTACAGAAGACCGGTGCCAGCCTTTGTTAATATAGCAATGGGAGACTAGTGATTAAAGATGAGTTAAAAGCAACATGCAAGTTTTTCGTTGTTTTATAAATTGGCCTTTCTCTCTCTCTCTCTCTTCCTTTATAGTTAGTCAATCATGCCACTTGCCTCAGAGGAAGCCACCATATTGGAATTCATATGAGGGGTCACCCCCAAGCTCCTTATAGTACTCCCTACTCAGCCAGTCCTAGTGGAGACTCTGAGAACAGAAACCTGAAGCTGTCCTGTCAAAACTGCATGCCTTTTGGGGGCTGTGCGCATCAAGCTACCAAGTACAAATCCCAAGATGTCTGCCAGATTCCTGGTGGAATCCAAATGGGGTGGGGAGGAGGTGTCTTAGTTAGGGTTTCCATTGCTGTAAAGTGACACCATGACCAACGCAACTCTTATAAAAGACAGCATTTAATTGGAACTGGCTTATAGGTTCAGAGGTTCAGTCCATTATCTTCAAGGTGGGAAGCATGGCAGCATGCAGACAGACGTGGTGCTGGAGAAGGAGCTGAAAGTTCAACACCTTATTCCAAAGGGAACCAGGAGAAGACTGTCTCTCATGTGGCCAGGAGGAGGGTCTCAAAGCCCACCCTCACAGTGACTTGCTTCCTCCAACAGGGCCACACCTCCTAATAGCAGCACTCCTGGGCCAAGAACATTCAAACCACCACAGGGGTGTAGGGATGCTATTCCTAATCCCTACTCCTGGTTGCTGGGAAGACACCAGGCAAACGCTGCTGCTGTTGGCAGTAATGACCGTGCTTGTGATACTAAAGGCACTATGGGAAGACAACAGTCTCGGAACCACACAGAGGAATACATGAGCAATATGAAAGTAACTTTCTTTTTGTAATAAAGTGGTTACCCAAATTGGTACTACCAAGCTTACACAGCCAAGATGGCCTCCGTCTTTTATCCCTAACGCATGTGGGGACTGTATCTGATCCTACTGGTGGGAGATCAACTTCACAACCACCCAGAAGAGGAAGGAGAAGGCTCAGGGAGCATTCAGTTCAGTTTGAATGTTCCCTCCAGTTGGTGGCACTGTTTGGAGACATTTAGAAGGTGCTGGAGGAAGTATGTCACTGGGGGGTAGATTTTAAGAACTTGAACCCTCACCTACTTCTTCATGCTTGTGGCTTAAGATGTGAGTTCTGCCAGGTCCACTTCCCACCTGGCGCCATGGACTCTCACCCTCTGACACTGTAAGCCAAAATAAACCCTCTCTTCTCTAAGTTGCCTTGGTCATGATGTTCTATCACAGCAACAGAGGAAATAGCCAATACAGAGGGTGATACATTTATTAGAAGAGTTATTATTGCTGCCACAGGCAGATGACAACATGGCAGGAGCTCTGAAGGGAGGAAGTGAGGGATCACGGGGCACAAGTGGGAATTTCTTCTTTTTCTGAGGATGGGTGTGGCTTCTCAGCATAAGCCCGCAGCCAGGCTCTCTGATTCCCCAGACAGGAAGCAGGCAGGCTGGTGTGAAGCAGGGTTTGTGGCCATTAGTACCCATTTGGATGTCAACTCCTGGCACCTGGGAGTGTGTTAGCTAGAGATTCACAGTCAATGTTCCTATGAGAAGAGAGGGTTGAGGAAGATTCTGTGGCCTGTGCGGCAGCTGGATGCTAACATGACATCCATTTTTTAAAGATTTATTTATTTATTTATTTGTTTGTTTGTTTGTTTGTTTAATGTATATGAGTACACAAACACACCAGAAGAGGGCATCAGATTCCATTCAGGTGATCGTGAGCCACCATGTGGTTGCTGGGAACTGAACTCAGGACCTCTGGAAGAGCGGTCAGTGCTCTTAACTGCTGAGCCATCTCTCCAGCCCACATCCGTATACTCTTAAGGTGGGCTCCCAAGTTCAGAGCAGAGGAACCCTCATCTTCCAGCAGAAGTGAACAGTGGAGGCTCCAGCCTTTTTTCCCACTCCCCAATCTAAGGAAGGTAACTTGACTTACCCAATCTGGAACTGTGAGACACACACACACACACACACACACACACACACACACACACACACACAAATGCAGATGTGCAGCTCTGCCCCCTCCCAGCCCCATGGTCAAGCTGACTTGTTCCTAGCTGTCCCTTTCACCAACCTTTTCCTGCAGGTGGCAAGATGCAAAATGGCATACAAAGCTTCAAACCTTCAAATGTCAGTACCTGAGTAAGCACATCATTTCAGAGCGATAGGTGAGATTGTGCTGAAGTCTAATCCCCCACAAACCACTCAGGGGGCCATTGAGGACTCCTGGCATTCCTGCGTGAACTGCCTGGCTGTTAGGCCCTTTCTTCTGAGGAAGCTGTTTTCTCCAGGAATGGTATTACCATCTGCCATCATGACTGTAGTGGAGACCTCCAGAGTACATTGGCCCTGCCGAATACTCTGCCCCTGCTGTAAGTCTACTAGGCAAAAACAGACAAACATGCTTGTGTCTCTCCCCGATGTCAGAACATATTGAATAACAATAAGTTCACAAGCTGTATCAGTTTGCCAAGCAGTTCCCATTTCCCTTGATAGCTGGCTGTTGGCAAACATGAGTTCTTTTATTCTGATCTTCAGTGTTAACGCTCAGATCACACATTACACATCACACAGGAGGTATTTTTATATATGTTCACATGTATCATATGTATAGATGTAGATGCAGAACCTGTAAATGTTGAGGAGGCCACAACAGCAGTTGCCAAGCTCAAAGAGGTCTCACTAGAGGCAGAGGCACAGCTGGATGCTAACATTGAATTTTCATACACTTAACCTGGGCAAATACAGGTATTTAGATATTAGCAAATTCAGGCCTATCCTGGCATTATACTTCCTTAATCCTAGCACTCAGGAGGAAGAGTGGGATGGATCTCTGAATTCTAAGCCAACCTGGTCTACCAATAGGTTCCAGGACAACCAGGGCTACATAGTGAGACCTAGAGGCAGGCTGGAAGCTTTTGGGGATTGGGCCAGGTTTCCAGCTTGAGTGAAGCTAGAGGTGAGGAGAAAGACTGGGTTTGGATGGGTGGATGGATGGATGGATGGATGGACAAGTGGGTTGACGGGTAAATACATGGATGGGTGGATGGGTAGGTGGGTAAGTGGATGGAAAGGTGGGTGGATGGATGGATGGATGAGTGGGCAGATGACAGACCAGACGGATGGGTGGAAAGGTGGGTGGGTGGAAAGGTGGGTGGGTAGATGGATGGATGGATAGATAGATGGATGGATGGACAAGTGGGTTGATGGGTAAATGAATGGATGTGTGGATGGGTGGGTGGGTGAGTAGGTGGGTGGAAAGGTAGATGGGTAGATGGGTGGATGGGTGGGTAGATGGGTGGATGGATGGATGGACAGGTGGTGGGCAGATGGGTGGGAGGGTAGGTGGATGGGTGGATGGGTGAAATGGATGCATGGATGGATGGGTGGATGGATGGATGGATGGATGGATGGATGCAATTCAACTAATGCAATGGTCACTGAGCTCAGGTAAAGTCCTCAGAACAGCTGAGTGTTCTCTACCCATGTGTCCCTTGATGGCACACTGTATGGGCAGATAAAATACCAGTAGGGTCTTATAGTGTTAAATACCCTTCTCTCTATGGCATTCAGGCCAGGGTGCTGCACCCTATGACCGACCATCTGGTGGCTTCAAGTTCTCCAACCTAGACTTTATGACATGGATTTAATATGTCCACATGGTTATATGAGCCAAATATAGTTGGGTACGTTCATAGGTAGTCCACAGTTACCATTCTTAACCTGTGCCCCCAAAAAGCCAGGTTTAATTAATAATTCAGCAAAAAGAGCCAAATGAAGAGTAAAAAGCAGAAAAGGAAGATGCAGTCAATGTGGCCACACTGAGAAGAGGAACACAGAGAGATCTAGCAACCCCAAGACCACCTTTAGGATGTTTAAGTAGAGGGCTGGGGTTATGTGTATCCAAGCAGTCCTGGCCAAGGTGTGGTCCATCCCTGATGGACACTCTCTCTCCTGTAAGGTGGTGGTTTGACAATCTGTAAGTCCCTTTCATGAGAAAAGTTTCCCCAGGCTGGCCCAGGCTTTCAGCTTTCTGCTCTTCCCGCATGAAGTTCCTGGGGACATTGTAACTTCTTGGGCATCTATTGTTTCAATGGTCTGGGAGGCAAAATGAGGGACAGCAAGTATCTCTTTTTACTATGGTAACCCCTGCCAGACTGGACACGGGACATTTGACTTACAGGAATAAGTAATCAATCTGTGCTTCGTGTATGGTACGGAACAGCTAATGTCTACATGGTTACAAACCAGTCACCCTACCTCTATATTTATGCTTAAAGCCCAATACTGCTTGTTAAATTAGTCACAGCAGCCAGGAAGCAAAGGAGGGAGGACATGGTCCCAATGTCTCCTCCAAAGGCCTGTCCCTACGAACCTAACTTCCTCTGCTAAGCCCTGCCTCCTGGAGGATCCATCACCTCCCATTGGAACCACAGGCTGGAAGCGAGTCCTAGGCAGACCCATACTGTATCAATATCAGGTCTAAGTGAGCAAGATAGTATTCCTGTTAGACAGTATTTTCCAGAGAGCAGAGAATGCACTCATTTTAATGTTTGATTATCATGAAAAGGAAGACACGATTTTAAAATGCTTGCATTGACCTTCTCCACGGCAGTCGTGTCTGCTTTCCTGGCCCCAGCAGAATCTAATTTAGAAGCTCTTCACCCTTTCTCGTCTTCCTTCCGTGTTTTCTGGCACGGTCCCAGATCAGACTTCATCTGGTGCAACAAAGCATGATACGAATCCCAACGGGGTTGGCTTGGGGCCTGTTGTGAGATCTGTAGAAACGATGCTTACATCGTGGTGTTTTTCTTGTCCCGTATCACCTGGACAGGTATTCCCTTCAGGCTGTCTGTAACTGTCACGGAAGGCAATTTCACGAGAGGCGTCCTTCCACTTGGGATTTCTGGGCGGAGGGGTTGCCGTTAGCATCTGATTTGCACCGACTCGGTTGGCCTCTGCTGCCAAAGAGAGAGGCAAGAGACAGGCTTTCTGTAACACTGGCAGTCTGACTTCATCTCCTCCAACAGATCGGCAGCAGAACAGGCTCTCGCCTGTCAGACCTACCTGAAGTGCCCTCTTTCATGCAGTCCTATCTGTCAGCTGGAGGACCAGTTCTGGGAGGTGGACGTTGTTCTTCGGGCTCCAAAGGCCATATGCTAAAAATACTGATGGCAGAGACGGACACCCGAAGAGCACTTGGCAGTGAGGATACTGTGCTGTGTGGAGCCTGTCCTTCAGGGGAAGATCTCCCAGCTGCCCAGTGTAAACAAACACAGTGCCCCACCCTACGTGAGCTCTGAGAACGGGAGGGGGGCTTCTCACTCTGGGTGACAGGGGACCCCTCTCTTTCTGGCCTCTCACCCTCTGCTTCCTGGTCTAAGTGTCCTCTTTGGGTTCATGAGATCTTGATACCCCATAGTTGTGGGTTGAGTATCCCTCTAAATCAATCCGTGGGAACATGTGGAGGTTTCAGTGGATGGCTTAATGTCTTTCTTGTGTGTTTTGGGCAGTTCTCAGGAAAGCGAGTGAGTTCTCACTCCCGTGGTGCCGAACTAGTTCCTGGACCATCAAGTTCTGAGAAGGAGAAAGCAACGCACAGCCTTCACCAACCAGACATGGCTGCCCAGTCTTGGACTTCTCAGGTTCCAGAGCAATCAACCAAAATAAACAAGTGATTTGGGCCTCAGGTAGTCCCTTTAGTAATGGAAAACTGAAAGGTCCCGAGTACAAGACGCAAGGGGGATAAATGGGCAGATTATCCCTTCAGGTGAGAAGTTTACTGTGGGTTGAATAAGATGGCTCAGAAGCTAAGGGTGAGTGCTGTTCTTCCAGAGGACCAGAGTTCAATTCCCAGCACCTGCATTAGGCAGCTTACTCAAGTACAGCTTTGTGTATAATTCCAGCTCTATGAGAGGTAACTCTTTTGACCTCTGAGAGCATACACAGAGAGGGGAGGGGAGGGAGGGAGGGGGAGGGGGGAATGGGAGGGGGAGGGAGAAGGAGAATAATGTCTTAAAAGCAGTTTATTGGACTAGAGAGATGGCTCAGTGGTTGAGAGCACTGCCTGCTCTTCTGAAGGTCCTGAGTTCAATTCCCAGCAATCACATGGTGGCTCACAACCACCTGAATGGGATCCAATTCCCTCTTCTGGTGTGTCTGAAGACAGCTACAGTGTACTCATACACATAAAATTAATAATCTCTTTTTAAAATGTAGTCAGATACACAGAAGGTAATAGATTGACTTTTTAGCCCAAGGTCATCTTAGCTTCAGACTTGTAACATCCTAACTCTCCATACCTGGCAACAAAGTTCAGCTAAAGCTACTTTTCTAGGACTTGAACTCTAACAGGCACCTTTCACTGTCCCTTCAGATTGCCTCGGCCCTTGACTTTATCCTGAAGTATCTCCTTCCATTCTCTCTAAACAGACTTTCCATGCCCTCTGTCTTCAGTATTTCAGGAAACCCTTAAGCCATGACTCTGCCTCTAACCTCTCTCGGCAACCCAGACATGCACAGAATTCGCACTAGCCTTGGCTTTGCATCTCTTAGAAAACATTACCTAGTTGTTCACAAAACAGTGGGCAACACCTCCGTGTCTCTATTCAGTTGTCCATTGCTGTGTGACAAGTTACTTCCAAAAGTTAGTGACTTATTATAGCAATAACATATCATCTTTCCAATTAGAGAGATTGGCTCAGCAGTTAAGCACACCCATCAGTCTTGAAGAGGACCAGGGTTCGGTTCCCAGCACACACACACACACACACACACACACACACACACACACACACACACGGTGGCTTACGACCCTTTTCTGCCTCTATGAGCGTCAAATGTGGTGCACATACGTAAATGCAGGCTATCACTCATGCATTCACAAGTAAATAACTAAATCTAAAGATTAAAACAAAAGACAGGGGCCTGGAGCGAGCAAGGGGTGCGGGGAATAGAAGACAAAAAACAAAAAAAAAAATTATCTCATTTTTCCTGTGGCTCAGAACTCCAGGGGTATCTTAGCTGAGCTTTCTGGCAAAGGATCTCTCTTGGGTCAAGATATTGGCTGTGGGCATAGCTATCCTCACCCTTGGTTTGTTCTAGAAGAATCTCTGCTATGACGGCTTGCTCACATGCAGAACAAGTTGCAGCTGGCTGTCGGCAGGAGACTTTATTTCCTCCTCTGGGTGAGCTTCTTCCTAGACCTTCTGCCTCCTCATGACTGGGCAGCTAGCTTCCCACAAGCAGACAGGAAGGAAGTGGCTACGCCTTCTTCAACTTGGTCCTAGAAGCGCTAGCCCATCCTTCCAGCCATAGTCAGTTGCCGAGTCAGTCACAAGTGATTCAGAGATGGGCAAGACACTTGCAGCTGGTTTGAAGGATGCGAACAGGTTCACAAATGAATGCTGGGCAGCAGCTGGCATTTGGAGGGGGCCAGGGTAGCACTGCAGACTCCAGGAACTATGCAAAAGAGGTGTCTTCGGGTCAGGAAATTAACTCTGCTGCAACAGGCTTTGAGAGCAACAGCAACCCTGCAAAGAGTACCAGAGAAATGGAGTATGGTGTGTCCATTTCACCTGGACCATCTTCTGAAGCCCCAAATTAAACAACCTCACCAACATCACCCTCTTTCCATGTAGAGAGATTAAAAGAAGACAATAAAGGAAATAGAAAAGGTG

**ENSMUSG00000102196.1**

CACATTAGACCCTGGAACTCCTGGTCCAGTGTCTTAGTCAGGGTTCTCTAGAGTCACAGAACTTATGGATAGTGTCTATATATTAAGGGGATTTGTTATGATGACTTAGAGTCTGTAGTCCAACTCCCCAACAATGGTCAGCTGTGAATGGGGAGTCCAAGGATCTAGTAGTTGCTCAGTCCCACAAAGCTAGCAGATCCAACAGATGTGTTGGCAAGTCAATGCAAGTAGGCGAAGAAGAGTGAATCTTCCCTCTTTCAGTGTCCTTATGTAGGTCTCCAGCAGAAGGTGTGGCCCAGATTAAAGGTGAGTGTCACCAGACCTGGATCTGGGACTTCTTTTGTTTCAGGATGACCTTGAACTCAGAGATCTCCTTGCCTTAGTCTCCTGGGATTCATAGCCACTATGCCTCAAGATCTCCATGCTAAGATCCAGGTCAGAAACTTGTATCTCTCAGCCTTCCAATTCTGGATTGTAGCTCATTCCAGATATACTCTAGTTGACAACCAGGAGTAACCATTACACCCAGCTAGCCTAGTCTGCTTGGTGAAGTTCCAGGCCAAATGAGAGAAGAGACCTCGTGTCAAACAAAAAGTACCAAATGCCCGAGAAATATCACCTAAGGTTGTTCTCTGATGTCCACATAAGCACACACATGTGCACTCCATCCACTTTCCCACCCCCATCCCCACCCTCACCCCCTGCACAACACAATTCTAGTTTCTCAGTGCATTGTCAGTCTCTTGATCCATTCATGCTTGTTCTGACCTCTTCCCTGGTGCTTGGTTAAGTCCTTGAACTTCCTCTGACACCTCAGCTGGACATGAAGTGGCCAGATGAATGGCCAGGTGAGCAACTAGGACTCTTGGTCGAGGAGGAAGTGACCATTTCTGGTACTTGGAGTTCTTCCTGTTCTCACCCCACACTCCTTGCCTGTCTAGTGTCAGGGAATTTGAGGGTCCTTTGTACTATCACTTAAGTGCCTGACTTCTTGTCCCTGACCTCTTTTTCAGGACCTTCACTGGCTCCCAGACAGCTGTTTTCATGTGGTATGAGGCCCCTATGTTTCTTCCAAATTTGTCTGTTCTGAAAGCATATCAGGCAGCCAGAGCCATTTCTCAAAGAACAAGGTCCTTGCTTTTTACGTCGATTATGCATTGTTTACATTTAAGACACTACCAGAAAAACTATGTTTCAACTAACAATCAAGACACCCCTGCCTCCCCCCTTCCCCCACCAAGTCAGGGTCACAGGCTGGTCTAATCTGGGCAAGCCTTCAGTTGAGACTCCCTTCTCAGATGACCCTAGGCTGTTTGTCATTGACAGTTCAAGCTAATCAGGACATTTAGACACTGTGAGGACCTGTGTTCTCCTCTGAGCCACAGGTCACCCCGTTCCCCTCAGACAAGGCAGGAGCAGAGCAAAGATGATGCCTTCTTTGTTTCTGTTCTTTTTTTTTTTTTTTAATCTTCCTTTCTTGCCAGCAACTCCCACTTCCTGCCACTATTAACACAACCCTCTCTGCCCTCATCCAATCCCTGGAATGGAGGTCAGGAGGACCCAGGACTTTCCATCTGAACTCTCACGGCTACTTACAGAATCACAGAGGTTAGCGGGAAGATTGAAGATAAATGAGTTAATTCTTCATGTGAAAACCTTTAAATGCCTCTGATAAAGGAGTTATGGTTTAGTGTTGATTTAGATCCAAGGAACTTTTAGCCTCCCACGGATTTAAGTTATCTATTACCTACCTATCACGTATGTATGTATGTATGTATGTATGTATGTATGTATGTATGTATGTATGTATGTATACAATGCATGTATCTGTCTATCATCTATCTATCTATCTATCTATCTATCTATCTATCTATCTATCTATCTATCTATCTGCCTATCTATCTATCTGCCTATCTATCTATCTGCCTGTCTATTTATCTATCTATCCATCTATCTGTCTGTCTGTCTGTCTGTCTGTCTGTCTGTCTGTCTGCCTGCCTGCCTGCCTGCCTGCCTGCCTGCCTGCCTGCCTGCCTGCCTGCCTGCCTATCTATCTATCTATCTATCTATCTATCTATCTATCTATCTATCTATCCATCTATCCATCTATTTATCCATCATCTGCCTATTATCTATCTATCTATCTGCCTGTCTATCATCTTATCTATCTATCTATCTATCTATCTATCTATCTATCTATCTATCTATCTATCACCTATCCATCTAGCTGTCTTCTTTCATCCACAGAGGTTTCTTGACTCAGGTTAGCAATATGTTTTAGTAACCCCAGTGTAGGCTGAAAAGCTGCATTTGAACCCCCAACTGTGGCCTGGAGAGCAAAGTCAGTGGTTAGGAACACTTGCAGCTGTTGCAACAGAACTTGAGTTCAGTTCCCAGCATCCACACCAAGTGGCTCAAAACCACCTGCAACTCTGGCACCCTCTTCTCGCCTCCATGGCATCTGCACTCACATGGCACACAATCTCTCTCATATACACTTAGCACTCACATGGCACACAATCTCTCTCATATACACTTAACACTCACATGGCACACAATCTCTCTCATATACACTTATAAAAATAACAAATAAACATCCAACCTACACAGATATTGATTATCAATTTGGTGCTGTAATGCATCAGTTGTTCCCCTTCATGTCTATGTGGCTGGCTGAGGTTATTTGTGGCTTGCTGCTGCTGCCCAGTGTCACAGAGAGTATCACAGCACATACTGTGAGTCCAAGGAAAGATCAAAATGCAAAGTTCAAAGTATGCTTTCTCCTGAACCTCTATTAACTTATCCTTTCATAAAGTTTAGAAAAACAGTAAGTTGGAAATTATCATCCATCTATATGTCTATTTATCATTACCAATATGTCTCTATCACCCTTCTATCTATATGTCTATTTATCATCACCTATATATCCCTATCATCCATCTATCTATGAATCTATTTATCAGCACCCATATATCTTTATCATCCATTCATCCATCCAACCATTTATCTACAGAATGGACCGATTTATATTCCATTTTATCTATCATCTACCTTTTCCGGCCTGTCTTGTTTTTACATATGTATGTGTGTGCATCTATATGTTATACATGTGTTCAAAGTCATCCATGTACATAATTTCAAAGCTCTTCAGAGAAGTCTCCTGGATAAATCACAAGACAGTTTGTGCCCTGCTCTTTGTCAGGTTCTAAATTTTGAGCTTCCATGTGGCTTGCTGTTAGTCAAGATGAGAATATTTCCTCTCTACTCCTTCATAGAGACTGTCTCCTCTTGCAATTTTGTTTGGTTTACTTTGCATTTCTGTCATTAGCTACACGTGGCTATTGCTTGAAGCGAATCTCTATAAAGCATTATTACTACTTCTCCACATTTCACATTTCTGTGCCTTCCTGAAACCTGTGCTGTAATAGTGTAAGATTTCTCTCTCTACTGTGCAGAGTCTTCCTCAATTTCTTCCACATTCCAAAGCTTCATTGATATCTTTCCTTTGTTGTCTCCTTTTTCCACTTTGTTCTTTTAAATATTGTAACTTTCTTTTCAAAACTTAAGTATAGTCAGGTAGGAAGGATTTGAATGAATACTTTATCCCCGATTTTGCACCAGAGCTTCATGGTTTTTGTTTTGTTTTGTTTGTTTGTTTGTTTGTTTGTTTGTTTTTATTCCCTTAGGTGACTCAGATTGGCTGCCAATCTGAGTGTTCTCAACTTAAAGCTATAAACCTTTTCCATTTTATAACATACTTCACACACATAGCCTTATGGCTCTGGGAATTTAGTTCTGCTCAAATGTCCTTGCAAAAGACAACCTGAAGATAGAGGCATCCCTAGCCACCATCACTTGGTGTAGGAGGGCGAAGCTGCAGCTGGGCCTTTGCAGTGGGTTTCCTTTGAGCTTGTTTACAAGACTGGCAGGGCCTCCTGGGTAGGTAGAGAGAATGAGTAAAGGTTCTGAGACAGGAATATGCACACTGAGACACTGAGAGTGGAGTAATGAGTCAACCAGCCTGACACGAATAACTTGTGCTATATTTTGTAATCATTTAGTTGACCTATTTATATCTTATGCACATGTGCATGTGTGCGCGCACACACACACATACCACATACACACACAGACACATGCACACACAGATATACAGCGTGTCCTCTATTCTTCACTCAACTCTTGTTTCAGCAACATAAATGTGTTAGTATAGAGTTCTGGAATAATTTCAGTGAAATATTCTCCCTTCCTCTTGTTCAAGTGAGCTTCATGTTGGCTGCTTGAAGTTAGTTTCCTTCATGTCTTTTTATGCTTAGATTCAGCTTTCTCCTTCAATAGATAAATAACCATGGCTTCTGTCCCTCCAGGCATCTACTCCTGACCCCAAGATGGGAGAGGAGAATGAACAAGGAAAAGGTGATGGGAAAAATCGTCCTGGACTTGAACGGTTATACGTGGGTTTACTTGGGTATTTCTGGTCTGGTAGGTGACTAAGGCTGGTGC

**ENSMUSG00000109028.1**

ATTTTTTTAACAAACAAACAAAAAGGAAAACAAAACCAACGACTCTTTGCCCTCTTAGCCCATGACCTCTCTGTGGTCGTGGTTATGTGGGGGTTCTCTCTTCCTCCCTTCACATACAACTTCTTGAAAGAATATTCTCATTGGTTTTGAGATCCTGGTGTCTCTGGATACCAGTGAGTCCTCATCCCCACCCTCACACCCACCCAGTGACGCCTTTTCTGTTATCTTTATCCCTTGCCATTACACATTAACCTTGGAAACCTCGCTGGCTTTCTGCCTGCTTCCCTGCCAGCCTGAGGAACCCTTTCTCTTGCCAGACATTCCTTTCTCAGGATTTCCCCCTCTATCGACCATTCTTTGTTTGATACTTTATCTTTAAGCAAACTCATCCATTCTTGTTATTTTACTTATCTCCTCTACCCGAAGGTCTGGGCCACCATAATTTCTCTCCTAAATTCTTATCTATCTCCACCTGGATTTCCTCAGGCTCAGCAAAGAGTCAAAGTTCAGAGCTGTCTTTACAGGTCACTGCCCTGTCCTCTTTGTTGAAGCCATTTTGTTTGTATATGGTATAAATTTTTCCTGGTGGAACAATCTCTATACTTAGGCCTTATCTTAATCCAATGATCGTGTCTGAGCTCTAACGCCTTTCTGGGTTTAATTGTAAGCTCAATCAATTTTATCTAGGTATGTTGCCCACACTCACAGCTAGACAGACTAATGGAAGGGTCTGTAGTACCTCTGTGATGCTCATTCTTATGTTCCCATGTTAGCTGCTAGAAGCTTTCTGATATCAAGCAAGAAGGGAGAAGATCGGTTGGCAAATGCCAATTGAGTCCACAACTTTTTACAGGGAAAATTTCCAAAAAGCTCCACCTAGAAATCTTGGCATATGTAGAGGAGACCAGAGGAGTGTTACACAACTATCTTGATCGACCAAAGACACAGAGAAATACAGTTAAAGGAATTTGAGCATGATGCTGCTCCAATATATAATGATTCTAGTGGCCAAGAGAAAATTCATGATACATAGAGACAGCTGTTGCACAATTAACAATATCCACAGAGATCCTCTTCTTTCTTTTCCATGTTTGCTTACAATAAACTCCTACTAAGACCAGCAAATGTAAATGTAAGTAAATGAAAAATTAAATTGAATTCCTACTGAATATGGTAATATGAATTGAGGCATTTCCTACCATTGTCGTTGTCGTCATCATCATCATCACTGTCTTCCTCCTTGTTATTGTCATCATCATCATCCTCAACAACAACAACATGGCCTCTTACTTAAGGGCACAACTGTTGCTTAGTCTGCTGCTACTATTTTCCCTGTTCATTAAGTCACTCATTCAGAATAAAAGCACTGATGATGGACACCATGTTACACGCTATGGATGAATGACTATGACCTTTGTCTTAAGTCTGATCATTACACATAGCAAACACACATCCAGTTAGCACAGTATACCATAGAAATATGAATAGACACTGTGCAGCTCTTCAACATAAATAAATAACCATGACCTTTCTCTTCAGGGACTTACCGGTTGAGTTGAGCAGCCCAACACAGAATGAGTGTCTCTCGGTGCAGCACAATAAGCTACACACTAGAGATGGGTAAGAGAATTGTAGAATCAGAGAGAAAGTCCTGAAATACACCGGGGAATGGCTGAGAAGACTTCACTGGAGAGGAGATGTCAGTAGAGCTGTATGTGAGTAGGAGTCTACCATGCCAACATCTGGATGATAAAATTTGTTTCACAACTATTTTGAATGAAAAACCAAATAGTCACATACTTAACCTCCGTTGAATCTAATTCTTTAAAAGAAAGTCATTGCAAGAGAAACAAGTTAAAATATAACATTTGGGGGTCACGGGACTGATAAATGAGAGAAAAACTGGCCTTAAAGAGTGTACCGTACAATCACCATGCCCTGAAATTAACTGAGAACACATTTGTCAGGAGTGCATGTACCCTACAGAGAATTCCTCCAATTAACATCAAAAGCGATCATTCTTAACCTCTCATGCATTAAGAAAAGCTTGTCGGCACCCAATGTTCTGTTCATAATCAATGGAGCCCTTTATGTATGTTATTTCAAATTTATCACCTTGCATATTACTAAAATGAGTGAATTAAGAGATGAATCTGCTGTAGATCTAGAAAGTAGATTTATTTGTTGTCCGCAGGCTCTCGTGGGCTTTTCCTTTGGTCTCTGAGACCCTTCACTCGACAGCTTCTCATAGGTCAGGGATCTTTATTGACACCCACTGACAGCATGTGTTGGTGCTCATAGACGGGCAGCTAGATCAGAACATGCAGGTTTAGAAGGGTTACTTATATTCTGAAAAGGTCGCCCAGGGCACTATCAACCTATGCTGAGGAGGCAGGAAGATGTGTCAGCCCAACCATAACACATTGTTATAAAAAGCATGCATTACAATAAATAATGCCTGATGCTTTTAAGGTGATTCAAAGATCAACATATTCATAGTCTAGAGTAAAATAGTGCAGCATTCTTACTGGCTATTATACTAACATTAGATTAGGATATATTTTTCAACATAGCCCCTAAGCACATTTAAAAGAATCTACTTTCGAGCTGGATGATAGTGGCACACTCCTTTAATCCCAGCTTGCTTAATTAAATCCCAGTTGGGATTAAAGGTGTGCACCACCATCACTCAGTTTGTGAAAGGCAGAGGCAGAGGCAGGTAGATCTCTGTGAGTTTGAGGCCAGCCTGGTCTATAGAGTGAGTTCCAGGACAGCCAGGGCTGCTATACAGAGAAACTCCAAAATTTGAAAGAAGTGGGAGTGAGTGGGTAGGGGAGCAGGGTGGGGGGAGGGGGGTGAGGGTACAGGGGACATTTGGGATAGCATTTGAAATGTAAATGAAGAAAATATCTAAAAAAAAAAAATTAAAAAAATAACAAAAAAATTGAAAAACAAAAGACAAAGCAACTACAAAAAAGTCCACTTTGGAGATGATACCTGCAATGGTAACGATTAGGAGCAGGAGCAGGACCAGGAGGATGACAATGAGTTTGAGGCCAGTTCATGCTTATCGCAAGACCCTGTCTCATAAATCAAACAACGTAGGTAAGACTTAAGAAGGACTACCACTAGGGCCATTAAACAAAACGCAGAGTATCCAGGGCTACAGCACTTTGCAGGCATAAGAATTCGTGTCTTTACACAAGTTCTGACTGCATGAAAAAGTCCCCACTGTGGCCGTGATGTAGACAAGAAAACAGCCTTCAAATGCTACCAACGCTTTGACACTGGTTTCTGTTATCTTTAGAATGCTCTACAGCCTATAAATAACTGCATCTAGAAACACGAATCTCATAATCTGGATAGAAAGGGAAGTCTTGAGAGGAAAACAAAGAAAGAACATGTAATAGAAGACCAAGGAACTGCTCATCGGTCTTTGCGTCCACGGGCAGTTCGGACTTGCACGCCCCAAGGTCAAGTTCATGGAGTCAGCTGTACTCTGTTCCTGGGCTGACTATTCCTCACCAACTTTCCTCTACACTGCTGCCTATGTTTCCTAACCGAAGACCCTTAAAGCTTTGCTAATTGCTCACTGAACACAGTGACAAGGCCTTCCTAGTTCCCAGCTCAAATCTTCAAACCGATGCCTCTCATGCTTCCTCATCTCTTTTAGTCCAACACTTAGGAAACTCGTCACCTGTCACCTTTATGACATTCCTCTCCTTGCCCCTCCCCCACCCCAGTTTGCTTCCAGTTCCCAGAATGTTCATCCTGAATTAACCTCGCCCTTATTTCCTCTATGTAATTTACGCCTCTCTTCTATTGCTTTCACGAGGATTTCGTTTCCTGCATGGTGACTTGTGTCTCTACTGAGTTGAGACTCTCCTCTTCCCCAAAAGCCATGGCATCTCTTGTGTTCTTTACAGAGTGATAGCCTGTGTTACAGCCACCTGAGAGTTTGTGAGCACTGCCTTTAAATGTTCCCACTACAAGGCTCCTAGAGAGCTGCCCCAGGAGCCGGCCTTCCTAACAAGTTCCTGGGGTTTGCAGCTGTTGCTGCTGACACAAGGCTGACACATTTGAGAAACACTACTTGCCAAAATACAAAGAAGAGTGGAGGTCAAATGTAGAATCCTCTGTCTGGGTGTAGCAGAGAAGACAGGTTTTATAGCGTGCTGTTTAAACGTGGAAAGTCATGTTTGGTTTCCGCATCCCTAGCTTAATCTTTGATCAAGCAGCTAACCCTTCGAAGTTTTCACTTCCTGGTCAGACAGCTGGGGCCATCCTTAACTCTGTTAGAGGTTTGTTCTGCAAGGCCAAATCCAAATCCAAAAGGGATCATGCTACAAAATGTTCTGGAAATGCTTGCTACTAATCCTTACCTATGTATTCCACACGTTTCCTTTAAAAGAAAGGGTTTTTTTTTTGTTTTGTTTTGTTTTTTTTGTTTTTTGCCCAGCTACAGCAGTTTATCTCAAAACATTTTCTTGGACTTTTGACTAAAGAACATTTCCTTTGAGATTAATGCAATAGTAATTTCCATTTCATTTGAAGAGGCTTTGAAAAGTAAGGAATCAGGTACATCAAAATAATTATTTTATAAAGGAGCTAGCATTGAAAAAAATATAATGTATAAAACCAGGTTGCTGAAGCAAAATGTCATCTCCCACTGCTTTGTCAGTCCAAGGCATTTAGTCGTTGCAGGAACATTATAAAACATAGTTCAATTTTCTTTATTGTTACTTTTCTTGGGGGGGGTTGGAGTTATTTTTTTTATTATGGATTTTATGATAAATAACTGAGCTTCAACAGCACTTTTGAATTTGCTAGACATTAAAACAATAGAGAAAGACCATCTTATAAATTGAAACTCACATGTGTGAACACTAAATGCTGGGATTTGATTTCTTTGTTTCTATAGAAACAAAAGAAAATTGAGATTAAAATAAGCTAAAATGGAATGTGTTTTATGAAATAGATAAAGGAGCTTATTTTACTTGTGGGCTGTGCAGTGTGGTAAAAATTTTTACATTTAAAATCCAAGCTGTCTTTGGACCATTCTGCCTTATTATAAGGAAAGGGCCAATGGAATGGGGGTTGGGCTGGAGACTCCAAAGTAGAGGTTCAGTGTAGGATGCCATGCTGCTATGGTGAATAGGTAGTTTGATATCAGCAGAATAGCCAATCTTTTTGTTGTTTCCTTCTTCTTAGAGACATTTCAGTGGCTGGCTGGATAAGGGGCAATGAAAACAGTCTTCAATGTAAAAACCAAGGCCAATAACTTCTGTGGTGTTAAAGTCTTGGAAACAGTTTAGCCAAGACTGTTATCAGTGATAATGAGTCCTTGTGTTGCTAAAAACAGACCAACAGCCAGGTAATAGTGGTGTATTTCTTTAGTGTCAGAACTTGGGAAGCAGAGACAGATAGATCTTTGTTCAAGGCCAGCTTCATCTACAGAGAGTTCCAGGACAGCCAGGGTTACACAGAGAAACCCTTTCTTGAAAAAGAAACAACAACAACAACAAAAGAAAACAAACAAACAAAAGTAGTAAAACCATAACAAAAGACAGACCAGCAGCAGAATATATCAAAGAACCAGCGTTCTCTGTAGCAATGCAGAAGAAAGAGAACCAAGTGATGATGCCCAGGGAAAGAGACCAAGTCTTGTGGGATACATGGTGCTGTTTGTGGAGGACTAAAGGGGTGGGGAGAATCATCTCAACTCAAGGTCATGTTCAGGTGCATGTGTAGTTGGGGTGCTGTCCATGTGTGTGGAGACCAAAGGGTAACCTCAGATGTTATTACTCAGGAATACTATTCACTTCGCTCTTTGAGTCAGGGGACTCTATCTGACATGGCATACTCCAGGTAGACTGAAAGAACCCAGATGCCCCTCAACAGAGGAATGGATACAGAAAATGTGGTACGTTTACACAATGGAGTACTATTCAGCTATTAAAAAGAATAAATTTATGAAATTCCTAGGCAAATGGTTGGACCTGGAGGGCATCATCCTGAGTGAGGTAACCCAATCACAAGGGAACTCACACAATATG

**ENSMUSG00000110393.2**

GCGACCCCCGACCCTGCGACCTCTGACTCGATCACCCGTGACCCCGTAATACCCCACCCTGCGAGCCCCGACCCCAAGACCCTTGACCTCTCTAACCGTGAACCACTCGTGACTCCGAGTCTCCCGACCCCGCCACCCACGATGCCGCAACACCCCACCACTCGACCTCTGACCCTGCCCACGCGCTGTCCCTTGGCGCCCCCGCCTGCGCCCGACCAGCACGAGGCCGGAGGAGGACGACGACGACAACAGGACCTAGAGAGGTGAGTGACAATGTGGAAGCCTAGACGGGGGGGGGCGGGGGGTTGTGACGTCACAGGAGGACATCTGAGGTCATGGGAATCCCTAGCCGGTGGGTGGGGGAGGGACCAGGACGTGGAGTGGCCGAGGCTGTGAGAGGAGGAAGATAGGCGTATACCGGTACAGTCGGGGTCACAAGGCAATATAGATGTTGGGGTCACAGGTTAGGAGTTCACGTATGTTATCGGGGTCAAGTGTGAGGGCAGCTCCAAATAACAGGTCAAGGGTCACGTGGGATGTCAGAGGTTACCTCAGTGGACAGGGAGCACCGCGCCATCCCAGAGACACTCTGTGACCCCCCACTCCCACCCCGCCGAACTCGGACCCCCGCCCGGGCACGCGTGACCGTTCCTTGGGTGGTGACTGGAATGACCCTGCAGTGCTGTAGGCGCTGGTGGAGCTGGCCTGGGGTTGCAACTTCGTTTTTCTGTCCTGGTTCCGCAGGACTTCGGGCAGGCGCGGGGGTGGGTAGGGCGGAATCACGGGTGTCCCAGGGCTGTTTCGTGAATGCCACGTGTGGGCTCCGTGTCCCCGTTAGGGACCTGAGTGGCGATGGTTAGGGGGCGTGGCCACGGCGGACAGTGTGACCTCACAGGGTCACTGCCAGGAAGGAAGGGGGAGAGAGGTGCACCAACCCTGATAGCTGTGTTCCAACCAGCACACCTGAGAGTGACTCTAAAGAGACCTGAGGGGTCAAAAGCCTGGTCTTCGGGGTCATCTCTGAGCAGCTGCTTTGGCCTGAGTCTAAAAATGTTCCGGCTAATCTCCTTGCCAGGGGTGGGGGCTGGGGCGGGGCCGAAGCTGCTGCGGGGCAGGATGGGGCGGGGGTGAGAGTGACCAGAGGGGTCAAGAGTGGCCTGAATCTCTGCCTGTGACCCTGGCACCCCCTGCCCCTGCCCCATCCTTTGATGCTGCAGTTGTGCCTTTCCACTGTCCTCTCCATCCTGCTCCCTGCAGGTCCAGGCCTGCAGGGAGGAGCACCCCCCTCCTCTGCAGCATCCCTGCCTTGTATTTCTCCCCCTCCTCGCAGCCCCCTCTTCCCCCAGGGCACCCCCTCAACCCCCAGGCCCTGGCTGTTTATGATATCATCGCAAAAGGCTGACTGTAAAGATGAGTCATTGTTGTTCTTTGTGTCAGTGGCTTGTCTGTCAAATTTTTCTCTGTGTGTTTTGGTTTTTGGATTTTTCTTGAGTATTTGTGGAGGGAGATGACCAAAGCGAATGTGGAACACCACTGCAATGATACTCTGGAGAGTCATAGCCACAAAGGGCTTGGAAGATGTTCCAGAGTCTGGTATAGGGGACGGTGTGCCCGTGGTAGTAATTGCTCGGAAGGTTAGAGCAAATTTACAGCTGCCATCCTATCTATCTTCATTTTCTACTCTGTGCAAATGGAGGAATCTGCTGGGTGGCCTTTACCTCCCCCTTGTGGCTGTCTTTTATGTGTGTGACACTACTATTCTGTGACTGAGAGGAAGAGACTTTCCATCCCAATCAATTTAGTTACTCACCTTGGAGTTAAATCCTTACCAAGAGGACCAGGACCTACACTGCCCATGACGGAAAGCTCTTCAAGACAGCATCCAGGGCAAAATCAAGCTCTCCCTACCCTGTTTTACAGGTTGGAAAGGACAAAGACCTCATTTATCCCCCATGTGATTGACTCTGTTCCCAGGCCATATGTGACTCTGTAGTTTTGAACTTGGAGCTGTCAGTAGATAGAAAGTTCTTCATCCATTCCAACAAAGTTACCATATCTTCCTTAGACCAGCAGTCTTATTTCAAGGTCTGTTGGGCAATCTGCTCCTCTGTTTACCTTCCTGCTGTTTTAGCCATCTCTTTAGTTTCACTGGCCTAGGAACCCAGGGTTAAAGACAGCTCACCCCAGGGCAGCACAGGCTCTGAACTCCCCATTTCAGTGTATCCTTCCTTGCTCTGAGCTAGCTGCATTTCCCATTTTGTCCTGACTCAGCCCCAACTCCAACTCCCTCTTGTGGTGTCAGGACCTCCTGTAATGGTTAGCTCAGGTTTTGGGCTTTTACAAGTTCCTCATTTATTACTGTCTCTGTGTTATAATTGTCTTAAATTTTCAACATTTCAGAGCTACAGTCATTTGAAGGAGCCTTATAATTGAGGGTGTGCGCTCAGCAGAATGCCTGCTTTCTGTATCTATGAGAGAGAGTGTTGATGATTGATATGGGAAGGCTCTTCTACTGAGGGTGTCAATATTCCTATGCAAGTGGGTCTGGGCTAGATGAAGCTAGGTGGGAATGTGACAGTGACCAAGCAAGAAAGCAAGCCAGGAAACAGTGTTTGTCCTTCGTTTTTCGTTTTTTTCCCCTTCATTCTCTGCCTTTAGTTTCTATTTTAACTTTCCAGAATGATGGACTGTGATCTGACAGAACCATGTACAACAACCATTTATTACCTGAGATGATTTTACTCAGGATTCAATCACAGCAGCAGAGTAGCAAGTTAGAACATTAACAACCCAAAAAAATGGTATCAAAATGAGATTTTGTTGTTAGAATCTATGTTCTCTTTTGAAGGAGTGTGGAAAGTATCAGAAAATGAGATGGCAAAAGTCATAGAAAGCTGTACATGGAGCTTAATTGGTTGCTCTCATAGGACCTGGAAGATTGGAGTGTTGACAGTATTGCAGCCAGAGGAAGTTCAGGTTATAGTATGTCAGTGGAAAATAGACTTTGTGAGAAATTTAGCTAGAGGTCATTTGTGTGATATTTTGGCCAAAAATCTTTCCTACTCAGCTTTTTCCCTGAAATACTGAATAGGGCAAAAATCTAAAAATTATGTACTAATTTGTATATTTTTATTCTATTTACATGTATTTCTTCACTTTTCCCATTTATTTATTTATTCAATTTACATTCTAATCGCTATCCCCTCCCCATTCCATATTACACAGTCCCTCATACCTGACTCCTTTTTATCTTCCCAGGGAGGGTGGGAACCACTTGAGTATCCCTCAACCTTGGCATGTCAAATCTGCAGGTTAGGCACATCCTCTCCTACTGAGGTCAAACAAGGCAGCCAAGTTAGGGGAATAGTTTGGACAAACAGGCAACAGCTTAAGGGTCATCCCCCACTCCAATTGTTGGGGGACCCTCGTGAAGACTGAGTAGCACATAAACTACATATATGGTGGGGGCCTCAGTCCATCCTTTGTATGTGGCCTTCAAATATTGCCTTGAGTGTCAGAAGAGACTTTAGACATTGAACAGTGCAGGGTCTCCTACACACTATGAGGATCACTGTAGACTGAATGCATTTTCATCACAGGATGAACATGAGCCTATAGTGTCCAGGGTCTAAATGCAGTTGATTTTTTAAAAAAAAAGTTCTAGATAAGATGGAACTTGTGGAAGTTCTTGTAGGAGGAGCCTTTCCAGATTCGTTTTGGGGAGATGATAGTGTGTATGAGTTAAAAATAAAATTGCTTTCTTGCTTTTTTGTTTGTTTCTCTAATAAGTTATATATTCATTATAACCCTGATCACTGTCCCAACTCTAGGTCCCTTGCCCTCACAGAGTCCCTCCTCCCAATCTCCTCTGTTTCCCCTTTTAGAGGTGTGGGCCCATAAGTATCCCCACACTGTGGTATGTCACGTCTCTGGTAGATGAAGTGCATCCTCTCCCACTGAGGCCAGACATGGCAGCTCAGTGAGGGAACAGATTCCAAAGATAGGCAGCAGCTTTAAGGACAGCCTCTGCTCCAGTTGTTGGAGGACCCTCATGAAGAATGTACATCTGCTACTAATGTGCCAGGGGCCTTAGTCCAACCTGTGTATGCTGTTTGATTGGTGGTTCAGACTCTGAGTGTCCACACAGGTCCAGGAGAGTTGAGTCAGTTGGTCTTACTGTGGAGTTCCTATCTTCTTTAGGGCCTTCAATCCTTCCCCCAACTCTTCCATAAGAGTCTCCAAGATCTATTCAGTGTTTGATTGTGGTTCTCTGCATCTGTTTCAGTCAGCTACTGGGTTGAGCCTCTCAATAGACAGTTATGCAAGACTCCTGTGTGGAATATCACTAATTCTGTCATGGATTGGTGCTAGCCCATGGGAAGGGTCCCTAGTTGGGCGAGTTTTCATTGGCCATTCCCTCTGTCTTTGATCCGTCTTTGCCCCTGCATTTCTTTTAGACAGGACAGAGTTTTGTGTGTGTGTGTGTCTACTGTGTTGTGGATTAGTTTAATCCTTTGGCTTTCTTCTCATGCTCCCATATATTCACCATTTGAACTCTGTCCCTATATCACATAAGATACACAAGAGGTATTCACAAAAGTATAAACATTTGGGGCTGAAGAGTTTACTCAGTGGTTAAGGGTACTGACTGATCTTCCAGAGGTCCTGAGTTCAATTCCCAGCAACCACATAGTGGCTCGCAGCCATCTGTAATGGAATCTGATGCCCTCCTCTTGTGTGTCTGAAGACTCACCTACAGAAAATAAATAAATCTTTAAAAGTATAAACATTTGCGATTTAATGATAAGCTATTTTGGAAGCCTCGTTGTTTTATATTTTTACTTTATTCTTTTGTTTTTGTTTTTTTAAGACAAAATTTCTCTGTGGAGCCCTTTCTGTCCTGATAGTAGGTATAGAAACCATATAAGCTACCTGATATATTTTTGTCGAATAAGTATATGTTTTTGTGTAGCTCTTAATCCACATCTTTAATTTTAAAATTTGGCCACTTATATAGATGATACAATATTTCTCAGGATGGCATATGCTCATGTTCATACATCAGTACTACCTGAGGTTTCAGAGTCATTGGACTCTAGGTGTGTGACATTCTGCCTAACTCTTGTGTTTGTTCTGAAATTTTTAAATAGAAGCATTTTGCCTGTGTGCATGAATATGAATATCATGTGTGTATTCATCTTTAGTGTTTTTGGAAGACGGCCTCATAACTCCTGATCTTTTTTGTTTGTTTGTTTGGTTGGTTGGTTTGGTTTTTTTGGGGGTTTTTTGGGGGTTTTTTTTTTTTTTTGGTTTTTGGTTTTTTTTTGAGACAGGGTTTCTCTGTTGTAGCCCTGGCTGTCCTGGAACTCACTCTGTAGACCAGGCTGGCCTTGAACTCAGAAATCCTCCTGCCTCTGCCTCCCAAATGCTGGGATTAAAGGCGTACACCACCACGCCCGGCATAACTCCTGATCTTGAAGTAACAGTTTGTTGTTGGTCTCCACATAAATGCTAGAAATTGACCACCATTATGTGGAAGACCAGCAAGAGCTTTTCACTGTTGAGCTATTTCTCCGGCCCTATGTTGCTTATTTATGATCAACATTTATATTACTAGTATTTTCATCTCTCCATTTGTAACTGTTTAAACCTTATTTAAGTTCTTTTTTTAATTTCTCCTATTCAAAAGGTGCAAAAGCAACACAGGTGAACACTATATTTCAAGTTCCTTCTAAAATGACAGGATGATTAGATTATAACTCCTGTGTGAAGAAATCAGTGGAGGAAGCACCAGAAATATATGGATATATTGTGGAGGAAGTGTACATTTACTTTACAGTGATGTCAGTATCATGCTTTGTGTTGTACACATTTATGGGATATTTTAGGATTCAGTGACTTATGATGATGTGCATGTGAACTTCACTAAAGAAGAGTGGGATTTGCTGGATCCTTCCCAGATGAATCTCTACAAAGACGTGATGCTGGAGACCTACTGGAACCTCATTGCTATAGGTAGGACTGTGAATTTTCTCTTGCTTTTCAAAATAAGAGAACAAATGTTTCTTGGTTATTGATGAACTTCTGCCCTTTCAATTGAAAACAAGGAAGATTGATGTGAATAAATCAGGTTCATTGCTATGAGAATTCACAGGAACTTGAATTTTGTCTAATTTCCAATAGTCTCTCATTCGTTTTTTTGGTACTGTGTTTTAGGCTACAATTGGGAAGATCATCATATTGAAGAACAATGTCAAAGTTCTAAAAGTCATGAAAGGTAATTTTCTTTTTTGGGGGGGGTGGGGGGTTGTTTTTGGTTTGGTTTTTGGTTTTTGGTTTTTTGAGACAGGGTTTCTCTGTGTAGCCCTGGCTGTCCTGGAACTTACTTTGTACACTAGGCTGGCCTCAAACTCAGAAATCCGCCTGTCTCTGCCTCCCAAGTGCTGGGATTAAAGGCATGTGCCACCACCGCTCGGCATGAAAGGTAATTTTCATGTGCAAGCTGATACAACTATGCCTCTGAGGAAATTTTAATATGTTCTGGAAGCACTAAGGAAAAGCAAGAGTGTCAAATATCAGTCCTTTAAGTATAGCTATAATTATAATATTCTCACAAAACCATGTACCTCAATGTCAGTTATCTAATTTTTGTTTGCAAGGTATTCCTCTAAGAAAGAAGACAAGGAAATATGCCTTAAGAGATACCTTTATTTGATATGTAGCTCCATTAGAGCTATGCTGTAGAACTACTAATCTGTATAGTATCATAATATTTAGATGCTGCACATTGAATAGTGATAAATATATATCCAAAACCTTCTGATAAGCAAATAGTTCATTGTAAAGTTGGTGATACTCATATTCTTGTAGTGACATTGCTAAGTTTATTGGTCAGCTTATGAGTGTCAGGAATGTTTTCATGAAGAAACCTTAATCCATATACCACATGTCTACAAGAAACAAAAAGTCATATGGTGTGAATGCAATGTGGAAACCTTTCATTTATTCTTCTTCTCTTAATGGATGTATCAAGTGTCAGAAATGATAAAACCATGTGAGCATAAGGCTATTGAAAGGACCAATGTAACTGTTGTTCCATTTTTATTCAGATACATGAAATACATTATATAGAAATCTCATATAAAAAAAAGGATGTTATAAAATGATTTATCAGGGTATAATGAGTATGGTAAAACTTTCATATGTGCCGAATATCATTGCAGGAATGTAAGAAGTCATAGTGGAGAGAAACCCTATGAATGTAATAAGTGTGGTAAAGCCTTTGCAAGACCCATTTATCTCCAAAATCATAAAAGAACACATACTGGAGAGAAAGCTTATGAATGTAATCAATGTGATGAAGCCTTTGCAAGACCCAGTCAACTCCAAAGACATAAAAGAATGCATACTGCGGAGAAACCCTATGACTGTAATCAGTGTGGTAAAGCCTTTGCAAGACCCAGTCATCTCCAATATCATAAAAGCACACATACTGGAGATAAACCTTATGAATGTAATCAATGTGGTAAAGCCTTCGTATGGCACAGTCATCTCCAAATACATAAAAGAACACATAGTGGAGAAAAACCTTATGAATGTATTCTATGTGGTAAAGCCTTTGCAATACCCAGTCATCTCCAAAGACATAAAAGCATACATACTGGAGAGAAACCTTATGAATGTAATCAATGTGGTAAAGCCTTTTCACAGGGGAGTCATCTCCAATATCATAAAAGAACACATACCGGAGAGAAAGCTTATGAATGTAATCAATGTGGTAAAGCCTTTTCACAGGGGAGTCATCTCCAATATCAGAACACATACGGGAGAGAAAGCTTATGAATGTAATCAATGTGATGAAGCCTTTGCAAGACCCAGTCAACTCCAAAGACATAAAAGAATGCATACTGCGGAGAAACCCTATGAATGTAATCAGTGTGGTAAAGCCTATGCAGAAGAGAGAACACTCCAATATCATAAAAGAATACATACTGGAGAGAAACCTTATAATCAATGAGGTGAAGCCTTTGCAACACCCAGTCATCTCCAGTGTTATACAATAACACATATTGGAGAGAAACCTTATGAATATAATCAATGTGATGAAGCCTTTGCAAGACCCAGTCATCTCCAAAGACATAAAAGAACACATACTGGAGAGAAGCCTTATGAATGTATTCAATTTGATAAAGTCTTTTCATGGCACAGTACTCTCCAGTATCATAAAAGAGCACATACTGGGGAGAAACCTTAGGGATGGCATTAATGTGGTAAAGCCTGTTCACAAGGGAGAAGTCTCCAAAAATATAAAAGAACACATACTGGAGAGAAACCCTATGAATGTAATCTATGTGATAAAAGCCTTTGCACTAAGTTCTTGTCTCAAAAATCATAAAATAACC

**ENSMUSG00000110399.1**

GTTGAGTCACCCAAGGGAGGCTGTGTGTGCTGCAGACGACTGGACAGGAGGAGCGGAGCTACCCAAGGCTGTGCAAACCCAGATGATGCCACCAAGGGCCACAGGTGCTGATGTAGAGCTCTGCTGACTGAGTTTCCTAACTAGGTCTTGGTCTTGTGTTAGTACAATTTTCTCTTGCTGTGTCCTCGTCCCTCCCTTTGGAATGGGGATGTTTGTGTGTCATTGTATAATGAACTATCTAGCTTGTGTTTTCGTTGTTTCCGGCGTTCATAATGAGAGTCTTTAGTCTCAGAATAGACCTTGGACTTTTGAATGGTGTTGGTAATCAAAAATGTGACTTTTTAAAATTTAAAGCCGGGCGTGGTGGCGCACGCCTTTAATTCCAGCACTCCGGAGGCAGAGGCAGGCAGATTTCTGAGTTTGAGGTCAGCCTGGTCGACAAAGTGAGTTCCAGGACAGCCAGGGCTACACAGAGAAACCCTGTCTCATAGAAACAAAAACAAAACAAAACAAAACAGATTAAAATTTAATTTCATGTGTATGAATGTTTTGCCTGCATATGTGTCTGGATTCTAGACATGCCTGGTCCCTGATGGAGGCTAGAAGTGGGTACTAGATCCCCTGAAACTGGATTTACAGCTGATCATGAGCCTCCATGTGGGAGCTGGGGCTCAAACCTGGGTTCTCTAGAAGATCTACCAGTGTTCTTACCTGCTGAGCCATCTCTCCAGCCCTAACACTGGTCACTTTTAAAGTTGAATTAATGCATTTTACATTATGGGATGGCCATGAGCCTAGGGGGCCAGGGTGGACAATTCAAAGTGGTTCAAAGTGATGTGTCTGGGTGTCAAGTTAACAAGAGAGGGACTGTGATAGTTAATTCACATATGTTCACATGTATTTGTGTGATATGACTAGGTATAGAAACCAAGAGGTAGATATTGGATAGCTTCCATCTCTCTCCACATTAATTTTTAGGCAGAGTCTCTCTCTGAACCTGGTGCACACAGACTCAACCAGAGCACCAAGCCTGAAGAAGGCTGTCTCTGTTTCTCAGGCTTTATGGGATAACAAGAACAGATCACTCTGCCCCACTACTTTATGTGACTGCTAAGATAAAGCTCAGGTCTTCCTGTTTGCATAACAAGTGCTTTATTGACAGAGCCAATCTCCCCAGTCCCACGAGAGTTCATTTTTATTGTCAAATTGAATGTATTAACAAATACGTAGTAGCTGGGCAGTGGTGGTACATGCCTTTAATCCTAGAATTTGGGAGACAGAGGCAGGTGGATCTCTGTGTTAGTGGCTGGCCTGGTCCACAGACTGAGTTCCTGGATCACCAGGGCTAAATGGAACAACCCCATCTTGAAAAACAAACAAACAAATGGCCCCAACCAAACAAAAGAAACATCCAGTAGCCTGGTGTAGTGGTGCATGTCTTTAATCCTGGCAGAGGCAGGTGGATCTCTGTGAGTTTGAAGCCAGCCTGCTGTACAGAGAGTTTCAGGACAGCCAGAGCTCTGTCTTGAATGACCCCGGCTGCCAAGGAAGGAAGGAAGGAAGGAAGGAAGGAAGGAAGGAAGGAAGGAAGGAAGGAAGGAAGGGTGAGGTACACTTTTGGGTGTATCTTTGAGGGTATGACTGTCCTAAATGTAAGTGGTATCTTTGCATGGGCTGGTATCCTGGGCTGAATTAAAGAGGGGGAAGCGCCAGCATTTTCTGTCTGTTTCCTGTTCCTCTGAGAATGAATTGAACAACATTGTAATCTTTAAAGTTTTATTTGTTTGCATGTTTATGCCTCTCTCTCTCTCTCTCTCTCTCTGTGTGTGTGTGTGTGTGTGTGTGTGTGTGTGTGAATATGTGCAGAGTGCTTGTGTCTGTGCACATCTACAGAGGCCCGAGGAGGGTAGTGAGTCTCTGTCAACCTCCACCTATTCCTTTGAGACAGGATCTCTCCCTAAACTGGGGCTTGCATTTTCTCAGAGTTGAAGCCAAACAGAAGCAATTCTCATGTCTCTCTGCCCCAGAGCTACCCTGACAGCCATGTGTGGAACAGCCAGCTTGGTGTGTGGGTGTGGGGATTCAAACCCTGATCCTCATGATCACACAACAAAGCACTCTTAACTGCTGAGCCTGTCTCCAGCTGCAGCACTGTAGTTTTTGTGTGCTTCTTCTGTTTAATCCTTCCCATCTATCTTGAGTATTTTGTTGGCTTTACAGTACTGTAACAAATACTTAAGATAAACTTATGGAGCAAAAAGGTTTGTTTGGGCTATAGCTTTGGCAGTGTCAGTCCATGTTAGGTGGCCCTGTTGCTCTTAGGTCTGCAGTGAGATCTCCATCTCACTGTTTAAGTCATGGGTAGGTTGTAAGAAAGAAGAGGCTAAAAGTCCTGCATCCCTTAAGAGAGCATGCTCACAGTGTCTACTCCAGAGCCTCCTGCCAGGCGCCAGGTCCTCACTCCAGTTCCCTCCGACTTCCATAGGGCTGCTCTGGAGATCAAGCACGGACCTCTGGGTGACGCAGTTGATAACTCATTGCTCTGCCTTCACATGGGAATGTAGGGTGTTTTCCTCAGTTTCTCTCTACCTTAGGTTTTCTTTGTTTATGTGCACTGGTGTTTTGCCTACATGTGTGTCTGTGTGAGGGTGCCAGATCCCCTGGACTCAAATTACAGTGAGGACTGTCATGTGGGTGTTGGCCCAAGGGCTCGCCATGGGCCGTCATGTCCTAGTACACGATGGGGGTTGACCATGGCGATGGGGGTTGACCATGGCGGCAGCAAGTGGCAAAAAGCAACCAAAGCAACCGCGGCGTCTGCAAACGCCTAGATTTTTGGTTTTGGTTTTTGTTTTGTTTTTATTAAAAAGTTTGGCAAAGAACCAGGGAAAGTTACCTGCTCAGTAACCCCAGCACTTTGGAAGCTGAAGCAGGAGGATGCTAAGTTTGATACCAATCTAAGTTGCAAAGCAAGATGCTGTCTCTTAAAGAAATAGAGGAATATGGAGGACAAGGAAATAAAGATGACTTCTTCTGGTCTGGGCTAGGGTTTCAGCATGGGCGTGGGCTGTATAACCAAAATAACAAAGACAAAGAGTAATTCTGTTACCCTGGGAAGGTAAACCACGGATTCCATTACTTGAATAAATATGGCGTTATTTGAGTTGTCTGAAAAGTTTTATGGCTTTCAAGCAATGTATTCATTGTCATGCTGTCAGAATTACTTATATAAATCTGTATATATGCCCTTGCCCTCTTCTCCTTTTGTCTTTTGTCTCCTTCTAAGTGTGTGATTTTCCTACCTCAGCTGAGTAGTTGATACTATGGGTGCTGTCACTGTCCAGCTCTCTCCCACTTCCCCTTCTCCCCCCTCTCCCTACCTCCTCCCTTTTCCCCTCCCCCTTCCCCCCTTTCTTGGTAATTTATGTCTGGTCCCTTTCCTCCCTCCCAGCTGAGGTTTATCAGTTCCATTGTAATTTTAAAAAGCTTTTAGTCTCATGGGTCCCTGCTGTTCTTGTCTTTAATTCTCTGGTCTTCAATCTTTATTTATTTATGTATTTATTTTTCTTTCTTTTTTCCTTCCTTTCTTTCTTTCTTTCTTTCTTTCTTTCTTTCTTTCTTTCTTTCTTTCTTTCTTTCTTTCTTTCTTTCTAATTTTATTTTTCGAGACAGGGTTTCTCTGTATATTCCTGGCTCTCCTGGAACTCACTTTGTAGATCAGGCTGGCCTTGAACTCAGAAATCCACCAGCCTCTGCCTCCCAAGTGCTGGGATTAAAGGCTTGTGCCACCACACCCGGCTTATTTATTTTTTTCCTTCTGATTGTTTCAGGATTACTTTGCTCTTTTTCTTGAAGGTAGGGGTTTAGAATACAAGTACTTCACAGCCTGTCTCCCTCTAAGTCTCCGTTTAACTGTACTTTGTAAACTGTGACATGTTGTATTTTAAATCAGTCTCAGATATTTCCTGATTTTTTTCTTTAGGAGGGAGGGAGTGTTGGGAATTGAACCCACGGCCTGGTGTGTCAAACCTGTATTCTACCTCTGAGTTACATCTCCAGCTCCAAGACTTCCCTTTAAAAAAAAAAAGCTAAAAACTTTTAATTAAAAAACAAAACAAAAAAAAATCCTTTGGGGACACAGGTGTTCCTAGTCTGCTTTCTTCTGTCACTGTAGTAAAGACTATGACCAAAAATCACTTGGGGAGGAAAGCGTCTATCTGCCTTACATATTCTGATCACACTATCACTGAGAGTCGCCAAGGTAGACCACAGCTGGTCAGTCTCAGAGGGAGGACTGGACAGAGGCTATGGAGGAGTGCAGCTTACTGGCTTGCTCCTGTGGCTGACTCAGCCTTCCCTCGTATTCATGTGATACGGCTTTTCTCTTTTGAGACCTACCTCCCTATGTAGCTCTGGCTGGCCTGGCATTCACTATATAAATCAGATCAACCTTAATTTTTTTAATGATTAAATACTTCTTTGTTATATGAATTTTGTTTATTCACTTAGCAGTTTCCTTTTTTTTTTTTTTTTTTTTAAGACAGGGTCCTACTACATCACACCGATTGGCCTGGCACTTGCTATGCAGACCTGGCTGACTTCAAACTCAGAGATCTACCTGCCTCTGCCTACTGAATGCTGGGATTAAAGGCATGCACCACCATGCCTGGCTTAGTTTTATTTTATTTTATATATTTATTTTTGTTGTTTCTGAGACAGGCTAGCCAGGAACTCACTCTGTAGACCAAGCTGGCCATAAAGTCACAGAGCTCTGCCTGCCTCTATTGTCAGCTTGACACAAAATATAGTGACCTGGGAAGAAGGGACCTCAATTGAAGGTTTGATTAAGAGTGGCCTCTGAGTGGTGGCGCACGCCTTTAATCCCAGCACTTGGGAGGCAGAGGCAGGCGGATTTCTGAGTTCGAGGCCAGCCTGGTCTACAGAGCGAGTTCCAGGACAGCCAGGACTATACAGAGAAACCCTGTCTCCAAAACAAAACAAAACAAAACAAACAAACAAACAAAAGAGTGGCCTCTGGCTGTGACTCTGACAAGGTCTTGACTGAGGGTTGCTGTGGGAGGGCCCAGCTCACTGTGGGTGGCAGCATCTCTGGGCAGGTTTCTATAAGAACTCTAACATGATTGAGAGCTGTAAGCCCAAATCCTGCATGGTTCGGAGCAAGGTGAGGGTACTGTCCTAGGTCTCTCAAAGCCACAGGGCAGATCCCTCACAGGAGTGAAGAGGAGAAGTTCTGTCATAGCTGGAAAATGTGGGCTGTGGCGGAGCTTCCTGTGTCCCACAGAGAAATCTTACCAGAAAAAGGACTTAGGAGAACATCCAACACAGGAACTGAAAACATTGGTTTAAGACCGTGTTTATTTATACTTTGAAAGTTTAAGGAAAGAAAAGTTTCTAAAACCACCTGGAACGGATACCTCGATTAGCAATGCAGAATTTTTAATAGCTCTTGGTTCGGTTTGACTATTTTTGGGTCAATTTTGTTTCATCTGGACTTTGAGTGAGCAAAGCCGTAGGCAGTGTTTTAAACTCTTTCGGGTAAGTAGAGTCTGTAGTTTTGTCCCCTAATGACGTGTGAACTCTTTCCTTCTTCTTGCTCAGGCACTTGCTGGACACTTGTCGTGGGTTATTAATTCCCTCTTGGAAAAGGACCAGCAATTTTCTGGCATTTATTATTTATTTATTTCTGAGACAAGGACTCACTATGTAGCCGTGGCTGTCCTGAAACTCCAAGTTCTTCTCAATCTTCCTGGTGCTGTGACCCTTTAATACAGTTCCTCACGTTGTGGTGAGTTCCCCCAACCATAAAATTATTTTTGTTGCGACTTCATAACTGTAAATTTGCTCCCGTGAATAGATTGTAATATATATGTATCTGTGTTTTCTAATGGTCTTAGGAGATCCCTGTGAAAGGCTCACTCATTCGACCCTTTCACCTCCTCTTCCTGGGCGGAGGAGTTTATGTATCTCATGACTTCCTCTTTCTTGGTTAGCTTCTTTATTTTTGTGTGTGTCACACCCTGCAGCCTCCTTAGAGATAATACTTAGATAAAACATTTTGTACTTGGGGAAAAAAAACATCCCTCTATGATTAGCATTTTAGTCCAGTGTGAAATCTGGTTAGAAATTATATATATTGAGGCCATGTCTCACTATTTCTAGATCACAGTTATTTTATTTGTTTATTTTTGTTTTGTTTTCTGCTGTCCTCCTTCCTACGGGGCCTCACAAGGAGACCCTTGAATTCCACGGGTTGCTGAAGATGACCTTGAACTGCTAGACCTCCCATCTCTCCCTCTGGAGTGCTGGGATTATAGGCCTGTGTACCACAGGCTGTCTATACATCTTTAGGGCTTCTCTCTGGGTAGGCGAGCACTCTATCAATGAGCCACATCCCCAGCCCACCTGGCAGCTTTTGAGGAGTTCAAAACGTCCTGATTGCTCGTATTATATTACAAGTATTATATTTTTATATGTGGACTATGTATTATAACAGAAACAGGATGAGTTGGGGTCCTGGGACTCCCTTCAAAGGGTTCACCCTCGATGACTTTCTTGGATCAACCTCCAACCTCCTAAAGGTCCCATACTCCGACCCACAATGCAATATCACTGTTGACCAAGCCTGTATGGGTCTTTGGAGGACATTATATCTTCACTATAGCAGTGTCCAATAGCTTCCTTTATTCTAGGTACTATGTGGTCTCTTTCAGCCCACACATAGATGGCTTTAGTTCTGACAACATTTCATGGATTATTTATTTATTTATTTATTTTTAAATATTTTTATTACATATTTTCCTCAATTACATTTCCAATGCTATCCCAAAAGTCCCCCATACCCTCCCCCCCACTTCCCTACCCACCCATTCCCATTTTTTTGGCCCTGGCATTCCCCTGTACTGAGGCATATACAGTTTGCATGTCCAATGGACCTCTCTTTCCAGTGATGGCCGACTAGGCCATCTTTTGATACATATGCAGCTAGAGTCAAGAGCTCCGGGGTACTGGTTAGTTCATAATGTTGTTCCACCTATAGGGTTGCAGATCCCTTTAGCTCCTTGGGTACTTTCTCTAGCTCCTCCATTGGGAGCCCTGTGATCCATCCAATAGTCATGGATTATTTCTTTAATGATTTATTTTTCCTTAGTTTCTCCTGAGTTTTCATTCTAGAATTCTATTATTTGAAAATTGGATGTCTTGGGCTAGTTGTACAGCTTCTCAATATTTCTTTCTATTGTCTTACAATTCTTTTTTTTTTTTTTTTTTGCAAAAAGTATTGTACCTTCAAATATAACCCTTATGTGTTTATCAGTCACCAACAGAGACAAATCTAGAAAGATAATATCTTTCTAGGAGAAGAGTGTGCTATCTTAATGTACTTTCCTTCTTAAATTTTTGGGTTTTTTATTACATTTGTTTTGTGTGAGCATGTATGTGCAGGTCTGTGTGTGTGCTCATGTACACACAAGTCACAGTGCACATATGGAGGTTAAAGGACAATTTGCAGAATGGGTTTTTTCCTTCCACCTTCAGGTCTGAGGGATCAAGCACAGGTTGTCATGTTAGGTGGCAGCTATCTCTACCTATTGAGGCATTGTCTGGCCTTAATTCACTTTCTTTTGCTGTAGCAAAACCTATAAGGTAGTTTATAAAGAATAAAGCTTTATTTAGCTCACGGATCTCGGAGCATGCTACTCTCAACTCTCAGAATACGGATGGGTATGGAACCAGGGTCCTGATGGAGCTAGACAAGTATTCTCCTGCGGAGACACACCACAGCCTACCTCTTCCTTCTTTTCTTTGAATTATGGTCTTGTTATGCATTTCCTAGACTATCATGAAACATGCAATTCTCCCTGTCTCAGCTTCTCAAGAATGTACAGAGATAAGGGCCCTGTTGCTGCATCATGACTGACCCAAGGGTTCCACACATGGAGAATGAGGAAGCATGCCAGTTTGGGTCATAGATGATAAGGTCACCCACATCCTCAAGGGGCTTTTTCTCACTTCATCTCATCTGTGCTCAAAGGCTGACCCTTACCTCCAAATATCTTCCAACACAGGACTTTGGGATTGTTTCCAATATAAGAATTCTTGGGAGCCATATTTAAAGACATGTCTGGGGGCTGGAGAGGTGGCTCAGTGGTTAAGAACACTGACTGTTCTTCCAGAGGACCTGGGGTTCAATTCCCGGCCACCAACAAAGCAGCTTACAACTGTCTGTAACTCCAGTATCTGATACCCTCATATAGACATACATGCAGGCAACACACCAGTGTACATAAAATAAAAATAAATTAATTGTTTTTAAAAAGTATATCTGTGCCGGGCGGTGGTGGCATACGCCTTTAATCCCAGCACTTGGGAGGCAGAGGCAGACAGATTTCTGAGTTCGAGGCCAGCCTGGTCTACAGAGTGAGTTTCAGGACAGCCAGAGCTACACAGAGAAACCCTGTCTGGAAAAACCAAAAAAAAAAAAAAAGTTTCTCTGTGTGTATTCTGTGTGTGATGTGTGTATATGGTGTGTGTTAGTGCCAGTATGAATATGCCAAGAAACTCACGTTGGGGTCAGAGGTCCTTGCTCTCTACCTTGAGACCACCTTGGGTCTCTCGCTGTTCACTTCTGTGTATGCCAGGCTAACTCAGGATATCCCCGCCTCTGTCTCCCAGAGCATTATGAGTTCAGAAGCTCATGACCAAGCCCAGGTCCATATAAGCATGAGTTCTGGTTACATGAACTCATGTCCTCACATCTGTCAGGAAGTGCCTTGCCCATTGAGCCACCTCCTCAGCTCCAACAGTGCCAGATGCTGACAGAAACATTGCCGAGGAAGTTGGGAAGGAGAACCTAACTTTCAGATTACACTGTCCTTGGTCCAGGGAATCAGGAAGTTGCTGTTATCGTCAAGATACCAGTGAAAGGCAGGGTATCTCTACAGATAACTCACTTGCGTTATAGCCTGGAGGGTGATTAGCTATACCATAGAAGCCAGACGCTCCATAACCCATGTCTGCCCAGGATACCTGCTGCTTGCTCTGGGAGGTCAGTAACTCTGATCTCCAGTTTGCCACCGAAATAAATCTGCACACAAAAGTAAAAGAATTGAGAAGCTAAAAGTGAACAGACAAACAACAGATAAACAAAACACCCAGATAAACAAAAACCAAATTTATCAAAAAACAACCTTGTGGCTATTTTTACCTTTTTCTCCTTCTAGAGTCTTCCTTGGACTCAATTTCTTTCTCTCTTTCTCCCTTCCTTCCTCTCTCTCTCTCTCTCTCTCTCTCTCTCTCTCTCTCTTTCTTTTTTTTTTTGTTTTTCGAGACAGGCTTTTCTTTTCTTTTCTTTTCTTTTCTTTTCTTTTCTTTTCTTTTCTTTTCTTTTCTTTTCTTTTTTTTTTGAGACAGGGTTTCTCTGTGTAGCCCTGGCTGTTCTGGGACTCAGACTGTAGACCAGGCTGGCCTTGAACTCACAGAGATTCCACCTGCTTCTGCTTCCAGAGTGCTGGGACTAGAGGCAGTCACCACACTTCCTGGCTTCTAAATCTTTTTTGGAAGGGGTCGGCGGGGGACAGGGTGAGAGTCTGAATCTCTCAATGCACACTTAATGCCATAGCCCGTCTTTCACCCTGAACCCTCGTGGCTTCTTTGTGCTCCATTTGTCTTTTGAGTGAATTTTTCCATGATGTCTTCCAATTGATAGATTTATCAATGTTTTTCTGTCTTTGGTTGCATACAGTCTAGAGTTTAACCTGAAGGCTGACCTTTAAGAAAACCACTGATTATTTTTACAGCTCAGATTTCCAATGGGTTCTTGGGTTTGCAGCCCCCTCTCCCTTTACATTTTCCACGAGGTCCATTTCTACCGTTTCCATTTAATATTTCTTATACATTTTCAGCAGTTATTCTGCCTTTCCTATGTATTGGTGCTGTGTAGATAGAAATGTGTGTGAAGGTCTTTTCTACACTATTCTGTAAACCATTGTCAGCTAGACTCCACGTGGATGATTTTACCACCCTCTAACGAGGAAGCGTTCGATGTCTTCATTGTGTGTATTTTGGAATTTTGATTTGTAGACCTTGAAAGGCAGTTCTCCCCACATACACTCAATGTGTGTGCATGTTTGTGTGTGCGTATGTGTGTGTTGTGTATGGTGTGTGTGTGTTTATGTATTTTCTTTTTTCTCTTCAAGGTGCAGTTGGTAGTTTTGTGGCTGCTTCTATTTGGAAGCTCTCCTAACCTCTCATTCCAGGAAATAAAGCCCTATGTTGTTTCACGGATCTGCATCCTGTGCTAGACTAGTGCCCATCTGGAAGTGGAGCTTGTGGGTGGCTTCCTCCTTGTGAGGCTGTGATCCTCTCACCCTCTTAGCTCATCCTACCCAGCTCACAGAATCTCAACGCTGCATCTTCCTCCGG

**Gm38850**

GGGGAGACCTTGGAGGAGGTTCTCTGGGAGTCTGGGGGGACCTTGGAGGAGGTGCTCTGGGAGTTTCACCCGTATTTCTCATCACATTTTCAGTCTTCCCACCCCCATCTTCCTTCCCAATGGTGACCCCTGAGTTGAGAGTTCACTCCGGCCCAGTGGTGCCCATGCATCACCTGGGAAATGAAGCACTTGCTTGCCCTCCCCCACGCACTCCCAGGGAATACCTTCAGATGACTCCCACAGACACCGAGGTGTGCACACTCAGTTCTCTCTGCAGCCCTGCCTGAGTCACATGTATATGACTGTGTGCACTGTGTGCACATGCATGCACACCTGCAGTTACCGTCAGGCAACTGTGCCTATGGAGAGTAGGGAAGATGGTGAGCAGAGGTCCAGGTACTGTCTGTGTGGAGGGGCCTGCCACCTGTACCTCACCTTGCCCCGCCCCAAGCGCAGCTGACTCTCCTGCCTGCTGCTGCTCCTGACCGGAACCTTTAGTCTCCAGATCAACTTGTCCATCCTTCAGTGACATTGCCTAGTGGTTCTCAGCTCTGCTGCACTTTAGACTCACTGGCAAAACTTATAGTTAATTCTTTAAAATATCTTAGATGCATTATACATATATGCGTGTGTTTTCTGGGGCAGAGAACAGCTGCTTATGTCTACTGAATCTATAAAATAAAATGGGAATGGGAAGCGTGCAAATGTGCAACGGTGAATCAAGTTCAGTGCAGCAGAGGCCTGTGTGTCCACCCTGCCTTCTTAGGACCAATACTGTGGCAGGGTCCTACACTCCACTCACAACTCTCCTTCCCCATGGAGGCTCTAAAGCTAATTGCCCAGCTCCAGTAAGGACATTGTTTATTTGTTTGTTTGGTTGGTTTTGTTTTTGTTTTTTTAAAACAGGGTTTCTCTGTCTAACTCTGGATGCCCTAGAACTTCCTCTGTAGACCAGGCTAACCTTGAACTTATATAGATCTGCCTGCTTCTGCCTCTAGAGGGACTAAAGACATCCACAGCTACAGCCAGGCAGTATAGACTTCTTTAAGGGAAAAAAAGAAGGCAAAGGCAATCATAACTATAGGCCTCTACGATGCTGTAATGCTTTGAGGAGCCTTAAAACAGACAGTGCCCCAGCCCTCAGGTAGCGACTCTGAATTATTAGCTTTGGGTTGAGGCTGTGCACTAGAATCCTTCAAAGCTCCCCAGGTGGCTCTCACGTAGCTGGATTGAGACCATGGAACTGCTTCAATCTGAGCATCCATCCACCTGAAATACAGGCCACCCACCCAGATAGGTGGCCTTTTCAATGCCTGTGTGAACAGGAAGGTCTACTGTGATAGTGGCCCAGCTTTCCCCAAGCTCTCTAAAGACCCTGTCTCTGCAGGTCAGGTCAGAATTGTGCTATAAGTTTCATCCAAGAAAAGACAGACCAGACAGACCCTTACCAGGCAGGCAGGAACTGGGAAGAAAATCAAACCAACAGCTCTGTTCCCTTGCAGGATTTCCAGTGAGTGTGACAGTGTGGGCCTTCTCGCCTGTTCGTTGCTCTGTGACTGTGGCTGGCTGTTAAAGTCAGAGCTACCTTTATGCTTTCGGGGTTTATATAGCAGAATGCTCTTTGGGTAAAGATGGTAGCCGTGGCCTCCTTTCTTCATCCTTATAGTTTCTCTTGTGTCTGCCTCAAGTTCTTCTTTCAGACCTTTTAATGTAGGCATTTATTTGTATTATCCAGTTTTAGCCCTTTAGGCCAGGGCTTCGTGGTGGAGTGCATGCCTGGCATTCACAACCTTGGGTCCCATCCCAGCACTGCAAAAGAAAACCAGACTGCATCCTTTGAGCAAAGACTCCCGAGTCTTCTGTTATCTTATGCCTTTTTCCTCTGTGAGATTGCTACACAGTGTGTGTGTGTGTGTGTGTGTGTGTGTGTGTGTGTGTGTGTGTGTGTGTGTATGCAACAGTGCATGGGTGGTTGTTGTTGTTTTAAATAGTTCAGGGATGTTTAGTTTTGCTTACTATGGTCCAAGACCTGAGCCCTGTGTGGGATACTAGACAAATTCACACTTGGGTGTGAAACCAGCAGCCAGACCCGTGCCACAGAAGTGACAAGTCTGTCTAAGGAGAAGAACTCAGAAACACCTTGTTAGTCCATAAAGGGCTGCTGCTGTGGCACGAGAAGGGCCATGGAGACAGAGTCCCAGAAGGTGACTTGAAGTCATCACTGGCATCACAGGCCACAGGCTGCTCCCATCTGCACTTGGCCTTGCTCGAGGCAGGGAGTCGTTTCTACTTTCCCACTTCCCGTTCCCTTGGAATTGGGTCTTGCTTGCAGAGTTCTAACAAGTCTCTGCCTGGTGTATAGCATGTGGTCTGAAAGGGAAGGAAGTGAGGCCTCCATCGCTTAATTCAGCCAAGCCTTGCTCACTGCAGTCCAGGGGAAGAGGATGCCGGTTTTTGGCCCTTCTCAGTCTCCCTTGTAGGCCTGCTTGGTTTAAGAAATTCTTGAATTGGGGTTCAGTCGCTACTCTGGTAGCTACCTGGCTTTGGTAGCTTTTCAGAAGAAAGCCACCCCATTGGTCCCTAGTACACTTGAGCCTTGTTTTTTTTTTCTGCCCTTTTGAGACAGAGTTACATATATCCCAGGCTGGCTTCACACTTCCTGTATCGCCCAGGATGACCTTGAATTTCTGATCCTCCTGCTGGGATGATGAGATTACAAGAATGCTCAGCCACGCCCAATTTATGCCATGCTGGGCCCAAACCCAGCAATCACACGACTTGAGTTAAGCACTCCCCACTCCACATAACCACATATAAAAAGATTGTGAGGGCTCTCAAAATAAAAGCAACCCAAAAAGAAATTCTAAGGTGGGATTCCCCCACCTGCTTTGTATTGCAGAGTTGAATACACTTGGGTTTTGTGGCACCTTAGACATTCAAAACAAACCTTAACCCTAGGTTGGGGCACAACAGTAAATACCTGGTGTCTACAAGAAGAAAGCAAGATGTGTAGAATGGAGACAGTGTTCGTTGAGCACCTACAGTTCCACAGGATCAAAGACATACATAAACAGCACATCTGTCGTGTAACTGTCCAGGTTATCATTTACCTTCAGGAAACCAGTATTCTCACTGATGGCCAAATTTCCCTGTGCGTACTTAACTCCTGCTTACAACCAAGGCTTTTGTTGTTGCTGTGTTTTATAGACTTCTTTGTTTTAATGTATATGTATGAGTATTATACCTGCATGTATGTATGTACATCACATGTGTGCCTGGTTCCTACAGAGTTCAGAAAAGGGTGTTAGATCCCTTGGAACTGGAGTTAGACTAGGTTGTGCTAAGGTACCACATAAATGCTGGAACCCAACTGGGTCCTCTTAGAAGAGCAGCCAGTAGTCCACCACTGAGCCTTCTCTCCAGTCCCCATGGCAACTTTTTAAAGACAATGTTTCTTTCCCTTTTTGAGACAGAGCTTCAGTACTGTAGCCCAGGATACCCTAAGATTTGCTATATAGCCCAGGCTGGCCTTGAAGTTGGGTGTCTTTTTACTTCAGCCTCCCATTTATTAGGATTTAGGAGGCAGAAACAGGCAGATACTTGAGTTTGAGGTCAGCCTGGTCTACAGAATGAGTTCCAGGACATATGCTAATATGCTAAGCTACTAGCTCTTTCCATCCAGGACTTGGTGTGTGTCTTAATTTACTTAGATCTCTTTTCTGTCTCTAAGCAAAGCAAAGCTTTGTAGCGTTTCCCCATAAGACTTACACAATTCTTGCAAAAAGTGTGCAGGGTTTGTGCAGATGGGATGCTGTTGGTTCTTGTAGTTAACAGGTTGATCTTTGGAACACGAGAATCCTTTGCGTCTCCTGTTGCGTGCCATGTGTTGGTTCTGCTCTCTTGTCTGGGAACTACTTTGGGGTAAGACCTTTTCAGGAAGCGCTGTGGTCATGGAGATGCTGACCCAGCAGCCATAGTGAATGGGTTGGGAGTTAGTGCCGCCGCAGGCACCTGTCGTCAGTAGCAGACACCCATGTTTTCTGGGATGATACAGATTTGTGTGTGTGCTCTGTGGAAATTTGCAGCCTTGGTCTGTGCTATATTTTCAATTTGTCCAGAAGGATTTCAGTGATGCCTGGCCTGGCATGAGAAACCTTTCTAGTCGTTTCCCTAGGGAATGTTCTGACGACTGTAGGTCCCTAGACCTGTTTTCCTGGGTTTCTGACTTTTCAAATGGCCAGATTCTTTCTATTGAAAGGATCAGGCATAGAGTGTACAGGACTGGGCCTGATGTGCACTGGGCCAGGGTGTGGTGTGCACTGGGCCACTCTCACTACCTCTGTCCACCCTTGACCACATTCAGAGAAGTCATCCATCCACCACAGGCATTGGTGTGCAAACGTAGGCTCTGCTGACCGTGTGCTGAAGTCCAGGGCCACTAGAACCATCTTCCACACATCCCATGGAGTGGGTGCCATCTGCCTTTGGAGACTGGGGGAAGAGACCTGTTGTTTGCTTATCAACTTCCAACCTTCCTTCCTGGGACTCTGAGGGCACCCTCTGACTTCATTGCCGGAAGTAGTAGTCACAGAGTGAGATAAACCTAACAGGTATTCATTAGAATATATTTTATATTTAAAACAAAGCTTGGAAAAGGTATACTAAAGGGGCACAAATCCCTATTTTTTTTTTTATTTTAGTAAACAATCTATTGTCTTCTGCACAGATCTGAGGCACGGTTGTTTCTGTGCCACAGGATCTGTGTGCCACCTTTGTTTCTACAAGATTTGTCTGCATGGTGATGAGGATTCTTCTGGGGCTTCAGTCAAATTAGTGACACATCACTATAGCACTTGATCAAGACATCACTGTAGAAGAAAAGGAGGAACTACTCCTTCAGTCTTCACCTGGATATTCTGTTTAATTCACAGGCTTTGTTCTCAACACAAAACCAGTTGAGACCTTTACTCACTCTCAAGAAGGCTTTGATTCCTTGGTAAGACTTGGTTCTTCTGACCTCAAACTGTACAGCATATGTACACCCAGTGATCCTGAAGGGGTCTGTGCTACTTAAGCCTGTGCATGCACCATGTCATGGTGGGAGAGGAAGTACTGACAGCCTACCCTCTCCTTTTTGGATAGAAATCACAGGCACCTCCTAAGGAAAACAGCAGTGCTGAGTGAGGCCGGGCTCCCACAGGTGCCACAGCCTTAAGCCCGTCAGCTGGACGGGCACGGTGTGACCTCTGTGGTCCAGTGCTTGGGGAGAAACAGAAACCTTGGTGTCTTTTTATTTTTAGTAGTGAACTAGGCACATAGAAAGTTTAGTGTATGTGTGACCCAGTTAGAGAGGAAAGCAGATTATCGGAGGCTGCATGCAGGGAGCTCTGGAGGCCCTGTGGGTGAAGAAGAGGCAAAGGGAGAGCATTCAGGGGCTCTCAGGAGTGGGGACTGATTTTAAACACCTCTGAGGCAGGGACTCCTCTTCTCCAGCATCACCGCAGCACCCAGGAAAGAAGTCCTATAGTCCCCCAGCATATAGTCTCCAGCATCACCGCAGCACCAGGAAAGAAGTCCTATAGTCCCCCAGCATATAGTCTCCAGCATCACCGCAGCACCCAGGAAAGAAGTCCTATAGTCCCCCAGCATATAGTCTCCAGCATCACCGCAGCACCAGGAAAGAAGTCCTATAGTCCCCCAGCATATAGTCTCCAGCATCACCGCAGCACCAGGAAAGAAGTCCTATAGTCCCCCAGCATATAGTCTCCAGCATCACCGCAGCACCCAGGAAAGAAGTCCTATAGTCCCCCAGCATATAGTCTCCAGCATCACCGCAGCACCAGGAAAGAAGTACTATAGTCCCCCAGCATATAGTCTCCAGCATCACCGCAGCACCAGGAAAGAAGTACTATAGTCCCCCAGCATATAGTCTCCAGCATCACCGCAGCACCCAGGAAAGAAGTACTATAGTCCCCCAGCATATAGTCTCCAGCATCACCGCAGCACCAGGAAAGAAGTCCTATAGTCCCCCGAGAAACTCTTAACAGAGTAAAGGTGGCAGAATCAAGCATTTGCCACACGATGTCCACCCATGCTCACGTGTCTGCCCCTTGACCAGCAAGTGCTCAGCGTGTTGCAGGGCTCATATCTAAGCGTTAGGCCCTACAGAAGATAAAGGTATTAACAAGCACACCGGCGCTTGACAGCGTCTAAGTCCAGAAGCTCAGGGTGGCCACTGGAGTCTGCAGCTCCTGCCTGCAGTGTTGGTCTTCACACTGAAGTTGTTCCTGGGGAGACAGATCACTAATGTGAGGCACTGCTAGCTCTGGGATCCTTTCTCTCTCACTTGGCAATTTGATTTTCCACCCTTCATGCCTGAGGAGAGTTGAGCAAAACTAACAGCACCGCAGCACGTGAGCGTGGTTGACATTTAGGCCTTTAATCTGTTGGGTTTGTTTTGATGGGGGCCACTCCCAGGGCAGGTCCTGCCTACTCGTCCCAGGATCTGCAGCCCTGTCTTTTGTCTGCTGAATTCAGCTCTCATCAGAGCATGTGTTCTGCTCTCCTGACGGGTGTGCTACCATGCCAGTGCTGTCTTTAGTCACCGCAGCTTTCTAAGGGTTCAGTATCTGTGCAGCGAGCTCCTCCTCTATTTCCAAAATGCTGACCCGTCGCACACGCATAATCTTCTAGATAAAGTTTAGGCGAATTTTCCAACATTTAAAAAAAAATGTTGGGATTTTGTTTTTGTGGACTTGTATTAAATTTGAAGCAGGCTGGCATTCTTGCTTTGAGGGGTCATCGCCAGGAATGTGCAGAGCTTTTTCCATTCACTCAGATTTTCCTGTCCTTCAGCAATATTTGATAGTTTTCTTCCCGTCAGTCCTTCTCGGGTTGTTTTAATTTCACTCCTAACTGTGGTACCGTTCCTCTTGCTTTGGGAGGAGTTGTAAGCTCTCCAAGTGTGTGTGGCTGTGCATCGAGGTCTGAGTCCACCTTTTGCTTTGTGAAGGCCTTGGGGGAAGAGGGGAGTGTTTCAGAACGCCAGAGCCTGTAGCCTGCAAAGTGAGGCACATGCCTCCCTCCTGACCTGGATCCTGCTGCCACATGAGACACGTGACCTCAGAGGGACCTCGTTACCCTCACTAGCTTATAAAGGCTTTGTTCATATTTTCCTGCCACATAAGCAATATCTGTATTTCCACAGAAGGCTTAGCTTGTTTACTAGATGTTTCAAGGACACAAGATGTCAGTTCTGAGTATTTTATCGCTCACTCCTGCTGATGAGGACCCTGAGCTTGCTGCTGCTTGAGAAGACTGAAGCAGTGTTTGTCCAATACCGCTAGCCTTGTGTAAGGATACAATGTATCGGACCCTCTGCTCCCAGTATCACAGAGGCTCTCTAGGGTAGGCTCTGTCCCACGGTCTACAGATAAGGAAACTGAGGCGGTGGAGTTCCAGGTCACATGAGAACTAAGTGGCAGAGCAACCTCCACCCTGCCCCTCTGCTAAAGTTGAGTTATTTAGCAATTGAATGGTGAAAGTCATAATAGATAGATAGATAGATAGATAGATAGATAGATAGATAGATAGATAGATAGATAGCCTGGGTGCTTGCTGACCTAGAGGAGGGGGGCTGATAAGTGTAGAAGTGTGGCTTCTCCAGCTGCTATAGACCCAAGCCTTTTGTCTATACATGCTACAGCACTTCTCTGGAGGAGGGGCCTGCAGCTGGCCAAGTTTATTACCTAGGCTCAAGGCAGTGTGGTTCTGCTAGACACAGGCCGAAGGGGTAACAGGAGCCAGTGTTTAGCACCACCTTCTCCTGCTGGGACAGGAGCTAAAGGCCACTCTTGGTAACAGAATACTGTCTAATGTGACAGTAAGATGGCCCAGGGGGACAGTCACAGGTGTGACCACATCACAAGAGGACAGTATAATAGACTTGTTCCCTGTAGTCTTTTCGTAGTGAGCGGGCTGATAAGCATCTTTATATAATTAATTAATCTGTAAACTTTAGAGTGAGAGGTGCAAGTCAGTCAAGTAACCATATAGTCACCAGTTGTACTTTTTCATATTTTTTTCCTTTCTAGATTTTTAAGATGCATGCCTGTGTGTGGGTATGTGCATGTAAGTGCAGTTGTGTGTGGAGACTGGAGGCATCAGATTCTACTGGTTGTGAGTTGCCCAACATGGGTGCTGAGAACCAAATCCAGGTCTTCTGGAAGAGCAAGTGCCCTAACCACTGAGCTGCCTCCAGCCCTCACGGAGTGTCCTTGGTATTAATTTTCTAGTGTGTGACTTGGCCAGTTTCAGTTTCACTGTAGCACTTCCTACATGTGCACTGTAGCTTGCAGTACTCTGACAGCATGATTGGTACTCATAGCTTCTGCTCCTCCTGGCCCCGGTGCTATTGAGTCTTTAGTGTGGGCTCGTCCACAGGGCAGCTCTTGCCAGCCAGCATCCAGACCTTTCCATTTGCCACCTCAGGGTGGTGTGCTCCCTGAGCTCAGAGCTGGTGCCTACTGAGATCTGAGCCACACTTCCCAGTGCTGCCCCCGGAGGCCCTGCACTGTTCCCCGTGGCTGCAGGCACTCCGTCTAGCTGGCTGTGTGCTGGAATGGGCATTGTCCAAGGCCAGGCTCTGGGTAGAAGACAGAGATGGACTCTGTCTTCGAGAAGCTTAAAGTCCACTGGAACTGCTGACGCCATCATCCATTCGTTCAGTGAGTGAGTTAGCTGCAGCTCTGCTTCCCAAAGAGTTTGAAAGAAGGGTTGAGGGACAGACACAGTCTGCAGGGAAGCCCCAAAACGAGGCTTCCCTGTGCTCAGGAGGGCTTTAAGGAGGTGGTGGACCTACACAGGAACCCTCAGAGTTGCCTGTCTCTCGTATTCCATCCCAGGGTGAAGACCCAAGTCTAGGTTTCATTGAAGTATGAAGTCCAGGGTTGGGGTTTCTTATGCTTCTGGTCACGCTGTGACACAGGATCTGGTGTGGCTGTGGTCTCTCCTCATTGGCCTTTGTCCAGCACACTTAGGATGAGAGAGGGGTCCTCCCAGTCACACATTTGTTAGCATTGCCCAGGATAAACTTGTCCCATATGTGAAAAA

**Gm41235**

ACCAAGAGAGCTTGCAGTAGGAGAAAGCGGGAGCCGAGCCAGCCTGGAGAGCAAACAGATATTGCTGTGGATGTTTCTGTTTAGGGCCGAGGAAGACAATGATGCTGTTTTTCCTACTGGTAAGTATTTTGACAAGCATTTTATATGCAGAAGAGGAGGGAAGGAGGGAAAAGAATGGACTCAATAATAATCAGCTGGCTTGATTTCCAAGTACCAGTTAAACATTTGATGAATAGCAGATGAGGAACAGCAGCATAGATCTCAAGGCATCGGGCTGGCACTGCTTATGTGATTTACGACAGACCACTGGGCTCCCCTGACCTGATAGTGCTGAACACGATCTGAGAGCCAAAGAACAAGCCGCCCTCCCTGACCATGGAGGGAGAGATCATAAGGGGGACAATAGCTCCACTCAGATTCCTGTGAGTCATCCTTCATCTACAAGTCAAATGTCTTGCTGGCGTGCTTGACTTTATAGGGGCACTTGTCTATCTTTTTAGTAAGCTCCTGTGACTAATGTATAGATTAAAGCGGAGTATGGAGTCATTCGTTTTATAATAAGTGTCAGGAGCCCTGGCAAGTTCCCTGTGCACTGTTCTCTAAACTGCCAAACCAGTAAATGAGGCCATCCAACTTAAAAAACCTGCCAGGGGACTACAGGGTACATGAGAGCAAATAGATAATTACAGATGTGCCCCTGTGCTCCCATCACAGATGTGCTCCTGTCCCACAGAGGACACACACAAAACCAAGAGGTGGAGGTTCAGTGAACTTACCAGGCCTAACTCTCTTGCTAGGAGGAAGCAATGGGATCATGGGCATTGTCTGTCAAATACTGATGAAAGCCAACAGATAGCTAAATCAGGCAGCTCAGAGACCATAAGCAAAGCTAAGATGTCCTTTCTGCAGCAGAGCAGATAGCTAGCAAAAGACCAACTGATTGAGAAAGGAAGAATCAAGCCAGGAAGGCAGTTTAGGCAAGCGCCATCAAGTGCTACGATACCTTGAGCATCATGTTGCTCTGGAGGGTAACTGGCTCAGGAGTGGATGGCAGGAGTAGTTGCTGGCTGGGGGCCTTTAGTGGACGTGTGCCTTTATACACAGTCACTCCCTCCAGGTCTGTCCCTTTCACTTTTGTTTCAACCATGGAATTTCCACCATTTCAACACCTCCCCAATGACCTCACTTAAACTTCCTTTGTTGTGTTGTGTAGAGCTCCCTTATAGATGTAGGGCTGATGAGAGGAGGTGTTACAGCGGCCATAATCTGATGATTCCTCACAGACATAAGAAAAACTAAGTTATAATTACAGAGGGATGGCCTGTCCCAGCTAGGGGCCATAGGTGAGAAGAGTGCACACTCTCTTTACTGGGCATCCCACTCTTGAAGACTTATCACAAGGAAATAGCAAGACTATGACAGAGGAAGAAAAGCCCCCAGATCTGGAAAGGCAAGGAGTCACTCAGAGCAGCCTTCTCACAGACAGCCCATTGTCACCAACCAACCATGAAACAGCTTCTGGGAATGATGAAAAAAACAAAAAACAAAAACCCTGACTGGTTCTTATTGGACTTAGAGCCTGCTTACCAGAAGGAAATCCATGACTGGTACTGTGAACAAAGCCACCAATCACATCATAATTTCAGAGGCGATAACCCAGAGGTAACTAACGAAGTCACTAAATGTGCCATAACCTAAAGGTTATGAATCCTAGAGGAGAGCCTACCACTGCCAGTTCCCTAAACCTAGCTGCATTCTAAATACTTACTCACGCACAGATAAGTGTAGCTCTGCCCCTCATCAACGATGCTTCTTTTTATTGTAGATGGAGACGACTACTACAACTATCTATAACGAGTAAGTGAGCATGGGTGCCCAGACCTAACTGATATACCAACAATGAAACCCCTACACCTAAGGCTCAGAGACAGTTGAGAAAGTACGTGTACATATGTGTGCACACGTGTGTGTGTGTGTGTGTGTGTGTGTGTGTGTGTGTGATAACTAAAGAAGAGGCCATGAATTTGTGAGGAATGGTGGCCAGAGTTAAAAGAAGAGAAGGAGGAGGGGTGGAAATGTTGTAAATACATAAATAAATAAATTAAAATCTCAAAAAATTAATTAAAAATATCTAAATACACACCACACAGACACATGTACACACACACACACACACACACACACACACACACACACACACATGCCAAGTACACACATTTGGCTAATCAGAGACCACCTGGTTGAGTGTAATAGAGGAGAACTCTGGAAAATCATCCTTCACAGATTATTCCTTGGAGAAGAGAGAGAGAGAGAGAGAGAGAGAGAGAGAGAGAGAGAGAGAGAGAGAGAGAGAGAGAGAGAGAAAGAAGGAGGAGGAGGAAGAGGAGGAGGAGGAGGAGGAGGAGGAGGAGGAGGAGGAGGAGGAGGAGGACTTCAGAACCAGGCACCCAGGCAGGCAGGATTCTGACTCTCTGAAGTCTTCTGGTTCTGACCTGTGTCCTGGCCCTCACATGTTCCCCTTTTTTGCAGAAGAAGAGGCCTAAAGATGAAGTCCTGCTCTCAGCTGGATCCCTGTCTGACCAGGAGCTTGGCTCCCAGACTTTCTCCTGAGAAAGAAGAATGCCTTTAAGTTTTCAGAGAGGAGACAGACTAGTTAAGAGTCTTGCCACACTGGAGGCTTCTGTCCCGGGCAGAGAATGTGCTCAGAGCAGTCTACAGGCTCTTTCTGCAGCTGCAGGTTGGGGGACTCATCTTCCTTCAGTCCACAAAGCTTCATATTTTGAATTCAGAAGGTAATTCATTCATGCAACTACTTGGACTACCCAAGATTCATTGCTTCAGAGACTTGCAGAGATTTGCAACTCAAGCCTTCAAGACCTGCCAGAGACAGGAGGTATGCTAGGCTTGTTACCCCTGTCAATGATCACTCTGCAGAATTGTTCTGCCAGGGATTGCTATTGGCTAGCACTCCAAAGCCCCTAACAGCGTTCAAACTGTAATACTTACCAACAGTTTTTGTGTGTCCCCCTGAAGCCTGGTGAGATCACAGGGCTGTAACACTTGTAACTTATGGGCTAGGCACCCTGAGGCCTGTTGAGGACATGCAACTGTAACTCCCACAATCTTTCTTAATTTGTACCCTGAAGACTGCTGAGAGCATAGAGCTGCAGCCCTGACACTGAATCTTGGTTGGCATCCTGCCACTGGCAAGGATCTCAGAGGATGTGTTTGAGTCTTGACTGTCCTCTTTGAATTGTTTCAACGTGGCAAATATGGTTTTCTAAATTTCCCTTTGCTGTCTACTGACTTCATTATTGGAAGATGTGCAAGAACCTGATACCGGAGAATTAACTATCTCAACTGTTAACTGGCTGGGAATTCAACAAAAATTCAATGGTGGGAGCCTATGGGAAGTCAACAGGAATCACTGCAATCAATAGAACAGTTTTGGAATGCCGAGGGAGAGATGGACTTTGTCATGCTTCTGTTGTTCAGAGCATCCACGCTTTATGCTTCATTGTACCTCTTTGGAGCAAAATACCAGGACTCTGTAAGCAGTTTTAAAGTGAATGGCACAGCTGCCAGTTTCCGGTGTCAGATGTCAGTCTGATCAATTCCCCTGCTAGGAATATTGATCTTGTTTCTCTTGAGTTTCCTTTCATAGATATCTGCTACCCATCATGAAGGGCTATGGTAAGGTTCTGAAGGAACTGGAGTTTTCTGGTATGAGTGACACCAGGCTGTTACTAGGAGGAGTCCTGACCAAACAGGTGTTGGGCACAGACCCTTGCATTTTATTTTCTTCTGTCTTTCTTCCAAATGGCACATGGCCAAAATCTTTTGTCTTGTGGACCTCTTGGTTGCTGGATGTCACACCTGATGCTGAAGGCACTAGGGACATGGGGACATTACAAGCTGTGCACATACAAGGGGTTTCCCATTGAAAGCTGATGAGTCTGCTGGAAGAGAGATTTCCTGGTGAGCTTGGCACAGAGTTTTACAGCAGGTTTGGGCATCACACAATGATGATGTAAACTCATCCTGTTTTCTTGAGGGAGGTAAGAGGCCTTCCTCTCAGGGGTATCCAGGTTGGTCTCATTGTATTGTTCTGTGCAACCTGAGGCCATGGAGACAATGAAGGACAGCAGCTAGGAGGATACATGGTCTTGAATGCCTTCTTCAGGAATCAGAATAATTGATTATTCACCATGAAGATGGGTTAGTCTGGAACCCTAACAAGAGTGGGCAAGCTAAACATTCCCAAGGGGTTGTAATATTTCCCATTGCTCTTCTTCAGGTTCACTTTTCAAATATCTAAAGTGTTTTTGTGATTAACTTGGGTAAAATTTGTTGACTTGAATTTGTTATCTCTCTCAGTTGGTGATATTTTCCTGGAAACATGTTTCAGATGAGTGTAAAGGCTCTCCTGTGGGATTGTTGTATAGTAGTTAAGAGATGGTAGAGCCCATGTGTGCATTGGGGTGGGATCTCAGTGGTGTTTCCTCAGGGAGGCATCAACATTGGTCAATAGGCTTTTTGATAATTCTAGGAGAGGTTCTGTATGAGGGCATTCTTTCCTGGAAAGAAAGAACACAACGAGGAGTA
