# Supplementary material for: Comparative transcription profiling of mRNA and lncRNA in pulmonary arterial hypertension after C75 treatment
Source: BMC Pulm Med. 2023 Jan 31;23:46. doi: 10.1186/s12890-023-02334-6 (PMC9887911; doi:10.1186/s12890-023-02334-6)
Supplement: Supplementary file 2 — Additional file 2. Table S2. Lists of six lncRNAs and 1623 miRNAs (in the attachment of supporting information). [file 12890_2023_2334_MOESM2_ESM.pdf]

miRNA	lncRNA	Tot Sco	Tot Ene	Max Sc	Max En	Positions								
mmu-r	ENSMU	2261	-255	156	-26	8258	8525	8406	8458	8478	8166	8214	8242	8362
mmu-r	Gm388	2016	-264	162	-20.7	485	5528	5583	5639	5694	5749	5805	5860	5915
mmu-r	Gm388	2000	-255	159	-24.6	7645	3399	486	5475	5526	5581	5637	5692	5747
mmu-r	ENSMU	1894	-226	184	-30.2	1820	8515	10141	10168	6012	7451	199	8497	8
mmu-r	ENSMU	1844	-82.1	149	-13.5	9203	200	11322	12858	876	5161	5173	10162	10176
mmu-r	Gm388	1812	-258	157	-23.2	3952	5519	5574	5630	5685	5740	5796	5851	5906
mmu-r	Gm388	1810	-204	153	-22.8	5530	5585	5641	5696	5751	5807	5862	5917	5973
mmu-r	Gm388	1730	-255	160	-23.5	5484	5540	5595	5651	5706	5761	5817	5872	5927
mmu-r	Gm388	1721	-299	159	-30.9	5515	5570	5626	5681	5736	5792	5846	5901	5957
mmu-r	ENSMU	1692	-144	167	-18.3	12892	13689	2551	10714	9431	12205	1817	4993	10736
mmu-r	Gm388	1686	-296	157	-28.9	5515	5570	5626	5681	5736	5792	5847	5902	5958
mmu-r	Gm388	1641	-244	153	-23.6	5846	5901	5957	5511	5566	5622	5677	5732	5788
mmu-r	Gm388	1637	-230	152	-22.6	5512	5567	5623	5678	5733	5789	2860	186	5844
mmu-r	Gm388	1627	-139	152	-16.4	5553	5664	5719	5830	5885	5996	5340	5497	5608
mmu-r	Gm388	1625	-191	149	-17.7	5527	5582	5638	5693	5748	5804	5859	5914	5970
mmu-r	Gm388	1592	-155	154	-18.8	6790	5549	5660	5715	5826	5881	5992	5496	5607
mmu-r	ENSMU	1571	-246	171	-34.8	8505	8532	8470	8449	8405	8299	8559	8158	6550
mmu-r	ENSMU	1554	-142	171	-17.6	9245	3548	7231	3571	3585	3601	3617	9303	5975
mmu-r	Gm388	1545	-171	145	-20.5	151	5481	5537	5592	5648	5703	5758	5814	5869
mmu-r	Gm388	1533	-233	156	-25.6	5480	5536	5591	5647	5702	5757	5813	5868	5923
mmu-r	ENSMU	1519	-294	158	-30.5	4115	7999	9275	9283	9291	9299	9307	9315	9323
mmu-r	ENSMU	1490	-137	156	-16.3	7906	7325	7745	7824	7491	8320	7242	7661	7576
mmu-r	ENSMU	1489	-145	155	-19	7324	7744	7823	7905	7241	7660	8322	7493	7577
mmu-r	ENSMU	1482	-109	155	-15.4	7325	7745	7824	7241	7661	7908	8322	8154	7493
mmu-r	ENSMU	1466	-167	154	-19.8	10234	5364	5764	7350	9268	4074	9260	824	1936
mmu-r	ENSMU	1464	-211	156	-21.8	4111	7995	9272	9280	9288	9296	9304	9312	9320
mmu-r	Gm388	1434	-187	184	-29.7	1900	1916	1932	4046	8124	8098	3677	6950	729
mmu-r	ENSMU	1424	-116	149	-13.8	7944	7813	7229	7314	7650	7734	7981	8059	8143
mmu-r	ENSMU	1418	-98.6	146	-15.4	7325	7745	7824	7243	7495	7579	7663	7910	8156
mmu-r	ENSMU	1417	-128	183	-32.2	4427	2877	4393	3645	9975	13395	9443	9807	4988
mmu-r	ENSMU	1385	-168	167	-22.1	10229	4072	9256	1932	811	822	7347	5360	5764
mmu-r	ENSMU	1384	-190	163	-26.2	8308	8249	8324	8424	4639	8227	3059	5596	6996
mmu-r	Gm388	1383	-195	154	-21.9	5516	5571	5627	5682	5737	5793	5847	5902	5958
mmu-r	ENSMU	1382	-125	174	-24.5	4429	3647	4393	9807	2877	13395	9974	9443	4988
mmu-r	ENSMU	1363	-161	166	-26.3	5763	9257	822	10226	1930	4074	7344	812	5362
mmu-r	ENSMU	1324	-113	154	-17	1791	939	2000	2568	9250	9283	9299	972	6360
mmu-r	ENSMU	1322	-128	155	-17.9	7019	5258	1726	4889	9003	316	5947	6118	6405
mmu-r	ENSMU	1317	-160	160	-22.1	315	4889	5946	6116	6402	1725	5256	7017	9001
mmu-r	ENSMU	1311	-125	156	-16.8	5608	1004	7702	7786	8028	7534	7619	7451	8112
mmu-r	ENSMU	1297	-198	163	-31.8	7991	4108	9269	9277	9285	9293	9301	9309	9317
mmu-r	Gm388	1281	-194	143	-23.2	5518	5573	5629	5684	5739	5795	5846	5901	5957
mmu-r	ENSMU	1281	-107	145	-14.6	3047	1627	10237	10753	10761	10769	10777	10785	3910
mmu-r	ENSMU	1262	-152	163	-21.1	7648	7310	8139	8307	7978	7227	8056	1535	
mmu-r	ENSMU	1251	-140	168	-19.5	517	3473	7126	11939	3463	7143	7159	11955	
mmu-r	ENSMU	1246	-219	190	-39.6	1453	1469	1485	1505	14183	7432	14144		
mmu-r	ENSMU	1239	-106	165	-16	567	3460	516	7138	7154	11942	11958	4172	
mmu-r	Gm388	1228	-131	165	-20.6	391	291	8097	8595	1301	730	4747	2022	
mmu-r	Gm388	1217	-94.4	154	-11.9	5557	5668	5723	6000	5502	5613	5779	4351	
mmu-r	ENSMU	1216	-105	164	-20	5591	603	4736	810	5367	9251	4081	12091	
mmu-r	ENSMU	1203	-167	158	-25.1	11873	370	11221	1669	2045	6132	12173	10975	
mmu-r	ENSMU	1201	-136	159	-22.6	7447	8496	8630	1274	9421	2620	1805	5875	
mmu-r	ENSMU	1196	-98.2	158	-18.9	7431	1454	1470	1486	1504	14180	14140	7566	
mmu-r	ENSMU	1175	-213	180	-35.3	8228	8312	8328	8412	8248	8459	3057		
mmu-r	ENSMU	1171	-136	149	-19.8	8348	7433	7601	8010	7265	7349	7848	8094	
mmu-r	ENSMU	1161	-151	158	-22.8	4768	9810	11272	178	11027	694	2125	11926	
mmu-r	ENSMU	1151	-135	145	-17.9	1534	7312	7648	7979	8141	8309	8057	7228	
mmu-r	ENSMU	1129	-142	166	-23.7	10749	3048	165	10769	10785	4138	10734		
mmu-r	ENSMU	1116	-218	190	-39.6	7139	7155	11941	11957	3473	519			
mmu-r	ENSMU	1088	-138	160	-21.5	7427	1454	1470	1486	1504	14180	14140		
mmu-r	ENSMU	1074	-168	160	-28.5	11271	7200	2123	10426	11027	177	11924		
mmu-r	ENSMU	1072	-130	165	-26.2	8000	11823	12654	52	5075	2109	12620		
mmu-r	ENSMU	1066	-133	168	-29	8324	8308	8248	4638	9134	5595	6995		
mmu-r	ENSMU	1066	-115	166	-21.3	7430	1451	1467	1483	1503	14181	14141		

mmu-r	ENSMU	1062	-95.1	160	-18	8132	7303	7555	8048	7723	8300	7803
mmu-r	ENSMU	1056	-152	159	-24.8	566	1402	4000	4921	8413	414	1296
mmu-r	ENSMU	1055	-92.4	161	-14.9	7428	889	8510	1835	2626	9954	3273
mmu-r	ENSMU	1054	-157	190	-33.6	12829	4443	4459	12845	4419	189	
mmu-r	ENSMU	1054	-151	157	-28.8	4137	163	3044	10730	10755	10770	10786
mmu-r	ENSMU	1032	-90.1	156	-15.2	8148	7350	7849	8011	8095	4806	267
mmu-r	ENSMU	1032	-90.1	156	-15.2	8148	7350	7849	8011	8095	4806	267
rco-mil	ENSMU	995	-103	147	-14.8	7801	7947	8129	7302	7554	7722	8299
rco-mil	ENSMU	992	-90.1	146	-14.2	7948	7801	8129	7302	7554	7722	8299
mmu-r	ENSMU	962	-170	164	-30.6	7994	9278	9294	9310	9258	4111	
mmu-r	ENSMU	956	-126	167	-21.9	3460	516	7138	7154	11942	11958	
mmu-r	ENSMU	956	-111	200	-37.3	9417	3660	8490	7156	1035	2564	
mmu-r	Gm412	952	-153	163	-28.6	2374	2392	2410	2359	2048	3737	
mmu-r	ENSMU	952	-28.8	168	-5.77	2940	2094	1918	2893	2846	4053	
mmu-r	ENSMU	946	-143	168	-29.2	9276	9292	9308	9324	7996	4110	
mmu-r	ENSMU	946	-95.5	177	-25.6	8519	10154	8500	10059	523	8633	
mmu-r	ENSMU	942	-64.4	184	-24.9	10174	6486	9922	5964	3537	5872	
mmu-r	ENSMU	942	-64.4	184	-24.9	10174	6486	9922	5964	3537	5872	
mmu-r	ENSMU	937	-109	161	-20.1	7355	7854	8100	4307	7271	7439	
mmu-r	ENSMU	933	-63	180	-22.9	10175	3538	5965	6485	5874	9925	
mmu-r	ENSMU	933	-63	180	-22.9	10175	3538	5965	6485	5874	9925	
mmu-r	ENSMU	933	-63	180	-22.9	10175	3538	5965	6485	5874	9925	
mmu-r	ENSMU	932	-60	179	-23.4	10176	6488	3539	5966	5874	9925	
mmu-r	ENSMU	932	-36.3	172	-10	3542	6772	5683	5363	4677	3787	
mmu-r	ENSMU	929	-125	171	-37.9	4084	634	2464	2502	2540	4125	
mmu-r	ENSMU	927	-147	160	-30	7975	9329	7104	8288	6214	11298	
mmu-r	ENSMU	925	-157	168	-37.6	6881	11537	12790	13205	4674	10309	
mmu-r	ENSMU	920	-149	169	-31.9	3566	6668	9576	5778	7397	5840	
mmu-r	ENSMU	916	-37.6	167	-10.6	12414	5221	10436	6517	11664	3637	
mmu-r	ENSMU	915	-61.6	179	-25	10176	6488	5966	3539	5874	9926	
mmu-r	ENSMU	914	-118	162	-24.6	636	2414	7008	6144	11481	3344	
mmu-r	ENSMU	911	-57	171	-19.9	10176	6488	5966	3539	5874	9925	
mmu-r	ENSMU	910	-136	170	-29.8	11298	9328	7974	7108	8292	6215	
mmu-r	ENSMU	909	-133	168	-32	11171	1017	996	4574	755	4540	
mmu-r	ENSMU	909	-118	159	-22.8	9271	9287	9303	9319	7996	4110	
mmu-r	ENSMU	907	-145	162	-32.4	4276	2505	7519	9569	9322	154	
mmu-r	ENSMU	906	-142	156	-27.5	9569	156	4277	9322	2503	7517	
mmu-r	ENSMU	904	-102	162	-23.6	2415	11631	10691	12659	358	4649	
mmu-r	ENSMU	902	-82.1	162	-18.9	3419	9842	178	3371	4057	7054	
mmu-r	ENSMU	898	-98.5	159	-21	573	1969	5356	4540	1665	1949	
mmu-r	ENSMU	890	-128	166	-31.3	84	10208	9810	1774	7036	8962	
mmu-r	ENSMU	890	-93.4	156	-21	13108	11470	7016	13310	6642	3036	
mmu-r	Gm388	888	-142	148	-23.7	5550	5661	5716	5827	5882	5993	
mmu-r	ENSMU	888	-127	157	-25.7	2898	171	2118	11925	11028	11272	
mmu-r	Gm388	887	-104	154	-23.3	2174	1422	8640	2608	3455	6281	
mmu-r	ENSMU	884	-60.1	150	-11.5	12051	13853	881	9447	9913	5168	
mmu-r	ENSMU	883	-64.5	154	-13.8	8927	5208	5499	9337	3617	11951	
mmu-r	ENSMU	881	-135	154	-26.5	8105	4059	12610	8749	143	5701	
mmu-r	ENSMU	880	-82.1	152	-18.9	2203	1555	1929	2081	2826	2920	
mmu-r	ENSMU	878	-127	147	-21.4	7356	7855	8101	7272	7440	59	
mmu-r	ENSMU	875	-92	156	-19.8	2625	8	8495	7452	1808	8508	
mmu-r	ENSMU	874	-77.6	188	-20.2	13858	5169	9917	9448	4824		
mmu-r	ENSMU	868	-86	151	-18	7532	8021	2389	7448	8109	7612	
mmu-r	ENSMU	868	-53.4	153	-11.5	4857	3644	9973	2003	31	6726	
mmu-r	ENSMU	867	-98.4	150	-22.1	11289	12172	11218	11872	368	10974	
mmu-r	ENSMU	860	-116	148	-22.4	2123	11268	175	11026	11923	2898	
mmu-r	ENSMU	854	-126	149	-21.1	7614	7362	7446	7861	8023	8107	
mmu-r	ENSMU	840	-139	174	-32.7	4095	4128	4111	637	3750		
mmu-r	ENSMU	826	-134	177	-37.4	995	5677	6123	11172	5633		
mmu-r	ENSMU	820	-160	169	-35.2	4107	9268	9289	9305	9321		
mmu-r	ENSMU	811	-169	171	-37.3	9278	9294	9310	7993	4109		
mmu-r	ENSMU	810	-113	173	-28.4	4090	4108	632	4125	3745		
mmu-r	ENSMU	803	-100	184	-28.3	14182	7431	6341	3031	7188		
mmu-r	ENSMU	801	-113	171	-29.7	4283	7436	9161	7237	9839		

mmu-r	ENSMU	798	-79.5	168	-20.8	4090	4127	4108	632	3751
mmu-r	ENSMU	795	-131	167	-32.2	1269	4890	389	1441	8925
mmu-r	Gm412	795	-81.7	176	-23	2145	2197	2161	2177	722
mmu-r	ENSMU	792	-94.9	176	-24.7	3463	3475	566	802	7725
mmu-r	ENSMU	792	-90.4	176	-23.2	3463	3475	566	802	7725
mmu-r	ENSMU	791	-118	164	-28	443	8436	4944	1496	2039
mmu-r	Gm388	789	-108	167	-26.7	4367	2214	5133	4914	7768
mmu-r	ENSMU	786	-89.7	165	-22.7	7817	7318	7738	2874	1846
mmu-r	ENSMU	784	-89.5	171	-23.5	3464	3476	567	803	7729
mmu-r	ENSMU	783	-103	163	-26.6	9330	1560	7485	10906	11299
mmu-r	ENSMU	781	-132	164	-31	1	11662	12383	11999	9572
mmu-r	ENSMU	781	-110	163	-27.5	6491	7177	831	7100	4050
mmu-r	ENSMU	780	-113	174	-33.2	8498	8274	8686	10929	6465
mmu-r	Gm388	779	-131	164	-29	462	7289	537	7082	2145
mmu-r	ENSMU	779	-110	171	-37.2	4085	635	4126	3745	4110
mmu-r	ENSMU	778	-61.3	162	-13.7	7549	8126	8294	7298	7716
mmu-r	ENSMU	777	-113	164	-25.1	11319	11242	3335	12753	7439
mmu-r	ENSMU	776	-109	167	-26.7	1484	10379	927	4508	282
mmu-r	ENSMU	776	-79.7	161	-20	7431	14184	6348	7195	3032
mmu-r	ENSMU	776	-79.7	161	-20	7430	14183	6347	7194	3031
mmu-r	ENSMU	776	-77	161	-18.6	7430	14183	6347	7194	3031
mmu-r	Gm388	775	-97.7	176	-27.1	8330	4857	3464	8307	4172
mmu-r	ENSMU	773	-112	180	-37.5	713	6297	1938	3335	946
mmu-r	ENSMU	771	-139	158	-29	4109	7996	9281	9297	9313
mmu-r	ENSMU	769	-110	159	-26.7	12534	12586	12321	6752	4554
mmu-r	ENSMU	769	-102	158	-22.8	3185	14201	4672	10027	6883
mmu-r	Gm388	768	-95.2	162	-28.1	6914	2865	1085	6983	6772
mmu-r	ENSMU	768	-67.4	159	-16.6	10688	9781	12060	9548	12114
mmu-r	ENSMU	767	-111	177	-35.7	10064	12477	1632	11584	9853
mmu-r	ENSMU	767	-87.3	157	-20.2	707	941	6291	9594	1936
mmu-r	ENSMU	767	-83.6	160	-20.7	8592	2571	4024	7586	4600
mmu-r	ENSMU	767	-80.7	158	-21.3	387	2920	6094	6214	1561
mmu-r	ENSMU	766	-142	171	-34.6	978	5472	1014	5707	963
mmu-r	ENSMU	765	-117	163	-29.4	12411	6307	7067	4250	526
mmu-r	Gm388	765	-116	157	-26	737	6071	6347	4330	7480
mmu-r	ENSMU	764	-75.1	186	-29.3	4466	1449	1727	5171	4721
mmu-r	ENSMU	764	-74.4	165	-23	14319	6171	11521	11436	7118
mmu-l	ENSMU	763	-99	162	-24.2	3284	7788	4019	3363	4381
mmu-r	ENSMU	763	-69.2	162	-17.8	9911	13848	5169	9445	4824
mmu-r	Gm388	761	-130	158	-31.6	6481	8881	200	2625	6458
mmu-r	Gm388	761	-107	160	-26.2	5112	444	790	6546	8991
mmu-r	ENSMU	761	-59.4	159	-14.3	5056	2557	7727	8987	6845
mmu-r	ENSMU	760	-80.6	161	-20.3	10211	10747	154	4119	6977
mmu-r	ENSMU	760	-63.4	156	-14.9	2102	13041	819	4995	1604
mmu-r	Gm388	759	-109	164	-28.8	447	6547	8991	791	5110
mmu-r	ENSMU	759	-59.9	163	-15.2	9931	4552	8796	12784	6627
mmu-r	ENSMU	758	-123	158	-27	6295	3331	3356	9839	1938
mmu-r	ENSMU	758	-111	162	-28.9	7950	9803	7397	6664	9085
mmu-r	ENSMU	757	-103	168	-24.7	524	4248	11923	7650	2119
mmu-r	ENSMU	757	-99	160	-21.3	1054	3606	5629	8752	6199
mmu-r	Gm388	757	-95.8	159	-24.3	4302	295	4280	8325	8555
mmu-r	Gm388	756	-123	164	-31.9	6460	6483	8884	198	2624
mmu-l	ENSMU	756	-97.1	161	-26.1	7791	3288	3366	4026	4384
mmu-r	ENSMU	755	-88	167	-22.4	8001	5112	11824	2110	52
mmu-r	ENSMU	755	-86.7	158	-19.6	10595	2190	453	3737	14079
mmu-r	Gm388	754	-91.5	159	-23.6	4303	4281	296	8326	8556
mmu-r	ENSMU	753	-113	155	-26.7	4883	8375	391	382	964
mmu-r	ENSMU	753	-59.3	155	-15.2	7572	7362	7561	13639	5145
mmu-r	ENSMU	752	-93.1	155	-22.1	1467	4913	8405	412	3869
mmu-r	ENSMU	751	-94.6	156	-22.3	285	4508	928	10377	1483
mmu-r	Gm388	750	-96.6	152	-24.7	6833	206	504	4881	3096
mmu-r	ENSMU	750	-83.1	163	-18	151	4118	431	6975	849
mmu-r	ENSMU	750	-72.6	156	-18	10687	12114	9781	9548	12060
mmu-r	Gm412	749	-88.3	158	-22.9	1906	858	2210	2822	2621

mmu-r	Gm388	749	-87	154	-21.1	6984	6771	1086	6918	2870
mmu-r	ENSMU	747	-103	155	-23.4	5855	8092	8668	3348	7317
mmu-r	ENSMU	747	-77.6	155	-16.7	274	7711	1965	3942	4267
mmu-r	ENSMU	746	-94.4	162	-22.2	10234	9632	10553	9038	13511
mmu-r	ENSMU	745	-108	152	-24.4	3900	11541	10011	10404	10937
mmu-r	Gm388	745	-94.1	153	-24	258	8780	1368	6490	6223
mmu-r	Gm388	745	-92.1	158	-24.3	4303	295	4280	8327	8555
mmu-r	ENSMU	745	-91.8	159	-23.8	10553	10233	13511	9633	9038
mmu-r	ENSMU	745	-83.9	159	-20.5	431	4117	850	152	6977
mmu-r	ENSMU	744	-106	153	-22.2	1494	8434	440	4941	2022
mmu-r	Gm388	744	-91.8	162	-25.2	536	8733	7487	4393	7362
mmu-r	Gm388	743	-108	158	-29.3	6975	3383	738	4327	6070
mmu-r	Gm388	743	-90.1	151	-20.8	7262	7812	2683	7083	454
mmu-r	Gm388	743	-81.8	166	-23.5	293	4302	4381	240	3773
mmu-r	ENSMU	742	-100	158	-23.1	1904	12242	2149	10956	2177
mmu-r	Gm388	742	-98.7	162	-24.7	456	7081	2679	7815	7264
mmu-r	ENSMU	742	-93.8	157	-23.3	539	4616	1418	2975	5763
mmu-r	ENSMU	742	-93.3	159	-24.2	14377	13538	4238	229	254
mmu-r	Gm388	741	-98	154	-22.4	1365	6231	258	6496	8775
mmu-r	ENSMU	741	-83.5	157	-19.3	3333	711	944	1936	6294
mmu-r	ENSMU	741	-75.1	151	-20.1	7329	12518	3908	5567	7012
mmu-l	ENSMU	740	-86.5	163	-22.3	7788	4022	3285	3363	4381
mmu-r	ENSMU	740	-78.4	156	-23.8	2918	1559	6211	388	6093
mmu-r	ENSMU	740	-59.1	159	-14.9	5538	1769	14	5252	4622
mmu-r	ENSMU	740	-52	154	-13	9914	9448	13854	5169	4825
mmu-r	Gm388	739	-104	154	-24.1	449	5117	6548	791	8995
mmu-r	ENSMU	739	-91.2	154	-21.1	13955	3350	4381	6894	7317
mmu-r	ENSMU	739	-89.5	156	-21.8	6748	1253	1519	8899	2918
mmu-r	ENSMU	739	-72.5	153	-18.3	3284	4023	7788	3363	4381
mmu-r	ENSMU	739	-66.4	152	-17.6	4861	5010	5658	5553	1297
mmu-r	ENSMU	738	-98.6	151	-20.9	444	4945	1497	8437	2041
mmu-r	ENSMU	738	-93.2	156	-19.8	943	6294	3330	708	1937
mmu-l	ENSMU	738	-72	154	-18.7	3284	4017	4383	7784	3363
mmu-r	ENSMU	737	-86.8	163	-25.3	7410	1640	4902	1855	7254
mmu-r	ENSMU	737	-85.6	157	-21.5	48	2103	11821	5106	7998
mmu-r	ENSMU	735	-93.8	164	-24	4606	3734	5019	8661	4838
mmu-l	ENSMU	735	-86.1	158	-22.7	3284	4019	4384	7788	3363
mmu-l	ENSMU	735	-81.5	154	-20.3	3284	4019	7788	4383	3363
mmu-r	ENSMU	735	-61	164	-20.3	3369	3238	5513	11	5231
mmu-r	Gm388	733	-113	154	-25.1	8726	258	6224	7711	6492
mmu-r	ENSMU	733	-83.3	155	-24.3	4715	8949	9474	8562	3689
mmu-r	Gm388	732	-83.6	154	-19.3	4702	4994	1843	4795	8203
mmu-l	ENSMU	732	-81.8	155	-18.7	7788	3284	4022	3363	4381
mmu-l	ENSMU	732	-72.6	158	-20.5	3284	4023	3362	7788	4383
mmu-r	ENSMU	732	-56.9	150	-13.9	2561	3725	9413	2442	519
mmu-r	ENSMU	731	-80.5	151	-18.1	4238	13533	14375	14295	252
mmu-r	ENSMU	731	-61.8	158	-15.8	5208	9120	12218	8730	11594
mmu-r	ENSMU	730	-87.6	155	-20.6	11523	6460	2982	2208	2236
mmu-r	ENSMU	730	-68.2	156	-17.1	13479	8685	13801	450	7514
mmu-r	ENSMU	729	-85.2	150	-18.6	10537	9558	12280	12672	10131
mmu-r	ENSMU	728	-67.1	153	-14.8	182	6997	8838	7754	2843
mmu-r	ENSMU	728	-47.8	153	-14.3	8127	8297	7550	7300	7721
mmu-r	ENSMU	726	-90.4	149	-22.8	4745	8443	9478	450	5284
mmu-l	ENSMU	724	-74.1	151	-18.8	7791	3284	4018	4383	3363
mmu-r	ENSMU	724	-57.5	150	-13.3	3253	3500	9728	9671	1422
mmu-r	ENSMU	723	-80.5	148	-19.5	4884	8376	383	965	1436
rco-mi	ENSMU	722	-67.6	151	-15.3	294	2934	3006	10270	197
rco-mi	ENSMU	722	-67.6	151	-15.3	294	2934	3006	10270	197
rco-mi	ENSMU	722	-67.6	151	-15.3	294	2934	3006	10270	197
mmu-r	ENSMU	722	-66.5	149	-15.5	4026	403	8396	4842	4904
mmu-r	ENSMU	722	-56.2	152	-14.5	5997	3936	5024	10409	13701
mmu-r	Gm388	718	-78.4	150	-17.9	8996	5116	6548	451	795
mmu-r	ENSMU	717	-92.7	146	-19.4	824	7349	1934	814	5362
mmu-r	ENSMU	715	-94.2	146	-23.4	12246	2356	8274	8496	11758

mmu-r	ENSMU	713	-63.1	147	-15.3	4028	3290	4386	3368	7793
mmu-r	ENSMU	712	-78.6	145	-23.2	2169	6215	9266	7522	3564
mmu-r	ENSMU	708	-68.3	143	-14.8	8102	4877	8369	1258	7091
mmu-r	ENSMU	707	-61	147	-13	8105	1259	4880	7094	8372
mmu-r	ENSMU	700	-80.9	184	-25.3	1756	1772	1788	3066	
mmu-r	ENSMU	695	-83	180	-22.1	1756	1772	1788	3064	
mmu-r	ENSMU	686	-69.6	180	-20.1	1757	1773	1789	3063	
mmu-r	ENSMU	686	-69.6	180	-20.1	1757	1773	1789	3063	
mmu-r	ENSMU	676	-68.6	177	-20.9	1754	1770	1786	3064	
mmu-r	ENSMU	676	-68.6	177	-20.9	1754	1770	1786	3064	
mmu-r	ENSMU	676	-68.6	177	-20.9	1754	1770	1786	3064	
mmu-r	ENSMU	671	-67.7	175	-19.8	1759	1775	1791	3065	
mmu-r	ENSMU	671	-63.9	175	-19	1759	1775	1791	3065	
mmu-r	ENSMU	653	-92.8	173	-30.3	10237	10796	1630	6817	
mmu-r	ENSMU	649	-99.3	179	-29.4	14110	8009	7841	8259	
mmu-r	ENSMU	648	-109	183	-34.9	12383	1	12348	49	
mmu-r	ENSMU	646	-133	175	-41.6	4222	900	985	11166	
mmu-r	Gm412	645	-95.6	181	-33.1	2145	2161	2177	722	
mmu-r	Gm388	644	-111	161	-27.8	5490	5601	5767	5933	
mmu-r	ENSMU	643	-124	178	-40.6	177	3426	3367	4384	
mmu-r	ENSMU	643	-65.9	180	-21.5	3380	5573	5860	4486	
mmu-r	ENSMU	642	-103	179	-35.4	1147	1086	1173	1051	
mmu-r	ENSMU	641	-64	167	-19	1759	1775	1791	3065	
mmu-r	ENSMU	638	-55.9	170	-17.7	1359	4995	9135	1382	
mmu-r	ENSMU	637	-87.6	175	-29.1	3716	9473	8740	5579	
mmu-r	ENSMU	635	-91.2	168	-29.6	5754	9932	87	3314	
mmu-r	ENSMU	635	-43.9	173	-16.9	5236	3759	5114	5472	
mmu-r	ENSMU	634	-115	167	-33	8439	8327	8222	8463	
mmu-r	Gm388	633	-106	171	-35.4	96	8733	8524	78	
mmu-r	Gm388	629	-98.7	176	-32.6	2457	2292	1477	3296	
mmu-r	ENSMU	629	-90.8	180	-37.5	6970	1333	9127	7589	
mmu-r	ENSMU	629	-73.9	162	-25.1	3908	9235	1377	5846	
mmu-r	ENSMU	629	-73.7	162	-21.3	10796	10236	1630	6816	
mmu-r	ENSMU	629	-71.3	170	-19.2	2745	7460	12628	9489	
mmu-r	ENSMU	628	-88.1	173	-26.1	12353	54	9556	2555	
mmu-r	ENSMU	627	-67.4	159	-16.9	434	2467	2505	2543	
mmu-r	Gm412	626	-76.6	177	-26.7	2148	2164	2180	725	
mmu-r	ENSMU	624	-71.1	169	-22.6	8039	6238	5763	5928	
mmu-r	ENSMU	624	-66.2	171	-20.7	9229	12785	7454	8796	
mmu-r	ENSMU	623	-101	163	-30.5	4201	1326	1934	3388	
mmu-r	ENSMU	623	-72.7	171	-25.5	7486	10909	9332	11303	
mmu-r	Gm388	623	-69.9	167	-22.4	5270	3528	6582	6502	
mmu-r	ENSMU	622	-101	165	-26.8	3368	3427	180	4382	
mmu-r	ENSMU	622	-98.1	168	-28.9	373	5	3146	3921	
mmu-r	ENSMU	622	-72.6	165	-21.5	296	193	10030	2724	
mmu-r	Gm388	621	-92.8	162	-24.3	6955	1545	8979	8567	
mmu-r	ENSMU	621	-89	171	-32.2	4134	10795	1790	9233	
mmu-r	Gm388	620	-68.8	159	-23.9	2060	3103	1806	4937	
mmu-r	Gm388	619	-84.3	160	-26.2	1009	2068	8441	3376	
mmu-r	ENSMU	619	-81.6	156	-23.5	3909	1376	7146	9230	
mmu-r	ENSMU	619	-63.6	164	-19.4	9226	7453	12784	8793	
mmu-r	ENSMU	619	-42.7	159	-13.6	2851	2792	7146	5717	
mmu-r	Gm388	617	-88.3	159	-24.3	8738	101	83	8531	
mmu-r	ENSMU	617	-79.7	170	-25.7	4114	2219	999	6503	
mmu-r	ENSMU	617	-62.4	161	-17.8	2830	2974	3105	2918	
mmu-r	ENSMU	617	-55.6	163	-16.1	7774	4816	4538	140	
mmu-r	ENSMU	616	-83.5	162	-25.6	4292	7488	2990	576	
mmu-r	ENSMU	616	-83.3	166	-27.9	177	3428	3369	4385	
mmu-r	ENSMU	616	-76.5	158	-21.2	1275	1448	1933	7679	
mmu-r	ENSMU	615	-92.7	156	-26	5855	6003	8289	8672	
mmu-r	Gm388	615	-92.2	162	-26.8	8735	81	96	8529	
mmu-r	ENSMU	615	-78.2	184	-29.4	4316	9039	8585	5597	
mmu-r	ENSMU	615	-41.6	165	-13.7	955	2681	4570	9737	
mmu-r	ENSMU	613	-103	160	-28.7	2170	1329	777	1872	

mmu-r	ENSMU	613	-82.6	156	-24.1	1331	9123	7586	6968
mmu-r	ENSMU	612	-72.4	160	-22.9	8587	1645	13935	5612
mmu-r	ENSMU	612	-71.8	164	-21.2	545	2338	7126	4172
mmu-r	ENSMU	611	-116	166	-32.9	183	112	609	592
mmu-r	ENSMU	611	-91.8	169	-28.1	5717	10216	4321	1291
mmu-r	ENSMU	611	-83.8	167	-27.7	6125	11171	5633	10754
mmu-r	Gm388	611	-83.2	172	-28.3	7809	3853	449	6549
mmu-r	ENSMU	611	-77.9	160	-21.6	1328	7582	9125	6962
mmu-r	ENSMU	611	-66.1	156	-17.7	2286	12644	7845	4573
mmu-r	Gm388	611	-60.8	166	-18.9	171	7688	4831	8311
mmu-r	ENSMU	611	-48.6	159	-16.4	2878	13396	4390	4989
mmu-r	ENSMU	611	-48.6	159	-16.4	2878	13396	4390	4989
mmu-r	ENSMU	611	-48.6	159	-16.4	2878	13396	4390	4989
mmu-r	ENSMU	610	-90.6	154	-26.6	1326	4197	1932	3389
mmu-r	ENSMU	610	-85.5	159	-23.2	1467	695	3486	9033
mmu-r	ENSMU	610	-85.2	162	-27.9	5624	10719	12016	1055
mmu-r	ENSMU	610	-83.4	164	-25.3	1342	107	6592	9179
mmu-r	ENSMU	610	-79.8	159	-30.6	9576	3654	9411	4525
mmu-r	Gm388	610	-64.8	162	-21.3	171	7687	4834	8311
mmu-r	ENSMU	610	-57.1	156	-15.3	5794	431	1456	8424
mmu-r	ENSMU	609	-105	160	-34.7	7144	9230	1369	3908
mmu-r	ENSMU	609	-81.8	159	-24.1	2123	4041	9065	710
mmu-r	ENSMU	609	-73.4	163	-20	1023	8031	9760	51
rco-mil	ENSMU	609	-67.8	166	-24.9	4148	9725	1706	5237
rco-mil	ENSMU	609	-67.8	166	-24.9	4148	9725	1706	5237
rco-mil	ENSMU	609	-67.8	166	-24.9	4148	9725	1706	5237
rco-mil	ENSMU	609	-67.8	166	-24.9	4148	9725	1706	5237
mmu-r	ENSMU	609	-54.2	164	-15	12153	11496	11365	8069
mmu-r	ENSMU	608	-97.3	157	-28.3	175	3368	3427	4383
mmu-r	ENSMU	608	-92.1	159	-26.7	2960	11685	6111	1223
mmu-r	ENSMU	608	-88	152	-22	7361	7445	7860	8106
mmu-r	ENSMU	607	-97.3	160	-26.5	4601	7198	2637	9595
mmu-r	ENSMU	607	-87.9	156	-23.7	9703	84	3637	7011
mmu-r	Gm388	607	-86.4	159	-26	3927	6900	5233	8983
mmu-r	ENSMU	607	-85	156	-26.7	1377	7148	3908	9236
mmu-r	Gm388	607	-79.8	162	-22.1	100	80	8530	8738
mmu-r	ENSMU	607	-70.8	163	-19.1	11059	4322	9202	5009
mmu-r	ENSMU	607	-68.7	153	-20.2	5124	1300	4040	964
mmu-r	ENSMU	607	-54.5	157	-17.9	922	11882	514	7320
mmu-r	ENSMU	606	-101	161	-34.3	4411	4899	7225	5358
mmu-r	ENSMU	606	-74.2	159	-21.1	7641	2793	9077	12414
mmu-r	ENSMU	606	-67.2	156	-20.1	1648	8583	13681	629
rco-mil	ENSMU	606	-67	160	-24.9	7247	9607	10728	12506
mmu-r	ENSMU	606	-57.8	158	-17.7	5831	9614	8614	807
mmu-r	ENSMU	606	-44.8	163	-16.3	11309	11443	9449	5006
mmu-r	ENSMU	605	-94.6	164	-34.9	6592	1332	9178	939
mmu-r	ENSMU	605	-82.7	165	-24.3	3239	5941	4661	1957
mmu-r	Gm388	605	-73.1	157	-21.9	2417	6200	4416	8927
mmu-r	Gm388	605	-72.9	157	-21.9	2417	6200	4416	8927
mmu-r	Gm388	605	-61	159	-20.3	4807	507	4882	3463
mmu-r	Gm412	604	-101	155	-29	709	1106	2665	1308
mmu-r	ENSMU	604	-79.1	167	-25.9	2478	7330	4679	7008
rco-mil	ENSMU	604	-66.5	160	-24.9	7247	10728	9607	12505
rco-mil	ENSMU	604	-66.5	160	-24.9	7247	10728	9607	12505
rco-mil	ENSMU	604	-66.5	160	-24.9	7247	10728	9607	12505
mmu-r	Gm388	604	-64.1	162	-18.7	171	7687	8309	4835
mmu-r	ENSMU	603	-97	159	-26.1	11542	7230	4274	9536
mmu-r	ENSMU	603	-87.5	160	-24.6	6165	4600	2574	676
mmu-r	Gm388	603	-79.5	164	-27.1	4395	8683	7263	6509
mmu-r	ENSMU	603	-78.6	159	-26.9	4559	4886	314	1724
mmu-r	ENSMU	603	-73.3	154	-20.1	9232	1377	3909	7149
mmu-r	ENSMU	603	-50.6	153	-14.8	6691	12813	2738	11863
mmu-r	Gm388	603	-48.2	159	-16.4	4835	171	8311	7689
mmu-r	ENSMU	602	-92.5	155	-27.4	717	7905	1680	7025

mmu-r	ENSMU	602	-78.8	158	-22.7	2204	2982	6460	9608
mmu-r	Gm388	602	-71.4	153	-19.1	8535	6575	5159	5238
rco-mil	ENSMU	602	-60.2	168	-19.4	4148	8762	5238	1905
rco-mil	ENSMU	602	-60.2	168	-19.4	4148	8762	5238	1905
rco-mil	ENSMU	602	-60.2	168	-19.4	4148	8762	5238	1905
mmu-r	ENSMU	602	-58.7	155	-18.3	905	1748	825	1283
mmu-r	ENSMU	602	-44.2	161	-19.3	9549	10702	9426	12810
mmu-r	ENSMU	601	-86.9	157	-27.8	3823	1455	2503	9564
mmu-r	ENSMU	601	-62.3	156	-18.2	4004	8527	10160	2050
mmu-r	ENSMU	601	-61.6	153	-17.9	3421	9922	7385	5585
mmu-r	ENSMU	601	-58.5	159	-18.3	6400	4534	12696	12681
mmu-r	Gm388	601	-55.7	155	-15.7	3008	7788	6246	7309
mmu-r	ENSMU	601	-36.9	158	-16.5	8114	335	737	1755
mmu-r	ENSMU	600	-96	156	-27.1	4202	1323	3385	1930
mmu-r	ENSMU	600	-91.1	174	-35.7	905	10089	5285	3233
mmu-r	ENSMU	600	-89.6	155	-27	3835	1305	1271	4709
mmu-r	ENSMU	600	-84.6	159	-24.1	6593	1333	940	9179
mmu-r	ENSMU	600	-78.7	154	-22	4872	8364	4558	5607
mmu-r	ENSMU	600	-77.4	158	-24.8	4200	543	8801	2299
rco-mil	ENSMU	600	-62.1	167	-24.3	4147	9726	1705	5237
mmu-r	ENSMU	600	-62	159	-18.7	967	5127	4041	1301
mmu-r	Gm388	600	-58.2	155	-17.1	5270	3528	6581	6503
mmu-r	ENSMU	600	-34.3	155	-10.3	14461	9676	10473	12197
mmu-r	ENSMU	599	-76.2	156	-24.4	671	6168	4596	2582
mmu-r	ENSMU	599	-71.9	156	-21.1	356	4857	1237	8349
mmu-r	ENSMU	599	-45.2	159	-18	2871	12669	177	7529
mmu-r	ENSMU	599	-43.2	157	-12.8	5229	3758	5113	5472
mmu-r	ENSMU	598	-85.1	163	-27.1	3603	3738	745	3965
mmu-r	ENSMU	598	-82.4	155	-22.7	159	1	43	660
mmu-r	ENSMU	598	-70.7	158	-20.5	10416	9268	5979	10341
mmu-r	ENSMU	598	-67.3	161	-21.4	7468	9817	13514	11661
mmu-r	ENSMU	598	-64.8	158	-20.4	12267	12546	397	9141
mmu-r	ENSMU	598	-43.9	156	-11.9	3632	1298	3641	3649
mmu-r	ENSMU	597	-82.8	159	-27.8	10211	1770	7037	9812
mmu-r	Gm388	597	-81.3	156	-23.9	5237	5157	6573	8530
mmu-r	ENSMU	597	-79.8	152	-21.5	697	3490	9028	1472
mmu-r	Gm388	597	-78.7	152	-21.2	3377	2068	8440	1007
mmu-r	ENSMU	597	-62.3	159	-19.2	4783	1798	7701	6073
mmu-r	ENSMU	597	-61.2	157	-18.9	1328	7584	11776	5688
mmu-r	ENSMU	596	-88	153	-24.2	4036	706	2120	9061
mmu-r	ENSMU	596	-83.4	153	-26.7	3387	1327	1934	4202
mmu-r	ENSMU	596	-68.9	152	-17.5	5127	1301	4041	964
mmu-r	Gm388	596	-68.4	153	-20.1	8981	7639	1544	6950
mmu-r	ENSMU	596	-65.5	155	-21.2	5643	5762	3399	5347
mmu-r	ENSMU	596	-62.8	157	-18	967	5127	1302	4041
mmu-r	Gm388	596	-57.3	155	-17.2	3008	6246	7790	7309
mmu-r	Gm388	596	-54.8	156	-15.9	171	4835	7689	8312
mmu-r	ENSMU	595	-92.6	167	-30.3	1330	6589	9179	940
mmu-r	ENSMU	595	-72.7	152	-20.2	9030	696	2881	9491
mmu-r	ENSMU	595	-70.9	152	-19.1	6206	3194	2960	3317
mmu-r	ENSMU	595	-56	158	-18.8	196	12855	11964	14466
mmu-r	ENSMU	595	-41.6	155	-12.3	5475	5113	3757	5230
mmu-r	ENSMU	595	-40.7	159	-14.1	12570	9810	4264	6867
mmu-r	ENSMU	594	-88.7	164	-24.8	5755	1401	4000	980
hiv1-m	ENSMU	594	-72.2	152	-19.2	9696	310	9008	11116
mmu-r	ENSMU	594	-53.9	156	-13.6	3426	2470	2508	2546
mmu-r	ENSMU	594	-52.5	150	-15.6	5863	3380	5576	4489
mmu-r	ENSMU	594	-49.3	155	-17.2	14281	4070	5930	3524
mmu-r	ENSMU	593	-91.8	154	-27.5	5720	10538	6329	12797
mmu-r	Gm388	593	-84.8	149	-22.5	4291	8888	6212	8475
mmu-r	ENSMU	593	-81.5	163	-27.8	3421	1155	3408	10933
mmu-r	ENSMU	593	-71.1	154	-21	960	5124	4037	1303
mmu-r	ENSMU	593	-68.6	159	-25.6	5057	5759	9936	163
mmu-r	ENSMU	593	-64.3	159	-19.2	7033	9487	7530	1913

mmu-r	ENSMU	593	-56.1	160	-16.4	2050	10160	4004	8528
mmu-r	ENSMU	593	-34.6	158	-11	8664	8955	10994	7523
mmu-r	ENSMU	592	-87.7	155	-25.4	10735	720	10002	12753
mmu-r	ENSMU	592	-62.9	152	-19.8	965	1302	4039	5128
mmu-r	ENSMU	592	-60.4	152	-20.9	6555	5086	12170	6270
mmu-r	ENSMU	592	-59.7	158	-17.7	14275	9087	8509	14366
mmu-r	ENSMU	592	-53.8	163	-17.4	4370	9405	9672	6420
mmu-r	ENSMU	592	-52.2	150	-14.8	362	4863	1242	8355
mmu-r	ENSMU	591	-86.7	154	-22.4	3731	4913	8405	1465
mmu-r	ENSMU	591	-86	158	-29.5	4058	142	5700	9485
mmu-r	ENSMU	591	-72.9	150	-20.5	375	4366	8204	10969
mmu-r	ENSMU	591	-56.2	162	-17.6	5148	5750	1448	4318
mmu-r	ENSMU	591	-39.1	156	-14.6	5233	1125	1760	3203
mmu-r	ENSMU	590	-70.9	157	-24.4	1670	5571	5254	3909
mmu-r	ENSMU	590	-70.3	155	-22.6	7644	2792	12416	9077
mmu-r	ENSMU	590	-67.8	156	-22.4	11	9722	1517	13257
mmu-r	ENSMU	590	-60.9	153	-17.1	5687	13187	6421	188
mmu-r	ENSMU	590	-60.1	155	-20.3	1302	4039	962	5127
mmu-r	Gm388	590	-59.2	154	-21.6	8845	8969	4739	3035
mmu-r	ENSMU	590	-49.2	159	-14.8	7236	9160	4364	10870
mmu-r	ENSMU	589	-98.9	157	-33.4	46	12382	12348	1
mmu-r	ENSMU	589	-78.9	152	-23.4	6673	3874	3681	7907
mmu-r	ENSMU	589	-60.9	158	-19.2	6681	10263	13711	5840
mmu-r	Gm388	589	-58.9	152	-16	3165	7144	4914	4966
mmu-r	ENSMU	589	-53.8	161	-18.5	3019	1730	5748	5087
mmu-r	ENSMU	589	-50.2	153	-15.6	10706	2609	13680	10085
mmu-r	ENSMU	589	-50	153	-14.4	1420	9669	3253	3502
mmu-r	Gm388	588	-63.3	151	-19.8	2179	8644	2612	3459
mmu-r	ENSMU	587	-80.3	152	-25.1	2168	4396	4415	8827
mmu-r	Gm388	587	-69.2	156	-21.6	5233	6904	3930	8988
mmu-r	ENSMU	587	-61.6	153	-21	692	2746	12086	7069
mmu-r	ENSMU	587	-61.4	150	-20	430	4115	149	848
mmu-r	ENSMU	587	-54.3	152	-14.8	2556	7339	7838	8084
mmu-r	Gm388	587	-54.1	154	-15	172	7691	4835	8312
mmu-r	ENSMU	587	-53.4	152	-14.4	2556	7339	7838	8084
mmu-r	ENSMU	587	-50.9	152	-16.6	4665	4653	2285	374
mmu-r	Gm412	587	-49	151	-13.5	1627	2620	2823	3478
mmu-r	ENSMU	587	-41.4	151	-15.1	5109	2597	5234	3757
mmu-r	ENSMU	587	-33.7	156	-10.1	212	11833	12578	14467
mmu-r	ENSMU	586	-73.6	149	-20	383	4883	8375	1436
mmu-r	ENSMU	586	-71.4	155	-20.7	6003	5851	8292	8673
mmu-r	ENSMU	586	-69.2	151	-19.9	3851	2091	1273	4715
mmu-r	ENSMU	586	-40	151	-13.1	11594	1818	11358	11373
mmu-r	ENSMU	586	-33.4	152	-9.29	9148	4984	496	4096
mmu-r	ENSMU	585	-81.4	156	-26.3	14296	3060	3425	10934
mmu-r	ENSMU	585	-63.6	155	-18.2	7018	1566	8108	8252
mmu-r	ENSMU	585	-63.2	150	-17.5	9555	5740	1501	2472
mmu-r	ENSMU	585	-52.5	154	-18.5	4152	1699	10788	11684
mmu-r	ENSMU	585	-47.6	153	-14	9171	9780	8470	5402
mmu-r	Gm388	584	-83.5	153	-23.4	6208	4293	8475	8888
mmu-r	ENSMU	584	-48.5	160	-16.6	6417	9668	4371	9404
mmu-r	ENSMU	584	-28.4	148	-9.05	5608	6479	3576	2374
mmu-r	ENSMU	583	-60.8	155	-18.8	1301	4040	964	5127
mmu-r	ENSMU	582	-81.9	149	-24.7	5940	3240	1956	4665
mmu-r	Gm388	582	-67.3	152	-22.5	6200	8675	4416	8927
mmu-r	ENSMU	582	-44.8	157	-18.2	2871	12667	178	7529
mmu-r	ENSMU	581	-58.6	148	-16.5	1301	967	4042	5124
mmu-r	ENSMU	581	-30.8	155	-17.8	6296	5192	4946	4840
mmu-r	ENSMU	580	-71.5	148	-20.2	2750	12087	7070	692
mmu-r	ENSMU	580	-66.3	156	-18.7	1226	6245	7020	6352
mmu-r	Gm388	579	-80.3	152	-28.3	1195	6520	2816	5420
mmu-r	ENSMU	579	-57.5	151	-15.8	9492	2882	698	9031
mmu-r	ENSMU	579	-56.6	152	-18.2	10212	7793	2611	12309
mmu-r	ENSMU	578	-64.3	151	-19.5	1301	5123	964	4036

mmu-r	ENSMU	577	-61.2	148	-17.8	1298	4041	5124	965
mmu-r	ENSMU	576	-69.3	149	-20	4555	1296	4000	4406
mmu-r	ENSMU	576	-61.1	148	-21.3	3650	4523	9408	9578
mmu-r	ENSMU	576	-24.1	152	-6.66	8486	7277	2851	8318
mmu-r	ENSMU	574	-38.2	147	-11.7	11358	506	1993	7735
mmu-r	Gm388	573	-76.6	151	-22.5	4288	6207	8474	8887
mmu-r	Gm388	573	-61.8	147	-18.9	1545	8568	8981	6955
mmu-r	ENSMU	572	-63.9	146	-18.5	1289	5717	4325	10218
mmu-r	Gm388	571	-46.2	151	-14.5	2110	2545	2981	4861
mmu-r	ENSMU	570	-42.3	145	-13.6	926	7322	11889	517
mmu-r	ENSMU	569	-44.1	145	-13.2	374	2285	4654	4666
mmu-r	ENSMU	567	-38.5	144	-12.6	11752	4840	6809	12533
mmu-r	ENSMU	565	-63.6	142	-23.3	3656	9413	9579	4526
mmu-r	ENSMU	562	-31	142	-8.47	5414	5426	6337	7408
mmu-r	ENSMU	541	-87.1	200	-37.3	5321	6337	3764	
mmu-r	Gm412	537	-98.4	190	-39.6	2158	2174	725	
mmu-r	ENSMU	525	-81	178	-31.1	636	4094	3748	
mmu-r	ENSMU	525	-54.2	175	-18.4	1757	1773	1789	
mmu-r	ENSMU	525	-54.2	175	-18.4	1757	1773	1789	
mmu-r	ENSMU	525	-54.2	175	-18.4	1757	1773	1789	
mmu-r	ENSMU	523	-76.6	205	-38.2	3474	565	795	
mmu-r	Gm412	520	-70	187	-30.6	1959	1975	3003	
mmu-r	ENSMU	502	-90.5	191	-43.3	6971	12481	7060	
mmu-r	ENSMU	502	-89.4	179	-34.4	3363	5939	8788	
mmu-r	ENSMU	501	-93	180	-35.5	3425	171	2449	
mmu-r	ENSMU	499	-93.1	190	-45.8	6972	12482	7061	
mmu-r	ENSMU	495	-76.8	181	-33.8	11218	283	617	
mmu-r	ENSMU	494	-79.9	182	-33.2	11216	10971	10652	
mmu-r	ENSMU	494	-77.3	195	-45.9	8420	9508	1851	
mmu-r	ENSMU	494	-49	171	-17.5	1958	6139	14130	
mmu-r	ENSMU	492	-65.8	168	-24.6	6215	3564	9266	
mmu-r	ENSMU	490	-66.9	167	-25.6	635	4093	3748	
mmu-r	ENSMU	490	-62	167	-24.4	635	4093	3748	
mmu-r	ENSMU	489	-88.4	166	-30.8	3876	6295	3428	
mmu-r	ENSMU	489	-52.5	169	-18.5	5425	7413	505	
mmu-r	ENSMU	488	-85.2	191	-44.6	992	6627	2794	
mmu-r	ENSMU	487	-83.1	177	-34.5	1273	4013	5398	
mmu-r	ENSMU	487	-52.8	165	-20.1	5417	505	7411	
mmu-r	ENSMU	486	-90	163	-32.4	3803	4542	169	
mmu-r	ENSMU	486	-41.9	165	-16.2	5520	3081	2	
mmu-r	ENSMU	485	-73	187	-36.4	427	803	817	
mmu-r	ENSMU	485	-69.8	163	-24.1	2724	5425	3040	
mmu-r	ENSMU	485	-47.5	168	-17.8	8568	4593	3389	
mmu-r	ENSMU	485	-42.1	183	-31	8519	3666	3221	
mmu-r	ENSMU	484	-74.3	164	-25.3	2758	5354	5456	
mmu-r	ENSMU	484	-70.6	175	-28.7	13239	5266	113	
mmu-r	ENSMU	483	-72.2	173	-25.2	1272	2089	3849	
mmu-r	ENSMU	483	-62.5	165	-24	638	3749	4094	
mmu-r	ENSMU	482	-93.9	164	-34.4	5341	715	6356	
mmu-r	ENSMU	482	-65.9	165	-22.5	4339	5636	2451	
mmu-r	ENSMU	482	-55.1	184	-29.6	3383	3223	10671	
mmu-r	Gm388	481	-88.1	172	-33.2	977	1668	8240	
mmu-r	ENSMU	481	-78.8	167	-27.3	687	10432	10793	
mmu-r	ENSMU	481	-64.3	175	-28.7	5378	2187	4535	
mmu-r	ENSMU	481	-62	172	-26.3	5050	7702	11626	
mmu-r	ENSMU	480	-51.1	163	-22	7169	12569	4001	
mmu-r	Gm388	479	-84	161	-29.5	738	7482	263	
mmu-r	ENSMU	479	-69.5	171	-27.8	952	2463	1116	
rco-mil	ENSMU	479	-66.2	167	-23.1	1537	4735	9775	
rco-mil	ENSMU	479	-66.2	167	-23.1	1537	4735	9775	
rco-mil	ENSMU	479	-66.2	167	-23.1	1537	4735	9775	
mmu-r	ENSMU	479	-60.7	162	-22.2	4614	536	3963	
mmu-r	ENSMU	479	-49	174	-24.2	8517	8498	10058	
mmu-r	ENSMU	478	-76.9	173	-34.1	4611	532	3958	

mmu-r	ENSMU	478	-34.9	163	-13.9	2943	11835	5958
mmu-r	ENSMU	477	-74.1	173	-33.8	11221	283	619
mmu-r	ENSMU	476	-87.3	164	-31.4	3835	1264	4710
mmu-r	ENSMU	476	-69.5	170	-27.6	5941	10226	5763
mmu-r	ENSMU	476	-55.8	162	-25.7	5861	2236	4095
mmu-r	ENSMU	476	-54	161	-19.7	10149	10170	8531
mmu-r	Gm388	476	-47.3	173	-24.1	8710	510	4113
mmu-r	Gm388	475	-79	165	-28.8	8539	6090	7485
mmu-r	ENSMU	475	-72	162	-26.3	10232	5941	5767
mmu-r	ENSMU	474	-74.4	166	-35	13544	11036	74
mmu-r	ENSMU	474	-69.7	180	-30	3423	10416	10339
mmu-r	ENSMU	474	-37.6	175	-24.9	8520	3668	3222
mmu-r	ENSMU	473	-76.8	164	-27.5	158	1169	1283
mmu-r	ENSMU	473	-70.3	167	-30.6	2194	3741	10598
mmu-r	ENSMU	473	-67.6	163	-23.5	3704	431	3862
mmu-r	ENSMU	473	-63.6	173	-25.2	2185	1670	7865
rco-mil	ENSMU	473	-60.7	162	-22.6	4735	1537	9773
mmu-r	ENSMU	473	-59.8	161	-23.2	2885	9747	2648
mmu-r	ENSMU	473	-56.5	164	-21	5678	6100	3681
mmu-r	ENSMU	473	-50.3	174	-22.8	10849	3781	11339
mmu-r	ENSMU	473	-48.4	163	-18.7	11964	12425	1856
mmu-r	ENSMU	472	-77.6	162	-31.7	46	4551	10427
mmu-r	ENSMU	472	-71.5	163	-26.1	9484	6577	5322
mmu-r	ENSMU	472	-61	180	-33.1	3743	10600	11983
mmu-r	ENSMU	472	-54.9	169	-25.6	2190	6774	2244
mmu-r	Gm412	472	-50.4	159	-19.9	3791	2705	3106
mmu-r	ENSMU	472	-34.6	179	-24.3	8519	3666	3221
mmu-r	ENSMU	471	-83.3	171	-32.4	1178	159	1290
mmu-r	ENSMU	471	-68.3	160	-26.6	9064	8233	2445
mmu-r	Gm388	471	-61.6	171	-27.6	7258	129	6893
mmu-r	ENSMU	471	-61.2	164	-26.2	4143	2155	3014
mmu-r	ENSMU	470	-82	162	-30.4	1956	3238	776
mmu-r	ENSMU	470	-58.1	160	-23.2	7182	7102	4073
mmu-r	ENSMU	470	-54.9	167	-22.8	2116	631	777
mmu-r	ENSMU	470	-45.3	158	-16.5	505	5417	7411
mmu-r	ENSMU	469	-69.2	164	-24.6	10228	5763	5940
mmu-r	ENSMU	469	-67.1	161	-24.3	2456	2493	2531
mmu-r	Gm412	469	-61.9	160	-24.1	2714	2915	891
mmu-r	ENSMU	469	-60.7	161	-23	11244	11320	6361
mmu-r	ENSMU	469	-60	168	-25	445	2960	3293
mmu-r	ENSMU	469	-59.9	164	-22.5	7182	7102	4070
mmu-r	ENSMU	469	-56	164	-20.1	6375	14231	3089
mmu-r	Gm388	468	-84.1	167	-39.8	8424	6226	8728
mmu-r	ENSMU	468	-81.4	169	-33.2	13265	4323	6546
mmu-r	ENSMU	468	-69.6	164	-26.6	13263	5952	5914
mmu-r	ENSMU	468	-55.1	159	-19.8	1565	10734	9226
mmu-r	ENSMU	467	-75.2	162	-29.7	683	903	1374
mmu-r	Gm388	467	-72.3	161	-30.2	7288	461	2145
rco-mil	ENSMU	467	-61.6	163	-25.2	8006	4329	2817
rco-mil	ENSMU	467	-61.6	163	-25.2	8006	4329	2817
mmu-r	ENSMU	467	-50	168	-18.4	6141	1980	3659
mmu-r	ENSMU	467	-48.9	169	-20.6	1110	9225	9692
mmu-r	ENSMU	466	-84.6	164	-35.3	4222	892	4046
mmu-r	ENSMU	466	-78.2	158	-27.6	2170	1327	1347
mmu-r	ENSMU	466	-75.9	161	-28.7	4601	9876	2898
mmu-r	ENSMU	466	-70.3	164	-28.3	1405	4356	4683
mmu-r	ENSMU	466	-48.1	159	-19.2	4999	305	7296
mmu-r	ENSMU	466	-32.3	161	-12.6	5433	5451	12516
mmu-r	ENSMU	466	-27.5	167	-14.5	6490	6466	6452
mmu-r	ENSMU	465	-75.2	164	-27.3	6517	9440	9512
mmu-r	ENSMU	465	-74.3	158	-28.8	9265	4072	10646
mmu-r	ENSMU	465	-70.5	168	-27.9	1141	5465	6063
mmu-r	ENSMU	465	-58.9	169	-26.5	10213	8637	5344
mmu-r	ENSMU	465	-55.2	161	-19.6	6857	4050	4337

mmu-r	ENSMU	464	-77.8	163	-30.2	8787	3362	5935
mmu-r	ENSMU	464	-72.4	157	-26.9	401	3780	3519
mmu-r	ENSMU	464	-65.4	161	-26.9	86	3638	1219
mmu-r	ENSMU	464	-64.6	159	-25.9	6331	540	5315
mmu-r	ENSMU	464	-54.6	162	-23.4	1124	10464	1153
mmu-r	ENSMU	464	-45.2	163	-19.5	1990	7856	3513
mmu-r	Gm388	464	-39.7	163	-14.4	5317	8281	1303
mmu-r	ENSMU	464	-33.3	157	-14.5	6613	7042	13632
mmu-r	ENSMU	463	-70.3	167	-28.1	4282	2509	50
mmu-r	Gm412	463	-66.3	165	-26.7	3477	1448	2688
mmu-r	Gm388	463	-65.1	166	-26.3	4752	8465	404
mmu-r	ENSMU	463	-64.6	160	-22.7	4222	103	8280
mmu-r	ENSMU	463	-59.4	158	-21.7	1999	2966	1029
mmu-r	ENSMU	463	-53	159	-21	443	8937	1399
mmu-r	ENSMU	463	-46.3	155	-16.7	13831	9668	12128
mmu-r	ENSMU	463	-43.2	167	-20.1	2831	4017	9281
mmu-r	ENSMU	462	-83	156	-29.4	2167	1324	1341
mmu-r	ENSMU	462	-66.1	162	-24.5	9010	11121	5941
mmu-r	ENSMU	462	-61	162	-24.1	11058	907	4488
mmu-r	ENSMU	462	-49.4	154	-17.1	2480	2518	2556
mmu-r	ENSMU	462	-47.6	156	-20.5	10090	4633	8927
mmu-r	ENSMU	462	-26.7	167	-12.5	6831	7780	502
mmu-r	ENSMU	461	-81.9	157	-29.2	3425	3874	6297
mmu-r	ENSMU	461	-71.7	164	-25.7	2374	836	14166
mmu-r	ENSMU	461	-66.1	159	-25	4339	6857	4052
mmu-r	ENSMU	461	-65	159	-24.6	7661	8662	2988
mmu-r	Gm388	461	-62.2	159	-22.9	8381	2680	6630
mmu-r	ENSMU	461	-61.2	158	-21	1177	1111	1076
mmu-r	ENSMU	461	-46.9	176	-26.4	8388	12573	3188
mmu-r	Gm388	460	-71.2	161	-24.1	6067	4325	734
mmu-r	ENSMU	460	-61	158	-25.2	8786	5550	13737
mmu-r	ENSMU	460	-56.9	164	-24	144	2727	1695
mmu-r	ENSMU	460	-54.3	159	-18.6	2911	1981	2801
mmu-r	Gm412	460	-52.9	173	-26.6	4046	1994	2519
mmu-r	ENSMU	460	-33.3	162	-13.7	2192	5818	4685
mmu-r	ENSMU	459	-76.8	168	-36.8	2356	8495	6997
mmu-r	ENSMU	459	-69	159	-26.5	10435	689	10796
mmu-r	ENSMU	459	-63.8	157	-25.1	8767	3128	8630
mmu-r	Gm412	459	-62.7	159	-23.5	2915	888	2711
mmu-r	Gm388	459	-60.3	167	-27.4	8517	2016	3393
mmu-r	Gm388	459	-57.3	158	-21.3	3251	7451	273
mmu-r	ENSMU	459	-50.1	156	-21.6	3158	12337	36
mmu-r	ENSMU	459	-35.2	163	-12.3	6374	2887	13799
mmu-r	Gm412	459	-31.2	165	-16.9	1742	2118	3266
mmu-r	ENSMU	458	-65.3	159	-25.9	4485	11058	907
mmu-r	Gm388	458	-58.9	155	-22.5	4834	2241	169
mmu-r	Gm388	458	-53.4	170	-20.1	7639	8979	6949
rco-mil	ENSMU	458	-50.9	166	-19.7	13047	3396	6083
mmu-r	ENSMU	458	-45.9	160	-15.9	410	9353	8218
mmu-r	ENSMU	458	-45.3	162	-17.7	8996	6328	3204
mmu-r	Gm388	458	-40.3	166	-16.3	2537	75	3916
mmu-r	Gm388	458	-32.2	156	-12.4	3497	953	1584
mmu-r	ENSMU	457	-72.6	160	-28.3	10388	3433	5070
mmu-r	Gm388	457	-64.5	157	-25.1	8518	3392	2015
mmu-r	ENSMU	457	-60.9	159	-25.1	7103	4071	7181
mmu-r	ENSMU	457	-60.2	157	-24.1	6213	9263	3561
mmu-r	ENSMU	457	-56.8	161	-24.5	6543	2572	5530
mmu-r	ENSMU	457	-48.2	154	-20	3368	11123	2253
mmu-r	ENSMU	457	-48	153	-18.2	3872	436	3708
mmu-r	ENSMU	457	-46.1	153	-17.3	629	7842	7911
mmu-r	ENSMU	457	-45.8	155	-17.6	8637	10212	5346
mmu-r	ENSMU	457	-45.4	167	-22.2	1365	752	1072
mmu-r	ENSMU	457	-45.3	160	-22.7	10619	12494	12298
mmu-r	ENSMU	457	-44	154	-15.9	5052	7707	11627

mmu-r	Gm388	457	-41.5	165	-20.9	8709	509	4114
mmu-r	Gm388	457	-41.1	162	-16.3	864	3871	3802
mmu-r	ENSMU	457	-36.2	161	-14.6	4572	12643	8935
mmu-r	ENSMU	457	-35.2	162	-16.2	9202	5848	10094
mmu-r	ENSMU	456	-81.2	160	-32.6	11150	10485	4402
mmu-r	ENSMU	456	-71.3	159	-29	9090	4634	5592
mmu-r	ENSMU	456	-68.7	156	-25.1	3879	3428	6296
mmu-r	ENSMU	456	-65.7	164	-26.8	2005	1072	9017
mmu-r	ENSMU	456	-65	160	-26.8	6497	5396	4007
mmu-r	ENSMU	456	-63.8	157	-24.2	1356	1332	7077
mmu-r	ENSMU	456	-61.7	157	-21.6	4621	2930	735
mmu-r	Gm412	456	-61.1	157	-22.9	2714	3151	890
mmu-r	ENSMU	456	-60.4	163	-21.6	11586	12475	10064
mmu-r	ENSMU	456	-59.6	155	-20.8	5086	6412	7575
rco-mil	ENSMU	456	-57.7	156	-20.1	13048	3399	6086
mmu-r	ENSMU	456	-57.3	154	-19.8	9797	619	7216
rco-mil	ENSMU	456	-56.4	164	-23.1	13048	3399	6086
mmu-r	ENSMU	456	-55.1	158	-21	859	10382	8130
mmu-r	ENSMU	456	-54	156	-20.1	1797	3471	4040
mmu-r	ENSMU	456	-52.9	154	-19.9	5765	7224	7560
mmu-r	ENSMU	456	-52.5	156	-20.1	5767	5942	10233
mmu-r	ENSMU	456	-45.8	156	-16.9	4012	11794	4703
mmu-r	ENSMU	456	-42.6	155	-17.1	13004	13704	438
mmu-r	ENSMU	456	-42.2	159	-17.1	2826	7561	8936
mmu-r	ENSMU	456	-41.1	157	-16.3	12427	7328	3524
mmu-r	Gm388	456	-40.6	156	-15.1	75	8300	4114
mmu-r	ENSMU	456	-34.2	162	-15.2	4591	9530	3388
mmu-r	ENSMU	456	-32.6	162	-12.3	6144	1962	14137
mmu-r	ENSMU	455	-84.4	156	-33.8	2499	2196	8165
mmu-r	Gm388	455	-69.6	159	-25.7	6527	8492	1561
mmu-r	ENSMU	455	-67.7	158	-27.8	14331	2448	8165
mmu-r	Gm388	455	-66	158	-26.3	2678	8377	6630
mmu-r	ENSMU	455	-65.1	164	-28.2	4303	7317	11261
mmu-r	ENSMU	455	-64.6	153	-22.2	4016	8196	8031
mmu-r	ENSMU	455	-62.9	160	-23.3	4059	881	1052
mmu-r	Gm388	455	-60.7	163	-23.5	3709	8868	1364
mmu-r	Gm388	455	-57.1	155	-21.7	8352	5102	6186
mmu-r	ENSMU	455	-55.6	154	-21.2	4883	4917	919
mmu-r	ENSMU	455	-55.6	155	-20.2	8612	11537	6970
mmu-r	ENSMU	455	-53.9	155	-20.9	8789	10682	10706
mmu-r	ENSMU	455	-52.4	156	-21.8	3794	5219	5453
mmu-r	ENSMU	455	-50.3	160	-22.3	8162	6585	4638
mmu-r	ENSMU	455	-47.5	170	-24.4	10394	11798	216
mmu-l	ENSMU	455	-43.1	160	-20.3	3056	3664	3222
mmu-r	ENSMU	455	-36.7	162	-13.2	6049	3279	1606
mmu-r	ENSMU	455	-36.3	160	-15	4521	10826	12693
mmu-r	Gm388	454	-72.9	155	-31	4417	6463	1738
mmu-r	Gm388	454	-65.6	163	-24.8	7283	457	2146
mmu-r	ENSMU	454	-63.9	156	-22.7	3612	3528	876
mmu-r	ENSMU	454	-63.9	153	-22.4	10281	11010	11907
mmu-r	ENSMU	454	-63.5	157	-27.8	4335	8007	2819
mmu-r	ENSMU	454	-62	153	-23.8	10296	1103	3056
mmu-r	ENSMU	454	-59.3	159	-23.8	107	2109	696
mmu-r	ENSMU	454	-56.6	156	-23.3	1762	959	3505
mmu-r	Gm388	454	-52.8	155	-20.9	2143	7285	455
mmu-r	ENSMU	454	-51.4	153	-21.2	8740	6136	6874
mmu-r	Gm388	454	-47	153	-20.3	3917	2537	74
mmu-r	ENSMU	454	-45.2	157	-18.6	6728	2191	3168
mmu-r	ENSMU	454	-43.7	158	-14.8	1198	2630	2357
mmu-r	ENSMU	454	-41.8	154	-14.4	6604	5431	9602
mmu-r	ENSMU	454	-37.1	155	-13.9	12427	3524	7328
mmu-r	Gm412	454	-34.2	163	-15.7	1742	3265	2115
mmu-r	Gm412	454	-29.9	159	-13.5	1742	2117	3265
mmu-r	ENSMU	454	-28.7	159	-13	6831	7780	502

mmu-r	ENSMU	453	-74.3	152	-28.6	1261	1165	1336
mmu-r	Gm388	453	-70.4	157	-29.8	3257	2329	3600
mmu-r	ENSMU	453	-70.2	160	-30.4	1226	2271	5522
mmu-r	ENSMU	453	-69.6	164	-30	12604	4422	7382
mmu-r	Gm388	453	-64.3	162	-24.2	17	4796	623
mmu-r	ENSMU	453	-60.7	163	-24.4	9485	5323	6578
mmu-r	ENSMU	453	-54.1	153	-19.3	6829	8656	8135
mmu-r	ENSMU	453	-51.5	158	-18.9	7459	6967	1619
mmu-r	ENSMU	453	-51.4	158	-20.1	10784	8289	4145
mmu-r	ENSMU	453	-48.7	154	-21.4	12685	9195	10160
mmu-r	ENSMU	453	-47	164	-20.7	5759	2599	3839
mmu-r	ENSMU	453	-46.2	159	-20.4	7745	12342	9762
mmu-r	ENSMU	453	-44.8	154	-19	672	7336	7170
mmu-r	ENSMU	453	-44.1	151	-14.7	2467	2505	2543
mmu-r	ENSMU	453	-43.3	168	-20.8	8998	6330	3206
mmu-r	ENSMU	453	-38.4	155	-14	6612	7047	13633
mmu-r	ENSMU	453	-25.6	155	-11	6831	7780	504
mmu-r	ENSMU	453	-25.5	155	-10.8	6831	7780	504
mmu-r	ENSMU	453	-22.7	155	-9.37	6831	502	7782
mmu-r	ENSMU	453	-22.6	155	-9.37	6831	502	7782
mmu-r	ENSMU	452	-66.5	154	-27.3	3789	1577	5820
rco-mi	Gm388	452	-63.5	159	-27.1	8491	8235	7280
rco-mi	Gm388	452	-63.5	159	-27.1	8491	8235	7280
mmu-r	ENSMU	452	-62.2	165	-26.5	4319	6999	2844
mmu-r	Gm388	452	-60.9	167	-28	1340	3735	797
mmu-r	ENSMU	452	-56.3	161	-20.4	4302	11415	5050
mmu-r	ENSMU	452	-55.2	153	-21	784	4230	3445
mmu-r	Gm388	452	-52.9	159	-22.1	8607	675	3966
mmu-r	ENSMU	452	-52.9	162	-21.4	1588	2697	2229
mmu-r	ENSMU	452	-47.5	155	-18.6	3214	4651	2260
mmu-r	Gm412	452	-32.6	165	-16.5	1742	3266	2116
mmu-r	ENSMU	452	-31.7	155	-14.4	502	5032	3178
mmu-r	ENSMU	451	-83.1	154	-32.8	9328	7975	13263
mmu-r	Gm388	451	-81.7	157	-30.2	741	7478	265
mmu-r	Gm388	451	-79.4	153	-27.2	8540	7482	6089
mmu-r	ENSMU	451	-79.2	157	-29.4	7192	2631	8902
mmu-r	ENSMU	451	-74.8	164	-29	2209	2490	2646
mmu-r	ENSMU	451	-64.6	156	-23.8	13929	12328	13192
mmu-r	ENSMU	451	-58.7	160	-22.9	10427	4548	41
mmu-r	ENSMU	451	-52.4	156	-23.6	2641	5994	3931
mmu-r	ENSMU	451	-50.9	155	-18.9	12056	12503	6520
mmu-r	ENSMU	451	-50.8	155	-18.5	4921	1324	4906
mmu-r	ENSMU	451	-50	152	-18.7	2334	543	7126
mmu-r	ENSMU	451	-49.8	154	-19.9	2179	1739	13417
mmu-r	Gm388	451	-47.3	157	-18.6	2145	456	7284
mmu-r	Gm388	451	-45.9	160	-20.5	1288	6421	9004
mmu-r	ENSMU	451	-42.8	157	-15.6	7636	3710	7831
mmu-r	ENSMU	451	-41.3	155	-14.6	1927	12415	7626
mmu-r	ENSMU	451	-28.4	165	-13.5	6833	7776	502
mmu-r	ENSMU	450	-69.8	152	-25.9	12177	7462	12624
mmu-r	ENSMU	450	-69.4	161	-29.4	3793	5821	1577
mmu-r	ENSMU	450	-66.9	154	-27	1523	1575	11055
mmu-r	ENSMU	450	-62.2	157	-22.2	1223	8196	5614
mmu-r	ENSMU	450	-59.9	160	-22	1550	4486	11051
mmu-r	ENSMU	450	-58.8	153	-21.7	2511	7541	4904
mmu-r	ENSMU	450	-52.5	152	-18.8	7024	5722	6249
mmu-r	Gm388	450	-52.4	153	-18.8	8521	3394	2018
mmu-r	Gm388	450	-51.7	156	-20.6	2452	76	4880
mmu-r	ENSMU	450	-48.1	153	-19	2098	7691	14350
mmu-r	ENSMU	450	-46.3	155	-15.8	6734	8972	10418
mmu-r	ENSMU	450	-46	160	-20.9	5759	2599	3836
mmu-r	ENSMU	450	-45.7	160	-16.9	1111	9226	9693
mmu-r	ENSMU	450	-43.7	155	-17.1	2252	3369	11124
mmu-r	ENSMU	450	-41.7	164	-19.9	12806	5491	11609

mmu-r	ENSMU	450	-37.5	162	-19	12457	760	9890
mmu-r	ENSMU	449	-78.1	152	-29.7	6300	3880	3431
mmu-r	Gm388	449	-69.5	162	-28.6	3387	2016	8523
mmu-r	ENSMU	449	-66.1	153	-24.5	1330	2171	1870
mmu-r	ENSMU	449	-65.4	152	-22.7	7678	10389	5070
mmu-r	ENSMU	449	-62.6	157	-22.6	4305	4646	9534
mmu-r	ENSMU	449	-62.5	155	-21.8	2257	4652	3214
mmu-r	ENSMU	449	-59.8	160	-25.4	1264	4290	1147
mmu-r	ENSMU	449	-59.3	156	-24.8	618	635	4196
mmu-r	Gm388	449	-58	155	-19.7	8927	4415	2417
mmu-r	ENSMU	449	-57.5	158	-22.2	371	2767	3144
mmu-r	Gm412	449	-55.9	155	-20.4	3761	3559	3974
mmu-r	ENSMU	449	-54.3	152	-22.4	4238	1056	5625
mmu-r	ENSMU	449	-52	153	-19.2	8790	10706	10682
rco-mil	ENSMU	449	-51.5	161	-21.3	13047	3396	6079
rco-mil	ENSMU	449	-51.5	161	-21.3	13047	3396	6079
mmu-r	ENSMU	449	-50.6	158	-21.5	11282	5082	12170
rco-mil	ENSMU	449	-50	161	-20.9	13047	3396	6079
mmu-r	ENSMU	449	-49.8	161	-18.2	4026	1720	4841
mmu-r	ENSMU	449	-47.3	154	-17.1	2347	6341	1445
mmu-r	ENSMU	449	-45.7	153	-16.9	171	3469	3128
mmu-r	ENSMU	449	-42.5	154	-15.5	11491	4842	9397
mmu-r	ENSMU	449	-41.7	159	-16.1	12425	1857	11962
mmu-r	ENSMU	449	-37.8	150	-14.8	246	8441	9652
mmu-r	ENSMU	448	-74	154	-28.3	1800	4040	554
mmu-r	Gm388	448	-70	152	-29.2	3875	6541	7755
mmu-r	ENSMU	448	-65.3	164	-29.1	7189	2633	8900
mmu-r	Gm388	448	-62.9	158	-23.5	8142	805	5373
mmu-r	Gm412	448	-62.7	166	-29.1	3986	4126	234
mmu-r	Gm388	448	-61.7	152	-21.6	4438	8883	1787
mmu-r	ENSMU	448	-60.5	157	-23.9	8184	2425	13212
mmu-r	ENSMU	448	-57.6	153	-22.4	1427	3673	9539
mmu-r	ENSMU	448	-55.2	155	-21.6	7673	4987	7425
mmu-r	ENSMU	448	-52.6	153	-17.9	282	6270	10083
rco-mil	ENSMU	448	-49.5	151	-19.5	714	4338	4881
rco-mil	ENSMU	448	-49.5	151	-19.5	714	4338	4881
rco-mil	ENSMU	448	-49.5	151	-19.5	714	4338	4881
rco-mil	ENSMU	448	-49.5	151	-19.5	714	4338	4881
mmu-r	ENSMU	448	-48.9	155	-17.2	12397	11043	6748
mmu-r	ENSMU	448	-48.4	163	-25	1023	4962	2454
mmu-r	Gm388	448	-43.7	159	-16.1	3034	4739	8965
mmu-r	ENSMU	448	-42.3	160	-20.2	2910	6701	8979
mmu-r	ENSMU	448	-38.5	157	-16.8	5174	3719	4088
mmu-r	Gm388	448	-37.6	153	-15.1	2611	2798	601
mmu-r	ENSMU	448	-32.8	156	-12.1	7589	8334	2817
mmu-r	ENSMU	448	-28.8	153	-11.3	4087	938	1643
mmu-r	ENSMU	448	-28.7	155	-13.8	5346	2333	5379
mmu-r	ENSMU	447	-62.6	154	-22.8	7460	6964	1616
mmu-r	ENSMU	447	-62.5	155	-28.4	10987	8413	12359
mmu-r	Gm388	447	-59.4	153	-22.7	1439	2257	5457
mmu-r	ENSMU	447	-59.2	161	-22	10388	5068	3432
mmu-r	ENSMU	447	-58.9	154	-24.5	5844	3472	7182
mmu-r	ENSMU	447	-58.9	155	-22.1	3407	3420	1150
mmu-r	Gm388	447	-58.8	151	-21.5	2776	8580	196
mmu-r	ENSMU	447	-58.2	152	-22.9	3777	8207	80
rco-mil	ENSMU	447	-52	158	-22.3	12199	8616	2975
rco-mil	ENSMU	447	-52	158	-22.3	12199	8616	2975
mmu-r	ENSMU	447	-51.1	156	-20.6	5830	9542	5316
mmu-r	ENSMU	447	-51.1	155	-19.6	11971	7739	6535
mmu-r	ENSMU	447	-50	157	-18.4	4440	2621	3607
mmu-r	ENSMU	447	-40.3	151	-16.4	9753	4004	10991
mmu-r	ENSMU	447	-37.3	150	-14.1	9981	12457	12400
mmu-r	ENSMU	447	-35	157	-16.1	1632	1817	2154
mmu-r	ENSMU	447	-25.3	152	-9.21	6450	6490	6464

mmu-r	ENSMU	446	-75.5	154	-30.8	1100	8489	9133
mmu-r	Gm388	446	-68.9	158	-29.3	195	218	6455
mmu-r	ENSMU	446	-68.3	153	-27.1	250	1114	952
mmu-r	ENSMU	446	-66.9	150	-24.3	9523	5741	10192
mmu-r	ENSMU	446	-66.3	154	-22.6	5957	33	4299
mmu-r	ENSMU	446	-66	157	-27.2	7852	6547	7448
mmu-r	ENSMU	446	-63.8	159	-23.3	85	1220	1968
mmu-r	ENSMU	446	-62.4	151	-23.1	1263	1334	1167
mmu-r	ENSMU	446	-60.7	151	-21.5	2759	5457	5357
mmu-r	Gm388	446	-60.5	153	-21.6	6494	259	4066
mmu-r	ENSMU	446	-60.3	155	-23.1	9227	1563	10733
mmu-r	ENSMU	446	-58.7	153	-21	6571	9564	3825
mmu-r	ENSMU	446	-58	154	-21	3433	4868	8360
mmu-r	Gm388	446	-57.5	158	-21.9	7285	2148	457
mmu-r	ENSMU	446	-57.3	151	-21.8	4289	4340	2047
mmu-r	ENSMU	446	-54.6	152	-22.2	2138	4155	9455
mmu-r	Gm388	446	-53.2	153	-20.6	3659	1246	4358
mmu-r	Gm388	446	-52.4	151	-21.1	1098	3395	2580
mmu-r	ENSMU	446	-48.8	155	-17.4	3832	1570	5068
mmu-r	ENSMU	446	-46.5	162	-22.2	3368	13872	11122
mmu-r	ENSMU	446	-44.4	153	-22.1	239	8080	1713
mmu-r	ENSMU	446	-39.4	156	-17	8696	2081	5972
mmu-r	Gm388	446	-38	151	-14.5	3724	4629	1187
mmu-r	ENSMU	445	-67.3	153	-26.5	2349	3143	2761
mmu-r	ENSMU	445	-62.4	154	-25.9	3470	1695	1138
mmu-r	ENSMU	445	-60.2	158	-23	8143	2628	1193
mmu-r	ENSMU	445	-60.2	159	-24.6	9773	3179	10145
mmu-r	ENSMU	445	-57.1	150	-20.9	6794	9083	7452
mmu-r	Gm388	445	-56.5	151	-20.4	7286	2143	457
mmu-r	ENSMU	445	-56.2	151	-22.1	11257	3628	4597
mmu-r	ENSMU	445	-54.9	150	-20.5	683	8922	8279
mmu-r	ENSMU	445	-52.8	155	-21.5	7341	8130	10943
mmu-r	ENSMU	445	-52	153	-19.9	448	3594	6958
mmu-r	ENSMU	445	-51.9	151	-19.5	19	6826	8654
mmu-r	ENSMU	445	-51.1	159	-20.9	2669	7566	3191
mmu-r	ENSMU	445	-49.6	152	-19.9	4237	1055	5626
rco-mil	ENSMU	445	-44.6	155	-15.9	713	4336	4881
mmu-r	ENSMU	445	-43	152	-16.8	10417	8971	6731
mmu-r	ENSMU	445	-42.4	151	-16.4	9089	988	9606
mmu-r	ENSMU	445	-38.6	161	-17.2	9654	245	8440
mmu-r	ENSMU	445	-38.1	159	-19.5	2593	6059	8945
mmu-r	Gm388	445	-36.4	156	-13.1	3658	6810	8454
mmu-r	ENSMU	445	-36.1	161	-15.5	11534	9162	7239
mmu-r	ENSMU	444	-64.7	155	-22.8	9555	10129	10538
mmu-r	ENSMU	444	-61.1	155	-24.5	7158	7765	3677
mmu-r	ENSMU	444	-60.6	160	-25.1	12422	6603	2758
mmu-r	Gm388	444	-59.3	152	-22.8	8234	8495	7282
mmu-r	Gm388	444	-57.5	151	-26	6940	7629	3767
mmu-r	Gm412	444	-53.6	154	-18.9	899	1453	2693
mmu-r	ENSMU	444	-53.1	152	-19.5	2209	5938	4197
mmu-r	ENSMU	444	-51.7	157	-22.1	930	5463	1047
mmu-r	Gm388	444	-48.4	151	-18.2	2143	455	7285
mmu-r	ENSMU	444	-43.6	157	-15.7	685	8920	8280
mmu-r	Gm388	444	-40.2	160	-16.4	1186	3723	4629
mmu-r	ENSMU	444	-35.1	148	-12.8	2483	2521	2559
mmu-r	ENSMU	444	-31	153	-14.7	12063	8346	4822
mmu-r	Gm388	443	-64.4	154	-22.9	8624	4290	1737
mmu-r	ENSMU	443	-62.8	158	-26.2	9127	1688	3565
rco-mil	Gm388	443	-61.6	151	-25.3	5472	7647	7806
rco-mil	Gm388	443	-61.6	151	-25.3	5472	7647	7806
rco-mil	Gm388	443	-61.6	151	-25.3	5472	7647	7806
mmu-r	ENSMU	443	-59.8	153	-20.2	1469	4915	8407
rco-mil	Gm388	443	-59.5	151	-22.8	5472	7647	7806
mmu-r	ENSMU	443	-59.4	152	-20.3	435	3706	3869

mmu-r	ENSMU	443	-57.2	156	-21.9	9660	6504	859
mmu-r	ENSMU	443	-56.2	153	-19.8	4208	1133	1064
mmu-r	ENSMU	443	-51.7	150	-19.7	5025	10357	5761
mmu-r	ENSMU	443	-47.5	157	-23.2	11280	7768	12306
mmu-r	ENSMU	443	-47.3	152	-18.5	11907	11009	10281
mmu-r	ENSMU	443	-47.3	159	-19.3	3420	5930	3394
mmu-r	ENSMU	443	-44.7	161	-16.1	838	14269	2428
mmu-r	ENSMU	443	-44.6	149	-15.6	7365	7864	2259
mmu-r	ENSMU	443	-42.7	149	-17.8	9199	10095	5848
mmu-r	ENSMU	443	-42.1	151	-16.3	8231	3406	3645
mmu-r	ENSMU	443	-41.6	150	-16.5	7486	709	9734
mmu-r	ENSMU	443	-37.5	152	-18.4	8826	5342	6056
mmu-r	ENSMU	443	-36.4	163	-17.9	9687	3099	11628
mmu-r	ENSMU	443	-27.2	157	-10.6	2552	1492	4792
mmu-r	ENSMU	442	-67	157	-26.1	7856	6552	7451
mmu-r	ENSMU	442	-64.8	154	-23.7	2713	12748	10001
mmu-r	Gm388	442	-59.7	150	-21	3578	1437	1015
mmu-r	ENSMU	442	-54.8	160	-19.4	3933	2643	5992
mmu-r	ENSMU	442	-53.4	150	-18.5	2897	4871	8363
mmu-r	Gm388	442	-48	150	-17.3	7290	457	2146
mmu-l	ENSMU	442	-46.2	160	-18.4	1477	7218	7884
mmu-r	ENSMU	442	-43.7	154	-17.3	6000	5843	8290
mmu-r	ENSMU	442	-42.3	162	-19.1	918	1911	2133
mmu-r	ENSMU	442	-35.3	150	-12.8	9530	4591	3389
mmu-r	ENSMU	442	-32.5	152	-13.7	3179	502	5032
mmu-r	ENSMU	441	-67.4	150	-24.9	6166	7909	8060
mmu-r	ENSMU	441	-63.3	159	-27.5	10506	12092	1976
mmu-r	ENSMU	441	-63	149	-23.5	461	6742	1424
mmu-r	Gm388	441	-61	157	-28.5	5060	8809	3136
mmu-r	ENSMU	441	-60.6	152	-21.6	3776	9533	7781
mmu-r	ENSMU	441	-58.1	156	-20.8	3792	1578	5821
mmu-r	ENSMU	441	-57.8	154	-20.9	7541	4904	2512
mmu-r	ENSMU	441	-55.8	152	-19.8	6326	5312	877
mmu-r	ENSMU	441	-54.3	151	-21.8	1551	4489	11053
mmu-r	ENSMU	441	-52.8	150	-18.5	8632	5342	10209
mmu-r	ENSMU	441	-49.8	152	-20.1	4238	1056	5627
mmu-r	ENSMU	441	-49.6	154	-18.5	2908	1886	1734
mmu-r	ENSMU	441	-48.5	152	-18.5	12200	2977	8618
mmu-r	ENSMU	441	-47.1	152	-19.7	4833	12042	11601
mmu-r	ENSMU	441	-46.5	148	-16.2	1249	8899	2917
mmu-r	ENSMU	441	-45.8	156	-16.5	3213	2652	2672
mmu-r	Gm388	441	-45.1	156	-18.9	5249	6274	1
mmu-r	ENSMU	441	-45	149	-15.4	6565	6572	1907
mmu-r	ENSMU	441	-44.6	149	-21.9	6686	10674	3225
mmu-r	ENSMU	441	-40.6	150	-15.4	3609	4250	3473
mmu-r	ENSMU	441	-34.4	150	-12.8	9529	4590	3382
mmu-r	ENSMU	441	-31.5	153	-13.2	11440	1276	8306
mmu-r	ENSMU	440	-61	150	-21.7	2898	6598	11681
mmu-r	ENSMU	440	-60.4	149	-22.1	1577	1529	11059
mmu-r	ENSMU	440	-60.4	147	-22.3	5341	11104	10471
mmu-r	ENSMU	440	-60.3	153	-26.5	900	8226	3705
mmu-r	ENSMU	440	-58	150	-23.8	6671	4192	11529
mmu-r	Gm388	440	-56.8	154	-23.3	7286	2146	461
mmu-r	ENSMU	440	-53.1	152	-21.6	3755	1244	5996
mmu-r	ENSMU	440	-53	153	-28.4	300	6137	3582
mmu-r	ENSMU	440	-52.8	150	-20.2	2656	13587	8385
mmu-r	Gm412	440	-47.6	160	-18.8	413	2257	4023
mmu-r	ENSMU	440	-47.4	152	-16.9	3546	689	8969
mmu-r	ENSMU	440	-42.7	153	-15.9	9090	9605	987
mmu-r	ENSMU	440	-42.7	153	-15.9	9090	9605	987
mmu-r	ENSMU	440	-42.6	153	-15.9	9090	9605	987
mmu-r	ENSMU	440	-39.6	151	-15.1	376	10143	4655
mmu-r	ENSMU	440	-37.7	150	-14.6	4276	433	4655
mmu-r	ENSMU	440	-37.1	151	-17.5	5404	5552	698

mmu-r	ENSMU	440	-35.1	154	-12.8	7036	6603	2688
mmu-r	ENSMU	440	-32.1	160	-14.8	9687	3099	11628
mmu-r	ENSMU	439	-59.4	158	-23.6	7394	390	13901
mmu-r	ENSMU	439	-56.4	156	-22.3	7047	7496	5334
mmu-r	ENSMU	439	-54.6	153	-19.2	1245	5998	3753
mmu-r	ENSMU	439	-53.9	155	-18.4	100	3735	13416
mmu-r	ENSMU	439	-49.5	149	-19.4	440	10795	413
mmu-r	ENSMU	439	-48.2	155	-17.2	10502	1930	4071
mmu-r	Gm388	439	-48	150	-17.9	8094	8865	1298
mmu-r	ENSMU	439	-44	150	-18.1	8437	7066	2746
mmu-r	ENSMU	439	-41.4	151	-16.1	2241	2776	7275
mmu-r	ENSMU	439	-40.7	151	-15	1128	841	1056
mmu-r	ENSMU	439	-37.4	153	-15.3	5178	2163	527
mmu-r	ENSMU	438	-67.6	151	-27.5	661	4797	8182
mmu-r	ENSMU	438	-62.6	151	-23.5	6549	7450	7857
mmu-r	ENSMU	438	-61.4	148	-25.8	2621	4441	3604
mmu-r	ENSMU	438	-61.3	150	-21.3	5111	1336	7060
mmu-r	ENSMU	438	-54.1	149	-19.1	5759	5024	10356
mmu-r	ENSMU	438	-49.8	148	-16.6	6133	7690	7774
mmu-r	ENSMU	438	-47.5	151	-20.5	8260	14111	2898
mmu-r	ENSMU	438	-47.2	154	-18.2	146	3849	1567
mmu-r	ENSMU	438	-45.6	151	-19.3	2757	12422	6602
mmu-r	ENSMU	438	-44	151	-19.1	13084	683	1986
mmu-r	ENSMU	438	-43.5	149	-18.3	3647	5701	4650
rco-mil	ENSMU	438	-37.9	155	-14.8	605	5022	4307
mmu-r	ENSMU	438	-36.5	153	-13.5	9686	3098	11628
mmu-r	Gm388	437	-65.5	151	-24	2230	7366	8486
mmu-r	ENSMU	437	-60.9	155	-22.7	5870	12674	10133
mmu-r	Gm388	437	-60.6	152	-22.4	25	2658	1
mmu-r	Gm388	437	-57	151	-23.7	2275	1338	3441
mmu-r	ENSMU	437	-54.3	156	-20.1	4301	11414	5049
mmu-r	Gm388	437	-54.3	149	-22	3271	1976	709
mmu-r	ENSMU	437	-52.6	150	-22.5	776	3793	1118
mmu-r	ENSMU	437	-50.7	157	-23.3	364	6308	11475
mmu-r	Gm388	437	-50	154	-23.2	8518	3394	2017
mmu-r	Gm412	437	-49.5	149	-20.6	4024	2254	414
mmu-r	ENSMU	437	-47.5	152	-17.6	6387	631	776
mmu-r	ENSMU	437	-46.4	151	-17.4	876	6326	5317
mmu-r	ENSMU	437	-44.9	151	-16.4	10257	8975	3526
mmu-r	ENSMU	437	-43.9	149	-17.3	2590	4237	8035
mmu-r	ENSMU	437	-36.8	149	-16.1	4774	12792	3753
mmu-r	ENSMU	437	-33.4	149	-14	7275	2241	2776
mmu-r	ENSMU	437	-28.9	151	-11.5	9655	249	8441
mmu-r	Gm388	436	-62.8	148	-24.6	3132	8806	7935
mmu-r	ENSMU	436	-60.9	148	-21.9	6543	10369	10022
mmu-r	ENSMU	436	-58.1	150	-25.8	5335	6346	166
mmu-r	ENSMU	436	-56.1	148	-21	14416	5486	7878
mmu-r	Gm412	436	-53.4	151	-23	4356	4057	2542
mmu-r	ENSMU	436	-51	150	-22.2	10214	8636	5344
mmu-r	ENSMU	436	-49.4	147	-17.6	4653	2259	3209
mmu-r	ENSMU	436	-48.3	154	-17.2	6290	5435	7481
mmu-r	ENSMU	436	-42.1	146	-16.7	3471	174	3127
mmu-r	ENSMU	436	-38.2	149	-14.4	3503	1976	3700
mmu-r	ENSMU	436	-32	152	-13.3	935	2975	12860
mmu-r	ENSMU	435	-66.9	148	-25.3	2414	2500	3245
mmu-r	Gm412	435	-54.2	146	-20	410	4023	2253
mmu-r	ENSMU	435	-53.5	148	-26.3	1698	7479	2129
mmu-r	ENSMU	435	-51.7	152	-21.1	3867	3705	432
mmu-r	ENSMU	435	-46.2	147	-18	5364	604	4732
mmu-r	ENSMU	435	-35.9	150	-15.7	4494	1807	4194
mmu-r	ENSMU	435	-29.4	151	-10.9	12476	11874	10303
mmu-r	ENSMU	434	-65.4	147	-22.9	1956	3239	775
mmu-r	ENSMU	434	-57.2	151	-20	4654	1025	831
mmu-r	ENSMU	434	-56.5	154	-22.1	10732	1563	9225

mmu-r	Gm388	434	-53.8	147	-21.2	7256	6891	127
mmu-r	Gm388	434	-49.9	147	-19.1	3994	2910	5138
mmu-r	ENSMU	434	-47.1	149	-17.3	2590	4237	8034
mmu-r	ENSMU	434	-46.5	145	-21.4	4776	7849	4719
mmu-r	ENSMU	434	-34.1	146	-12.1	1507	10522	10664
mmu-r	ENSMU	434	-33.9	152	-15.3	10732	1561	9223
mmu-r	Gm412	434	-31.8	149	-12.8	134	2916	2511
mmu-r	Gm388	433	-57.7	146	-22.1	8518	3390	2017
mmu-r	ENSMU	433	-56.2	153	-20.6	6899	605	2644
mmu-r	ENSMU	433	-55.3	146	-20.1	8204	10969	376
mmu-r	ENSMU	433	-49.7	153	-21.7	7863	6465	9628
mmu-r	ENSMU	433	-43	149	-18.7	4297	5255	5422
mmu-r	ENSMU	433	-37.1	147	-13.6	11052	1560	1041
mmu-r	Gm388	433	-26.4	147	-9.37	6730	2585	872
mmu-r	ENSMU	432	-75.2	145	-30.5	3432	3740	6289
mmu-r	ENSMU	432	-62.4	146	-23.2	1349	7538	9320
mmu-r	ENSMU	432	-56.8	147	-20.9	8993	1465	1975
mmu-r	Gm388	432	-52.7	150	-18.1	8540	6091	7485
mmu-r	Gm388	432	-52	150	-18.1	8541	6092	7486
mmu-r	Gm388	432	-48.4	148	-17.6	455	7288	2146
mmu-r	ENSMU	432	-42.6	145	-15.4	1662	12332	4546
mmu-r	ENSMU	432	-40.4	149	-14.4	5812	527	30
mmu-r	ENSMU	432	-38.3	150	-15.6	12858	2976	934
mmu-r	ENSMU	432	-32.6	147	-14.1	4740	10421	12986
mmu-r	ENSMU	432	-31.8	147	-13.4	9164	11468	12705
mmu-r	ENSMU	431	-57.6	145	-21.5	10040	793	4892
mmu-r	ENSMU	431	-50.4	149	-19.3	11243	11318	6358
mmu-r	ENSMU	431	-47.9	145	-19	10394	5075	7677
mmu-r	ENSMU	431	-46.2	146	-17.5	5518	3662	257
mmu-r	ENSMU	431	-44	147	-17.1	3962	537	4614
mmu-r	ENSMU	431	-43.9	147	-17.4	8468	10192	14143
mmu-r	ENSMU	431	-42.8	149	-15.8	2897	4871	8363
mmu-r	ENSMU	431	-42.7	147	-16.3	8468	14143	10191
mmu-r	Gm412	431	-42.5	149	-14.5	2000	610	4107
mmu-r	ENSMU	431	-42.1	147	-16.3	8048	1914	6571
mmu-r	ENSMU	431	-41	145	-15.2	2915	8895	1249
mmu-r	ENSMU	431	-32.5	149	-16.5	2897	4873	8365
mmu-r	ENSMU	430	-60.3	148	-22.8	7977	11300	9329
mmu-r	ENSMU	430	-58	146	-23.2	9875	4600	2898
mmu-r	ENSMU	430	-51.8	150	-19.1	3915	434	6678
mmu-r	ENSMU	430	-49.2	145	-21.8	5329	8067	5216
mmu-r	ENSMU	430	-47.9	150	-18	4325	9196	10218
mmu-r	ENSMU	430	-43.5	150	-16.1	793	5028	10321
mmu-r	ENSMU	430	-36.5	148	-20	2680	502	7189
mmu-r	ENSMU	430	-27.8	145	-9.71	11468	9164	12706
mmu-r	ENSMU	429	-53.9	148	-22	8273	10828	595
mmu-r	ENSMU	429	-50.7	147	-19.1	7449	6551	7857
mmu-r	ENSMU	429	-50.5	146	-18.9	1244	3752	5996
mmu-r	ENSMU	429	-50	149	-23.4	620	283	11221
mmu-r	ENSMU	429	-47.2	145	-17.6	13639	3780	7675
mmu-r	ENSMU	429	-30.7	143	-11	2480	2518	2556
mmu-r	ENSMU	429	-30.3	145	-13.1	325	4980	7692
mmu-r	ENSMU	428	-56.9	146	-22.1	2125	4041	712
mmu-r	ENSMU	428	-51.2	145	-19.5	7448	6549	7855
mmu-r	Gm388	428	-49.4	146	-18.3	4368	5134	3575
mmu-r	ENSMU	428	-46.5	146	-21.1	774	2115	633
mmu-r	Gm388	428	-45	146	-15.2	460	7289	2147
mmu-r	ENSMU	428	-42	146	-14.5	4397	2020	3194
mmu-r	ENSMU	428	-40.5	148	-14.2	11655	7489	12393
mmu-r	ENSMU	428	-38.4	147	-16.1	9145	13332	4795
mmu-r	ENSMU	428	-37.8	146	-13.7	5050	9832	5086
mmu-r	ENSMU	427	-40.1	147	-15	5601	6604	9490
mmu-r	ENSMU	427	-28.5	144	-11.6	11700	13823	2142
mmu-r	ENSMU	427	-22.3	147	-8.82	11568	8806	12344

mmu-r	ENSMU	426	-52.5	146	-20.2	6144	4911	8403
mmu-r	ENSMU	426	-47.8	145	-18.6	9700	2500	83
mmu-r	ENSMU	426	-43.3	144	-16.6	3553	3063	2210
mmu-r	ENSMU	426	-40.2	144	-14.9	10497	2930	4621
mmu-r	Gm412	426	-37.6	144	-14	1061	1076	2607
mmu-r	Gm388	425	-60.7	145	-22.1	2017	260	6494
mmu-r	ENSMU	425	-55.4	143	-19.2	7978	11301	9330
mmu-r	ENSMU	425	-43.5	143	-17.9	47	1248	4558
mmu-r	ENSMU	424	-45.8	142	-17.7	4354	7863	10349
mmu-r	ENSMU	424	-43.1	142	-17.7	5553	13738	8789
mmu-r	ENSMU	424	-41.9	142	-17.7	2327	5708	4167
mmu-r	ENSMU	424	-41.6	143	-15	2496	83	9700
mmu-r	Gm388	424	-34	142	-11.8	98	4521	795
mmu-r	ENSMU	424	-31.1	144	-13.5	1072	751	1366
mmu-r	ENSMU	424	-30.3	144	-11.7	7536	6981	7776
mmu-r	Gm388	423	-73.6	142	-30.8	30	6928	1
mmu-r	ENSMU	423	-35.1	141	-13.3	2897	4871	8363
mmu-r	ENSMU	423	-32.1	143	-11.5	2897	4872	8364
mmu-r	ENSMU	422	-71.9	141	-26.3	3843	5402	6421
mmu-r	ENSMU	422	-44	141	-16.4	4871	8363	2897
mmu-r	ENSMU	422	-38.8	141	-13.3	4729	6622	8702
mmu-r	Gm412	421	-32.2	141	-11.5	1064	1081	2612
mmu-r	ENSMU	421	-29.5	141	-11.2	4980	441	3355
mmu-r	ENSMU	420	-32.8	140	-11.7	2898	4872	8364
mmu-r	ENSMU	371	-41.6	188	-21.5	3637	3653	
mmu-r	ENSMU	360	-64.6	180	-32.3	4911	8403	
mmu-r	ENSMU	359	-57	188	-35.4	4124	3966	
mmu-r	ENSMU	358	-82.9	185	-45	5404	6419	
mmu-r	ENSMU	356	-52.5	189	-33.9	4123	3965	
mmu-r	ENSMU	356	-52.5	189	-33.9	4123	3965	
mmu-r	ENSMU	356	-52.5	189	-33.9	4123	3965	
mmu-r	ENSMU	344	-66.3	173	-37.1	4422	1167	
mmu-r	ENSMU	343	-70.2	174	-36.6	3389	3342	
mmu-r	ENSMU	337	-57.3	171	-31.4	14384	6200	
mmu-r	ENSMU	337	-40.5	174	-23.6	4698	5691	
mmu-r	Gm388	337	-29.8	170	-16	854	6436	
mmu-r	ENSMU	336	-48.8	175	-26.8	5331	6347	
mmu-r	ENSMU	334	-59.2	170	-29.6	6850	3385	
mmu-r	Gm388	333	-42.4	169	-21.6	9066	3478	
mmu-r	ENSMU	333	-40.8	181	-23.2	9694	10049	
mmu-r	ENSMU	333	-36.1	179	-25.4	1243	6075	
mmu-r	Gm412	332	-46	171	-23.2	1441	526	
mmu-r	ENSMU	332	-32.5	175	-20.1	6829	3492	
mmu-r	ENSMU	331	-58	169	-29.5	789	2641	
mmu-r	ENSMU	330	-32.5	183	-21.8	6819	12268	
mmu-r	Gm388	330	-27.7	173	-16.3	3237	3212	
mmu-r	Gm388	330	-27.7	173	-16.3	3237	3212	
mmu-r	ENSMU	329	-53.4	174	-29.6	1690	5101	
mmu-r	ENSMU	329	-33.1	165	-18.3	1144	3896	
mmu-r	ENSMU	328	-62.5	180	-38.6	1341	1592	
mmu-r	ENSMU	328	-52	167	-26.4	2629	7190	
mmu-r	ENSMU	328	-51.9	168	-27.9	1314	2255	
mmu-r	Gm412	328	-41.6	178	-29.7	4091	4542	
mmu-r	ENSMU	328	-41.1	174	-27.6	7170	6846	
mmu-r	Gm388	328	-33.5	168	-20.1	3908	4983	
mmu-r	ENSMU	328	-32.8	170	-20.6	12310	7796	
mmu-r	ENSMU	327	-52	168	-29.4	2405	3685	
mmu-r	ENSMU	327	-47.1	171	-31.2	11061	11249	
mmu-r	ENSMU	327	-40.3	171	-20.6	2460	4892	
mmu-r	ENSMU	327	-40.1	166	-21.6	702	2115	
mmu-r	ENSMU	326	-51.4	169	-27.9	9424	10458	
mmu-r	ENSMU	326	-48.5	186	-34.1	4474	13290	
mmu-r	ENSMU	326	-47.5	177	-28.1	1356	787	
mmu-r	ENSMU	326	-28.5	164	-15.6	196	10127	

mmu-r	Gm388	325	-58.3	163	-33.4	8240	1121
mmu-r	ENSML	325	-54.3	168	-29.5	721	12100
mmu-r	Gm388	325	-46.4	169	-25.8	2279	83
mmu-r	Gm388	325	-38.8	170	-25.1	2193	8285
mmu-r	ENSML	325	-36.4	175	-24.5	1243	6075
mmu-r	Gm412	325	-35.8	163	-18.1	339	4576
mmu-r	ENSML	324	-54.2	181	-33	3706	5875
mmu-r	ENSML	324	-40.1	167	-22.1	344	1559
mmu-r	ENSML	324	-36	175	-25.4	1243	6077
mmu-r	Gm388	324	-34.8	184	-24.6	894	1652
mmu-r	Gm412	324	-27.9	165	-16.2	1491	1888
mmu-r	ENSML	324	-27.6	165	-16.7	6831	3490
mmu-r	ENSML	323	-51.7	168	-27	5867	1839
mmu-r	ENSML	323	-48.9	166	-24.5	7812	11005
mmu-r	Gm412	323	-46.3	166	-26.5	2426	3061
mmu-r	Gm388	323	-43.7	170	-25.7	314	240
mmu-r	Gm388	323	-43.7	170	-25.7	314	240
mmu-r	Gm388	323	-43.6	170	-25.6	314	240
mmu-r	ENSML	323	-41.4	165	-22.3	4797	3155
mmu-r	ENSML	323	-35	163	-21	7816	14072
mmu-r	Gm412	322	-60.4	166	-32.2	4485	4055
mmu-r	ENSML	322	-38.1	167	-21.1	2357	42
mmu-r	Gm412	322	-37.6	165	-19.8	223	2013
mmu-r	Gm388	322	-37.5	163	-19.5	1175	3841
mmu-r	ENSML	321	-51.1	168	-28	139	35
mmu-r	Gm388	321	-47.6	166	-26.4	4334	6094
mmu-r	Gm388	321	-45.6	166	-23.3	715	8119
mmu-r	ENSML	321	-45	161	-23.6	4042	8797
mmu-r	ENSML	321	-42.5	169	-28.4	4254	3279
mmu-r	ENSML	321	-41	166	-25.5	3155	4799
mmu-r	ENSML	321	-40.3	164	-24	3683	1209
mmu-r	ENSML	321	-31.7	172	-18.4	10680	2932
mmu-r	Gm388	320	-52	166	-27.4	6315	7081
mmu-r	ENSML	320	-49	166	-25.7	3713	5876
mmu-r	ENSML	320	-46	165	-25.5	1051	12236
mmu-r	Gm412	320	-44.2	163	-23.4	4160	4275
mmu-r	Gm412	320	-42.9	170	-22.7	4289	1120
mmu-r	ENSML	320	-42.8	164	-26.6	2627	7427
mmu-r	ENSML	320	-38.2	169	-23.2	11405	6197
mmu-r	ENSML	320	-34.5	174	-23.6	11872	1530
mmu-r	ENSML	320	-34.3	171	-22.7	7952	8721
mmu-r	ENSML	320	-32.3	165	-18.1	2233	3143
mmu-r	ENSML	320	-30.7	160	-16.2	8260	10108
mmu-r	ENSML	320	-28.6	162	-17.9	1981	6240
mmu-r	ENSML	319	-53.7	163	-28.1	3937	587
mmu-r	ENSML	319	-51.4	164	-28.5	2700	2376
mmu-r	ENSML	319	-48	166	-30.1	3743	1129
mmu-r	ENSML	319	-44.4	161	-22.8	1299	3526
mmu-r	ENSML	319	-43.3	163	-22.6	1128	975
mmu-r	Gm412	319	-40.5	165	-24.3	873	2754
mmu-r	ENSML	319	-40	166	-20.8	1912	6080
mmu-r	ENSML	319	-39	166	-23.5	1009	1635
mmu-r	Gm388	319	-24.5	168	-13.1	3208	278
mmu-r	Gm388	318	-54.9	163	-29	8243	1120
mmu-r	ENSML	318	-51.7	160	-28.3	9580	1587
mmu-r	ENSML	318	-49.7	163	-25.6	10472	5341
mmu-r	ENSML	318	-49.1	160	-25	1345	1357
mmu-r	Gm388	318	-47.7	169	-26.5	6312	7078
mmu-r	Gm388	318	-46.5	159	-27.9	1750	4450
mmu-r	ENSML	318	-45.9	175	-28.6	293	4235
mmu-r	ENSML	318	-42.5	167	-24.6	311	4047
mmu-r	ENSML	318	-41.8	163	-21.3	13886	8692
mmu-r	ENSML	318	-41.3	162	-20.8	7204	9049
mmu-r	ENSML	318	-40.4	162	-20.9	1872	3077

mmu-r	Gm412	318	-40	165	-24.1	1436	3192
mmu-r	Gm388	318	-38.3	160	-19.2	2360	5403
mmu-r	ENSMU	318	-38	166	-23	5637	5610
mmu-r	ENSMU	318	-36.7	161	-18.6	1914	6081
mmu-r	ENSMU	318	-34.4	168	-17.6	881	7435
mmu-r	ENSMU	318	-33	160	-18.7	1940	2405
mmu-r	ENSMU	317	-54.9	169	-33.9	3742	4767
mmu-r	ENSMU	317	-51.5	163	-27.3	2487	2451
mmu-r	ENSMU	317	-50.9	162	-28.2	3999	963
mmu-r	ENSMU	317	-49.8	163	-26.3	4002	963
mmu-r	Gm388	317	-49.3	172	-30.2	3405	8997
mmu-r	Gm388	317	-48.7	160	-26.3	4463	2233
mmu-r	ENSMU	317	-48.6	162	-25.3	2113	700
mmu-r	ENSMU	317	-47.9	159	-25.6	1329	7584
mmu-r	ENSMU	317	-44.6	164	-27.8	5798	1372
mmu-r	ENSMU	317	-44.5	175	-33	13545	12423
mmu-r	ENSMU	317	-44.5	161	-23.5	11315	6158
mmu-r	Gm388	317	-44	161	-22.4	315	241
mmu-r	ENSMU	317	-42.3	171	-25.7	2610	760
rco-mil	Gm412	317	-39.7	169	-21.7	3790	2662
rco-mil	Gm412	317	-39.7	169	-21.7	3790	2662
rco-mil	Gm412	317	-39.7	169	-21.7	3790	2662
rco-mil	Gm412	317	-39.7	169	-21.7	3790	2662
mmu-r	ENSMU	317	-38.8	166	-21.6	9396	8637
mmu-r	ENSMU	317	-37.7	170	-23.2	3237	5940
mmu-r	Gm388	317	-28.3	166	-16.8	3206	1687
mmu-r	ENSMU	317	-24.9	170	-16.5	9096	4784
mmu-r	ENSMU	316	-56	162	-28.2	178	689
mmu-r	ENSMU	316	-48.6	161	-25.2	10235	3677
mmu-r	ENSMU	316	-47.7	159	-25.6	2498	924
mmu-r	ENSMU	316	-46.7	163	-26.7	13891	8276
mmu-r	ENSMU	316	-40	164	-21.2	2011	3631
mmu-r	Gm388	316	-39.8	162	-21.9	4572	7377
mmu-r	ENSMU	316	-38.7	174	-24.7	1338	2717
mmu-r	ENSMU	316	-38.1	170	-24.7	5969	5412
mmu-r	Gm388	316	-35.3	163	-18	6232	6626
mmu-r	Gm388	316	-34.6	159	-19	3882	1655
mmu-r	ENSMU	316	-33.4	161	-17.8	789	6962
mmu-r	ENSMU	316	-31.5	158	-16.8	301	2603
mmu-r	ENSMU	316	-30.6	167	-16.3	8230	5727
mmu-r	ENSMU	316	-30.4	168	-19.3	1244	6078
mmu-r	ENSMU	316	-28.6	160	-16.4	3035	2720
mmu-r	Gm388	315	-60.3	163	-31.3	7480	6088
mmu-r	ENSMU	315	-59.4	162	-30	3749	422
mmu-r	ENSMU	315	-56	164	-28.4	7535	7188
mmu-r	ENSMU	315	-50.2	166	-27.4	643	5061
mmu-r	ENSMU	315	-49	175	-32	324	10274
mmu-r	ENSMU	315	-48.3	163	-24.2	8669	3336
mmu-r	ENSMU	315	-47	160	-24.9	4642	524
mmu-r	ENSMU	315	-45.7	158	-24.6	4515	1230
mmu-r	ENSMU	315	-45.7	166	-27.2	2191	3188
mmu-r	ENSMU	315	-43.9	163	-22.5	8076	6609
mmu-r	ENSMU	315	-43.4	171	-29.1	11061	11249
rco-mil	ENSMU	315	-43.2	165	-25.8	3244	3351
rco-mil	ENSMU	315	-43.2	165	-25.8	3244	3351
rco-mil	ENSMU	315	-43.2	165	-25.8	3244	3351
mmu-r	Gm388	315	-41.7	163	-24.9	5401	2360
mmu-r	Gm388	315	-40.7	166	-25.4	307	5062
mmu-r	ENSMU	315	-39.5	158	-20.2	8031	8199
mmu-r	ENSMU	315	-37.9	166	-19	4653	10694
mmu-r	ENSMU	315	-34.2	168	-23	11443	7287
mmu-r	ENSMU	315	-31.5	168	-17.5	9915	3419
mmu-r	ENSMU	315	-31.5	164	-17.5	1519	3594
mmu-r	Gm388	315	-31.3	159	-16.6	1385	3322

mmu-r	Gm412	315	-31.1	159	-21.4	1015	3349
mmu-r	Gm388	315	-30.1	171	-18.6	3236	5314
mmu-r	ENSMU	315	-28.3	160	-17.2	12269	6822
mmu-r	ENSMU	315	-26.4	161	-14	13808	9746
mmu-r	Gm388	315	-22.8	163	-12.8	3239	3214
mmu-r	Gm388	314	-51	167	-30	1340	3738
mmu-r	ENSMU	314	-50.7	174	-29.6	8898	5291
mmu-r	ENSMU	314	-49.8	168	-30.7	1707	1470
mmu-r	ENSMU	314	-49.6	159	-28.8	5939	561
mmu-r	Gm388	314	-48.9	168	-28.1	7881	2419
mmu-r	ENSMU	314	-48.4	163	-26.3	54	5269
mmu-r	ENSMU	314	-48.2	165	-27	4136	1793
mmu-r	ENSMU	314	-46.3	161	-24.8	2099	2205
mmu-r	ENSMU	314	-43.4	160	-23.7	5145	1155
mmu-r	ENSMU	314	-43.1	166	-25.7	10192	10890
mmu-r	ENSMU	314	-41.7	159	-24	6869	2741
mmu-r	ENSMU	314	-40.7	159	-21.9	7487	7714
mmu-r	ENSMU	314	-39.9	160	-20.7	87	4124
mmu-r	Gm388	314	-39.6	159	-22	388	4742
rco-mil	ENSMU	314	-39.5	160	-22.1	1021	4498
mmu-r	ENSMU	314	-34.8	158	-20	3155	4795
mmu-r	Gm412	314	-33.9	165	-17.6	57	4384
mmu-r	ENSMU	314	-33.6	158	-17.9	3155	4795
mmu-r	ENSMU	314	-32.4	160	-19	2325	935
mmu-r	Gm412	314	-30.7	162	-16.1	3275	1110
mmu-r	ENSMU	314	-29.6	164	-14.9	44	2358
mmu-r	Gm388	314	-20.1	162	-10.1	6839	7171
mmu-r	ENSMU	313	-62.8	161	-35.9	13506	9032
mmu-r	ENSMU	313	-53.7	158	-28.6	1335	1168
mmu-r	ENSMU	313	-47.2	161	-25.4	97	3221
mmu-r	ENSMU	313	-45.3	173	-33.5	5473	12646
mmu-r	Gm412	313	-44.8	165	-26.1	1942	1338
mmu-r	Gm412	313	-44.8	165	-26.1	1942	1338
mmu-r	Gm412	313	-44.2	165	-25.5	1942	1338
mmu-r	ENSMU	313	-44.1	164	-23.5	9064	2322
mmu-r	ENSMU	313	-44	158	-23.9	8811	63
mmu-r	ENSMU	313	-43.6	172	-26.6	10170	2337
mmu-r	ENSMU	313	-41.3	157	-21.3	3917	435
mmu-r	ENSMU	313	-40.4	168	-24.1	10170	2338
mmu-r	Gm388	313	-39.9	163	-20.4	7106	5229
hiv1-m	ENSMU	313	-39.7	164	-22.9	1624	5437
mmu-r	ENSMU	313	-37.5	163	-21.1	694	3547
mmu-r	Gm412	313	-35.4	161	-18.3	2183	2195
mmu-r	Gm412	313	-35.4	161	-18.3	2182	2194
mmu-r	ENSMU	313	-35	157	-19	2137	1817
mmu-r	ENSMU	313	-34.3	161	-17.4	3699	303
mmu-r	Gm412	313	-33.7	161	-17.6	2182	2194
mmu-r	ENSMU	313	-33.5	159	-16.8	1817	4027
mmu-r	ENSMU	313	-29.8	160	-18.9	10238	6821
mmu-r	ENSMU	313	-22	158	-12.1	7103	7544
mmu-r	ENSMU	312	-67.2	166	-40	7201	6971
mmu-r	ENSMU	312	-54.6	160	-29.9	346	902
mmu-r	Gm388	312	-52.7	158	-28	8244	1120
mmu-r	ENSMU	312	-52.6	165	-31.9	3680	10236
mmu-r	ENSMU	312	-50.5	162	-29.5	9830	6027
mmu-r	Gm388	312	-50.1	159	-25.1	9063	820
mmu-r	Gm388	312	-49.4	167	-29.6	637	7651
mmu-r	ENSMU	312	-49.4	159	-27.5	1732	6219
mmu-r	Gm412	312	-48.9	161	-28.2	570	2555
mmu-r	Gm388	312	-44.9	161	-27	225	4010
mmu-r	Gm388	312	-44.8	159	-22.6	5090	5335
mmu-r	ENSMU	312	-44.1	163	-24.9	3576	475
mmu-r	ENSMU	312	-41.9	166	-27.6	3742	10602
mmu-r	ENSMU	312	-41.9	158	-22.2	8682	292

mmu-r	Gm412	312	-41.8	168	-23.1	4471	3197
mmu-r	Gm388	312	-41.5	160	-23.6	8621	1734
mmu-r	ENSMU	312	-38.6	160	-21	4022	1328
mmu-r	Gm388	312	-38.6	159	-19.8	6592	1335
mmu-r	ENSMU	312	-38.5	157	-19.9	3977	1334
mmu-r	ENSMU	312	-36.7	168	-23.5	1755	2951
mmu-r	ENSMU	312	-36	157	-19	920	5764
mmu-r	ENSMU	312	-35.5	165	-19.5	10997	1978
mmu-r	ENSMU	312	-34.5	159	-18.1	7572	4495
rco-mil	ENSMU	312	-33.8	165	-20.2	10815	12781
rco-mil	ENSMU	312	-31.8	157	-16	10815	12781
rco-mil	ENSMU	312	-31.8	157	-16	10815	12781
rco-mil	ENSMU	312	-31.8	157	-16	10815	12781
rco-mil	ENSMU	312	-31.8	157	-16	10815	12781
mmu-r	ENSMU	312	-29	160	-16.3	2064	5923
mmu-r	Gm388	312	-26.9	163	-15.7	3239	3212
mmu-r	ENSMU	312	-26.7	166	-17.8	5537	6025
mmu-r	ENSMU	312	-24.6	165	-17.1	3373	14
mmu-r	ENSMU	311	-61.7	162	-34.1	9577	794
mmu-r	ENSMU	311	-60.3	160	-31.1	592	112
mmu-r	ENSMU	311	-53.2	165	-33.5	7533	7189
mmu-r	ENSMU	311	-50.7	166	-30.2	8132	7685
mmu-r	ENSMU	311	-50.1	160	-25.7	4607	3736
mmu-r	ENSMU	311	-49.4	161	-27.6	671	1218
mmu-r	ENSMU	311	-45.8	157	-24.6	1520	755
mmu-r	ENSMU	311	-45.1	161	-23.1	7869	2365
mmu-r	ENSMU	311	-42.1	170	-24.8	1338	2720
mmu-r	ENSMU	311	-40.6	161	-21.2	13078	5196
mmu-r	ENSMU	311	-40.3	156	-22.1	3270	1354
mmu-r	ENSMU	311	-39.9	168	-25.7	4498	7746
rco-mil	ENSMU	311	-39.6	156	-20.3	1021	4498
mmu-r	ENSMU	311	-38.8	160	-21.7	5513	11649
mmu-r	ENSMU	311	-38.7	157	-20.9	6412	7576
mmu-r	ENSMU	311	-38.3	166	-22.3	2611	765
mmu-r	ENSMU	311	-37.7	158	-20.9	1912	6080
mmu-r	Gm412	311	-37.2	165	-22.2	872	2755
mmu-r	ENSMU	311	-35.3	159	-17.9	1913	6080
mmu-r	ENSMU	311	-34.8	160	-20.1	3932	551
mmu-r	ENSMU	311	-32.5	158	-16.8	1805	208
mmu-r	ENSMU	311	-31.9	157	-16.2	2854	11725
mmu-r	Gm388	311	-23.3	160	-12.1	3206	276
mmu-r	ENSMU	310	-51.4	156	-27.7	890	1733
mmu-r	ENSMU	310	-48.9	162	-29.6	6837	5799
mmu-r	ENSMU	310	-48.1	166	-25.8	7129	674
mmu-r	ENSMU	310	-47.9	160	-25.6	1324	3234
mmu-r	ENSMU	310	-46.8	159	-24.5	8140	3116
mmu-r	ENSMU	310	-46.4	156	-25.2	10249	10362
mmu-r	ENSMU	310	-46.1	159	-25.8	6422	5407
mmu-r	Gm388	310	-45.5	159	-27.2	8772	7763
mmu-r	ENSMU	310	-45.1	159	-23.8	3956	489
mmu-r	ENSMU	310	-44.3	167	-28.2	13940	2582
mmu-r	Gm388	310	-43.8	156	-23.2	823	1331
mmu-r	Gm388	310	-43.6	159	-23	4010	6067
mmu-r	ENSMU	310	-43.3	167	-29.6	14431	6715
mmu-r	ENSMU	310	-42.2	165	-23.7	568	140
mmu-r	ENSMU	310	-42.1	169	-31.4	14423	10932
mmu-r	Gm412	310	-41.2	157	-21.2	82	4112
mmu-r	Gm388	310	-41	157	-20.6	5296	7050
mmu-r	ENSMU	310	-40.4	165	-24.3	4280	236
mmu-r	ENSMU	310	-39.8	164	-22.2	13267	6912
mmu-r	ENSMU	310	-37.5	160	-22.6	4877	6074
mmu-r	ENSMU	310	-37.2	158	-21.9	392	5203
mmu-r	Gm412	310	-36.9	164	-23.3	305	3317
mmu-r	ENSMU	310	-35.2	158	-18.8	3480	4189

mmu-r	Gm388	310	-34.5	156	-17.9	3383	4568
mmu-r	Gm388	310	-34.4	167	-21.6	2356	5402
mmu-r	Gm412	310	-34	156	-19.1	526	1441
mmu-r	ENSMU	310	-33.5	161	-22.6	2107	6276
mmu-r	ENSMU	310	-32.3	163	-20.7	3107	3204
mmu-r	Gm388	310	-30.6	158	-16	1267	2219
mmu-r	ENSMU	310	-30.6	164	-16.8	1376	5802
mmu-r	ENSMU	310	-30.6	163	-17.6	2799	5477
mmu-r	ENSMU	310	-29.1	167	-18.8	2074	10384
mmu-r	Gm388	310	-28.4	156	-15	6377	562
mmu-r	Gm388	310	-28.4	162	-17.2	1808	4936
mmu-r	ENSMU	309	-56.6	156	-30.8	9580	787
mmu-r	Gm388	309	-52	155	-27.6	8546	5374
mmu-r	ENSMU	309	-50.7	159	-29.1	9577	12100
mmu-r	ENSMU	309	-50.5	158	-27.5	3354	9831
mmu-r	ENSMU	309	-49.3	162	-32.1	352	1820
mmu-r	ENSMU	309	-48.8	157	-26.3	6875	6095
mmu-r	ENSMU	309	-45.5	155	-26	3609	3745
mmu-r	ENSMU	309	-45.1	163	-27.6	4001	965
mmu-r	ENSMU	309	-44.6	157	-23.5	6850	3382
mmu-r	Gm412	309	-44.2	158	-25.3	2584	1864
mmu-r	ENSMU	309	-43.6	164	-26.1	4292	724
mmu-r	ENSMU	309	-43.5	155	-25.7	12335	6480
mmu-r	ENSMU	309	-43.4	162	-23.9	3861	2344
mmu-r	ENSMU	309	-42.7	161	-26.4	5755	11764
mmu-r	ENSMU	309	-41.7	159	-23.6	5872	10994
mmu-r	ENSMU	309	-40.7	169	-24	3364	8034
mmu-r	ENSMU	309	-39.7	157	-20.3	6722	1103
mmu-r	ENSMU	309	-39.3	162	-19.9	5023	1102
mmu-r	ENSMU	309	-38.3	161	-20	13962	2699
mmu-r	Gm388	309	-38.3	158	-19.5	1403	2011
mmu-r	Gm388	309	-37	159	-19.6	1317	648
mmu-r	ENSMU	309	-36.9	160	-20.4	6533	591
mmu-r	ENSMU	309	-36.8	155	-20.6	12551	4712
mmu-r	ENSMU	309	-36.8	159	-20.6	4404	11678
mmu-r	ENSMU	309	-36.4	156	-19.3	966	4004
mmu-r	ENSMU	309	-36.2	159	-23.3	3366	9
mmu-r	ENSMU	309	-34.7	155	-18.9	4216	9062
mmu-r	Gm388	309	-34.7	156	-19	2039	4906
mmu-r	ENSMU	309	-33.3	155	-18.4	4428	2484
mmu-r	ENSMU	309	-33	159	-19.9	4006	12751
mmu-r	ENSMU	309	-32.9	167	-23	3105	9982
mmu-r	ENSMU	309	-32.5	159	-16.5	9147	703
mmu-r	ENSMU	309	-32.3	158	-21.5	2514	4303
mmu-r	ENSMU	309	-32.1	155	-18.6	310	5619
mmu-r	Gm388	309	-30.8	156	-16.3	3919	3098
mmu-r	Gm388	309	-27.5	164	-14.1	3240	5315
mmu-r	ENSMU	309	-26.5	160	-16.4	6832	3491
mmu-r	ENSMU	309	-26.3	164	-15.2	1194	527
mmu-r	ENSMU	309	-23.5	157	-12.5	7915	8205
mmu-r	Gm388	308	-56.3	161	-28.6	831	3402
mmu-r	Gm388	308	-52.5	156	-31.2	8210	6100
mmu-r	ENSMU	308	-51.9	155	-27.9	969	2500
mmu-r	ENSMU	308	-48.9	156	-25.6	1323	4199
mmu-r	ENSMU	308	-48.6	163	-29.4	9119	1686
mmu-r	Gm388	308	-48.4	163	-27.7	271	120
mmu-r	ENSMU	308	-48.4	161	-29.2	7151	3672
mmu-r	ENSMU	308	-47.1	154	-24.7	645	5061
mmu-r	ENSMU	308	-45.9	163	-26.1	3711	5875
mmu-r	ENSMU	308	-45.8	159	-24.4	9420	12505
mmu-r	ENSMU	308	-45.7	160	-24.9	8148	3659
mmu-r	ENSMU	308	-45.4	165	-29.2	2852	5824
mmu-r	ENSMU	308	-43.4	158	-25.4	2227	1589
mmu-r	ENSMU	308	-43.2	162	-26.8	4079	8968

mmu-r	ENSMU	308	-42.8	157	-22.1	1469	1707
mmu-r	Gm412	308	-42.3	163	-26.3	3034	1217
hiv1-m	ENSMU	308	-41.7	154	-21.3	1497	8437
mmu-r	ENSMU	308	-40.9	157	-23	5227	7359
mmu-r	ENSMU	308	-40.1	159	-22	3573	473
mmu-r	Gm388	308	-39.9	161	-24.4	2370	2086
mmu-r	ENSMU	308	-39.2	154	-21.5	3093	8163
mmu-r	Gm388	308	-38.3	155	-19.8	384	3469
mmu-r	ENSMU	308	-37.7	161	-21.6	8964	1402
mmu-r	ENSMU	308	-36.9	161	-21	3954	515
mmu-r	ENSMU	308	-36.8	161	-20.6	292	8682
mmu-r	Gm388	308	-36.7	161	-20.4	7783	7821
mmu-r	ENSMU	308	-36.6	167	-19.8	4021	3488
mmu-r	ENSMU	308	-36.1	155	-18.2	6690	8127
mmu-r	ENSMU	308	-34.8	156	-17.5	11826	5735
mmu-r	ENSMU	308	-34.5	157	-20.1	7798	291
mmu-r	ENSMU	308	-33.2	158	-17.4	324	11037
mmu-r	ENSMU	308	-32.3	161	-17.3	11055	1525
mmu-r	Gm388	308	-32.1	165	-20.5	171	2775
mmu-r	ENSMU	308	-30.9	160	-18.6	6069	10527
mmu-r	ENSMU	308	-30.4	164	-19.4	3064	5118
mmu-r	ENSMU	308	-30	160	-18.7	1244	6078
mmu-r	Gm388	308	-28.6	157	-15.7	3746	4983
mmu-r	ENSMU	308	-27	157	-15.5	7724	2055
mmu-r	ENSMU	308	-25.6	157	-13.8	8260	10105
mmu-r	ENSMU	308	-23.4	159	-12.2	517	10448
mmu-r	ENSMU	307	-58.7	158	-33.1	590	3941
mmu-r	ENSMU	307	-54.9	159	-27.9	175	3818
mmu-r	ENSMU	307	-49.9	162	-28	3233	5934
mmu-r	Gm388	307	-49.9	154	-25.8	399	7707
mmu-r	ENSMU	307	-49.2	156	-26.9	3189	1707
mmu-r	Gm388	307	-47.6	156	-25.5	4890	158
mmu-r	ENSMU	307	-47.2	160	-26.5	8576	4270
mmu-r	ENSMU	307	-46.5	159	-24.8	4004	966
mmu-r	ENSMU	307	-45.1	159	-26.9	7683	8136
mmu-r	ENSMU	307	-44.6	161	-28.5	7735	4529
mmu-r	ENSMU	307	-42.8	154	-24.6	181	2256
mmu-r	ENSMU	307	-42.8	164	-23.5	3334	8670
mmu-r	Gm388	307	-42.4	155	-21.8	8178	7597
mmu-r	ENSMU	307	-42.3	155	-23.1	3548	3059
mmu-r	ENSMU	307	-42	155	-24.9	4119	3604
mmu-r	ENSMU	307	-40.4	167	-28.1	11157	11304
mmu-r	ENSMU	307	-40	157	-23.2	8792	3287
mmu-r	Gm388	307	-39.4	167	-25.6	3972	6924
mmu-r	Gm412	307	-38.6	156	-22.1	1943	1339
mmu-r	ENSMU	307	-38.2	158	-19.3	1912	6080
mmu-r	Gm388	307	-37.8	158	-22.1	6232	6625
mmu-r	ENSMU	307	-37.6	157	-19.4	13081	5199
mmu-r	ENSMU	307	-37.4	161	-18.9	876	2537
mmu-r	ENSMU	307	-37.4	155	-18.9	302	3698
mmu-r	ENSMU	307	-37.2	158	-20	1912	6080
mmu-r	Gm412	307	-37.1	166	-22.3	3571	3637
mmu-r	Gm388	307	-37.1	156	-21.1	7104	5226
mmu-r	Gm388	307	-37	158	-19.3	7241	6970
mmu-r	ENSMU	307	-36.3	154	-18.7	4797	3157
mmu-r	ENSMU	307	-34.3	165	-21.8	10104	13557
mmu-r	ENSMU	307	-34.2	155	-17.3	11705	4451
mmu-r	ENSMU	307	-34.1	154	-19.6	6919	6114
mmu-r	ENSMU	307	-33.8	161	-20.9	2207	5176
mmu-r	ENSMU	307	-33.8	154	-18.9	8637	8768
mmu-r	ENSMU	307	-33.7	154	-18.3	3506	2251
mmu-r	ENSMU	307	-33.4	162	-17.3	2256	181
mmu-r	ENSMU	307	-33	164	-23.9	8907	8934
mmu-r	ENSMU	307	-32.5	165	-20.9	4466	16

mmu-r	ENSMU	307	-32	155	-16	7088	11404
mmu-r	ENSMU	307	-30.7	159	-17.1	6393	6248
mmu-r	ENSMU	307	-30.3	155	-16.8	2026	674
mmu-r	ENSMU	307	-29.4	165	-19.7	1046	3166
mmu-r	ENSMU	307	-28.3	167	-19	5510	2952
mmu-r	ENSMU	307	-27.4	154	-15.1	12001	11079
mmu-r	ENSMU	307	-26.6	156	-17.1	5759	6106
mmu-r	ENSMU	307	-26.5	160	-13.7	11114	7279
mmu-r	ENSMU	307	-25.6	167	-16.7	2783	1378
mmu-r	ENSMU	307	-25.6	155	-13.7	8260	10108
mmu-r	ENSMU	307	-25.2	154	-14.1	1741	6743
mmu-r	Gm388	307	-24	158	-12.3	3235	277
mmu-r	Gm388	307	-21.6	155	-11.7	3239	3214
mmu-r	ENSMU	306	-53.2	154	-28	1710	1304
mmu-r	ENSMU	306	-51.1	159	-29	14036	8192
mmu-r	ENSMU	306	-50.4	166	-34.6	1179	6422
mmu-r	ENSMU	306	-50.4	163	-29.1	1326	446
mmu-r	ENSMU	306	-47	159	-24.1	6943	5054
mmu-r	ENSMU	306	-46.6	158	-28.4	7138	11805
mmu-r	ENSMU	306	-46.3	155	-24.7	4994	4359
mmu-r	Gm412	306	-45.8	157	-25.9	2472	2558
mmu-r	ENSMU	306	-45.6	166	-26.7	10800	7053
mmu-r	ENSMU	306	-45.5	154	-24.1	2128	6025
mmu-r	ENSMU	306	-45	156	-28.6	6	6928
mmu-r	ENSMU	306	-43.7	163	-24.6	7316	9526
mmu-r	Gm388	306	-43.4	161	-26.8	8186	7453
mmu-r	Gm388	306	-43.3	156	-23.6	2056	3099
rco-mil	ENSMU	306	-42.2	158	-23.1	13445	11779
rco-mil	ENSMU	306	-42.2	158	-23.1	13445	11779
mmu-r	ENSMU	306	-41.7	160	-26.5	13532	9692
mmu-r	ENSMU	306	-41.6	157	-22	938	7362
mmu-r	ENSMU	306	-41.5	159	-23.4	1756	2948
mmu-r	ENSMU	306	-40.9	156	-21.6	3434	2124
mmu-r	Gm388	306	-40	157	-21.2	823	9065
mmu-r	ENSMU	306	-39.2	158	-20.7	10310	14097
mmu-r	ENSMU	306	-38.9	166	-22.9	8634	2423
mmu-r	ENSMU	306	-38.8	163	-21.2	1834	881
mmu-r	ENSMU	306	-38	156	-20.7	6705	490
mmu-r	Gm412	306	-37.4	161	-21.1	2836	3951
mmu-r	ENSMU	306	-37	154	-19.5	3018	5553
mmu-r	ENSMU	306	-37	155	-19.4	8158	107
mmu-r	ENSMU	306	-36.5	163	-20.9	9627	3049
mmu-r	Gm388	306	-35.6	166	-20.5	8772	1882
mmu-r	ENSMU	306	-34.8	156	-19.4	116	4498
mmu-r	ENSMU	306	-34.6	158	-18.5	1741	13419
mmu-r	ENSMU	306	-34.6	158	-18.1	3507	1763
mmu-r	ENSMU	306	-33.8	159	-17.3	9919	3422
mmu-r	ENSMU	306	-33.8	154	-18.8	6564	1070
mmu-r	ENSMU	306	-32.8	153	-17.2	513	3953
mmu-r	ENSMU	306	-32.8	156	-18.7	4795	3155
mmu-r	ENSMU	306	-32.7	156	-17.7	3160	14187
mmu-r	ENSMU	306	-31.9	155	-20.3	7553	4927
mmu-r	ENSMU	306	-31.3	155	-21.5	1066	4522
rco-mil	Gm412	306	-31.2	161	-18.2	3790	2666
mmu-r	ENSMU	306	-31.1	156	-17.2	3145	2237
mmu-r	Gm388	306	-30.4	154	-16.8	4906	2386
mmu-r	ENSMU	306	-29.9	155	-16.1	1194	525
mmu-r	ENSMU	306	-29.5	164	-15.5	3964	6352
mmu-r	ENSMU	306	-29.3	160	-18.7	2460	5911
mmu-r	ENSMU	306	-28.4	162	-15.1	5433	7510
mmu-r	ENSMU	306	-28.3	156	-16.2	3898	1144
mmu-r	ENSMU	306	-28.1	166	-18.9	14004	3505
mmu-r	ENSMU	306	-26.9	157	-15.6	2382	5065
mmu-r	Gm388	306	-25.5	156	-14.6	4090	75

mmu-r	ENSMU	306	-25	166	-16.4	4791	4910
mmu-r	Gm388	306	-21.6	154	-11.7	3238	3213
mmu-r	Gm388	306	-21.6	154	-11.7	3238	3213
mmu-r	Gm388	306	-21.6	154	-11.7	3238	3213
mmu-r	ENSMU	305	-45.9	155	-25.9	1258	3681
mmu-r	ENSMU	305	-44	155	-25.4	3872	8668
mmu-r	ENSMU	305	-43.2	159	-25.8	4286	28
mmu-r	ENSMU	305	-43.1	154	-21.6	884	1837
mmu-r	Gm412	305	-41.6	154	-21.8	3766	280
mmu-r	ENSMU	305	-41.3	165	-26.5	755	232
mmu-r	ENSMU	305	-41	160	-20.7	3334	8667
mmu-r	ENSMU	305	-40	164	-20.7	516	11926
mmu-r	ENSMU	305	-39.7	162	-19.9	3141	368
mmu-r	ENSMU	305	-39.1	154	-20.8	7050	718
rco-mil	ENSMU	305	-38.9	161	-23.8	3244	3350
mmu-r	ENSMU	305	-38.1	160	-21.3	3898	1139
mmu-r	ENSMU	305	-38.1	157	-20.7	4390	5707
mmu-r	ENSMU	305	-38	159	-21.2	2557	1649
mmu-r	Gm388	305	-38	155	-20.1	7467	628
mmu-r	ENSMU	305	-37.8	163	-24.4	3366	1675
mmu-r	ENSMU	305	-36.8	158	-22.4	10942	6881
mmu-r	ENSMU	305	-36.4	153	-20.7	8802	12376
mmu-r	Gm388	305	-35.7	165	-23.4	3975	6927
mmu-r	ENSMU	305	-34.8	156	-17.5	6147	2027
mmu-r	ENSMU	305	-33.9	155	-18.5	2209	2101
mmu-r	ENSMU	305	-33.7	155	-17.2	1658	13308
mmu-r	ENSMU	305	-32.2	154	-16.9	6238	643
mmu-r	ENSMU	305	-32.2	154	-18.4	4131	4644
mmu-r	ENSMU	305	-31.8	156	-19	1137	1150
mmu-r	ENSMU	305	-31.6	157	-17.2	1816	4225
mmu-r	ENSMU	305	-31.3	153	-16.5	3805	490
mmu-r	ENSMU	305	-30.1	156	-18.8	1427	8366
mmu-r	ENSMU	305	-29.6	157	-15.4	990	9092
mmu-r	ENSMU	305	-28.8	157	-15.2	10705	6283
mmu-r	Gm412	305	-28.3	153	-16.8	4570	332
mmu-r	ENSMU	305	-27.5	154	-14.4	2716	2746
mmu-r	ENSMU	305	-25.4	153	-13.4	1758	3994
mmu-r	ENSMU	305	-24.9	155	-13.3	1476	10025
mmu-r	Gm388	305	-24	154	-13.1	7860	6296
mmu-r	ENSMU	304	-59.2	153	-31.1	6299	12409
mmu-r	Gm388	304	-53.6	154	-28	8873	1783
mmu-r	ENSMU	304	-52.5	153	-28.7	6834	5798
mmu-r	ENSMU	304	-52.4	153	-27.4	1702	3188
mmu-r	ENSMU	304	-47.7	155	-23.8	13553	3307
mmu-r	ENSMU	304	-47.1	155	-25.6	3594	4006
mmu-r	ENSMU	304	-47	159	-25.4	713	6400
mmu-r	ENSMU	304	-46.9	156	-24.7	1344	10454
mmu-r	ENSMU	304	-45.8	157	-26.2	10858	8617
mmu-r	ENSMU	304	-45.1	162	-26.9	1148	2417
mmu-r	ENSMU	304	-44.4	154	-22.9	1569	9933
mmu-r	ENSMU	304	-43.5	156	-22.2	1834	885
mmu-r	ENSMU	304	-43.2	154	-22.7	29	1927
mmu-r	ENSMU	304	-42.8	158	-22.4	4368	8201
mmu-r	Gm388	304	-42.7	159	-26.4	1628	2376
mmu-r	Gm412	304	-42.7	154	-22.7	679	2897
mmu-r	ENSMU	304	-42.6	153	-21.7	1544	6702
mmu-r	ENSMU	304	-42.2	159	-21.7	362	7022
mmu-r	Gm388	304	-41.4	162	-22.1	2521	2626
mmu-r	ENSMU	304	-40.4	155	-22.2	1773	559
mmu-r	ENSMU	304	-40.2	156	-20.1	8089	5855
mmu-r	Gm388	304	-40.1	162	-25.8	1427	1357
mmu-r	ENSMU	304	-39	159	-23.9	7487	7714
mmu-r	ENSMU	304	-39	154	-20.4	14480	8154
mmu-r	Gm388	304	-39	160	-22.6	6236	831

mmu-r	ENSMU	304	-37.7	163	-25.3	3920	6084
mmu-r	ENSMU	304	-37.7	162	-24.1	9588	12793
mmu-r	ENSMU	304	-37.5	156	-19.1	3887	2445
mmu-r	ENSMU	304	-37.5	153	-19.2	2875	1276
mmu-r	Gm388	304	-37.4	164	-24	9033	6780
mmu-r	ENSMU	304	-36.4	158	-19.8	3238	5938
mmu-r	ENSMU	304	-35.8	153	-20.7	3133	8834
mmu-r	ENSMU	304	-35.4	157	-22.7	3845	1777
mmu-r	ENSMU	304	-35.2	153	-18.8	5730	14393
mmu-r	ENSMU	304	-35.2	153	-21	11679	4408
mmu-r	Gm412	304	-34.6	159	-21.4	2921	627
mmu-r	ENSMU	304	-34	164	-20.6	12645	560
mmu-r	Gm412	304	-33	162	-20.6	3627	767
mmu-r	ENSMU	304	-33	164	-19.4	8687	1423
mmu-r	ENSMU	304	-32.8	159	-19.3	3576	271
mmu-r	ENSMU	304	-32.7	154	-19.2	4711	10257
mmu-r	ENSMU	304	-32.4	153	-19.4	4360	4270
mmu-r	Gm412	304	-31.1	154	-15.7	3509	3229
mmu-r	ENSMU	304	-29.9	157	-15.1	4070	3326
mmu-r	ENSMU	304	-27.2	154	-14.2	4150	3316
mmu-r	ENSMU	304	-26.4	159	-14.9	1194	527
mmu-r	ENSMU	304	-21.4	157	-11.2	6059	2470
mmu-r	ENSMU	303	-53.1	156	-30.1	750	338
mmu-r	ENSMU	303	-48.4	154	-27.5	8007	8258
mmu-r	ENSMU	303	-48.1	152	-24.8	9425	7094
mmu-r	ENSMU	303	-46.9	152	-24.2	1070	2002
mmu-r	ENSMU	303	-44.9	153	-24.9	1103	6720
mmu-r	ENSMU	303	-44	159	-23.2	6970	7199
mmu-r	ENSMU	303	-43.2	155	-23	4119	3602
mmu-r	Gm388	303	-42.8	156	-23	5133	3990
mmu-r	ENSMU	303	-42.6	156	-24.5	1737	10836
mmu-r	ENSMU	303	-41.7	153	-21.1	7860	5907
mmu-r	ENSMU	303	-41.4	157	-21.4	11040	9627
mmu-r	ENSMU	303	-41.3	160	-29.1	4054	5753
mmu-r	ENSMU	303	-41.2	154	-23.8	8892	8837
mmu-r	ENSMU	303	-40.5	154	-21.6	7484	7712
mmu-r	ENSMU	303	-39	158	-20.3	12063	8147
mmu-r	ENSMU	303	-38.6	155	-22.4	4041	1798
mmu-r	Gm412	303	-38.4	157	-21.5	2563	2475
mmu-r	Gm388	303	-37.2	154	-20.7	649	8507
mmu-r	ENSMU	303	-37.1	158	-21	2611	765
mmu-r	ENSMU	303	-37	153	-20.3	4138	7771
mmu-r	ENSMU	303	-36.7	153	-21.9	11926	402
mmu-r	Gm412	303	-36.5	153	-19.1	3446	367
mmu-r	ENSMU	303	-36.4	158	-19.2	399	3870
mmu-r	ENSMU	303	-36.3	155	-22.8	10963	2696
mmu-r	ENSMU	303	-35.9	154	-20.5	12931	428
mmu-r	Gm388	303	-35.8	155	-19.3	554	6375
mmu-r	Gm388	303	-35.8	154	-21.2	5091	5337
mmu-r	Gm388	303	-35.6	153	-24.2	6324	250
mmu-r	ENSMU	303	-35.6	152	-18.7	2737	7344
rco-mi	ENSMU	303	-34.8	152	-17.7	1021	4498
mmu-r	ENSMU	303	-34.4	153	-20	4145	8757
mmu-r	ENSMU	303	-34.4	155	-18.5	12619	2138
mmu-r	ENSMU	303	-32.7	152	-18.1	1701	2717
mmu-r	ENSMU	303	-32.3	163	-22	3051	3995
mmu-r	ENSMU	303	-32.1	158	-17.1	1904	443
mmu-r	ENSMU	303	-32.1	157	-17.8	9664	735
mmu-r	Gm388	303	-31.9	158	-17.3	5296	7056
mmu-r	ENSMU	303	-30.9	155	-16.5	2719	3034
mmu-r	ENSMU	303	-30.5	163	-20.8	11566	13013
mmu-r	ENSMU	303	-29.9	153	-17.1	1987	7852
mmu-r	ENSMU	303	-29.4	162	-17.9	602	9556
mmu-r	ENSMU	303	-27.5	154	-14.6	4094	2109

mmu-r	ENSMU	303	-24.6	160	-14.8	5669	544
mmu-r	ENSMU	303	-24	159	-13.4	2379	2192
mmu-r	ENSMU	303	-23.4	154	-12.9	3276	3253
mmu-r	Gm388	303	-21.7	156	-11.1	3209	279
mmu-r	Gm388	302	-57.8	152	-29	6464	8425
mmu-r	Gm388	302	-51.4	154	-28.5	6938	6543
mmu-r	ENSMU	302	-51.3	155	-26.9	171	3721
mmu-r	ENSMU	302	-50.2	156	-28.1	9448	4335
mmu-r	ENSMU	302	-47.8	157	-24.2	6027	3733
mmu-r	ENSMU	302	-47.6	152	-25.8	1306	9450
mmu-r	ENSMU	302	-43.9	162	-27	2530	4055
mmu-r	Gm388	302	-43.7	156	-22.5	4732	344
mmu-r	ENSMU	302	-43.6	156	-23.8	3190	1702
mmu-r	Gm388	302	-43.1	154	-25.1	8416	6481
mmu-r	Gm388	302	-42.6	152	-23.7	1865	923
mmu-r	ENSMU	302	-41.2	156	-24.9	702	2115
mmu-r	ENSMU	302	-40.8	157	-25.8	7038	3029
mmu-r	ENSMU	302	-40.7	151	-22.1	1155	6398
mmu-r	ENSMU	302	-40.7	153	-23.1	703	2115
mmu-r	ENSMU	302	-40.2	157	-23.2	10648	11223
mmu-r	ENSMU	302	-39.9	156	-22.5	9126	1689
mmu-r	ENSMU	302	-39.7	162	-22.9	1209	3686
mmu-r	ENSMU	302	-39.6	156	-23.4	2646	5493
mmu-r	ENSMU	302	-39.6	162	-23.4	106	9178
mmu-r	ENSMU	302	-39.5	154	-21.5	2248	4493
mmu-r	Gm412	302	-39.2	153	-20.1	2861	627
mmu-r	Gm412	302	-38.8	156	-19.6	1706	1563
mmu-r	ENSMU	302	-38.6	154	-20.1	8142	3114
mmu-r	ENSMU	302	-38.4	152	-19.7	8616	2592
mmu-r	ENSMU	302	-38.3	162	-23.7	3051	9358
mmu-r	ENSMU	302	-37.9	153	-19.2	2622	3861
rco-mil	ENSMU	302	-37.5	157	-22.1	3301	4341
rco-mil	ENSMU	302	-37.5	157	-22.1	3301	4341
rco-mil	ENSMU	302	-37.5	157	-22.1	3301	4341
rco-mil	ENSMU	302	-37.5	157	-22.1	3301	4341
mmu-r	ENSMU	302	-37.2	159	-21.3	7215	5736
mmu-r	Gm388	302	-36.8	157	-19.9	5353	1162
mmu-r	ENSMU	302	-36.8	154	-18.4	1754	384
mmu-r	ENSMU	302	-36.6	162	-24.8	1488	8274
mmu-r	ENSMU	302	-36.4	153	-18.4	611	5808
mmu-r	ENSMU	302	-36.3	154	-22.4	4378	13785
mmu-r	ENSMU	302	-36.2	152	-21.7	1847	9506
mmu-r	Gm388	302	-35.9	155	-18.3	6207	8494
mmu-r	Gm412	302	-35.6	153	-18.8	587	1710
mmu-r	ENSMU	302	-35	154	-19.4	81	3255
mmu-r	ENSMU	302	-35	157	-19.7	102	828
rco-mil	ENSMU	302	-34.7	152	-19.6	1021	4500
rco-mil	ENSMU	302	-34.1	152	-19	1021	4500
rco-mil	ENSMU	302	-34.1	152	-19	1021	4500
mmu-r	Gm412	302	-34	158	-21.4	3767	285
mmu-r	ENSMU	302	-33.8	159	-18.7	9374	10823
mmu-r	ENSMU	302	-33.3	154	-20.5	5637	9007
mmu-r	ENSMU	302	-33.1	152	-16.7	2284	6890
mmu-r	ENSMU	302	-33.1	162	-19	1516	1486
mmu-r	ENSMU	302	-32.8	152	-17.6	11327	12735
mmu-r	ENSMU	302	-32.1	155	-17.7	3695	298
mmu-r	ENSMU	302	-31.7	160	-16.4	4560	8486
mmu-r	ENSMU	302	-31.4	155	-17.1	10393	11796
mmu-r	ENSMU	302	-31.4	152	-15.7	4061	4052
mmu-r	ENSMU	302	-31.1	157	-17.4	6162	598
mmu-r	ENSMU	302	-30.8	159	-19.8	11974	11389
mmu-l	Gm388	302	-30.6	157	-17.4	4260	5011
mmu-r	ENSMU	302	-29.8	153	-15.8	38	139
mmu-r	ENSMU	302	-29.8	153	-16.5	9919	3418

mmu-r	ENSMU	302	-29.2	155	-16.3	1249	7833
mmu-r	ENSMU	302	-28.6	152	-15	5274	945
mmu-r	ENSMU	302	-28	155	-14.5	11639	1270
mmu-r	ENSMU	302	-27.7	154	-15.2	6608	3714
mmu-r	ENSMU	302	-26.8	156	-15.6	5936	9600
mmu-r	ENSMU	302	-23.1	156	-12.1	6936	8520
mmu-r	ENSMU	301	-57.2	160	-31.5	1167	4423
mmu-r	ENSMU	301	-51.7	161	-29.4	1673	1733
mmu-r	ENSMU	301	-51.3	156	-29	684	1195
mmu-r	Gm412	301	-51.2	154	-29.4	4059	4489
mmu-r	ENSMU	301	-49.6	154	-25.8	8900	12769
mmu-r	ENSMU	301	-47.2	154	-27.3	10509	10409
mmu-r	ENSMU	301	-46.8	155	-25	3387	586
mmu-r	ENSMU	301	-46.2	155	-23.7	1474	1710
mmu-r	Gm388	301	-45	154	-25.9	366	720
mmu-r	ENSMU	301	-44	153	-23.8	1475	1709
mmu-r	ENSMU	301	-43.9	155	-24.6	1380	2634
mmu-r	Gm412	301	-41.5	157	-27.9	1228	2623
mmu-r	Gm388	301	-41.5	154	-21.7	1333	825
mmu-r	Gm388	301	-41.2	155	-21.4	3400	8989
mmu-r	ENSMU	301	-41	153	-20.7	10908	7485
mmu-r	Gm412	301	-40.9	156	-27.9	2710	3560
mmu-r	ENSMU	301	-40.6	154	-22.8	2328	12706
mmu-r	Gm388	301	-40.2	156	-21.2	4011	5027
mmu-r	ENSMU	301	-40.1	158	-24.6	3254	89
mmu-r	Gm388	301	-39.8	158	-26	8019	1559
mmu-r	ENSMU	301	-39.7	151	-20.3	10421	1391
mmu-r	Gm412	301	-39.1	154	-24.7	2439	853
mmu-r	Gm388	301	-39.1	154	-20.3	1355	8894
mmu-r	ENSMU	301	-37.5	151	-21.3	6078	1912
mmu-r	ENSMU	301	-37.1	161	-22	9427	5710
mmu-r	ENSMU	301	-35.2	160	-22.6	11274	2247
mmu-r	ENSMU	301	-35.2	155	-18.7	7002	5425
mmu-r	ENSMU	301	-34.6	156	-21.7	8106	8255
mmu-r	ENSMU	301	-34.6	152	-17.9	2258	185
mmu-r	Gm388	301	-34.3	155	-25.2	633	1467
mmu-r	ENSMU	301	-34.1	156	-20.3	5642	3399
mmu-r	Gm412	301	-33.6	157	-18.8	4359	4474
mmu-r	ENSMU	301	-32.8	158	-19.4	3476	1304
mmu-r	ENSMU	301	-32.3	153	-18.7	4409	1189
mmu-r	ENSMU	301	-32.1	158	-21.8	685	8724
mmu-r	Gm388	301	-32	159	-22	2249	2403
mmu-r	ENSMU	301	-32	153	-17.7	4409	1189
mmu-r	ENSMU	301	-32	155	-17.1	5072	4391
mmu-r	Gm388	301	-32	152	-16.6	1243	8698
mmu-r	Gm388	301	-31.5	154	-17.5	4810	8023
mmu-r	ENSMU	301	-31.2	156	-19.2	3212	5889
mmu-r	ENSMU	301	-30.3	154	-16.1	3734	3040
mmu-r	ENSMU	301	-29.4	151	-18.5	3352	4318
mmu-r	Gm412	301	-29	151	-18.7	3920	4264
mmu-r	ENSMU	301	-28.9	156	-17.9	4565	1993
mmu-r	ENSMU	301	-28.3	161	-17.5	4555	5058
mmu-r	ENSMU	301	-27	153	-14.5	4505	4922
mmu-r	ENSMU	301	-26.3	155	-13.6	12129	3241
mmu-r	ENSMU	301	-23.7	154	-14.9	1041	9335
mmu-r	Gm388	301	-20.6	158	-11.6	4985	3746
mmu-r	ENSMU	300	-51.6	150	-26.9	4707	4949
mmu-r	ENSMU	300	-49.5	151	-26.5	2441	8751
mmu-r	ENSMU	300	-47.1	151	-25.2	3938	588
mmu-r	ENSMU	300	-46.9	154	-24.1	2105	3493
mmu-r	Gm388	300	-44.8	155	-23.6	2596	6087
mmu-r	ENSMU	300	-43.4	153	-22.3	1568	8227
mmu-r	ENSMU	300	-42.2	151	-22.4	10429	4554
mmu-r	Gm412	300	-41.3	159	-22.5	3603	301

mmu-r	ENSMU	300	-41	151	-27.1	5835	1007
mmu-r	ENSMU	300	-40.4	152	-21.5	3313	7075
mmu-r	ENSMU	300	-40.2	151	-20.9	7017	7863
rco-mil	ENSMU	300	-39.9	153	-24.4	5424	2226
mmu-r	Gm412	300	-39.6	158	-24.4	2225	4397
mmu-r	ENSMU	300	-38.5	155	-20	11923	404
mmu-r	ENSMU	300	-38.3	157	-19.3	7191	7540
mmu-r	Gm388	300	-37.8	153	-21	1497	8710
mmu-r	ENSMU	300	-37.2	152	-21.6	1837	884
mmu-r	ENSMU	300	-37.1	152	-20.4	2272	5662
mmu-r	ENSMU	300	-37	152	-18.8	7733	801
mmu-r	ENSMU	300	-36.8	155	-19.1	3208	7530
mmu-r	ENSMU	300	-36.8	154	-19.5	1628	7989
mmu-r	ENSMU	300	-36.5	157	-19.3	7192	7541
mmu-r	ENSMU	300	-36.3	154	-21.1	3489	8897
mmu-r	ENSMU	300	-35.9	154	-19.7	2240	1520
mmu-r	ENSMU	300	-35.8	157	-23.2	4106	4250
mmu-r	ENSMU	300	-35.8	160	-24	7979	9712
mmu-r	ENSMU	300	-34.3	153	-20.8	1277	6288
mmu-r	ENSMU	300	-33.6	152	-17.3	4517	2582
mmu-r	ENSMU	300	-33.6	152	-17.5	2347	6411
mmu-r	ENSMU	300	-33.6	155	-19.4	5741	14205
mmu-r	ENSMU	300	-33.5	154	-18.6	2635	1697
mmu-r	ENSMU	300	-33	154	-17.6	6873	2170
mmu-r	ENSMU	300	-32.8	151	-16.9	12620	2139
mmu-r	ENSMU	300	-32.4	151	-17.4	4049	3184
mmu-r	ENSMU	300	-32.1	157	-17.3	3350	4390
mmu-r	ENSMU	300	-32.1	154	-17.3	1344	3950
mmu-r	ENSMU	300	-31.7	152	-19.6	6502	7041
mmu-r	ENSMU	300	-31.3	152	-16.9	8801	4047
mmu-r	Gm388	300	-31.2	153	-18.5	1496	5031
mmu-r	ENSMU	300	-30.8	154	-16.3	8926	10090
mmu-r	Gm388	300	-30.4	151	-18.8	1228	8682
mmu-r	ENSMU	300	-29.8	153	-16.8	172	7070
mmu-r	ENSMU	300	-29.7	153	-15.4	4056	2531
mmu-r	ENSMU	300	-29.7	160	-17.2	8687	1423
mmu-r	ENSMU	300	-28.4	160	-17.2	1211	6345
mmu-r	ENSMU	300	-27.5	153	-13.9	3912	854
mmu-r	ENSMU	300	-27.2	151	-14.1	906	1749
mmu-r	ENSMU	300	-27.1	153	-16.1	9903	5601
mmu-r	ENSMU	300	-26.7	160	-18	1033	12279
mmu-r	Gm388	300	-26.6	155	-13.4	4441	4823
mmu-r	Gm388	300	-26.6	155	-13.4	4441	4823
mmu-r	ENSMU	300	-25.7	154	-13.7	5172	3717
mmu-r	ENSMU	300	-25.4	151	-14.8	6456	3853
mmu-r	ENSMU	300	-24.8	157	-14.8	5277	944
mmu-r	ENSMU	300	-23.8	154	-15.9	2168	1742
mmu-r	Gm388	300	-21.4	155	-12	3643	3328
mmu-r	ENSMU	299	-53.8	153	-27.3	6401	714
mmu-r	ENSMU	299	-50.1	157	-28.1	4189	6664
mmu-r	ENSMU	299	-47.7	152	-24.4	6345	5329
mmu-r	Gm412	299	-47.4	159	-29.5	1022	1906
mmu-r	Gm388	299	-46.6	156	-24.9	8850	3346
mmu-r	Gm388	299	-46.1	159	-27.7	86	2553
mmu-r	ENSMU	299	-44.3	159	-27.1	5266	5955
mmu-r	ENSMU	299	-43.3	159	-23.9	4439	5627
mmu-r	ENSMU	299	-42.1	151	-25	4235	5445
mmu-r	ENSMU	299	-41.4	150	-24	1120	2564
mmu-r	Gm388	299	-41.4	152	-22.3	5356	8571
mmu-r	ENSMU	299	-41.2	154	-20.7	31	5213
mmu-r	ENSMU	299	-39.1	154	-19.8	2529	4195
mmu-r	Gm388	299	-37.6	154	-19.9	7828	8615
mmu-r	ENSMU	299	-37.4	154	-19.5	12349	5741
mmu-r	Gm412	299	-36.2	154	-21.6	1942	4458

mmu-r	ENSMU	299	-35.7	155	-19	3780	1521
mmu-r	ENSMU	299	-35.7	154	-21.8	4675	4240
mmu-r	ENSMU	299	-35.2	155	-18	2190	664
mmu-r	Gm388	299	-34.7	153	-17.9	3907	4983
mmu-r	ENSMU	299	-34.2	151	-17.4	7719	683
mmu-r	Gm388	299	-34	155	-18.7	7470	8214
mmu-r	Gm388	299	-33.6	154	-19.3	4430	8188
mmu-r	Gm388	299	-33.2	154	-17.6	1418	1806
mmu-r	ENSMU	299	-33.2	150	-18.3	12216	6964
mmu-r	ENSMU	299	-32.6	153	-16.5	2718	3033
mmu-r	ENSMU	299	-32.6	150	-17.4	7104	7079
mmu-r	Gm388	299	-32.3	155	-20.5	8737	99
mmu-r	ENSMU	299	-32.3	155	-17.4	4938	2262
mmu-r	ENSMU	299	-31.4	156	-19.7	756	232
rco-mil	ENSMU	299	-31	150	-16.2	8114	10579
rco-mil	ENSMU	299	-31	150	-16.2	8114	10579
rco-mil	ENSMU	299	-31	150	-16.2	8114	10579
mmu-r	ENSMU	299	-30.9	154	-15.5	3483	4275
mmu-r	ENSMU	299	-30.7	152	-18.4	2805	58
rco-mil	ENSMU	299	-30	155	-16.4	4339	1430
rco-mil	ENSMU	299	-30	155	-16.4	4339	1430
rco-mil	ENSMU	299	-30	155	-16.4	4339	1430
mmu-r	ENSMU	299	-29.8	155	-15.8	7022	3501
mmu-r	ENSMU	299	-28.5	151	-14.5	225	1419
mmu-r	ENSMU	299	-28.3	151	-15.7	9034	11655
mmu-r	ENSMU	299	-28	155	-17.1	5770	6528
mmu-r	ENSMU	299	-27.6	159	-17.1	3881	4213
mmu-r	ENSMU	299	-27.6	155	-16.6	2751	3425
mmu-r	ENSMU	299	-27.4	151	-13.7	87	3255
mmu-r	ENSMU	299	-27.2	152	-17.2	4744	3283
mmu-r	ENSMU	299	-26.5	151	-15	482	701
mmu-r	ENSMU	299	-25.4	156	-14.1	12996	3210
mmu-r	ENSMU	299	-24.4	155	-13.9	1298	4373
mmu-r	ENSMU	299	-24.1	152	-15	1100	3420
mmu-r	ENSMU	299	-23.1	151	-11.8	8227	5554
mmu-r	ENSMU	298	-50.4	158	-28.4	1157	6397
mmu-r	Gm388	298	-48.7	151	-25.3	5181	220
mmu-r	ENSMU	298	-48	155	-29	6302	12410
mmu-r	ENSMU	298	-46.6	157	-24.5	3488	4019
mmu-r	Gm388	298	-45.6	153	-24.5	9041	3549
mmu-r	ENSMU	298	-45.6	154	-25.9	1546	6700
mmu-r	ENSMU	298	-45.6	150	-25.2	3389	586
mmu-r	Gm388	298	-44.8	154	-27.4	8989	3403
mmu-r	ENSMU	298	-44.6	150	-26.2	3961	2307
mmu-r	ENSMU	298	-44.6	153	-22.4	2729	276
mmu-r	ENSMU	298	-44.5	153	-25.1	7910	10413
mmu-r	ENSMU	298	-44.3	152	-24.4	4684	4357
mmu-r	ENSMU	298	-42.6	149	-21.3	2755	5453
mmu-r	ENSMU	298	-40.8	155	-28.6	4054	5753
mmu-r	Gm388	298	-40.7	156	-20.7	2337	2963
rco-mil	ENSMU	298	-40.5	150	-22.6	10917	4343
rco-mil	ENSMU	298	-40.5	150	-22.6	10917	4343
rco-mil	ENSMU	298	-40.5	150	-22.6	10917	4343
rco-mil	ENSMU	298	-40.5	150	-22.6	10917	4343
mmu-r	ENSMU	298	-40.3	158	-24.1	889	2584
mmu-r	Gm388	298	-40.3	149	-20.6	4821	8962
mmu-r	ENSMU	298	-40.2	154	-23.8	2727	276
mmu-r	ENSMU	298	-39.7	157	-23	10531	2476
mmu-r	ENSMU	298	-38.1	152	-21.6	3995	1913
mmu-r	ENSMU	298	-36.5	152	-21	3683	4261
mmu-r	ENSMU	298	-36.4	151	-19.2	6304	4249
mmu-r	ENSMU	298	-36.3	158	-22.7	4264	3936
mmu-r	ENSMU	298	-36.3	158	-20.8	114	1309
mmu-r	ENSMU	298	-36.2	154	-18.2	3542	4541

mmu-r	ENSMU	298	-36	150	-20.9	54	12354
mmu-r	ENSMU	298	-35.9	157	-20.2	7962	5473
mmu-r	ENSMU	298	-35.9	155	-19.2	207	4193
mmu-r	ENSMU	298	-35.7	158	-20.3	5559	5158
mmu-r	ENSMU	298	-35.6	152	-19.2	3953	513
mmu-r	ENSMU	298	-35.5	152	-20	6624	6606
mmu-r	ENSMU	298	-34.5	153	-17.4	4836	5013
mmu-r	ENSMU	298	-34.3	155	-23.6	8190	6541
mmu-r	Gm388	298	-34.3	158	-19.9	4466	9004
mmu-r	Gm388	298	-33.9	154	-19.7	4523	7812
mmu-r	Gm388	298	-33.8	154	-17.6	527	5
mmu-r	Gm388	298	-33.7	153	-16.9	7126	8853
mmu-r	ENSMU	298	-33.7	155	-17	4994	4355
mmu-r	ENSMU	298	-33.6	152	-18.5	245	3765
mmu-r	ENSMU	298	-32.8	150	-18.7	3155	4795
mmu-r	ENSMU	298	-32.5	151	-18.2	637	2280
mmu-r	ENSMU	298	-32.3	155	-17.7	1834	886
mmu-r	ENSMU	298	-31.8	153	-16.7	8786	6391
mmu-r	ENSMU	298	-31.7	156	-17.3	6211	10344
mmu-r	ENSMU	298	-31.7	150	-17.9	10658	6741
mmu-r	ENSMU	298	-31.6	152	-17.8	5057	6945
mmu-r	ENSMU	298	-31	157	-17.4	11926	403
mmu-r	ENSMU	298	-30.9	152	-18.5	5522	681
mmu-r	Gm412	298	-30.8	154	-15.8	4550	803
mmu-r	ENSMU	298	-30.3	154	-18.8	2328	1224
mmu-r	ENSMU	298	-30	156	-17	3551	4045
mmu-r	ENSMU	298	-29.9	157	-17.9	3509	2253
mmu-r	ENSMU	298	-29.1	158	-18.8	151	3725
mmu-r	ENSMU	298	-28.9	156	-16.7	1798	11331
mmu-r	Gm388	298	-27.9	152	-17.3	2838	3765
mmu-r	ENSMU	298	-27.6	155	-14.7	9485	3115
mmu-r	ENSMU	298	-26.8	158	-15.5	7508	5949
mmu-r	ENSMU	298	-26.8	149	-14.6	5386	6401
mmu-r	Gm388	298	-25.3	153	-14.4	4769	3489
mmu-r	ENSMU	298	-25	153	-15.4	598	3524
mmu-r	ENSMU	298	-24.5	153	-12.6	3912	854
mmu-r	Gm412	298	-24.2	152	-14.2	2979	3092
mmu-r	ENSMU	298	-23.8	158	-15	11354	580
mmu-r	ENSMU	298	-23.2	158	-15.3	1427	365
mmu-r	ENSMU	298	-22	154	-12.2	5199	4527
mmu-r	ENSMU	298	-21.1	153	-11.6	601	3523
mmu-r	ENSMU	297	-48.2	155	-29.2	3238	5940
mmu-r	ENSMU	297	-47.2	152	-25.5	2433	1693
mmu-r	ENSMU	297	-45.6	150	-23	608	1
mmu-r	ENSMU	297	-45.6	155	-24.4	1348	1327
mmu-r	ENSMU	297	-45.1	151	-24.2	13268	4325
mmu-r	ENSMU	297	-45	149	-25.1	7190	7531
mmu-r	Gm388	297	-43.9	149	-25.8	7711	439
mmu-r	Gm412	297	-43.3	155	-23.9	555	3155
mmu-r	ENSMU	297	-42.8	153	-22	4116	832
mmu-r	ENSMU	297	-42.5	157	-21.7	3382	6848
mmu-r	Gm412	297	-42	149	-24.2	1559	1704
mmu-r	ENSMU	297	-41.7	149	-22.3	4684	2379
mmu-r	Gm388	297	-41.2	152	-21.8	8789	430
mmu-r	ENSMU	297	-41.1	156	-23.2	1800	4042
mmu-r	ENSMU	297	-41	150	-23.3	5707	4391
mmu-r	ENSMU	297	-39.9	157	-20.5	3213	171
mmu-r	Gm412	297	-39.1	151	-22.3	1774	676
mmu-r	Gm388	297	-39	157	-25.2	2162	4418
mmu-r	ENSMU	297	-38.8	151	-20	2139	12620
mmu-r	Gm388	297	-38.5	157	-23.8	2162	4418
mmu-r	ENSMU	297	-38.5	156	-23	3675	10238
mmu-r	Gm388	297	-37.5	154	-23.5	2058	3105
mmu-r	ENSMU	297	-37.1	153	-20	2355	3113

mmu-r	ENSMU	297	-36.8	150	-20.2	2314	2662
mmu-r	ENSMU	297	-36.3	149	-19	1862	3607
mmu-r	ENSMU	297	-35.9	155	-22.2	3696	7384
mmu-r	ENSMU	297	-35.4	150	-18.8	1346	3398
mmu-r	ENSMU	297	-35.3	149	-18.1	3387	4224
mmu-r	ENSMU	297	-35.3	149	-17.7	3905	3155
mmu-r	ENSMU	297	-35.2	157	-23	3368	8035
mmu-r	ENSMU	297	-35	155	-17.7	5603	4559
mmu-r	ENSMU	297	-34.9	156	-20.4	755	233
mmu-r	ENSMU	297	-34.5	155	-19.2	162	1836
mmu-r	ENSMU	297	-33.9	152	-20.4	2648	7476
mmu-r	ENSMU	297	-33.7	151	-18.8	1278	3291
mmu-r	ENSMU	297	-33.6	155	-17.1	2565	1120
mmu-r	ENSMU	297	-33.3	151	-18.4	3859	2621
mmu-r	ENSMU	297	-33.1	150	-18.7	14340	13594
mmu-r	ENSMU	297	-33	149	-16.7	963	5125
hiv1-m	Gm388	297	-32.9	151	-18.2	1367	3594
mmu-r	ENSMU	297	-32.7	152	-16.4	11427	9453
mmu-r	ENSMU	297	-32.6	152	-19.2	8992	1466
mmu-r	ENSMU	297	-32.3	154	-16.4	5664	5740
mmu-r	ENSMU	297	-32.1	155	-19.3	6728	7442
mmu-r	ENSMU	297	-32	150	-17.6	3520	595
mmu-r	ENSMU	297	-31.7	149	-16.3	1071	746
mmu-r	ENSMU	297	-31.5	154	-18.7	1628	1879
mmu-r	ENSMU	297	-31.4	151	-16.7	4873	3165
mmu-r	ENSMU	297	-31.2	149	-15.8	963	5125
mmu-r	ENSMU	297	-30.8	149	-17.5	12873	4370
mmu-r	Gm388	297	-30	152	-16.2	4223	4792
mmu-r	ENSMU	297	-29.5	155	-16.3	9180	107
mmu-r	ENSMU	297	-29.3	149	-18.2	2799	2857
mmu-r	ENSMU	297	-29.3	155	-20.6	11531	3330
mmu-r	ENSMU	297	-28.7	152	-15.8	10951	5611
mmu-r	ENSMU	297	-28.5	157	-15.4	1199	3620
mmu-r	ENSMU	297	-28.5	156	-18.4	8709	7693
mmu-r	ENSMU	297	-28.3	152	-14.8	4215	245
mmu-r	ENSMU	297	-28	149	-17.5	477	7014
mmu-r	ENSMU	297	-26.4	155	-13.7	4145	249
mmu-r	ENSMU	297	-25.6	150	-16.7	4588	1037
mmu-r	ENSMU	297	-25.2	152	-12.8	1193	526
mmu-r	ENSMU	297	-24.3	154	-18	14300	3682
mmu-r	Gm388	297	-23.3	150	-11.7	2602	3449
mmu-r	ENSMU	297	-23.2	155	-11.6	3698	303
mmu-r	ENSMU	297	-22.6	149	-12	945	5277
mmu-r	Gm388	297	-22.3	151	-12.2	7861	6296
mmu-r	Gm388	297	-20.8	151	-12.6	6436	853
mmu-r	Gm412	297	-20.2	157	-10.3	4346	4332
mmu-r	Gm412	296	-53.1	154	-30.1	2566	3523
mmu-r	ENSMU	296	-50.3	156	-30.4	7141	11805
mmu-r	Gm388	296	-48.9	150	-29.8	6460	8428
mmu-r	Gm412	296	-47.2	155	-27.4	4053	4488
mmu-r	ENSMU	296	-45.7	155	-25.6	7380	6133
mmu-r	ENSMU	296	-45.4	149	-23.5	2114	695
mmu-r	Gm388	296	-43.3	154	-24.4	6460	8427
mmu-r	ENSMU	296	-43.1	148	-23.6	3312	13293
mmu-r	ENSMU	296	-42.6	152	-23.4	1826	356
mmu-r	ENSMU	296	-42.6	152	-21.6	2415	14404
mmu-r	ENSMU	296	-42.5	152	-22.5	7542	5047
mmu-r	ENSMU	296	-41.9	149	-21.2	424	3750
mmu-r	ENSMU	296	-40.6	149	-23	8140	7151
mmu-r	Gm412	296	-40.4	156	-23.2	4076	2723
mmu-r	ENSMU	296	-39.8	148	-21.6	2361	9359
mmu-r	ENSMU	296	-39.4	150	-21.1	5855	8091
mmu-r	ENSMU	296	-39.1	151	-19.9	2727	2745
mmu-r	ENSMU	296	-39.1	154	-21.9	1969	4176

mmu-r	ENSMU	296	-39	156	-23.8	2440	8582
mmu-r	ENSMU	296	-38.4	150	-20.2	8026	8199
mmu-r	ENSMU	296	-38.3	155	-22.3	4552	5601
mmu-r	ENSMU	296	-38.3	155	-21.8	284	4507
mmu-r	ENSMU	296	-38	148	-21.4	7966	10633
mmu-r	ENSMU	296	-37.3	148	-18.8	3363	8030
mmu-r	Gm388	296	-37.1	150	-19.2	1189	2776
mmu-r	ENSMU	296	-36.9	155	-22.9	9425	5290
mmu-r	ENSMU	296	-36.6	151	-20.3	12352	4297
mmu-r	Gm388	296	-36.4	149	-18.9	4819	8964
mmu-r	ENSMU	296	-36.1	151	-19.1	535	3457
mmu-r	ENSMU	296	-36	148	-23.3	685	1990
mmu-r	ENSMU	296	-35.9	156	-19.7	6202	5715
mmu-r	Gm388	296	-35.8	149	-18.8	4529	5451
mmu-r	ENSMU	296	-35.6	148	-18	889	2587
mmu-r	ENSMU	296	-35.4	152	-21.7	6851	5080
mmu-r	ENSMU	296	-35.3	155	-18.2	2189	664
mmu-r	ENSMU	296	-35.2	153	-17.9	12457	12365
mmu-r	ENSMU	296	-35	156	-20.9	799	124
mmu-r	Gm388	296	-34.3	156	-21.7	7042	7589
mmu-r	Gm388	296	-34.1	149	-18	650	1317
mmu-r	ENSMU	296	-33.9	151	-24	10904	7778
mmu-r	ENSMU	296	-33.8	149	-18.9	962	5518
mmu-r	ENSMU	296	-33.4	149	-17.6	6233	646
mmu-r	ENSMU	296	-33.1	152	-18.1	6162	596
mmu-r	ENSMU	296	-32.4	150	-18.9	2413	1469
mmu-r	ENSMU	296	-31.8	154	-17.4	585	3888
mmu-r	ENSMU	296	-31.5	148	-16.9	513	3954
mmu-r	ENSMU	296	-31.4	149	-16.8	8205	5952
mmu-r	ENSMU	296	-31.4	150	-15.9	7720	683
mmu-r	ENSMU	296	-31.3	156	-17.8	1406	2321
mmu-r	ENSMU	296	-30.4	152	-15.2	3093	1606
mmu-r	Gm388	296	-30.4	151	-19.2	835	7223
mmu-r	Gm388	296	-30.2	154	-17.1	3471	7038
mmu-r	ENSMU	296	-30	150	-16.6	4452	11705
mmu-r	ENSMU	296	-29.4	149	-15.6	8899	1518
mmu-r	ENSMU	296	-29.2	156	-19	7971	10638
mmu-r	ENSMU	296	-28.6	151	-14.8	1505	45
mmu-r	ENSMU	296	-28.5	152	-16.3	7613	864
mmu-r	ENSMU	296	-28.5	154	-15.5	9625	5926
mmu-r	ENSMU	296	-27.8	149	-14.4	9333	3654
mmu-r	ENSMU	296	-27.7	152	-16.1	3898	1145
mmu-r	ENSMU	296	-27.1	149	-14.4	13247	14253
mmu-r	ENSMU	296	-26.8	149	-14.5	6718	7934
mmu-r	ENSMU	296	-26.5	151	-13.6	5856	2819
mmu-r	ENSMU	296	-25.2	149	-15.8	6196	2109
mmu-r	ENSMU	296	-24.9	156	-14.4	6355	4641
mmu-r	ENSMU	296	-24.8	151	-12.4	3154	4796
mmu-r	ENSMU	296	-23.7	150	-14.2	2396	5540
mmu-r	ENSMU	296	-23.1	149	-12.2	5554	5346
mmu-r	ENSMU	296	-22.6	149	-12.7	12266	6820
mmu-r	Gm388	295	-46.5	150	-23.4	9043	7889
mmu-r	ENSMU	295	-44.8	152	-27.2	2413	1150
mmu-r	ENSMU	295	-44.8	150	-25.8	4329	362
mmu-r	ENSMU	295	-44.2	149	-24.5	5733	4044
mmu-r	Gm388	295	-44.1	148	-23.3	8243	1120
mmu-r	Gm388	295	-43.8	154	-23.1	220	5175
mmu-r	Gm388	295	-43.8	155	-24	6208	8472
mmu-r	ENSMU	295	-42.9	151	-22.1	6159	7502
mmu-r	Gm412	295	-41.8	148	-22.4	3783	2463
hiv1-m	ENSMU	295	-40.8	151	-22.9	11130	3753
mmu-r	ENSMU	295	-40	150	-23.8	277	9728
mmu-r	ENSMU	295	-39.1	154	-21.8	2507	6953
mmu-r	ENSMU	295	-39	148	-20.7	6852	1077

mmu-r	ENSMU	295	-38.3	148	-20.2	6210	10344
mmu-r	ENSMU	295	-38.2	150	-19.4	3426	3369
mmu-r	Gm388	295	-37.8	151	-19.1	8135	4473
mmu-r	ENSMU	295	-37.3	155	-21.8	285	4508
mmu-r	ENSMU	295	-37.2	153	-22.4	11971	8246
mmu-r	ENSMU	295	-37.2	150	-21.7	483	2173
mmu-r	ENSMU	295	-37.2	148	-20.9	7336	3140
mmu-r	ENSMU	295	-36.9	154	-20.5	1210	3688
mmu-r	ENSMU	295	-36.4	155	-22.2	2292	6887
mmu-r	ENSMU	295	-36.3	155	-20.6	3314	26
mmu-r	ENSMU	295	-34.5	152	-19.4	4229	3391
mmu-r	ENSMU	295	-34.5	148	-17.4	5663	2273
mmu-r	ENSMU	295	-34.4	150	-18	2241	4822
mmu-r	ENSMU	295	-33	153	-17.1	2976	648
mmu-r	ENSMU	295	-32.7	155	-21.7	896	1953
mmu-r	ENSMU	295	-32.5	148	-18.8	5276	163
mmu-r	ENSMU	295	-32.3	149	-16.5	8921	6949
mmu-r	ENSMU	295	-31.8	155	-17	4788	5430
mmu-r	ENSMU	295	-30.9	148	-15.8	803	7735
mmu-r	ENSMU	295	-30.9	148	-15.8	803	7735
mmu-r	ENSMU	295	-30.6	149	-17.2	6765	1192
mmu-r	ENSMU	295	-30.4	150	-17.1	4305	9139
mmu-r	Gm388	295	-30.3	153	-16.6	3460	4806
mmu-r	ENSMU	295	-30.3	149	-16.6	192	158
mmu-r	Gm388	295	-30.1	155	-20.8	4985	7928
mmu-r	Gm388	295	-30.1	151	-17.4	7624	7965
mmu-r	ENSMU	295	-30.1	153	-18.4	5363	5381
mmu-r	ENSMU	295	-29.7	148	-18.5	453	858
rco-mil	ENSMU	295	-29.7	150	-17.2	3303	4341
mmu-r	Gm412	295	-29.5	148	-16.4	757	1571
mmu-r	ENSMU	295	-29.4	154	-15.6	651	5983
mmu-r	ENSMU	295	-29.2	154	-17.8	3368	5887
mmu-r	ENSMU	295	-29.1	151	-16.8	4158	5668
mmu-r	ENSMU	295	-29	150	-15.2	1592	2232
mmu-r	ENSMU	295	-29	151	-16.9	7931	2520
mmu-r	ENSMU	295	-28.8	150	-17.4	496	1688
mmu-r	Gm388	295	-28.1	150	-14.2	8167	8718
mmu-r	ENSMU	295	-27.8	153	-17.3	10260	3677
mmu-r	ENSMU	295	-27.4	149	-17.2	6765	1192
rco-mil	ENSMU	295	-27.1	148	-15.2	5432	2227
rco-mil	ENSMU	295	-27.1	148	-15.2	5432	2227
rco-mil	ENSMU	295	-27	155	-13.5	2551	9817
rco-mil	ENSMU	295	-27	155	-13.5	2551	9817
mmu-r	ENSMU	295	-26.8	155	-18.3	4064	953
mmu-r	ENSMU	295	-26.7	155	-18.4	2695	4048
mmu-r	ENSMU	295	-26.4	148	-13.7	5923	3470
mmu-r	ENSMU	295	-26.4	150	-15.4	6811	4792
mmu-r	ENSMU	295	-26.4	153	-14.6	4789	4911
mmu-r	ENSMU	295	-24.9	148	-14.1	12235	5268
mmu-r	ENSMU	295	-24.5	153	-14.8	9877	9685
mmu-r	ENSMU	295	-22.7	148	-12.9	837	1469
mmu-r	ENSMU	295	-21.8	155	-11	2793	2854
mmu-r	ENSMU	295	-20.3	152	-14.1	9046	3480
mmu-r	ENSMU	294	-55.5	152	-29.2	1178	1174
mmu-r	Gm388	294	-45	148	-22.9	6972	1253
mmu-r	ENSMU	294	-44.8	153	-27.5	4493	25
mmu-r	Gm388	294	-44.7	154	-24.8	219	5176
mmu-r	Gm388	294	-44.2	154	-25.5	7052	2372
mmu-r	ENSMU	294	-40.6	152	-22	6398	1158
mmu-r	ENSMU	294	-40.5	150	-20.5	1707	1206
mmu-r	Gm388	294	-39.7	151	-22.5	6969	2352
mmu-r	ENSMU	294	-39.2	151	-21.2	4739	1828
mmu-r	ENSMU	294	-38.9	148	-23.4	6915	11516
mmu-r	ENSMU	294	-38	147	-20.4	3731	6028

mmu-r	Gm388	294	-38	149	-19.7	2814	5420
mmu-r	ENSMU	294	-37.1	153	-20.9	1270	9939
mmu-r	ENSMU	294	-37	148	-19.3	5243	10066
mmu-r	ENSMU	294	-36.9	154	-19.8	1973	4180
mmu-r	ENSMU	294	-35.4	150	-19.4	4062	4769
mmu-r	ENSMU	294	-35.4	150	-21	2077	10360
mmu-r	ENSMU	294	-34.5	153	-22.3	8997	1466
mmu-r	ENSMU	294	-34.2	149	-20.2	6167	785
mmu-r	ENSMU	294	-33.6	150	-17.7	885	1834
mmu-r	Gm388	294	-32.2	147	-16.6	6043	7467
mmu-r	ENSMU	294	-31.4	152	-19	9027	8653
mmu-r	ENSMU	294	-31	151	-16.2	2640	1701
mmu-r	ENSMU	294	-30.7	154	-20.3	12266	9618
mmu-r	ENSMU	294	-30.6	148	-16.6	8002	12213
mmu-r	ENSMU	294	-30.3	154	-18.8	4465	19
mmu-r	ENSMU	294	-30.1	151	-15.5	11565	3574
mmu-r	ENSMU	294	-30	152	-17.5	5879	3964
mmu-r	ENSMU	294	-29.4	149	-15.8	488	10154
mmu-r	ENSMU	294	-28.5	149	-15.6	3237	5511
mmu-r	ENSMU	294	-28.4	152	-16.9	5522	686
mmu-r	ENSMU	294	-28.2	154	-14.7	3563	10422
mmu-r	Gm388	294	-28	148	-14.8	559	6374
mmu-r	ENSMU	294	-27.7	154	-15.7	4125	11647
mmu-r	ENSMU	294	-27.7	154	-15.7	4125	11647
mmu-r	ENSMU	294	-27.5	151	-17.4	671	7576
mmu-r	ENSMU	294	-27.1	152	-17.8	7452	5359
mmu-r	ENSMU	294	-26.9	150	-13.7	4867	3580
mmu-r	ENSMU	294	-26.8	153	-16.8	3354	4318
mmu-r	ENSMU	294	-26.6	148	-14.7	8125	10978
mmu-r	ENSMU	294	-26.1	152	-15.5	13073	12149
mmu-r	ENSMU	294	-25.9	154	-13.8	1099	2928
mmu-r	ENSMU	294	-25.3	152	-12.9	4266	2203
mmu-r	ENSMU	294	-25.3	153	-16.1	3354	4318
mmu-r	ENSMU	294	-23.2	148	-13.1	1242	6077
mmu-r	ENSMU	294	-22.3	154	-12.5	12927	9638
mmu-r	ENSMU	294	-21.5	150	-11.7	852	2027
mmu-r	Gm388	293	-49	147	-24.9	1121	8244
mmu-r	ENSMU	293	-48.5	151	-28.1	7201	6975
mmu-r	ENSMU	293	-44	149	-23.7	6131	7383
mmu-r	ENSMU	293	-43.9	150	-24.7	4130	495
mmu-r	ENSMU	293	-42.6	149	-25.3	2424	676
mmu-r	ENSMU	293	-42.5	147	-21.5	7377	579
mmu-r	ENSMU	293	-42.3	151	-24.8	7376	574
mmu-r	ENSMU	293	-40.9	147	-21.7	4297	12352
mmu-r	Gm388	293	-40.7	153	-21.5	3570	3578
mmu-r	ENSMU	293	-39.8	147	-21.5	4143	6618
mmu-r	ENSMU	293	-39.7	153	-23.6	6129	7377
mmu-r	Gm412	293	-39.1	153	-25.6	2589	4410
mmu-r	ENSMU	293	-39	151	-20.4	4126	5259
mmu-r	ENSMU	293	-38.5	153	-20.6	4196	1324
mmu-r	ENSMU	293	-38.5	147	-20.9	2748	2428
mmu-r	ENSMU	293	-37.3	151	-22.3	1740	10835
mmu-r	ENSMU	293	-37.3	151	-21.6	10334	5082
mmu-r	ENSMU	293	-36.6	151	-23.8	2448	2104
hiv1-m	ENSMU	293	-36.1	149	-18.3	419	1531
mmu-r	ENSMU	293	-35.7	153	-21.3	1345	1541
rco-mi	ENSMU	293	-34.9	150	-19.6	182	4114
mmu-r	ENSMU	293	-34.5	152	-20.1	5726	606
mmu-r	Gm388	293	-34	150	-20	6208	8472
mmu-r	Gm412	293	-33.6	149	-19	2925	4089
mmu-r	ENSMU	293	-33.5	148	-17.2	14122	6742
mmu-r	ENSMU	293	-31.8	150	-16.6	10218	6577
mmu-r	ENSMU	293	-31.7	149	-18.8	5877	3960
mmu-r	ENSMU	293	-31.2	153	-19.1	6883	9358

mmu-r	ENSMU	293	-31.1	152	-18.2	3858	10888
mmu-r	ENSMU	293	-31	147	-16.2	781	1359
mmu-r	ENSMU	293	-30.8	151	-17.9	2515	5922
mmu-r	Gm388	293	-30.4	148	-15.7	2733	3343
mmu-r	ENSMU	293	-30.3	148	-16.4	7079	7061
mmu-r	ENSMU	293	-30.3	148	-15.2	2308	3962
mmu-r	ENSMU	293	-30.1	148	-17	4241	2095
mmu-r	ENSMU	293	-29.9	153	-16.1	1055	3799
mmu-r	ENSMU	293	-29.9	147	-16.7	6782	8153
mmu-r	ENSMU	293	-29.3	148	-15.1	3134	348
mmu-r	ENSMU	293	-28	150	-14.1	7466	7882
mmu-r	ENSMU	293	-27.8	147	-14.6	6782	8153
mmu-r	ENSMU	293	-27.7	151	-15.3	12709	221
mmu-r	ENSMU	293	-27.5	148	-14.7	2756	3335
mmu-r	ENSMU	293	-27.1	151	-13.9	3120	9195
mmu-r	ENSMU	293	-26.7	150	-16	4635	6353
mmu-r	ENSMU	293	-26.3	149	-14	10946	5610
mmu-r	ENSMU	293	-26.2	150	-16.7	4790	4910
mmu-r	ENSMU	293	-25.9	152	-13.3	2118	1875
mmu-r	ENSMU	293	-25.4	148	-15	7439	184
mmu-r	ENSMU	293	-25.1	150	-13.3	5310	6322
mmu-r	ENSMU	293	-23.9	149	-12.6	866	7614
mmu-r	ENSMU	293	-23.7	148	-12.4	7958	1961
mmu-r	Gm388	293	-23.5	147	-12.4	7852	760
mmu-r	ENSMU	293	-23.4	149	-12.1	6529	5772
mmu-r	ENSMU	293	-22.8	148	-13.9	339	1806
mmu-r	ENSMU	293	-22.7	151	-14.1	2486	11860
mmu-r	ENSMU	293	-21	153	-12.3	7031	6602
mmu-r	ENSMU	293	-21	149	-13.2	1697	2078
mmu-r	ENSMU	292	-50.6	149	-25.5	12407	6299
mmu-r	ENSMU	292	-45.6	152	-26.9	5067	7897
mmu-r	ENSMU	292	-45.5	147	-24.6	2728	3043
mmu-r	ENSMU	292	-45	146	-24.4	1333	7200
rco-mi	Gm388	292	-44.1	147	-23.1	8623	1012
mmu-r	ENSMU	292	-43.6	148	-22.2	1256	2394
mmu-r	Gm388	292	-42.4	151	-21.7	8474	6210
mmu-r	ENSMU	292	-41.7	152	-26.9	4296	5219
mmu-r	ENSMU	292	-40.5	148	-22.7	6164	7500
mmu-r	Gm388	292	-40.4	149	-25.4	4535	2814
mmu-r	ENSMU	292	-40.3	150	-20.8	1277	3289
mmu-r	ENSMU	292	-40	149	-20	3815	435
mmu-r	ENSMU	292	-39.7	150	-21.7	9124	1687
mmu-r	ENSMU	292	-38.7	147	-19.4	3813	437
mmu-r	ENSMU	292	-38.6	152	-19.9	1243	211
mmu-r	ENSMU	292	-38.4	148	-22	833	287
mmu-r	ENSMU	292	-38	152	-25.5	4542	6019
mmu-r	ENSMU	292	-37.5	150	-20.4	8384	1259
mmu-r	ENSMU	292	-37.3	150	-19.3	3489	4021
mmu-r	ENSMU	292	-36.5	152	-21.5	6833	5800
mmu-r	ENSMU	292	-36.4	147	-19.4	1705	1473
mmu-r	ENSMU	292	-36.4	148	-19	527	6975
mmu-r	ENSMU	292	-36.2	152	-19.7	10550	13508
mmu-r	ENSMU	292	-36.1	149	-18.8	11234	12616
mmu-r	ENSMU	292	-36.1	146	-18.3	969	4007
mmu-r	ENSMU	292	-35.9	152	-19	2035	2
mmu-r	Gm388	292	-35.4	147	-19.1	7483	740
mmu-r	ENSMU	292	-35.2	149	-18.8	12409	6306
mmu-r	Gm388	292	-34.5	150	-17.8	634	7648
mmu-r	ENSMU	292	-34.1	152	-17.4	3950	512
mmu-r	ENSMU	292	-33.8	150	-16.9	1832	496
mmu-r	ENSMU	292	-33.7	148	-18.7	5065	3114
mmu-r	ENSMU	292	-33.4	152	-17.5	2630	8970
mmu-r	ENSMU	292	-32.3	146	-16.1	7698	7782
mmu-r	ENSMU	292	-31.8	148	-17.7	5119	6525

mmu-r	ENSMU	292	-31.6	152	-17.3	4285	3594
mmu-r	ENSMU	292	-31.6	151	-17.1	2662	2315
mmu-r	ENSMU	292	-31.5	147	-16.9	5563	5158
mmu-r	ENSMU	292	-31.3	149	-16.6	2675	6009
mmu-r	ENSMU	292	-30.6	152	-18.2	3858	10887
mmu-r	ENSMU	292	-30.4	148	-15.3	531	6979
mmu-r	ENSMU	292	-30.2	147	-16.2	795	7252
mmu-r	Gm388	292	-30	148	-16.5	4339	5410
mmu-r	ENSMU	292	-30	148	-15.5	4747	6661
mmu-r	ENSMU	292	-29.7	152	-17.9	1625	7916
mmu-r	ENSMU	292	-29.2	147	-16.2	4494	2253
mmu-r	Gm388	292	-29.2	151	-16	4439	4821
mmu-r	ENSMU	292	-28.8	152	-16	32	1932
mmu-r	ENSMU	292	-28.5	148	-17.3	4795	3155
mmu-r	Gm388	292	-27.8	147	-14.9	2712	6111
mmu-r	ENSMU	292	-27.4	147	-15.6	7990	13332
mmu-r	Gm388	292	-27.1	152	-15.7	7308	6756
mmu-r	ENSMU	292	-27.1	147	-14.7	729	1601
mmu-r	ENSMU	292	-27.1	152	-14.5	2767	193
mmu-r	ENSMU	292	-25.8	152	-14.1	7288	6530
mmu-r	ENSMU	292	-25.7	150	-14.2	11714	3023
mmu-r	Gm388	292	-25.5	146	-13.9	850	7313
mmu-r	ENSMU	292	-24.4	150	-14.2	13796	6580
mmu-r	ENSMU	292	-23.4	148	-12.7	2078	1693
mmu-r	ENSMU	292	-22.1	147	-11.5	6285	6383
mmu-r	ENSMU	292	-21.4	146	-11.4	9809	11781
mmu-r	ENSMU	292	-21.1	152	-15	4721	6704
mmu-r	ENSMU	292	-21	146	-10.9	4396	5714
mmu-r	ENSMU	292	-21	148	-11	5677	3958
mmu-r	ENSMU	291	-49.1	149	-25.9	1346	1320
mmu-r	ENSMU	291	-46.7	151	-28.5	4033	3472
mmu-r	Gm388	291	-45	148	-29.1	5183	227
mmu-r	Gm412	291	-44.5	147	-23.8	3961	3020
mmu-r	ENSMU	291	-43.8	146	-23.3	661	683
mmu-r	Gm388	291	-43.5	147	-22.5	1625	2373
mmu-r	Gm388	291	-43.4	150	-24.3	1161	5354
mmu-r	Gm388	291	-43	147	-23.6	222	5182
mmu-r	ENSMU	291	-40.9	149	-22	6614	6465
mmu-r	ENSMU	291	-39.4	147	-20.8	942	4603
mmu-r	ENSMU	291	-39.3	146	-20.3	5251	6302
mmu-r	Gm412	291	-39.2	151	-26.1	3976	715
mmu-r	Gm388	291	-38.4	151	-20.7	7893	5183
rco-mil	Gm388	291	-38.1	148	-23.5	1008	8626
rco-mil	Gm388	291	-38.1	148	-23.5	1008	8626
mmu-r	ENSMU	291	-37.7	151	-19.2	2344	6365
mmu-r	ENSMU	291	-37.6	146	-19.2	2166	138
mmu-r	ENSMU	291	-37.3	148	-23	5798	3389
mmu-r	Gm412	291	-34.5	151	-21.6	4120	3667
mmu-r	ENSMU	291	-34.2	149	-19.1	1343	1356
mmu-r	ENSMU	291	-33.9	146	-17	4702	9332
mmu-r	Gm388	291	-33.9	147	-18.7	6226	3306
mmu-r	ENSMU	291	-33.8	150	-17.8	7183	2144
mmu-r	ENSMU	291	-33.8	151	-21.3	8489	10005
mmu-r	ENSMU	291	-33.7	148	-17	5663	2273
mmu-r	ENSMU	291	-33.2	150	-19.2	446	1903
mmu-r	ENSMU	291	-33.1	148	-16.8	1338	1738
mmu-r	Gm388	291	-32.8	150	-17.8	3736	4149
mmu-r	ENSMU	291	-32.5	150	-16.5	2042	1467
mmu-r	ENSMU	291	-32	151	-20.8	6206	7579
mmu-r	ENSMU	291	-31.9	146	-18.2	6563	3885
mmu-r	ENSMU	291	-31.4	148	-17.7	8428	7126
rco-mil	ENSMU	291	-30.3	151	-15.5	621	4786
rco-mil	ENSMU	291	-30.3	151	-15.5	621	4786
mmu-r	ENSMU	291	-29.9	147	-15.9	6862	6904

mmu-r	ENSMU	291	-29.1	151	-17.1	1241	2387
mmu-r	ENSMU	291	-29	148	-18	7908	4864
mmu-r	ENSMU	291	-28.6	151	-18	8758	8892
mmu-r	ENSMU	291	-28.5	151	-15.3	1309	5775
mmu-r	ENSMU	291	-28.5	151	-15.3	1923	1638
mmu-r	ENSMU	291	-28.2	151	-15	4556	5603
mmu-r	ENSMU	291	-28.2	147	-14.3	4927	7552
mmu-r	ENSMU	291	-27.9	148	-14.5	9934	5756
mmu-r	ENSMU	291	-27.8	150	-15.4	12737	11328
mmu-r	ENSMU	291	-27.6	149	-14.3	1199	3618
mmu-r	ENSMU	291	-27.6	147	-14	3497	7017
mmu-r	Gm388	291	-27.1	147	-15.1	4439	9047
mmu-r	ENSMU	291	-26.9	151	-13.8	590	9108
mmu-r	ENSMU	291	-26.9	151	-13.8	590	9108
mmu-r	ENSMU	291	-26.9	151	-13.8	590	9108
mmu-r	ENSMU	291	-26.8	149	-15.2	7251	476
mmu-r	ENSMU	291	-26.6	151	-16.6	7108	14074
mmu-r	ENSMU	291	-26	149	-16.3	12926	13045
mmu-r	ENSMU	291	-25.5	150	-13.7	2954	3661
mmu-r	ENSMU	291	-24.6	148	-14.4	4033	8735
mmu-r	ENSMU	291	-24.2	151	-13.6	1051	135
mmu-r	ENSMU	291	-24.1	148	-13.6	1790	9714
mmu-r	ENSMU	291	-23.7	151	-14.7	3313	4781
mmu-r	ENSMU	291	-23.4	151	-14.2	3093	1664
mmu-r	ENSMU	291	-23.2	151	-12.2	1394	10380
mmu-r	ENSMU	291	-23	147	-12.1	6464	2217
mmu-r	ENSMU	291	-22.9	150	-12.5	3882	534
mmu-r	ENSMU	291	-22.7	149	-14.1	6153	13339
mmu-r	ENSMU	291	-22.6	146	-12.1	11760	11479
mmu-r	ENSMU	291	-22.5	151	-16.5	4823	2246
mmu-r	ENSMU	291	-21.6	150	-12.2	3882	534
mmu-r	ENSMU	291	-20.7	151	-12.3	4425	3932
mmu-r	ENSMU	291	-20.2	146	-12.1	1145	3899
mmu-r	ENSMU	290	-48.6	147	-28.3	558	1770
mmu-r	ENSMU	290	-43.9	146	-22.8	7660	666
mmu-r	Gm388	290	-41.5	150	-24.9	407	3994
mmu-r	ENSMU	290	-41.2	147	-21.3	1407	8052
mmu-r	Gm388	290	-40.5	150	-21.4	3402	6942
mmu-r	ENSMU	290	-38.5	145	-21.7	1885	3936
mmu-r	ENSMU	290	-36.1	149	-20.5	3633	11710
mmu-r	ENSMU	290	-35.7	150	-20.5	289	5478
mmu-r	Gm388	290	-35.3	149	-21.8	2149	3180
mmu-r	Gm388	290	-34.9	150	-18.8	8470	6207
mmu-r	ENSMU	290	-34.7	146	-17.4	8871	10865
mmu-r	ENSMU	290	-34.6	150	-20.8	637	7086
mmu-r	ENSMU	290	-34.1	147	-21.9	5056	6944
mmu-r	ENSMU	290	-33.1	150	-16.8	8876	8583
mmu-r	ENSMU	290	-32.9	148	-17.7	11984	12119
mmu-r	Gm388	290	-32.5	148	-19.8	3019	8841
mmu-r	ENSMU	290	-31.8	145	-15.9	7698	7782
mmu-r	ENSMU	290	-31.8	146	-20.2	7832	9482
mmu-r	ENSMU	290	-31.8	150	-17.4	3952	3972
mmu-r	ENSMU	290	-31.6	149	-20.4	1846	966
mmu-r	ENSMU	290	-31.2	150	-20.9	7060	5025
mmu-r	Gm388	290	-31	150	-17	4440	9048
mmu-r	ENSMU	290	-30.4	146	-15.5	5727	3580
mmu-r	Gm388	290	-29.2	145	-15.5	2191	8284
mmu-r	ENSMU	290	-29.1	146	-15.8	7514	188
mmu-r	ENSMU	290	-28.9	145	-16.4	4338	7745
mmu-r	ENSMU	290	-28.6	150	-17.4	8561	4589
mmu-r	Gm388	290	-28.4	147	-14.4	5293	7051
mmu-r	ENSMU	290	-28.3	145	-17.3	1049	1176
mmu-r	ENSMU	290	-28.1	150	-16.6	7234	11532
mmu-r	ENSMU	290	-28.1	146	-14.5	810	8188

mmu-r	ENSMU	290	-28	150	-19.1	4805	1023
mmu-r	Gm412	290	-27.8	148	-17.2	4218	2966
mmu-r	ENSMU	290	-27.5	148	-14.3	6566	1908
mmu-r	ENSMU	290	-27.5	150	-15.7	3451	12810
mmu-r	ENSMU	290	-27.5	145	-13.7	7361	7860
mmu-r	ENSMU	290	-27.1	148	-18.3	12745	7327
mmu-r	ENSMU	290	-27	147	-16.4	5385	4192
mmu-r	Gm388	290	-26.6	150	-16.5	4440	9048
mmu-r	ENSMU	290	-26.6	148	-15.4	12093	9361
mmu-r	ENSMU	290	-26.6	147	-14	1107	9801
mmu-r	ENSMU	290	-26.5	146	-14.8	9175	3268
mmu-r	ENSMU	290	-26.2	147	-13.8	4284	5105
mmu-r	Gm388	290	-26.1	146	-14.9	2694	1872
mmu-r	ENSMU	290	-25.7	150	-13.8	4796	3153
mmu-r	ENSMU	290	-25.3	150	-14.7	11905	11008
mmu-r	Gm388	290	-25.2	150	-15.1	4441	9049
mmu-r	ENSMU	290	-24.8	150	-12.6	4796	3153
mmu-r	ENSMU	290	-22.6	150	-12.1	5511	4530
mmu-r	ENSMU	290	-22.5	146	-11.7	908	1752
mmu-r	ENSMU	290	-21.8	148	-13.5	3551	2089
mmu-r	Gm388	290	-21.1	150	-12.9	4137	8455
mmu-r	ENSMU	290	-20	150	-10.7	7442	184
mmu-r	ENSMU	289	-45.1	147	-22.7	6402	718
mmu-r	ENSMU	289	-44.5	148	-25.7	5061	5080
mmu-r	ENSMU	289	-43.9	149	-22	4186	4214
mmu-r	ENSMU	289	-42.3	145	-23.9	13900	1993
hiv1-m	Gm412	289	-39.7	148	-20.2	2025	2866
mmu-r	Gm412	289	-39.3	145	-20.2	4060	4487
mmu-r	ENSMU	289	-38.5	149	-21.6	8164	3091
mmu-r	ENSMU	289	-38.2	147	-20.7	3935	588
mmu-r	ENSMU	289	-38	149	-21.9	2	10353
mmu-r	ENSMU	289	-37.6	146	-20.2	1692	9127
mmu-r	ENSMU	289	-37	146	-20.6	3742	3607
mmu-r	Gm388	289	-36.8	146	-18.6	3544	7066
mmu-r	Gm412	289	-36.7	149	-19.9	3564	2716
mmu-r	ENSMU	289	-35.6	147	-18.4	3302	3931
mmu-r	ENSMU	289	-34.6	147	-18.5	59	5272
mmu-r	ENSMU	289	-34.4	149	-18.4	1471	3270
mmu-r	ENSMU	289	-34.1	148	-18	1	7983
mmu-r	ENSMU	289	-31.2	147	-16.4	3858	10888
mmu-r	ENSMU	289	-30.7	147	-15.4	3215	1475
mmu-r	ENSMU	289	-30.6	146	-15.6	12651	11623
mmu-r	ENSMU	289	-30.5	146	-16.5	293	3897
mmu-r	ENSMU	289	-30.1	146	-15.7	8248	12665
mmu-r	ENSMU	289	-29.6	147	-15.9	574	2076
mmu-r	ENSMU	289	-29.6	147	-15.9	574	2076
mmu-r	ENSMU	289	-29.6	147	-15.9	574	2076
mmu-r	ENSMU	289	-29.3	145	-16.6	7217	8281
mmu-r	ENSMU	289	-27.5	148	-14.3	9774	12377
mmu-r	ENSMU	289	-27.4	147	-14.2	1224	5326
mmu-r	ENSMU	289	-27	145	-14.5	2607	683
mmu-r	ENSMU	289	-26.9	148	-16	6503	1931
mmu-r	Gm388	289	-26.7	147	-14.5	1952	8180
mmu-r	ENSMU	289	-26	148	-13.8	11029	10862
mmu-r	Gm412	289	-25.6	148	-13.5	655	33
mmu-r	ENSMU	289	-24.8	149	-12.5	3726	150
mmu-r	ENSMU	289	-24.3	149	-12.8	2021	1059
mmu-r	Gm388	289	-24.1	145	-12.6	1796	7042
mmu-r	Gm388	289	-24.1	149	-14.3	8734	100
mmu-r	ENSMU	289	-23.8	146	-15.5	4929	1585
mmu-r	Gm388	289	-23.8	147	-14.8	2603	3448
mmu-r	ENSMU	289	-23	149	-16.1	4429	3087
mmu-r	ENSMU	289	-23	146	-13.9	9982	12401
mmu-r	ENSMU	289	-22.4	149	-11.3	6981	6076

mmu-r	ENSMU	289	-21.6	149	-13.1	5151	774
mmu-r	ENSMU	289	-21.4	145	-13.1	10002	8340
mmu-r	ENSMU	288	-46	148	-24.6	791	9581
mmu-r	ENSMU	288	-44.9	146	-27.9	6089	8508
mmu-r	Gm412	288	-44.3	145	-22.7	4060	4487
mmu-r	ENSMU	288	-43.7	148	-23.5	10431	10797
mmu-r	ENSMU	288	-43.6	148	-27.8	6832	5800
mmu-r	ENSMU	288	-39.1	148	-21.1	7022	11547
mmu-r	ENSMU	288	-39	145	-21	697	2108
mmu-r	ENSMU	288	-38.6	148	-20.3	6133	7379
mmu-r	Gm388	288	-38.4	146	-20.5	7486	7361
mmu-r	ENSMU	288	-38.4	148	-19.4	942	4603
mmu-r	ENSMU	288	-38.3	146	-22.2	6518	7739
mmu-r	ENSMU	288	-38	148	-19.5	1552	6671
mmu-r	ENSMU	288	-36.9	145	-18.7	487	2171
mmu-r	ENSMU	288	-35.4	147	-19.9	6727	6418
mmu-r	ENSMU	288	-35	146	-18	2178	470
mmu-r	ENSMU	288	-34.9	144	-20.2	3481	8011
mmu-r	ENSMU	288	-34.1	148	-17.4	3950	512
mmu-r	Gm388	288	-33.9	147	-20.6	5092	5335
mmu-r	ENSMU	288	-32.8	148	-16.8	3793	921
mmu-r	ENSMU	288	-32.8	148	-16.8	3793	921
mmu-r	ENSMU	288	-32.8	148	-16.8	3793	921
mmu-r	ENSMU	288	-32.7	146	-17.2	5627	4438
mmu-r	ENSMU	288	-32.6	148	-16.3	10243	3684
mmu-r	ENSMU	288	-32.5	148	-18	1418	7155
mmu-r	ENSMU	288	-32.2	147	-16.3	4645	3008
mmu-r	ENSMU	288	-32.2	148	-17.3	10102	1157
mmu-r	ENSMU	288	-32	144	-18.8	1018	5839
mmu-r	Gm388	288	-32	148	-17.4	6856	79
hiv1-m	ENSMU	288	-31.9	146	-17.5	9046	408
mmu-r	ENSMU	288	-31.7	148	-18.7	1556	6675
mmu-r	ENSMU	288	-31.5	146	-18.7	2155	3320
mmu-r	ENSMU	288	-31.4	145	-16.4	5726	4444
rco-mil	ENSMU	288	-31.4	146	-16.4	7218	7871
mmu-r	Gm388	288	-30.7	146	-15.7	8685	1227
mmu-r	ENSMU	288	-30.7	146	-16.7	1240	338
mmu-r	ENSMU	288	-30.4	145	-16.5	3595	4284
mmu-r	ENSMU	288	-30	148	-15.5	7314	3750
rco-mil	ENSMU	288	-29.9	146	-17.2	7219	7872
rco-mil	ENSMU	288	-29.9	146	-17.2	7219	7872
mmu-r	ENSMU	288	-29.8	148	-18.4	8105	8914
mmu-r	ENSMU	288	-29.6	148	-15.1	5065	3117
mmu-r	Gm388	288	-29.1	147	-15.6	3862	4458
mmu-r	Gm388	288	-28.9	146	-14.9	1336	3738
mmu-r	ENSMU	288	-28.5	146	-14.3	7711	3126
mmu-r	Gm388	288	-28.4	145	-15.6	2501	6609
mmu-r	Gm412	288	-28.4	146	-14.4	4316	2011
mmu-r	Gm388	288	-28	148	-17.8	8737	100
mmu-r	ENSMU	288	-28	146	-14.3	7711	3126
mmu-r	ENSMU	288	-27.9	148	-15.9	3100	3837
mmu-r	ENSMU	288	-27.3	148	-16	3154	4797
mmu-r	Gm388	288	-26.9	146	-14.4	7419	5145
mmu-r	ENSMU	288	-26.9	145	-14	3806	483
mmu-r	ENSMU	288	-26.5	148	-15.6	2395	1548
mmu-r	ENSMU	288	-26.5	148	-14.1	7776	848
mmu-r	ENSMU	288	-24.7	145	-12.7	9931	5754
mmu-r	ENSMU	288	-24.6	144	-13.2	6711	14001
mmu-r	ENSMU	288	-24.3	145	-13.9	5778	3688
mmu-r	ENSMU	288	-24	145	-15.3	12273	2849
mmu-r	Gm412	288	-23.3	145	-12.4	1293	669
mmu-r	ENSMU	288	-23.1	147	-14.2	13956	1638
mmu-r	ENSMU	288	-22	148	-11.4	7102	7179
mmu-r	Gm388	288	-22	145	-11.4	6161	4639

mmu-r	ENSMU	288	-21.7	144	-13.1	8440	11068
mmu-r	ENSMU	288	-21.7	145	-11	3404	9642
mmu-r	ENSMU	288	-20.9	147	-12.2	1653	2013
mmu-r	ENSMU	288	-20.9	144	-12.1	10348	11077
rco-mil	ENSMU	288	-20.5	148	-12.8	1799	2580
rco-mil	ENSMU	288	-20.5	148	-12.8	1799	2580
mmu-r	ENSMU	287	-39.2	146	-27.4	5164	10196
mmu-r	ENSMU	287	-38.5	147	-21.1	4068	1059
mmu-r	ENSMU	287	-38.4	146	-23.4	1010	1636
mmu-r	Gm388	287	-35.7	147	-21.7	6201	8470
mmu-r	ENSMU	287	-34.6	145	-17.5	2402	2831
rco-mil	ENSMU	287	-34.2	147	-17.4	8637	2429
mmu-r	Gm412	287	-34	147	-20.1	3200	1629
mmu-r	ENSMU	287	-33.4	145	-19.4	3367	5886
mmu-r	ENSMU	287	-31.9	147	-16.8	5	10354
mmu-r	ENSMU	287	-31.2	147	-18.8	8145	2632
mmu-r	ENSMU	287	-31	147	-16.7	4893	2460
mmu-r	ENSMU	287	-30.8	147	-17	3956	488
mmu-r	ENSMU	287	-30.7	146	-15.5	4550	10428
mmu-r	ENSMU	287	-29.8	145	-16.6	113	2308
mmu-r	ENSMU	287	-27.6	144	-15.7	6720	7935
mmu-r	ENSMU	287	-27	147	-13.7	4203	2259
mmu-r	ENSMU	287	-26.8	145	-13.5	3497	7021
mmu-r	ENSMU	287	-26.6	147	-17.5	850	5
mmu-r	ENSMU	287	-26.5	146	-15.8	3125	10648
mmu-r	ENSMU	287	-26.3	145	-13.8	3480	1694
mmu-r	ENSMU	287	-25	147	-13.3	1056	4119
mmu-r	ENSMU	287	-24.8	144	-13	2675	6009
mmu-r	ENSMU	287	-24.8	147	-14.7	3534	5179
mmu-r	ENSMU	287	-24.4	144	-13.5	6720	7935
mmu-r	ENSMU	287	-24.3	147	-12.4	7620	481
mmu-r	ENSMU	287	-23.6	147	-14.6	1718	4407
mmu-r	ENSMU	287	-23.3	147	-12.3	5727	4445
mmu-r	ENSMU	287	-22.1	147	-12.6	4018	9283
mmu-r	ENSMU	287	-21.9	147	-12.1	3467	4070
mmu-r	Gm388	286	-41.7	144	-25.2	1280	1626
mmu-r	ENSMU	286	-41.5	146	-22.5	12496	10093
mmu-r	Gm388	286	-40	144	-22.4	5179	218
mmu-r	ENSMU	286	-39.7	145	-22.8	3277	173
mmu-r	ENSMU	286	-38.7	146	-20.7	11985	12118
mmu-r	ENSMU	286	-38.1	145	-21.9	5770	6007
mmu-r	ENSMU	286	-37.7	143	-20.1	275	2725
mmu-r	ENSMU	286	-37	145	-22.7	9801	1107
mmu-r	ENSMU	286	-36.4	145	-19.8	3207	7529
mmu-r	ENSMU	286	-35.6	146	-24.4	8617	2592
mmu-r	Gm388	286	-35.5	146	-17.8	6634	5467
mmu-r	ENSMU	286	-35.2	146	-26.5	6267	2108
mmu-r	ENSMU	286	-34.8	146	-18.3	2009	8673
mmu-r	ENSMU	286	-34	146	-19.5	4246	1527
mmu-r	ENSMU	286	-33.2	144	-17.2	1018	4803
mmu-r	ENSMU	286	-32.9	146	-17.9	4575	6408
mmu-r	Gm412	286	-32.9	146	-17.2	2546	177
mmu-r	ENSMU	286	-31.3	143	-17.4	4250	6134
mmu-r	ENSMU	286	-31	144	-16.7	1517	1274
mmu-r	ENSMU	286	-28.3	143	-14.5	5756	9934
mmu-r	ENSMU	286	-28.2	144	-16.4	9927	10124
mmu-r	Gm412	286	-27.8	146	-14.2	1703	4536
mmu-r	ENSMU	286	-27.3	143	-14.4	7058	7082
mmu-r	ENSMU	286	-27.1	146	-14.6	7514	9812
mmu-r	ENSMU	286	-27.1	143	-13.8	6597	13939
mmu-r	ENSMU	286	-27	143	-13.6	701	8101
mmu-r	ENSMU	286	-26.8	145	-15.7	4854	367
mmu-r	ENSMU	286	-26.7	143	-14.8	6721	7936
mmu-r	ENSMU	286	-26	146	-13	14074	7818

mmu-r	ENSMU	286	-25.7	143	-13.1	6359	12954
mmu-r	ENSMU	286	-25.6	146	-17	12031	7173
mmu-r	ENSMU	286	-25.5	145	-14.7	1920	4009
mmu-r	ENSMU	286	-25.1	145	-13.4	548	1616
rco-mil	ENSMU	286	-24.7	146	-13.3	6211	3175
mmu-r	ENSMU	286	-24.6	145	-15.1	1150	3855
mmu-r	Gm388	286	-23.9	146	-13.1	7751	525
mmu-r	Gm388	286	-23.9	145	-12.8	2571	4837
mmu-r	ENSMU	286	-23.9	146	-12.4	6707	8858
mmu-r	ENSMU	286	-22.5	146	-12.6	3727	1554
mmu-r	Gm388	286	-22.4	146	-12.4	2704	2978
mmu-r	ENSMU	286	-22.1	145	-12	1166	6211
mmu-r	ENSMU	286	-20.7	146	-11.3	9457	10339
mmu-r	Gm388	286	-20.5	144	-11.2	4825	8965
mmu-r	ENSMU	286	-20.4	143	-10.7	6359	12954
mmu-r	Gm412	286	-20.3	146	-10.7	919	1869
mmu-r	ENSMU	285	-42.9	144	-24.6	7380	6133
mmu-r	ENSMU	285	-41.8	145	-20.9	9034	13508
mmu-r	Gm388	285	-40.8	145	-22.1	5180	223
mmu-r	ENSMU	285	-40.2	144	-20.5	3609	2621
mmu-r	ENSMU	285	-39.5	145	-22.7	7058	6969
mmu-r	ENSMU	285	-38.4	144	-19.2	12533	731
mmu-r	ENSMU	285	-38.2	143	-19.5	3381	6848
mmu-r	ENSMU	285	-37.8	145	-19.6	9156	64
mmu-r	ENSMU	285	-36	144	-18.7	10107	7985
mmu-r	ENSMU	285	-35.1	145	-19	6084	5209
mmu-r	ENSMU	285	-33.8	145	-17.4	9630	9035
mmu-r	ENSMU	285	-33.4	145	-17.1	5040	3629
mmu-r	ENSMU	285	-33.3	145	-17.5	3339	6853
rco-mil	ENSMU	285	-31	145	-16.4	5971	6007
mmu-r	ENSMU	285	-30.3	145	-17	3957	5678
rco-mil	Gm388	285	-30.1	145	-19.5	711	1946
mmu-r	ENSMU	285	-29.7	143	-18.7	1069	5747
mmu-r	ENSMU	285	-28.7	145	-16.7	9	4850
mmu-r	Gm412	285	-28.4	145	-16.6	3087	2789
mmu-r	Gm388	285	-28.2	145	-14.6	2122	5448
mmu-r	Gm388	285	-27.9	145	-17.9	3469	5098
mmu-r	Gm388	285	-27.4	144	-14.6	336	3418
mmu-r	Gm412	285	-27.3	143	-15.6	1112	3642
mmu-r	ENSMU	285	-27.2	145	-14.6	2620	3858
mmu-r	ENSMU	285	-26.7	143	-13.6	3361	6861
mmu-r	ENSMU	285	-26.1	145	-16.3	5767	4400
mmu-r	Gm388	285	-26.1	145	-15.6	2883	2316
rco-mil	ENSMU	285	-24.6	145	-12.8	6212	3175
rco-mil	ENSMU	285	-24.6	145	-12.8	6212	3175
rco-mil	ENSMU	285	-24.6	145	-12.8	6212	3175
rco-mil	ENSMU	285	-24.6	145	-12.8	6212	3175
mmu-r	Gm388	285	-24.5	145	-12.3	4700	8657
mmu-r	ENSMU	285	-23	145	-14	1587	4933
mmu-r	ENSMU	285	-22.7	144	-11.6	9639	10388
mmu-r	Gm412	285	-22.4	145	-11.4	1149	3366
mmu-l	ENSMU	285	-22.2	145	-11.7	5849	6374
mmu-r	ENSMU	285	-21.6	145	-12.6	8629	3115
mmu-r	ENSMU	284	-41.3	144	-23.3	563	5941
mmu-r	Gm412	284	-38.4	144	-19.6	688	572
mmu-r	ENSMU	284	-35.6	144	-18.8	172	3213
mmu-r	ENSMU	284	-35.2	144	-19.2	3682	10241
mmu-r	ENSMU	284	-33.3	143	-16.7	1101	6718
mmu-r	ENSMU	284	-33.3	144	-17.4	25	4282
mmu-r	ENSMU	284	-33.2	144	-18.7	6898	5269
mmu-r	ENSMU	284	-33.1	144	-17.6	2256	1315
mmu-r	Gm388	284	-32.6	144	-18.1	7441	1754
mmu-r	ENSMU	284	-32.2	144	-18.9	13514	3108
mmu-r	Gm388	284	-30.9	144	-19.1	1494	8712

mmu-r	Gm388	284	-30.7	144	-17.5	6542	4167
mmu-r	ENSMU	284	-30.7	144	-15.6	2196	3193
mmu-r	ENSMU	284	-30.4	143	-17.5	5872	6590
mmu-r	ENSMU	284	-30.1	144	-15.5	3401	1347
mmu-r	ENSMU	284	-29.8	144	-18	8644	4302
mmu-r	ENSMU	284	-29.5	144	-17	1071	4720
mmu-r	ENSMU	284	-28.4	144	-16.3	622	4788
mmu-r	ENSMU	284	-28.3	144	-16.7	6121	9201
mmu-r	ENSMU	284	-28.2	143	-14.9	8255	8107
mmu-r	ENSMU	284	-27.1	144	-17.6	7915	5975
mmu-r	Gm412	284	-25.4	144	-14.6	4260	3920
mmu-r	ENSMU	284	-25	144	-15.9	556	3136
mmu-r	ENSMU	284	-24.7	144	-15.4	3154	4797
mmu-r	ENSMU	284	-24	144	-13.8	5257	484
mmu-r	ENSMU	284	-24	142	-13.1	7601	8467
mmu-r	Gm388	284	-20.7	143	-11.6	3245	5024
mmu-r	ENSMU	283	-35.7	142	-18.1	4043	8796
mmu-r	ENSMU	283	-33.1	142	-18.7	3317	2498
mmu-r	ENSMU	283	-32.2	143	-17.4	2008	1074
mmu-r	ENSMU	283	-30.4	143	-17	11975	11381
mmu-r	ENSMU	283	-29.2	143	-16.5	6030	4855
mmu-r	ENSMU	283	-28.8	142	-15.4	7568	2286
mmu-r	Gm388	283	-25.7	143	-14.8	6038	2853
mmu-r	ENSMU	283	-24.7	143	-13.1	3687	1209
mmu-r	Gm388	283	-24.5	143	-13.2	8119	321
mmu-r	ENSMU	283	-22.7	142	-11.5	2686	8665
mmu-r	ENSMU	283	-22	143	-12.2	13670	10569
mmu-r	ENSMU	282	-46.3	142	-24.3	2009	1073
mmu-r	ENSMU	282	-44.5	141	-26	9692	14403
mmu-r	Gm388	282	-41.1	142	-21.2	15	4475
mmu-r	Gm388	282	-39.2	142	-22.2	4452	5429
mmu-r	ENSMU	282	-38	141	-21.2	711	946
mmu-r	ENSMU	282	-38	141	-19.3	591	3937
mmu-r	ENSMU	282	-35.1	141	-17.7	363	2925
mmu-r	ENSMU	282	-33.3	142	-17.4	3677	2245
mmu-r	ENSMU	282	-31.6	142	-15.8	2469	4992
mmu-r	ENSMU	282	-30.8	142	-16.1	3595	4007
mmu-r	Gm412	282	-30.8	142	-16.4	3688	4123
mmu-r	Gm388	282	-29.4	142	-15.9	546	2242
mmu-r	ENSMU	282	-29.3	142	-17	399	355
mmu-r	Gm388	282	-29.2	142	-14.7	5326	3950
mmu-r	ENSMU	282	-27.1	141	-14.9	6882	10944
mmu-r	ENSMU	282	-26.1	142	-13.3	2010	8674
mmu-r	ENSMU	282	-25.7	142	-15.1	852	2028
mmu-r	ENSMU	282	-25.5	142	-14.6	2210	3551
mmu-r	Gm388	282	-22.5	142	-12.4	832	7338
mmu-r	ENSMU	282	-20.2	142	-11	10101	9653
mmu-r	ENSMU	281	-39.7	141	-22.1	6968	11468
mmu-r	ENSMU	281	-34.8	141	-18.3	3108	13516
mmu-r	ENSMU	281	-34.5	141	-18.5	10349	4353
rco-mil	ENSMU	281	-32.6	141	-17.1	8639	2429
rco-mil	ENSMU	281	-32.6	141	-17.1	8639	2429
mmu-r	ENSMU	281	-32.2	141	-16.8	5346	5552
mmu-r	ENSMU	281	-32	141	-17.3	2009	1075
mmu-r	ENSMU	281	-27.9	141	-17.5	852	2028
mmu-r	ENSMU	281	-27.2	141	-15	7989	10108
mmu-r	Gm388	281	-27.1	141	-15.7	8118	321
mmu-r	ENSMU	281	-24.9	141	-14.5	553	3136
mmu-r	ENSMU	281	-24.6	141	-14.3	11569	3579
mmu-r	ENSMU	281	-23.1	141	-13	10828	11275
mmu-r	Gm412	281	-21	141	-10.7	1128	1489
mmu-r	Gm388	280	-33.4	140	-17.7	3386	4362
mmu-r	ENSMU	280	-33	140	-16.5	7528	8189
mmu-r	ENSMU	280	-32.2	140	-16.1	7694	7778

mmu-r	Gm388	280	-30.9	140	-17.8	6208	8494
mmu-r	ENSMU	280	-30.6	140	-15.8	2250	3223
mmu-r	ENSMU	280	-29.7	140	-18.1	637	6145
mmu-r	ENSMU	280	-29.1	140	-15.1	452	8445
mmu-r	ENSMU	280	-29	140	-17.2	64	9156
rco-mil	ENSMU	280	-29	140	-15.6	4164	4869
mmu-r	ENSMU	280	-28.6	140	-15.5	2458	4892
mmu-r	ENSMU	280	-27.9	140	-16.1	1477	3217
rco-mil	ENSMU	280	-26.4	140	-13.3	2115	7814
mmu-r	Gm412	280	-25.8	140	-13.7	1541	1589
mmu-r	ENSMU	280	-25.7	140	-12.9	572	5355
mmu-r	ENSMU	280	-25.5	140	-12.8	1974	10855
mmu-r	ENSMU	280	-24.5	140	-12.3	10652	10659
mmu-r	ENSMU	280	-24.1	140	-13.3	677	4596
mmu-r	ENSMU	280	-22.1	140	-12.6	5579	5614
mmu-r	Gm388	280	-22.1	140	-12	2578	3395
mmu-r	ENSMU	280	-20.9	140	-11.3	594	6531
mmu-r	ENSMU	280	-20.4	140	-11.6	3457	5712
mmu-r	ENSMU	280	-20.3	140	-10.7	3308	3933
mmu-r	ENSMU	197	-48.7	197	-48.7	8143	
mmu-r	ENSMU	197	-41.3	197	-41.3	1496	
mmu-r	Gm412	197	-33.8	197	-33.8	2181	
mmu-r	Gm388	196	-35.8	196	-35.8	1933	
mmu-r	ENSMU	196	-35.4	196	-35.4	3396	
mmu-r	ENSMU	192	-32.8	192	-32.8	13183	
mmu-r	ENSMU	191	-43.3	191	-43.3	714	
mmu-r	Gm388	191	-38.9	191	-38.9	1935	
mmu-r	ENSMU	190	-45.8	190	-45.8	715	
mmu-r	ENSMU	184	-29.7	184	-29.7	7437	
mmu-r	Gm388	179	-29.2	179	-29.2	7097	
mmu-r	ENSMU	179	-24.3	179	-24.3	1431	
mmu-r	Gm388	178	-35.5	178	-35.5	422	
mmu-r	ENSMU	178	-34.5	178	-34.5	8134	
mmu-r	ENSMU	178	-33.8	178	-33.8	8146	
mmu-r	ENSMU	178	-30.9	178	-30.9	6897	
mmu-r	ENSMU	178	-27.1	178	-27.1	1243	
mmu-r	ENSMU	177	-32.2	177	-32.2	1507	
mmu-r	ENSMU	177	-23.8	177	-23.8	3075	
mmu-r	ENSMU	177	-21.1	177	-21.1	3081	
mmu-r	ENSMU	176	-40.8	176	-40.8	594	
mmu-r	Gm388	176	-27.6	176	-27.6	3407	
mmu-r	ENSMU	175	-35.8	175	-35.8	5437	
mmu-r	ENSMU	175	-30.2	175	-30.2	3019	
mmu-r	ENSMU	175	-28.9	175	-28.9	424	
mmu-r	ENSMU	175	-28.5	175	-28.5	7423	
mmu-r	ENSMU	175	-28.5	175	-28.5	7423	
mmu-r	ENSMU	175	-28.5	175	-28.5	7423	
mmu-r	ENSMU	175	-28.2	175	-28.2	2686	
mmu-r	ENSMU	174	-35.2	174	-35.2	3822	
mmu-r	ENSMU	174	-33.8	174	-33.8	1212	
mmu-r	ENSMU	174	-29.6	174	-29.6	1911	
mmu-r	ENSMU	174	-27.5	174	-27.5	266	
mmu-r	ENSMU	174	-23.2	174	-23.2	8520	
mmu-r	ENSMU	173	-33.8	173	-33.8	1552	
mmu-r	ENSMU	173	-31.5	173	-31.5	7421	
mmu-r	Gm388	173	-27.8	173	-27.8	4705	
rco-mil	Gm412	173	-26	173	-26	2746	
rco-mil	Gm412	173	-26	173	-26	2746	
rco-mil	Gm412	173	-26	173	-26	2746	
rco-mil	Gm412	173	-26	173	-26	2746	
mmu-r	ENSMU	173	-21.8	173	-21.8	4633	
mmu-r	ENSMU	173	-21	173	-21	1431	
mmu-r	ENSMU	172	-36.6	172	-36.6	7406	
mmu-r	ENSMU	172	-30.7	172	-30.7	510	

mmu-r	ENSMU	172	-29.8	172	-29.8	3345
mmu-r	ENSMU	172	-29	172	-29	6035
mmu-r	ENSMU	172	-28.8	172	-28.8	1497
mmu-r	ENSMU	172	-28.1	172	-28.1	4119
mmu-r	Gm412	172	-27.8	172	-27.8	1945
mmu-r	ENSMU	172	-26.1	172	-26.1	7470
mmu-r	ENSMU	172	-26	172	-26	7468
mmu-r	ENSMU	172	-21.6	172	-21.6	1991
mmu-r	ENSMU	171	-33.9	171	-33.9	2823
mmu-r	ENSMU	171	-33.7	171	-33.7	10512
mmu-r	ENSMU	171	-28.2	171	-28.2	6190
mmu-r	Gm412	171	-26.4	171	-26.4	4122
mmu-r	Gm412	171	-25.2	171	-25.2	4167
mmu-r	Gm412	171	-22.6	171	-22.6	1922
mmu-r	ENSMU	170	-36	170	-36	1345
mmu-r	ENSMU	170	-35.1	170	-35.1	2288
mmu-r	ENSMU	170	-31.4	170	-31.4	156
mmu-r	ENSMU	170	-30.4	170	-30.4	1014
mmu-r	ENSMU	170	-29.1	170	-29.1	3745
mmu-r	ENSMU	170	-28.6	170	-28.6	12183
mmu-r	ENSMU	170	-28.6	170	-28.6	9091
mmu-r	Gm388	170	-27.8	170	-27.8	8561
mmu-r	ENSMU	170	-27.6	170	-27.6	7424
mmu-r	Gm388	170	-26.5	170	-26.5	7047
mmu-r	ENSMU	170	-25.3	170	-25.3	1228
mmu-r	ENSMU	170	-20.1	170	-20.1	1340
mmu-r	ENSMU	169	-33.1	169	-33.1	662
mmu-r	Gm412	169	-30.3	169	-30.3	2482
mmu-r	ENSMU	169	-30.3	169	-30.3	2685
mmu-r	ENSMU	169	-30	169	-30	9018
mmu-r	ENSMU	169	-29.2	169	-29.2	530
mmu-r	ENSMU	169	-25.1	169	-25.1	11970
mmu-r	ENSMU	169	-24.4	169	-24.4	4464
mmu-r	ENSMU	169	-24.4	169	-24.4	1649
mmu-r	ENSMU	169	-23.2	169	-23.2	6438
mmu-r	ENSMU	169	-22.5	169	-22.5	1496
mmu-r	ENSMU	169	-21.4	169	-21.4	5614
mmu-r	ENSMU	168	-37	168	-37	1554
mmu-r	ENSMU	168	-35.1	168	-35.1	750
mmu-r	ENSMU	168	-32.3	168	-32.3	13436
mmu-r	ENSMU	168	-28.8	168	-28.8	744
mmu-r	ENSMU	168	-28.7	168	-28.7	321
mmu-r	Gm388	168	-28.6	168	-28.6	5432
mmu-r	ENSMU	168	-27.6	168	-27.6	661
mmu-r	ENSMU	168	-27.4	168	-27.4	6831
mmu-r	ENSMU	168	-26.9	168	-26.9	3334
mmu-r	ENSMU	168	-26.6	168	-26.6	1692
mmu-r	ENSMU	168	-26.4	168	-26.4	647
mmu-r	Gm388	168	-26.3	168	-26.3	7969
mmu-r	ENSMU	168	-24.4	168	-24.4	5725
mmu-r	Gm388	168	-24.2	168	-24.2	4346
mmu-r	ENSMU	168	-24	168	-24	728
mmu-r	ENSMU	168	-23.4	168	-23.4	362
mmu-r	ENSMU	168	-22.5	168	-22.5	2135
mmu-r	ENSMU	168	-20.5	168	-20.5	4146
mmu-r	Gm412	168	-20.1	168	-20.1	4352
mmu-r	Gm388	168	-20.1	168	-20.1	3445
mmu-r	ENSMU	167	-41.7	167	-41.7	612
mmu-r	ENSMU	167	-35.9	167	-35.9	14166
mmu-r	ENSMU	167	-34.9	167	-34.9	7139
mmu-r	ENSMU	167	-33.7	167	-33.7	2840
mmu-r	Gm388	167	-32.4	167	-32.4	1273
mmu-r	ENSMU	167	-32.2	167	-32.2	5690
mmu-r	ENSMU	167	-31.9	167	-31.9	1559

mmu-r	ENSMU	167	-29	167	-29	1270
mmu-r	ENSMU	167	-28.7	167	-28.7	10647
mmu-r	ENSMU	167	-28.2	167	-28.2	13552
mmu-r	ENSMU	167	-28.1	167	-28.1	1160
mmu-r	ENSMU	167	-27.9	167	-27.9	8140
mmu-r	ENSMU	167	-27.5	167	-27.5	8990
mmu-r	ENSMU	167	-27.5	167	-27.5	284
mmu-r	Gm412	167	-27.5	167	-27.5	4477
mmu-r	ENSMU	167	-26.6	167	-26.6	1558
mmu-r	Gm388	167	-25.9	167	-25.9	2175
mmu-r	ENSMU	167	-25.7	167	-25.7	181
mmu-r	Gm388	167	-25.3	167	-25.3	3865
mmu-r	ENSMU	167	-25	167	-25	5082
mmu-r	ENSMU	167	-24.7	167	-24.7	8751
mmu-r	ENSMU	167	-24.3	167	-24.3	1463
mmu-r	ENSMU	167	-23.5	167	-23.5	243
mmu-r	ENSMU	167	-23.2	167	-23.2	5077
mmu-r	Gm412	167	-23.2	167	-23.2	1467
mmu-r	ENSMU	167	-23.2	167	-23.2	2211
mmu-r	ENSMU	167	-22.9	167	-22.9	6210
mmu-r	ENSMU	167	-22.7	167	-22.7	1217
mmu-r	Gm388	167	-22.5	167	-22.5	1255
mmu-r	ENSMU	167	-21.1	167	-21.1	976
mmu-r	ENSMU	167	-20.7	167	-20.7	679
mmu-r	Gm388	166	-31.5	166	-31.5	7015
mmu-r	Gm412	166	-31	166	-31	1780
mmu-r	ENSMU	166	-29.4	166	-29.4	3975
mmu-r	ENSMU	166	-29.3	166	-29.3	4503
mmu-r	ENSMU	166	-28.7	166	-28.7	10104
mmu-r	ENSMU	166	-28.6	166	-28.6	227
mmu-r	Gm412	166	-28.3	166	-28.3	153
mmu-r	ENSMU	166	-27.3	166	-27.3	1232
mmu-r	ENSMU	166	-27	166	-27	9633
mmu-r	ENSMU	166	-26.4	166	-26.4	8851
mmu-r	Gm412	166	-26.4	166	-26.4	1088
mmu-r	ENSMU	166	-25.9	166	-25.9	1863
mmu-r	ENSMU	166	-25.9	166	-25.9	12298
mmu-r	ENSMU	166	-24.7	166	-24.7	294
mmu-r	ENSMU	166	-23.8	166	-23.8	174
mmu-r	ENSMU	166	-23.5	166	-23.5	4602
mmu-r	ENSMU	166	-23.5	166	-23.5	8124
mmu-r	ENSMU	166	-23.3	166	-23.3	987
mmu-r	ENSMU	166	-23.1	166	-23.1	11134
mmu-r	ENSMU	166	-22.4	166	-22.4	2154
mmu-r	ENSMU	166	-22.3	166	-22.3	3256
rco-mil	Gm388	166	-21.9	166	-21.9	3705
mmu-r	ENSMU	166	-20.2	166	-20.2	1802
mmu-r	ENSMU	166	-20.1	166	-20.1	7528
mmu-r	ENSMU	165	-36	165	-36	3723
mmu-r	ENSMU	165	-34.1	165	-34.1	3990
mmu-r	Gm388	165	-33.2	165	-33.2	360
mmu-r	Gm412	165	-33.1	165	-33.1	4478
mmu-r	Gm388	165	-29.9	165	-29.9	7064
mmu-r	ENSMU	165	-29.1	165	-29.1	1006
mmu-r	ENSMU	165	-29	165	-29	2063
mmu-r	ENSMU	165	-29	165	-29	1090
mmu-r	Gm388	165	-28.9	165	-28.9	8630
mmu-r	ENSMU	165	-28.7	165	-28.7	7899
mmu-r	ENSMU	165	-27.7	165	-27.7	2432
mmu-r	ENSMU	165	-27.4	165	-27.4	219
mmu-r	ENSMU	165	-27.3	165	-27.3	6278
mmu-r	ENSMU	165	-27.2	165	-27.2	1368
mmu-r	ENSMU	165	-26.6	165	-26.6	8671
mmu-r	Gm412	165	-26.2	165	-26.2	2481

mmu-r	ENSMU	165	-25.7	165	-25.7	12382
mmu-r	ENSMU	165	-25.5	165	-25.5	3896
mmu-r	ENSMU	165	-25.2	165	-25.2	3878
mmu-r	Gm412	165	-24.9	165	-24.9	2050
mmu-r	Gm412	165	-24.7	165	-24.7	3099
mmu-r	Gm412	165	-24.4	165	-24.4	3188
mmu-r	ENSMU	165	-24	165	-24	300
mmu-r	ENSMU	165	-23.7	165	-23.7	2536
mmu-r	ENSMU	165	-23.5	165	-23.5	7942
mmu-r	ENSMU	165	-23	165	-23	4084
mmu-r	ENSMU	165	-22	165	-22	4099
mmu-r	Gm388	165	-21.9	165	-21.9	1138
mmu-r	ENSMU	165	-21.4	165	-21.4	905
mmu-r	Gm412	165	-21	165	-21	576
mmu-r	ENSMU	165	-20	165	-20	3115
mmu-r	ENSMU	164	-31.1	164	-31.1	9827
mmu-r	ENSMU	164	-30.8	164	-30.8	2268
mmu-r	ENSMU	164	-27.9	164	-27.9	2177
mmu-r	ENSMU	164	-27.3	164	-27.3	6036
mmu-r	Gm412	164	-27.2	164	-27.2	1097
mmu-r	ENSMU	164	-26.9	164	-26.9	2893
mmu-r	ENSMU	164	-26.6	164	-26.6	3462
mmu-r	ENSMU	164	-26.4	164	-26.4	543
mmu-r	ENSMU	164	-26	164	-26	4650
mmu-r	ENSMU	164	-25.8	164	-25.8	200
mmu-r	ENSMU	164	-25.3	164	-25.3	1013
mmu-r	ENSMU	164	-24.8	164	-24.8	6953
mmu-r	ENSMU	164	-24.7	164	-24.7	1092
mmu-r	ENSMU	164	-24.6	164	-24.6	14022
mmu-r	ENSMU	164	-24.3	164	-24.3	12729
mmu-r	ENSMU	164	-23.6	164	-23.6	7369
mmu-r	Gm412	164	-22.9	164	-22.9	2475
mmu-r	Gm412	164	-22.5	164	-22.5	2649
mmu-r	ENSMU	164	-22.4	164	-22.4	5525
mmu-r	ENSMU	164	-22.3	164	-22.3	4442
mmu-r	Gm388	164	-22.1	164	-22.1	948
mmu-r	ENSMU	164	-22	164	-22	698
rco-mi	Gm388	164	-21.2	164	-21.2	2415
rco-mi	Gm388	164	-21.2	164	-21.2	2415
rco-mi	Gm388	164	-21.2	164	-21.2	2415
rco-mi	Gm388	164	-21.2	164	-21.2	2415
mmu-r	ENSMU	164	-20.4	164	-20.4	1216
mmu-r	ENSMU	164	-20.2	164	-20.2	12404
mmu-r	ENSMU	163	-29.1	163	-29.1	3882
mmu-r	ENSMU	163	-28.9	163	-28.9	56
mmu-r	Gm388	163	-28.4	163	-28.4	85
mmu-r	Gm388	163	-27.7	163	-27.7	4516
mmu-r	ENSMU	163	-27.7	163	-27.7	13933
mmu-r	ENSMU	163	-27.6	163	-27.6	2696
mmu-r	ENSMU	163	-26.5	163	-26.5	4348
mmu-r	ENSMU	163	-26.4	163	-26.4	3810
mmu-r	Gm388	163	-26.3	163	-26.3	8437
mmu-r	ENSMU	163	-25.3	163	-25.3	7358
mmu-r	ENSMU	163	-24.7	163	-24.7	1362
mmu-r	ENSMU	163	-24.1	163	-24.1	6980
mmu-r	ENSMU	163	-23.9	163	-23.9	58
mmu-r	Gm388	163	-23.8	163	-23.8	1661
mmu-r	ENSMU	163	-23.7	163	-23.7	2489
mmu-r	ENSMU	163	-23.6	163	-23.6	1085
mmu-r	ENSMU	163	-23.3	163	-23.3	374
mmu-r	ENSMU	163	-22.9	163	-22.9	991
rco-mi	Gm388	163	-22.6	163	-22.6	434
mmu-r	ENSMU	163	-22.6	163	-22.6	1244
mmu-r	ENSMU	163	-22.5	163	-22.5	7400

mmu-r	ENSMU	163	-22.3	163	-22.3	3976
mmu-r	Gm412	163	-22	163	-22	2343
mmu-r	Gm412	163	-21.9	163	-21.9	464
mmu-r	ENSMU	163	-21.7	163	-21.7	3042
mmu-l	ENSMU	163	-21.3	163	-21.3	1477
mmu-r	ENSMU	163	-21.1	163	-21.1	6004
mmu-r	Gm388	163	-21	163	-21	4497
mmu-r	ENSMU	163	-20.8	163	-20.8	2949
mmu-r	ENSMU	163	-20.8	163	-20.8	9590
mmu-r	ENSMU	163	-20.7	163	-20.7	9821
mmu-r	ENSMU	163	-20.6	163	-20.6	8285
mmu-r	Gm412	163	-20.5	163	-20.5	3117
mmu-r	ENSMU	163	-20.3	163	-20.3	1294
mmu-r	ENSMU	162	-36	162	-36	8654
mmu-r	ENSMU	162	-30.3	162	-30.3	7556
mmu-r	ENSMU	162	-29.1	162	-29.1	296
mmu-r	ENSMU	162	-28.7	162	-28.7	9645
mmu-r	ENSMU	162	-28.5	162	-28.5	3986
mmu-r	ENSMU	162	-28.2	162	-28.2	2893
mmu-r	Gm388	162	-28.2	162	-28.2	831
mmu-r	ENSMU	162	-28.2	162	-28.2	5166
mmu-r	Gm388	162	-27.5	162	-27.5	5420
mmu-r	ENSMU	162	-26.4	162	-26.4	690
mmu-r	ENSMU	162	-26	162	-26	3815
mmu-r	ENSMU	162	-25.8	162	-25.8	14171
mmu-r	ENSMU	162	-25.7	162	-25.7	1348
mmu-r	ENSMU	162	-24.8	162	-24.8	6697
mmu-r	ENSMU	162	-24.8	162	-24.8	6957
mmu-r	ENSMU	162	-24.7	162	-24.7	360
mmu-r	Gm388	162	-24.4	162	-24.4	2409
mmu-r	ENSMU	162	-24.4	162	-24.4	7952
mmu-r	ENSMU	162	-23.9	162	-23.9	2239
mmu-r	ENSMU	162	-23.7	162	-23.7	760
mmu-l	Gm388	162	-23.7	162	-23.7	4318
mmu-r	ENSMU	162	-23.7	162	-23.7	11238
mmu-r	ENSMU	162	-23.3	162	-23.3	6438
mmu-r	Gm388	162	-23.2	162	-23.2	8382
mmu-r	Gm388	162	-23	162	-23	5467
mmu-r	ENSMU	162	-22.9	162	-22.9	6793
mmu-r	ENSMU	162	-22.9	162	-22.9	7426
mmu-r	Gm412	162	-22.7	162	-22.7	3726
mmu-r	ENSMU	162	-22.7	162	-22.7	3460
mmu-r	ENSMU	162	-22.6	162	-22.6	11235
rco-mil	ENSMU	162	-22.6	162	-22.6	6088
mmu-r	ENSMU	162	-22.5	162	-22.5	269
rco-mil	ENSMU	162	-22.3	162	-22.3	356
mmu-r	ENSMU	162	-22.2	162	-22.2	4648
mmu-r	ENSMU	162	-21.9	162	-21.9	3208
mmu-r	Gm388	162	-21.9	162	-21.9	1524
mmu-r	ENSMU	162	-21.9	162	-21.9	11685
mmu-r	ENSMU	162	-21.6	162	-21.6	9716
mmu-r	Gm412	162	-21.6	162	-21.6	2702
mmu-r	ENSMU	162	-21.6	162	-21.6	4325
mmu-r	ENSMU	162	-21.5	162	-21.5	800
rco-mil	Gm388	162	-21.3	162	-21.3	3702
rco-mil	Gm388	162	-21.3	162	-21.3	3702
rco-mil	Gm388	162	-21.3	162	-21.3	3702
rco-mil	Gm388	162	-21.3	162	-21.3	3702
mmu-r	ENSMU	162	-21.2	162	-21.2	3105
mmu-r	Gm388	162	-21.1	162	-21.1	509
mmu-r	ENSMU	162	-20.9	162	-20.9	90
mmu-r	ENSMU	162	-20.7	162	-20.7	1210
mmu-r	ENSMU	162	-20.5	162	-20.5	2679
mmu-r	ENSMU	162	-20.5	162	-20.5	5572

mmu-r	Gm412	162	-20.5	162	-20.5	396
rco-mil	ENSMU	162	-20.4	162	-20.4	6089
rco-mil	ENSMU	162	-20.4	162	-20.4	6089
mmu-r	Gm388	161	-32.5	161	-32.5	6626
mmu-r	ENSMU	161	-31.6	161	-31.6	4366
mmu-r	Gm388	161	-29.3	161	-29.3	4183
mmu-r	ENSMU	161	-29	161	-29	298
mmu-r	ENSMU	161	-28.7	161	-28.7	9634
mmu-r	ENSMU	161	-28.1	161	-28.1	2059
mmu-r	ENSMU	161	-27.7	161	-27.7	3842
mmu-r	Gm388	161	-27.6	161	-27.6	783
mmu-r	Gm412	161	-27.1	161	-27.1	3076
mmu-r	Gm388	161	-26.3	161	-26.3	6092
mmu-r	ENSMU	161	-25.1	161	-25.1	7554
mmu-r	ENSMU	161	-25	161	-25	1038
mmu-r	ENSMU	161	-24.9	161	-24.9	6976
mmu-r	ENSMU	161	-24.7	161	-24.7	3873
mmu-r	ENSMU	161	-24.6	161	-24.6	1232
mmu-r	ENSMU	161	-24.4	161	-24.4	7927
hiv1-m	ENSMU	161	-24.4	161	-24.4	7712
mmu-r	ENSMU	161	-24	161	-24	3369
mmu-r	ENSMU	161	-24	161	-24	11874
mmu-r	ENSMU	161	-23.9	161	-23.9	6959
mmu-r	Gm388	161	-23.5	161	-23.5	242
mmu-r	ENSMU	161	-23.5	161	-23.5	7927
mmu-r	ENSMU	161	-23.3	161	-23.3	91
mmu-r	Gm388	161	-23.3	161	-23.3	8237
mmu-r	ENSMU	161	-23.3	161	-23.3	2181
mmu-r	ENSMU	161	-23.2	161	-23.2	3978
mmu-r	ENSMU	161	-23.2	161	-23.2	6834
mmu-r	Gm388	161	-23.1	161	-23.1	4183
mmu-r	Gm412	161	-23	161	-23	1154
mmu-r	ENSMU	161	-23	161	-23	5743
mmu-r	ENSMU	161	-22.3	161	-22.3	1633
mmu-r	Gm412	161	-22.3	161	-22.3	577
mmu-r	ENSMU	161	-22	161	-22	7554
mmu-r	Gm388	161	-22	161	-22	2718
mmu-r	ENSMU	161	-22	161	-22	458
mmu-r	Gm412	161	-21.9	161	-21.9	463
mmu-r	ENSMU	161	-21.3	161	-21.3	3315
mmu-r	ENSMU	161	-21.1	161	-21.1	5613
mmu-r	ENSMU	161	-20.9	161	-20.9	3368
mmu-r	ENSMU	161	-20.8	161	-20.8	7429
mmu-r	ENSMU	161	-20.5	161	-20.5	3102
mmu-r	Gm412	161	-20.4	161	-20.4	2561
mmu-r	ENSMU	161	-20.1	161	-20.1	14300
mmu-r	ENSMU	160	-30.2	160	-30.2	6852
mmu-r	ENSMU	160	-29.4	160	-29.4	5743
mmu-r	ENSMU	160	-28.7	160	-28.7	3925
mmu-r	ENSMU	160	-28.4	160	-28.4	3013
mmu-r	Gm388	160	-28.2	160	-28.2	5397
mmu-r	ENSMU	160	-27.8	160	-27.8	12373
mmu-r	ENSMU	160	-27.5	160	-27.5	1870
mmu-r	ENSMU	160	-27.4	160	-27.4	664
mmu-r	ENSMU	160	-27.2	160	-27.2	1705
mmu-r	ENSMU	160	-27.2	160	-27.2	3133
mmu-r	ENSMU	160	-26.9	160	-26.9	2104
mmu-r	Gm388	160	-26.9	160	-26.9	2675
mmu-r	ENSMU	160	-26.1	160	-26.1	4664
mmu-r	Gm412	160	-26	160	-26	1153
mmu-r	ENSMU	160	-25.8	160	-25.8	88
mmu-r	ENSMU	160	-25.7	160	-25.7	6417
mmu-r	Gm388	160	-25.2	160	-25.2	7871
mmu-r	Gm412	160	-25.1	160	-25.1	3772

mmu-r	ENSMU	160	-25	160	-25	3888
mmu-r	ENSMU	160	-24.7	160	-24.7	8131
mmu-r	ENSMU	160	-24.6	160	-24.6	1289
mmu-r	ENSMU	160	-24.5	160	-24.5	864
mmu-r	Gm412	160	-23.7	160	-23.7	3560
mmu-r	Gm388	160	-23.5	160	-23.5	7486
mmu-r	ENSMU	160	-23.3	160	-23.3	2893
mmu-r	ENSMU	160	-23.3	160	-23.3	1385
mmu-r	ENSMU	160	-23.3	160	-23.3	4503
mmu-r	Gm388	160	-23.1	160	-23.1	6271
mmu-r	Gm388	160	-23	160	-23	2029
mmu-r	ENSMU	160	-23	160	-23	3669
mmu-r	ENSMU	160	-23	160	-23	2440
mmu-r	ENSMU	160	-22.6	160	-22.6	3935
mmu-r	Gm388	160	-22.5	160	-22.5	8237
mmu-r	ENSMU	160	-22.5	160	-22.5	7149
mmu-r	ENSMU	160	-22.4	160	-22.4	2310
mmu-r	ENSMU	160	-22.1	160	-22.1	1511
mmu-r	ENSMU	160	-22.1	160	-22.1	9831
mmu-r	ENSMU	160	-22	160	-22	1835
mmu-r	Gm388	160	-21.9	160	-21.9	1561
mmu-r	ENSMU	160	-21.8	160	-21.8	3946
mmu-r	ENSMU	160	-21.5	160	-21.5	3988
mmu-r	ENSMU	160	-21.4	160	-21.4	3381
mmu-r	ENSMU	160	-21.3	160	-21.3	1052
mmu-r	ENSMU	160	-21.1	160	-21.1	249
mmu-r	Gm412	160	-20.7	160	-20.7	2696
mmu-r	ENSMU	160	-20.5	160	-20.5	1597
mmu-r	ENSMU	160	-20.2	160	-20.2	5725
mmu-r	ENSMU	159	-30.8	159	-30.8	1949
mmu-r	ENSMU	159	-30.3	159	-30.3	55
mmu-r	ENSMU	159	-30.2	159	-30.2	2823
mmu-r	ENSMU	159	-28.9	159	-28.9	2161
mmu-r	ENSMU	159	-28.4	159	-28.4	3874
mmu-r	ENSMU	159	-28.2	159	-28.2	2362
mmu-r	ENSMU	159	-27.7	159	-27.7	7201
mmu-r	Gm388	159	-27.4	159	-27.4	783
mmu-r	Gm412	159	-27.3	159	-27.3	21
mmu-r	ENSMU	159	-27.2	159	-27.2	3860
mmu-r	Gm388	159	-27	159	-27	6467
mmu-r	ENSMU	159	-26.4	159	-26.4	8145
mmu-r	ENSMU	159	-26.4	159	-26.4	4031
mmu-r	ENSMU	159	-26.2	159	-26.2	2154
mmu-r	ENSMU	159	-26	159	-26	2811
mmu-r	ENSMU	159	-26	159	-26	708
mmu-r	ENSMU	159	-26	159	-26	8790
mmu-r	ENSMU	159	-25.8	159	-25.8	3889
mmu-r	ENSMU	159	-25.5	159	-25.5	1237
mmu-r	ENSMU	159	-25.5	159	-25.5	2143
mmu-r	Gm388	159	-25.3	159	-25.3	7065
mmu-r	ENSMU	159	-25.2	159	-25.2	216
mmu-r	ENSMU	159	-25.2	159	-25.2	3745
mmu-r	ENSMU	159	-25.1	159	-25.1	3993
mmu-r	ENSMU	159	-24.9	159	-24.9	11527
mmu-r	ENSMU	159	-24.9	159	-24.9	3994
mmu-r	ENSMU	159	-24.8	159	-24.8	872
mmu-r	ENSMU	159	-24.6	159	-24.6	4357
mmu-r	ENSMU	159	-24.2	159	-24.2	2102
mmu-r	ENSMU	159	-24.1	159	-24.1	9033
mmu-r	ENSMU	159	-24	159	-24	3743
mmu-r	ENSMU	159	-23.8	159	-23.8	4399
mmu-r	ENSMU	159	-23.8	159	-23.8	5403
mmu-r	ENSMU	159	-23.8	159	-23.8	667
mmu-r	ENSMU	159	-23.7	159	-23.7	5854

mmu-r	ENSMU	159	-23.7	159	-23.7	1205
mmu-r	ENSMU	159	-23.5	159	-23.5	2765
mmu-r	ENSMU	159	-23.5	159	-23.5	4346
mmu-r	Gm412	159	-23.3	159	-23.3	1421
mmu-r	ENSMU	159	-23.2	159	-23.2	1911
mmu-r	ENSMU	159	-22.9	159	-22.9	1907
mmu-r	ENSMU	159	-22.1	159	-22.1	3758
mmu-r	ENSMU	159	-22	159	-22	918
mmu-r	ENSMU	159	-21.9	159	-21.9	7506
mmu-r	ENSMU	159	-21.9	159	-21.9	3530
mmu-r	ENSMU	159	-21.6	159	-21.6	2428
mmu-r	ENSMU	159	-21.5	159	-21.5	1575
mmu-r	Gm388	159	-21	159	-21	296
mmu-r	Gm412	159	-20.8	159	-20.8	863
mmu-r	ENSMU	159	-20.2	159	-20.2	2809
mmu-r	ENSMU	159	-20.2	159	-20.2	2207
mmu-r	ENSMU	159	-20.1	159	-20.1	346
mmu-r	ENSMU	158	-32.6	158	-32.6	5198
mmu-r	ENSMU	158	-31.5	158	-31.5	3720
mmu-r	ENSMU	158	-29.6	158	-29.6	7007
mmu-r	ENSMU	158	-29.4	158	-29.4	1309
mmu-r	ENSMU	158	-29	158	-29	8143
mmu-r	ENSMU	158	-28.7	158	-28.7	376
mmu-r	Gm388	158	-28.6	158	-28.6	2908
mmu-r	ENSMU	158	-28.4	158	-28.4	5114
mmu-r	ENSMU	158	-28.2	158	-28.2	1553
mmu-r	ENSMU	158	-27.2	158	-27.2	3941
mmu-r	ENSMU	158	-26.9	158	-26.9	873
mmu-r	ENSMU	158	-26.9	158	-26.9	8486
mmu-r	ENSMU	158	-26.7	158	-26.7	68
mmu-r	ENSMU	158	-26	158	-26	1256
mmu-r	Gm412	158	-26	158	-26	30
mmu-r	ENSMU	158	-25.9	158	-25.9	14046
mmu-r	Gm388	158	-25.9	158	-25.9	7786
mmu-r	Gm388	158	-25.9	158	-25.9	7486
mmu-r	ENSMU	158	-25.7	158	-25.7	665
mmu-r	ENSMU	158	-25.5	158	-25.5	4324
mmu-r	ENSMU	158	-25.3	158	-25.3	8787
mmu-r	Gm388	158	-25.3	158	-25.3	358
mmu-r	ENSMU	158	-25.2	158	-25.2	6361
mmu-r	Gm412	158	-25	158	-25	1780
mmu-r	ENSMU	158	-24.2	158	-24.2	1309
mmu-r	Gm388	158	-24.1	158	-24.1	7474
mmu-r	Gm388	158	-24.1	158	-24.1	4365
mmu-r	Gm412	158	-24.1	158	-24.1	642
mmu-r	ENSMU	158	-23.9	158	-23.9	2255
mmu-r	ENSMU	158	-23.8	158	-23.8	7366
mmu-r	ENSMU	158	-23.8	158	-23.8	7162
mmu-r	ENSMU	158	-23.7	158	-23.7	2946
mmu-r	Gm388	158	-23.6	158	-23.6	346
mmu-r	ENSMU	158	-23.5	158	-23.5	12574
mmu-l	Gm388	158	-22.9	158	-22.9	4318
mmu-r	Gm388	158	-22.6	158	-22.6	8898
mmu-r	ENSMU	158	-22.4	158	-22.4	10380
mmu-r	ENSMU	158	-22.4	158	-22.4	2255
mmu-r	ENSMU	158	-22.3	158	-22.3	5100
mmu-r	ENSMU	158	-22.2	158	-22.2	9211
mmu-r	ENSMU	158	-22.1	158	-22.1	5939
mmu-r	ENSMU	158	-22.1	158	-22.1	2749
mmu-r	Gm412	158	-22	158	-22	24
mmu-r	ENSMU	158	-22	158	-22	3766
mmu-r	ENSMU	158	-21.6	158	-21.6	266
mmu-r	ENSMU	158	-21.5	158	-21.5	6976
mmu-r	ENSMU	158	-21.5	158	-21.5	12740

mmu-r	ENSMU	158	-21	158	-21	3911
mmu-l	Gm388	158	-20.7	158	-20.7	4318
mmu-r	ENSMU	158	-20.6	158	-20.6	8988
rco-mi	ENSMU	158	-20.6	158	-20.6	6578
rco-mi	ENSMU	158	-20.6	158	-20.6	6578
mmu-r	ENSMU	158	-20.6	158	-20.6	6697
mmu-r	ENSMU	158	-20.5	158	-20.5	688
mmu-r	ENSMU	158	-20.5	158	-20.5	5559
mmu-r	ENSMU	158	-20.4	158	-20.4	4178
rco-mi	Gm388	158	-20.3	158	-20.3	4467
mmu-r	ENSMU	158	-20.2	158	-20.2	2387
mmu-r	ENSMU	158	-20.1	158	-20.1	3695
mmu-l	Gm388	158	-20.1	158	-20.1	4319
mmu-r	ENSMU	158	-20.1	158	-20.1	3438
mmu-r	ENSMU	158	-20.1	158	-20.1	1482
mmu-r	Gm388	158	-20.1	158	-20.1	2411
mmu-r	ENSMU	157	-32.6	157	-32.6	2821
mmu-r	ENSMU	157	-30	157	-30	2422
mmu-r	ENSMU	157	-30	157	-30	2832
mmu-r	ENSMU	157	-29.8	157	-29.8	10651
mmu-r	ENSMU	157	-28.2	157	-28.2	5849
mmu-r	ENSMU	157	-27.7	157	-27.7	102
mmu-r	Gm412	157	-27.3	157	-27.3	966
mmu-r	ENSMU	157	-26.6	157	-26.6	790
mmu-r	ENSMU	157	-26.4	157	-26.4	611
mmu-r	ENSMU	157	-26.2	157	-26.2	1556
mmu-r	ENSMU	157	-25.9	157	-25.9	3452
mmu-r	Gm388	157	-25.6	157	-25.6	7874
mmu-r	ENSMU	157	-25.3	157	-25.3	8783
mmu-r	ENSMU	157	-25.3	157	-25.3	11016
mmu-r	ENSMU	157	-25.1	157	-25.1	84
mmu-r	Gm388	157	-24.5	157	-24.5	8530
mmu-r	Gm388	157	-23.8	157	-23.8	5431
mmu-r	ENSMU	157	-23.8	157	-23.8	2821
mmu-r	ENSMU	157	-23.6	157	-23.6	578
mmu-r	ENSMU	157	-23.5	157	-23.5	3354
mmu-r	ENSMU	157	-23.4	157	-23.4	299
mmu-r	Gm412	157	-23	157	-23	2502
mmu-r	Gm412	157	-22.8	157	-22.8	951
mmu-r	ENSMU	157	-22.7	157	-22.7	14332
mmu-r	ENSMU	157	-22.4	157	-22.4	1717
mmu-r	ENSMU	157	-22.3	157	-22.3	7927
mmu-r	ENSMU	157	-21.9	157	-21.9	1599
mmu-r	Gm388	157	-21.8	157	-21.8	1382
mmu-r	ENSMU	157	-21.7	157	-21.7	1545
mmu-r	ENSMU	157	-21.6	157	-21.6	8028
mmu-r	ENSMU	157	-21.5	157	-21.5	8677
mmu-r	ENSMU	157	-21.3	157	-21.3	4343
mmu-r	Gm388	157	-21	157	-21	8650
mmu-r	ENSMU	157	-20.8	157	-20.8	7063
mmu-r	Gm412	157	-20.8	157	-20.8	728
mmu-r	ENSMU	157	-20.7	157	-20.7	544
mmu-r	ENSMU	157	-20.6	157	-20.6	724
mmu-r	ENSMU	157	-20.5	157	-20.5	8854
mmu-r	ENSMU	157	-20.4	157	-20.4	4439
mmu-r	Gm412	157	-20.4	157	-20.4	1092
mmu-r	Gm412	157	-20.2	157	-20.2	50
mmu-r	ENSMU	157	-20.1	157	-20.1	2351
mmu-r	ENSMU	157	-20.1	157	-20.1	871
mmu-r	ENSMU	156	-29.8	156	-29.8	3338
mmu-r	ENSMU	156	-29.5	156	-29.5	9029
mmu-r	ENSMU	156	-29.1	156	-29.1	10653
mmu-r	Gm388	156	-28.4	156	-28.4	1124
mmu-r	Gm388	156	-28.2	156	-28.2	1184

mmu-r	ENSML	156	-27.6	156	-27.6	3882
mmu-r	ENSML	156	-26.9	156	-26.9	1536
mmu-r	ENSML	156	-26.6	156	-26.6	1037
mmu-r	Gm388	156	-26.2	156	-26.2	3370
mmu-r	ENSML	156	-26	156	-26	1236
mmu-r	ENSML	156	-25.5	156	-25.5	4861
mmu-r	ENSML	156	-25.3	156	-25.3	6314
mmu-r	ENSML	156	-25.3	156	-25.3	4042
mmu-r	ENSML	156	-25	156	-25	920
mmu-r	ENSML	156	-24.9	156	-24.9	89
mmu-r	ENSML	156	-24.9	156	-24.9	123
mmu-r	ENSML	156	-24.9	156	-24.9	709
mmu-r	ENSML	156	-24.7	156	-24.7	2893
mmu-r	ENSML	156	-24.3	156	-24.3	8249
mmu-r	ENSML	156	-24.3	156	-24.3	11235
mmu-r	ENSML	156	-24.3	156	-24.3	6718
mmu-r	ENSML	156	-24.2	156	-24.2	1245
mmu-r	ENSML	156	-24.2	156	-24.2	11235
mmu-r	ENSML	156	-24.1	156	-24.1	7348
mmu-r	ENSML	156	-24.1	156	-24.1	2379
mmu-r	ENSML	156	-24.1	156	-24.1	2307
mmu-r	Gm388	156	-23.9	156	-23.9	2379
mmu-r	ENSML	156	-23.8	156	-23.8	8810
mmu-r	Gm412	156	-23.6	156	-23.6	2483
hiv1-m	Gm412	156	-23.5	156	-23.5	355
mmu-r	Gm388	156	-22.7	156	-22.7	6790
mmu-r	ENSML	156	-22.6	156	-22.6	1360
mmu-r	ENSML	156	-22.4	156	-22.4	4016
mmu-r	ENSML	156	-22.4	156	-22.4	2101
mmu-r	ENSML	156	-22.4	156	-22.4	10103
mmu-r	ENSML	156	-22.3	156	-22.3	2532
mmu-r	ENSML	156	-22.3	156	-22.3	7552
mmu-r	ENSML	156	-22.3	156	-22.3	3888
mmu-r	Gm412	156	-22.1	156	-22.1	2875
mmu-r	ENSML	156	-22.1	156	-22.1	3309
mmu-r	ENSML	156	-22	156	-22	1577
mmu-r	ENSML	156	-21.9	156	-21.9	6421
mmu-r	ENSML	156	-21.7	156	-21.7	1244
mmu-r	Gm412	156	-21.6	156	-21.6	3313
mmu-r	ENSML	156	-21.5	156	-21.5	4270
mmu-r	ENSML	156	-21.5	156	-21.5	1245
mmu-r	ENSML	156	-21.4	156	-21.4	12659
mmu-r	Gm388	156	-21.3	156	-21.3	2141
mmu-r	Gm388	156	-21.3	156	-21.3	718
mmu-r	ENSML	156	-21.2	156	-21.2	2346
mmu-r	ENSML	156	-21.1	156	-21.1	3937
mmu-r	Gm388	156	-21	156	-21	6572
mmu-r	ENSML	156	-20.9	156	-20.9	4231
mmu-r	Gm412	156	-20.6	156	-20.6	3563
mmu-r	ENSML	156	-20.5	156	-20.5	12780
mmu-r	Gm388	156	-20.5	156	-20.5	1669
mmu-r	ENSML	156	-20.4	156	-20.4	2976
mmu-r	ENSML	156	-20.1	156	-20.1	7795
mmu-r	ENSML	156	-20	156	-20	3213
mmu-r	ENSML	155	-35.8	155	-35.8	7529
mmu-r	ENSML	155	-34.5	155	-34.5	181
mmu-r	Gm388	155	-29.2	155	-29.2	8425
mmu-r	Gm412	155	-28.9	155	-28.9	2489
mmu-r	Gm388	155	-28.1	155	-28.1	187
mmu-r	Gm388	155	-27.8	155	-27.8	8245
mmu-r	ENSML	155	-27.8	155	-27.8	2499
mmu-r	ENSML	155	-27.3	155	-27.3	841
mmu-r	Gm412	155	-27.2	155	-27.2	4073
mmu-r	ENSML	155	-27.1	155	-27.1	1054

mmu-r	ENSMU	155	-25.3	155	-25.3	1313
mmu-r	ENSMU	155	-25.3	155	-25.3	4652
mmu-r	Gm412	155	-25.1	155	-25.1	2570
mmu-r	ENSMU	155	-24.8	155	-24.8	6237
mmu-r	ENSMU	155	-24.7	155	-24.7	1207
mmu-r	ENSMU	155	-24.7	155	-24.7	4325
mmu-r	Gm388	155	-24.4	155	-24.4	7069
mmu-r	ENSMU	155	-24.2	155	-24.2	8783
mmu-r	ENSMU	155	-24.2	155	-24.2	6496
mmu-r	ENSMU	155	-24.2	155	-24.2	5563
mmu-r	Gm388	155	-23.5	155	-23.5	3539
mmu-r	ENSMU	155	-23.5	155	-23.5	377
mmu-r	ENSMU	155	-23.2	155	-23.2	7365
mmu-r	ENSMU	155	-23.1	155	-23.1	3258
mmu-r	ENSMU	155	-23.1	155	-23.1	1384
mmu-r	ENSMU	155	-23.1	155	-23.1	6713
mmu-r	ENSMU	155	-23.1	155	-23.1	5869
mmu-r	Gm412	155	-22.7	155	-22.7	3767
mmu-r	ENSMU	155	-22.7	155	-22.7	5205
mmu-r	Gm388	155	-22.6	155	-22.6	4751
mmu-r	ENSMU	155	-22.5	155	-22.5	3677
mmu-r	ENSMU	155	-22.3	155	-22.3	6306
mmu-r	Gm388	155	-22.2	155	-22.2	371
mmu-r	ENSMU	155	-22.1	155	-22.1	7012
mmu-r	Gm388	155	-21.9	155	-21.9	8017
mmu-r	ENSMU	155	-21.9	155	-21.9	8745
mmu-r	ENSMU	155	-21.8	155	-21.8	4723
mmu-r	ENSMU	155	-21.8	155	-21.8	2236
mmu-r	ENSMU	155	-21.6	155	-21.6	4286
mmu-r	ENSMU	155	-21.6	155	-21.6	3878
mmu-r	ENSMU	155	-21.5	155	-21.5	2022
mmu-r	ENSMU	155	-21.4	155	-21.4	5626
mmu-r	Gm412	155	-21.3	155	-21.3	4125
mmu-r	ENSMU	155	-21.3	155	-21.3	4099
mmu-r	ENSMU	155	-21.2	155	-21.2	3290
mmu-r	Gm412	155	-21.2	155	-21.2	2042
mmu-r	ENSMU	155	-21.1	155	-21.1	6035
mmu-r	ENSMU	155	-21.1	155	-21.1	2397
mmu-r	ENSMU	155	-20.9	155	-20.9	10970
mmu-r	ENSMU	155	-20.8	155	-20.8	2714
mmu-r	Gm412	155	-20.7	155	-20.7	1870
mmu-r	Gm412	155	-20.7	155	-20.7	1328
mmu-r	Gm388	155	-20.6	155	-20.6	8436
mmu-r	ENSMU	155	-20.6	155	-20.6	4908
mmu-r	ENSMU	155	-20.4	155	-20.4	3723
mmu-r	Gm388	155	-20.4	155	-20.4	3576
mmu-r	ENSMU	155	-20.3	155	-20.3	6979
mmu-r	ENSMU	155	-20.3	155	-20.3	4199
mmu-r	Gm412	155	-20.3	155	-20.3	1090
mmu-r	ENSMU	155	-20.3	155	-20.3	9741
rco-mil	ENSMU	155	-20.3	155	-20.3	2540
rco-mil	ENSMU	155	-20.3	155	-20.3	2540
rco-mil	ENSMU	155	-20.3	155	-20.3	2540
rco-mil	ENSMU	155	-20.3	155	-20.3	2540
rco-mil	ENSMU	155	-20.3	155	-20.3	2540
mmu-r	ENSMU	155	-20.2	155	-20.2	5795
mmu-r	ENSMU	155	-20.1	155	-20.1	6210
mmu-r	ENSMU	155	-20.1	155	-20.1	6594
mmu-r	Gm412	155	-20.1	155	-20.1	3010
mmu-r	Gm388	155	-20	155	-20	3471
mmu-r	ENSMU	155	-20	155	-20	3718
mmu-r	Gm388	154	-30.3	154	-30.3	8246
mmu-r	Gm388	154	-30.2	154	-30.2	4538
mmu-r	Gm388	154	-28.6	154	-28.6	7404

mmu-r	ENSMU	154	-28.2	154	-28.2	230
mmu-r	Gm388	154	-28.1	154	-28.1	8799
mmu-r	ENSMU	154	-27.8	154	-27.8	8898
mmu-r	Gm388	154	-27.1	154	-27.1	795
mmu-r	Gm388	154	-26.5	154	-26.5	1639
mmu-r	Gm412	154	-26.3	154	-26.3	2572
mmu-r	ENSMU	154	-26.2	154	-26.2	1238
mmu-r	ENSMU	154	-25.8	154	-25.8	2761
mmu-r	ENSMU	154	-25.7	154	-25.7	2252
mmu-r	ENSMU	154	-25.2	154	-25.2	11085
mmu-r	ENSMU	154	-24.9	154	-24.9	11556
mmu-r	ENSMU	154	-24.6	154	-24.6	3717
mmu-r	ENSMU	154	-24.4	154	-24.4	1516
mmu-r	Gm412	154	-24.4	154	-24.4	2499
mmu-r	ENSMU	154	-24.2	154	-24.2	10647
mmu-r	ENSMU	154	-23.7	154	-23.7	3725
mmu-r	Gm412	154	-23.7	154	-23.7	3587
mmu-r	ENSMU	154	-23.7	154	-23.7	615
mmu-r	ENSMU	154	-23.6	154	-23.6	4345
mmu-r	Gm388	154	-23.6	154	-23.6	6559
mmu-r	ENSMU	154	-23.5	154	-23.5	8683
mmu-r	Gm388	154	-23.1	154	-23.1	7065
mmu-r	Gm412	154	-22.9	154	-22.9	2875
mmu-r	ENSMU	154	-22.9	154	-22.9	1360
mmu-r	ENSMU	154	-22.8	154	-22.8	6544
mmu-l	Gm388	154	-22.7	154	-22.7	4319
mmu-r	ENSMU	154	-22.5	154	-22.5	1579
mmu-r	ENSMU	154	-22.5	154	-22.5	1508
mmu-r	Gm412	154	-22.4	154	-22.4	1095
mmu-r	ENSMU	154	-22.4	154	-22.4	150
mmu-r	ENSMU	154	-22.3	154	-22.3	2143
mmu-r	ENSMU	154	-22.3	154	-22.3	2525
mmu-r	Gm412	154	-22.2	154	-22.2	642
mmu-r	ENSMU	154	-22.2	154	-22.2	1573
mmu-r	ENSMU	154	-22.2	154	-22.2	9019
mmu-r	ENSMU	154	-22	154	-22	8021
mmu-r	ENSMU	154	-21.8	154	-21.8	1160
mmu-r	Gm412	154	-21.7	154	-21.7	4475
mmu-r	ENSMU	154	-21.7	154	-21.7	3068
mmu-r	ENSMU	154	-21.4	154	-21.4	2637
mmu-r	ENSMU	154	-21.3	154	-21.3	286
mmu-r	ENSMU	154	-21.3	154	-21.3	3844
mmu-r	ENSMU	154	-21	154	-21	749
mmu-r	ENSMU	154	-20.9	154	-20.9	10160
mmu-r	ENSMU	154	-20.8	154	-20.8	4046
mmu-r	ENSMU	154	-20.8	154	-20.8	7589
mmu-r	ENSMU	154	-20.8	154	-20.8	1681
mmu-r	ENSMU	154	-20.8	154	-20.8	11885
mmu-r	ENSMU	154	-20.8	154	-20.8	4596
mmu-l	Gm388	154	-20.6	154	-20.6	4318
mmu-r	ENSMU	154	-20.5	154	-20.5	3208
mmu-r	Gm388	154	-20.5	154	-20.5	1641
mmu-r	Gm388	154	-20.5	154	-20.5	7648
mmu-r	ENSMU	154	-20.2	154	-20.2	268
mmu-r	Gm388	154	-20.2	154	-20.2	2869
mmu-r	ENSMU	154	-20.1	154	-20.1	2615
mmu-l	Gm388	154	-20.1	154	-20.1	4322
mmu-r	Gm412	154	-20.1	154	-20.1	3305
mmu-r	Gm412	154	-20.1	154	-20.1	3536
mmu-r	ENSMU	153	-30.7	153	-30.7	1180
mmu-r	ENSMU	153	-28.6	153	-28.6	1659
mmu-r	ENSMU	153	-28.4	153	-28.4	8382
mmu-r	ENSMU	153	-27	153	-27	2684
mmu-r	ENSMU	153	-26.7	153	-26.7	3832

mmu-r	Gm388	153	-26.4	153	-26.4	6479
mmu-r	ENSMU	153	-26.2	153	-26.2	2707
mmu-r	ENSMU	153	-25.9	153	-25.9	522
mmu-r	Gm388	153	-25.8	153	-25.8	7617
mmu-r	ENSMU	153	-25.6	153	-25.6	760
mmu-r	Gm412	153	-25.4	153	-25.4	1096
mmu-r	Gm388	153	-25.1	153	-25.1	423
mmu-r	ENSMU	153	-25.1	153	-25.1	1314
mmu-r	Gm388	153	-24.4	153	-24.4	4156
mmu-r	ENSMU	153	-24.3	153	-24.3	3718
mmu-r	Gm412	153	-24.3	153	-24.3	3712
mmu-r	Gm388	153	-24.2	153	-24.2	1790
mmu-r	Gm388	153	-24.1	153	-24.1	8688
mmu-r	ENSMU	153	-23.9	153	-23.9	2298
mmu-r	ENSMU	153	-23.8	153	-23.8	4470
mmu-r	ENSMU	153	-23.7	153	-23.7	1414
mmu-r	ENSMU	153	-23.5	153	-23.5	4125
mmu-r	Gm388	153	-23.4	153	-23.4	2457
mmu-r	Gm388	153	-23.3	153	-23.3	142
mmu-r	Gm388	153	-23.2	153	-23.2	7899
mmu-r	ENSMU	153	-23	153	-23	2546
mmu-r	ENSMU	153	-22.9	153	-22.9	8144
mmu-r	ENSMU	153	-22.9	153	-22.9	4845
mmu-r	ENSMU	153	-22.8	153	-22.8	615
mmu-r	Gm412	153	-22.7	153	-22.7	1061
mmu-r	Gm412	153	-22.4	153	-22.4	4447
mmu-r	ENSMU	153	-22.3	153	-22.3	895
mmu-r	ENSMU	153	-22.2	153	-22.2	8714
mmu-r	ENSMU	153	-22.1	153	-22.1	1095
mmu-r	Gm412	153	-22.1	153	-22.1	3102
mmu-r	ENSMU	153	-22	153	-22	9842
mmu-r	Gm388	153	-21.9	153	-21.9	4577
mmu-r	Gm412	153	-21.8	153	-21.8	3600
mmu-r	ENSMU	153	-21.6	153	-21.6	9155
mmu-r	ENSMU	153	-21.6	153	-21.6	8752
mmu-r	ENSMU	153	-21.5	153	-21.5	7529
mmu-r	ENSMU	153	-21.3	153	-21.3	2103
mmu-r	Gm412	153	-21.2	153	-21.2	3099
mmu-r	ENSMU	153	-21.2	153	-21.2	10294
mmu-r	ENSMU	153	-21.1	153	-21.1	8579
mmu-r	ENSMU	153	-21.1	153	-21.1	10098
mmu-r	ENSMU	153	-21	153	-21	9058
mmu-r	ENSMU	153	-20.8	153	-20.8	474
mmu-r	ENSMU	153	-20.8	153	-20.8	6325
mmu-r	ENSMU	153	-20.7	153	-20.7	3469
mmu-r	ENSMU	153	-20.7	153	-20.7	2276
mmu-r	ENSMU	153	-20.7	153	-20.7	835
mmu-r	ENSMU	153	-20.6	153	-20.6	904
mmu-r	ENSMU	153	-20.6	153	-20.6	6866
mmu-r	ENSMU	153	-20.6	153	-20.6	8796
mmu-r	ENSMU	153	-20.5	153	-20.5	3376
mmu-r	Gm388	153	-20.5	153	-20.5	1796
mmu-r	ENSMU	153	-20.3	153	-20.3	281
mmu-r	Gm412	153	-20.2	153	-20.2	21
mmu-r	ENSMU	153	-20.2	153	-20.2	10428
mmu-r	ENSMU	153	-20	153	-20	5398
mmu-r	ENSMU	152	-29.8	152	-29.8	785
mmu-r	Gm412	152	-28.1	152	-28.1	4477
mmu-r	ENSMU	152	-27.1	152	-27.1	10289
mmu-r	ENSMU	152	-26.9	152	-26.9	2501
mmu-r	ENSMU	152	-26.5	152	-26.5	1353
mmu-r	ENSMU	152	-26.2	152	-26.2	10418
mmu-r	ENSMU	152	-26.2	152	-26.2	5837
mmu-r	ENSMU	152	-25.8	152	-25.8	1210

mmu-r	Gm388	152	-25.7	152	-25.7	5383
mmu-r	ENSMU	152	-25.7	152	-25.7	10650
mmu-r	ENSMU	152	-25.4	152	-25.4	3097
mmu-r	ENSMU	152	-25.4	152	-25.4	5674
mmu-r	ENSMU	152	-25.3	152	-25.3	324
mmu-r	ENSMU	152	-24.8	152	-24.8	159
mmu-r	ENSMU	152	-24.7	152	-24.7	3396
mmu-r	Gm388	152	-24.7	152	-24.7	1192
mmu-r	ENSMU	152	-24.4	152	-24.4	123
mmu-r	ENSMU	152	-24.2	152	-24.2	682
mmu-r	ENSMU	152	-24.2	152	-24.2	10424
mmu-r	Gm412	152	-24.1	152	-24.1	1706
mmu-r	Gm388	152	-24	152	-24	4533
mmu-r	ENSMU	152	-23.8	152	-23.8	206
mmu-r	ENSMU	152	-23.8	152	-23.8	11030
mmu-r	ENSMU	152	-23.8	152	-23.8	590
mmu-r	ENSMU	152	-23.8	152	-23.8	4680
mmu-r	ENSMU	152	-23.5	152	-23.5	2893
mmu-r	ENSMU	152	-23.2	152	-23.2	1341
mmu-r	ENSMU	152	-23.2	152	-23.2	11107
mmu-r	ENSMU	152	-23	152	-23	1319
mmu-r	ENSMU	152	-22.9	152	-22.9	1217
mmu-r	ENSMU	152	-22.9	152	-22.9	2499
mmu-r	Gm412	152	-22.9	152	-22.9	3916
mmu-r	ENSMU	152	-22.8	152	-22.8	5694
mmu-r	ENSMU	152	-22.8	152	-22.8	7349
mmu-r	ENSMU	152	-22.8	152	-22.8	7711
mmu-r	ENSMU	152	-22.6	152	-22.6	13446
mmu-r	ENSMU	152	-22.4	152	-22.4	5634
mmu-r	Gm388	152	-22.4	152	-22.4	3409
rco-mil	ENSMU	152	-22.4	152	-22.4	2756
rco-mil	ENSMU	152	-22.4	152	-22.4	2756
mmu-r	ENSMU	152	-22.4	152	-22.4	8236
mmu-r	ENSMU	152	-22.3	152	-22.3	8671
mmu-r	ENSMU	152	-22.1	152	-22.1	170
mmu-r	ENSMU	152	-22.1	152	-22.1	3911
mmu-r	ENSMU	152	-22	152	-22	7079
mmu-r	Gm412	152	-22	152	-22	1197
mmu-r	ENSMU	152	-21.9	152	-21.9	1710
mmu-r	ENSMU	152	-21.9	152	-21.9	4642
mmu-r	Gm388	152	-21.8	152	-21.8	2181
rco-mil	ENSMU	152	-21.7	152	-21.7	1667
rco-mil	ENSMU	152	-21.7	152	-21.7	1667
mmu-r	Gm388	152	-21.4	152	-21.4	2276
mmu-r	ENSMU	152	-21.3	152	-21.3	2668
mmu-r	ENSMU	152	-21.2	152	-21.2	1443
mmu-r	ENSMU	152	-20.9	152	-20.9	2949
mmu-r	Gm388	152	-20.9	152	-20.9	7032
mmu-r	ENSMU	152	-20.8	152	-20.8	3871
mmu-r	ENSMU	152	-20.8	152	-20.8	623
mmu-r	ENSMU	152	-20.8	152	-20.8	615
mmu-r	ENSMU	152	-20.6	152	-20.6	101
mmu-r	Gm412	152	-20.6	152	-20.6	320
mmu-r	ENSMU	152	-20.6	152	-20.6	2683
mmu-r	ENSMU	152	-20.5	152	-20.5	5688
mmu-r	ENSMU	152	-20.5	152	-20.5	2977
mmu-r	ENSMU	152	-20.5	152	-20.5	4137
mmu-r	ENSMU	152	-20.3	152	-20.3	5072
mmu-r	Gm412	152	-20.3	152	-20.3	2541
mmu-r	ENSMU	152	-20.3	152	-20.3	4422
mmu-r	ENSMU	152	-20	152	-20	3966
mmu-r	Gm388	152	-20	152	-20	4437
mmu-r	ENSMU	152	-20	152	-20	4065
mmu-r	Gm388	151	-27.1	151	-27.1	8241

mmu-r	Gm388	151	-26.8	151	-26.8	189
mmu-r	Gm388	151	-26.3	151	-26.3	1186
mmu-r	ENSMU	151	-25.8	151	-25.8	3713
mmu-r	Gm388	151	-25.4	151	-25.4	8540
mmu-r	Gm412	151	-25.2	151	-25.2	28
mmu-r	ENSMU	151	-25.1	151	-25.1	267
mmu-r	ENSMU	151	-25	151	-25	10366
mmu-r	ENSMU	151	-24.7	151	-24.7	7922
mmu-r	ENSMU	151	-24.5	151	-24.5	5849
mmu-r	ENSMU	151	-24.2	151	-24.2	4256
mmu-r	Gm388	151	-24.2	151	-24.2	5107
mmu-r	Gm412	151	-24.2	151	-24.2	1420
mmu-r	Gm412	151	-24	151	-24	1153
mmu-r	ENSMU	151	-23.8	151	-23.8	1658
mmu-r	Gm412	151	-23.7	151	-23.7	352
mmu-r	ENSMU	151	-23.6	151	-23.6	1933
mmu-r	ENSMU	151	-23.6	151	-23.6	1202
mmu-r	Gm412	151	-23.4	151	-23.4	1775
mmu-r	ENSMU	151	-23.4	151	-23.4	1175
mmu-r	ENSMU	151	-23.1	151	-23.1	4278
mmu-r	Gm388	151	-23	151	-23	9045
mmu-r	ENSMU	151	-22.8	151	-22.8	11122
mmu-r	Gm412	151	-22.8	151	-22.8	2439
mmu-r	ENSMU	151	-22.6	151	-22.6	2057
mmu-r	Gm388	151	-22.5	151	-22.5	5201
mmu-r	ENSMU	151	-22.1	151	-22.1	2132
mmu-r	ENSMU	151	-22.1	151	-22.1	1006
mmu-r	ENSMU	151	-21.9	151	-21.9	3758
mmu-r	Gm412	151	-21.8	151	-21.8	3989
mmu-r	ENSMU	151	-21.8	151	-21.8	3684
mmu-r	ENSMU	151	-21.7	151	-21.7	7859
mmu-r	ENSMU	151	-21.6	151	-21.6	902
mmu-r	ENSMU	151	-21.6	151	-21.6	902
mmu-r	ENSMU	151	-21.6	151	-21.6	171
mmu-r	ENSMU	151	-21.6	151	-21.6	1163
mmu-r	ENSMU	151	-21.5	151	-21.5	490
mmu-r	ENSMU	151	-21.5	151	-21.5	2255
mmu-r	Gm388	151	-21.4	151	-21.4	433
mmu-r	Gm388	151	-21.3	151	-21.3	8382
mmu-r	ENSMU	151	-21.3	151	-21.3	6454
mmu-r	Gm388	151	-21.2	151	-21.2	149
mmu-r	Gm412	151	-21.1	151	-21.1	297
mmu-r	ENSMU	151	-21	151	-21	1217
mmu-r	Gm388	151	-21	151	-21	8235
mmu-r	ENSMU	151	-20.9	151	-20.9	10338
mmu-r	ENSMU	151	-20.8	151	-20.8	9153
mmu-r	ENSMU	151	-20.7	151	-20.7	1896
mmu-r	ENSMU	151	-20.5	151	-20.5	4908
mmu-r	ENSMU	151	-20.4	151	-20.4	1636
mmu-r	Gm412	151	-20.3	151	-20.3	1757
mmu-r	ENSMU	151	-20.3	151	-20.3	8573
mmu-r	ENSMU	151	-20.2	151	-20.2	863
rco-mil	ENSMU	151	-20.2	151	-20.2	3365
mmu-r	Gm388	151	-20.1	151	-20.1	8990
mmu-r	ENSMU	151	-20.1	151	-20.1	9628
mmu-r	ENSMU	151	-20	151	-20	491
mmu-r	ENSMU	151	-20	151	-20	4882
mmu-r	ENSMU	150	-33.7	150	-33.7	532
mmu-r	ENSMU	150	-28	150	-28	1215
mmu-r	Gm388	150	-27.3	150	-27.3	7483
mmu-r	Gm388	150	-27.1	150	-27.1	8831
mmu-r	ENSMU	150	-26.6	150	-26.6	4075
mmu-r	Gm388	150	-25.6	150	-25.6	8259
mmu-r	ENSMU	150	-25.2	150	-25.2	1238

mmu-r	Gm388	150	-24.8	150	-24.8	2382
mmu-r	ENSMU	150	-24.7	150	-24.7	2382
mmu-r	ENSMU	150	-24.5	150	-24.5	1089
mmu-r	ENSMU	150	-24	150	-24	831
mmu-r	ENSMU	150	-23.9	150	-23.9	5877
mmu-r	ENSMU	150	-23.6	150	-23.6	1161
mmu-r	ENSMU	150	-23.4	150	-23.4	1719
mmu-r	ENSMU	150	-23.4	150	-23.4	6958
mmu-r	Gm388	150	-23.3	150	-23.3	6123
mmu-r	Gm388	150	-23.3	150	-23.3	158
mmu-r	ENSMU	150	-23	150	-23	347
mmu-r	Gm388	150	-22.9	150	-22.9	7065
mmu-r	Gm412	150	-22.8	150	-22.8	2882
mmu-r	ENSMU	150	-22.6	150	-22.6	1484
mmu-r	ENSMU	150	-22.5	150	-22.5	3886
mmu-r	ENSMU	150	-22.5	150	-22.5	4675
mmu-r	Gm388	150	-22.4	150	-22.4	8441
mmu-r	ENSMU	150	-22.3	150	-22.3	2735
mmu-r	Gm412	150	-22.2	150	-22.2	4414
mmu-r	ENSMU	150	-21.8	150	-21.8	2041
mmu-r	Gm388	150	-21.8	150	-21.8	1781
mmu-r	ENSMU	150	-21.8	150	-21.8	5634
mmu-r	ENSMU	150	-21.7	150	-21.7	5131
mmu-r	Gm412	150	-21.7	150	-21.7	1854
mmu-r	ENSMU	150	-21.6	150	-21.6	5658
mmu-r	Gm412	150	-21.5	150	-21.5	2041
mmu-r	ENSMU	150	-21.4	150	-21.4	3449
mmu-r	Gm388	150	-21.4	150	-21.4	1479
mmu-r	ENSMU	150	-21.1	150	-21.1	1499
mmu-r	ENSMU	150	-21.1	150	-21.1	142
mmu-r	ENSMU	150	-21.1	150	-21.1	814
mmu-r	Gm388	150	-20.9	150	-20.9	7818
mmu-r	ENSMU	150	-20.8	150	-20.8	1560
mmu-r	ENSMU	150	-20.8	150	-20.8	3716
mmu-r	ENSMU	150	-20.7	150	-20.7	5869
mmu-r	ENSMU	150	-20.7	150	-20.7	7894
mmu-r	ENSMU	150	-20.6	150	-20.6	3333
mmu-l	ENSMU	150	-20.5	150	-20.5	9433
mmu-r	ENSMU	150	-20.5	150	-20.5	5486
mmu-r	ENSMU	150	-20.5	150	-20.5	3208
mmu-r	ENSMU	150	-20.5	150	-20.5	7151
mmu-r	ENSMU	150	-20.3	150	-20.3	392
rco-mi	ENSMU	150	-20.2	150	-20.2	6578
mmu-r	ENSMU	150	-20.2	150	-20.2	1366
mmu-r	ENSMU	150	-20.2	150	-20.2	3896
mmu-r	ENSMU	150	-20.1	150	-20.1	5804
mmu-r	ENSMU	150	-20.1	150	-20.1	999
mmu-r	Gm388	150	-20	150	-20	133
mmu-r	Gm412	149	-29.2	149	-29.2	1062
mmu-r	ENSMU	149	-28.3	149	-28.3	2266
mmu-r	Gm388	149	-27.5	149	-27.5	7407
mmu-r	ENSMU	149	-26.2	149	-26.2	3483
mmu-r	ENSMU	149	-25.1	149	-25.1	1555
mmu-r	Gm412	149	-24.5	149	-24.5	352
mmu-r	ENSMU	149	-24.4	149	-24.4	3886
mmu-r	ENSMU	149	-24.1	149	-24.1	3469
mmu-r	ENSMU	149	-24	149	-24	834
mmu-r	ENSMU	149	-23.8	149	-23.8	8149
mmu-r	Gm412	149	-23.5	149	-23.5	1642
mmu-r	ENSMU	149	-23.5	149	-23.5	3991
mmu-r	ENSMU	149	-23.2	149	-23.2	3315
mmu-r	Gm412	149	-23.2	149	-23.2	3768
mmu-r	ENSMU	149	-23.2	149	-23.2	12704
mmu-r	Gm412	149	-22.8	149	-22.8	1099

mmu-r	ENSMU	149	-22.7	149	-22.7	10847
mmu-r	ENSMU	149	-22.2	149	-22.2	4421
mmu-r	Gm412	149	-22.2	149	-22.2	3157
mmu-r	ENSMU	149	-22.2	149	-22.2	271
mmu-r	ENSMU	149	-21.9	149	-21.9	3718
mmu-r	ENSMU	149	-21.8	149	-21.8	3111
mmu-r	ENSMU	149	-21.8	149	-21.8	12768
mmu-r	ENSMU	149	-21.8	149	-21.8	8749
mmu-r	ENSMU	149	-21.8	149	-21.8	706
mmu-r	ENSMU	149	-21.8	149	-21.8	9026
mmu-r	ENSMU	149	-21.4	149	-21.4	8985
mmu-r	ENSMU	149	-21.4	149	-21.4	14037
mmu-r	ENSMU	149	-21.3	149	-21.3	1112
mmu-r	Gm388	149	-21.2	149	-21.2	8993
mmu-r	Gm388	149	-21.1	149	-21.1	6633
mmu-r	ENSMU	149	-21.1	149	-21.1	4647
mmu-r	ENSMU	149	-20.9	149	-20.9	1971
mmu-r	Gm388	149	-20.7	149	-20.7	8830
mmu-r	Gm412	149	-20.6	149	-20.6	2651
mmu-r	Gm388	149	-20.6	149	-20.6	7331
mmu-r	ENSMU	149	-20.5	149	-20.5	7990
mmu-r	Gm388	149	-20.3	149	-20.3	8626
mmu-r	Gm388	149	-20.1	149	-20.1	946
mmu-r	Gm388	149	-20.1	149	-20.1	4998
hiv1-m	Gm388	149	-20.1	149	-20.1	8489
mmu-r	ENSMU	148	-29.4	148	-29.4	334
mmu-r	ENSMU	148	-28.1	148	-28.1	3993
mmu-r	ENSMU	148	-26.7	148	-26.7	2391
mmu-r	ENSMU	148	-25.8	148	-25.8	8786
mmu-r	ENSMU	148	-25.5	148	-25.5	4326
mmu-r	ENSMU	148	-25.3	148	-25.3	2545
mmu-r	Gm388	148	-25.3	148	-25.3	181
mmu-r	ENSMU	148	-24.8	148	-24.8	7137
mmu-r	Gm388	148	-24.8	148	-24.8	8539
mmu-r	ENSMU	148	-24.6	148	-24.6	5393
mmu-r	ENSMU	148	-24.5	148	-24.5	3919
mmu-r	ENSMU	148	-24.3	148	-24.3	664
mmu-r	ENSMU	148	-23.8	148	-23.8	14038
mmu-r	Gm412	148	-23.7	148	-23.7	1642
mmu-r	ENSMU	148	-23.7	148	-23.7	3329
mmu-r	ENSMU	148	-23.6	148	-23.6	8787
mmu-r	Gm388	148	-23.1	148	-23.1	7799
mmu-r	ENSMU	148	-23.1	148	-23.1	1097
mmu-r	ENSMU	148	-23	148	-23	8143
mmu-r	ENSMU	148	-22.8	148	-22.8	3913
mmu-r	ENSMU	148	-22.5	148	-22.5	5944
mmu-r	ENSMU	148	-22.4	148	-22.4	6960
mmu-r	ENSMU	148	-22.4	148	-22.4	7365
mmu-r	Gm412	148	-22.3	148	-22.3	1049
mmu-r	ENSMU	148	-22.3	148	-22.3	2556
mmu-r	ENSMU	148	-22.2	148	-22.2	2664
mmu-r	ENSMU	148	-22.2	148	-22.2	5999
mmu-r	ENSMU	148	-22.1	148	-22.1	796
mmu-r	ENSMU	148	-21.8	148	-21.8	2351
mmu-r	ENSMU	148	-21.7	148	-21.7	7058
mmu-r	Gm388	148	-21.6	148	-21.6	5213
mmu-r	ENSMU	148	-21.6	148	-21.6	8151
mmu-r	ENSMU	148	-21.5	148	-21.5	1242
mmu-r	ENSMU	148	-21.4	148	-21.4	7021
mmu-r	ENSMU	148	-21.2	148	-21.2	361
mmu-r	ENSMU	148	-21.1	148	-21.1	5855
mmu-r	ENSMU	148	-21.1	148	-21.1	8139
mmu-r	ENSMU	148	-21	148	-21	11237
mmu-r	ENSMU	148	-20.9	148	-20.9	504

mmu-r	ENSMU	148	-20.9	148	-20.9	1091
mmu-r	ENSMU	148	-20.9	148	-20.9	14165
mmu-r	ENSMU	148	-20.8	148	-20.8	7715
mmu-r	ENSMU	148	-20.7	148	-20.7	17
mmu-r	ENSMU	148	-20.6	148	-20.6	6959
mmu-r	ENSMU	148	-20.6	148	-20.6	5645
mmu-r	ENSMU	148	-20.5	148	-20.5	5871
mmu-r	Gm388	148	-20.5	148	-20.5	8676
mmu-r	Gm388	148	-20.4	148	-20.4	1422
mmu-r	ENSMU	148	-20.3	148	-20.3	1884
mmu-r	ENSMU	148	-20.3	148	-20.3	313
mmu-r	ENSMU	148	-20.3	148	-20.3	8435
mmu-r	ENSMU	148	-20.2	148	-20.2	695
mmu-r	ENSMU	148	-20.1	148	-20.1	4641
mmu-r	ENSMU	148	-20.1	148	-20.1	89
mmu-r	Gm388	148	-20	148	-20	2780
mmu-r	ENSMU	147	-28.8	147	-28.8	4364
mmu-r	ENSMU	147	-28.7	147	-28.7	608
mmu-r	ENSMU	147	-27.5	147	-27.5	2314
mmu-r	ENSMU	147	-25.5	147	-25.5	2362
mmu-r	ENSMU	147	-25.3	147	-25.3	4186
mmu-r	ENSMU	147	-25.1	147	-25.1	2272
mmu-r	ENSMU	147	-24.8	147	-24.8	1110
mmu-r	ENSMU	147	-24.8	147	-24.8	1630
mmu-r	ENSMU	147	-24.6	147	-24.6	2848
mmu-r	ENSMU	147	-24.6	147	-24.6	2848
mmu-r	ENSMU	147	-24.2	147	-24.2	5797
mmu-r	Gm388	147	-24	147	-24	7401
rco-mil	ENSMU	147	-23.4	147	-23.4	3459
rco-mil	ENSMU	147	-23.4	147	-23.4	3459
rco-mil	ENSMU	147	-23.4	147	-23.4	3459
mmu-r	Gm388	147	-23	147	-23	3987
mmu-r	Gm388	147	-22.8	147	-22.8	3784
mmu-r	ENSMU	147	-22.8	147	-22.8	3883
mmu-r	ENSMU	147	-22.4	147	-22.4	11692
mmu-r	ENSMU	147	-22.2	147	-22.2	2399
mmu-r	ENSMU	147	-22.1	147	-22.1	2167
mmu-r	Gm388	147	-22.1	147	-22.1	7608
mmu-r	ENSMU	147	-21.8	147	-21.8	1207
mmu-r	ENSMU	147	-21.8	147	-21.8	2229
mmu-r	Gm412	147	-21.7	147	-21.7	2431
mmu-r	ENSMU	147	-21.7	147	-21.7	1363
mmu-r	Gm388	147	-21.3	147	-21.3	8295
mmu-r	ENSMU	147	-21.2	147	-21.2	1561
mmu-r	Gm388	147	-21.1	147	-21.1	8523
mmu-r	ENSMU	147	-21.1	147	-21.1	10901
rco-mil	ENSMU	147	-21.1	147	-21.1	3459
mmu-r	ENSMU	147	-21	147	-21	4648
mmu-r	Gm388	147	-20.9	147	-20.9	1442
mmu-r	ENSMU	147	-20.8	147	-20.8	1240
mmu-r	ENSMU	147	-20.7	147	-20.7	425
mmu-r	ENSMU	147	-20.6	147	-20.6	1206
mmu-r	ENSMU	147	-20.6	147	-20.6	3342
mmu-r	Gm412	147	-20.6	147	-20.6	1312
mmu-r	ENSMU	147	-20.6	147	-20.6	4864
mmu-r	ENSMU	147	-20.5	147	-20.5	1672
mmu-r	ENSMU	147	-20.5	147	-20.5	782
mmu-r	ENSMU	147	-20.5	147	-20.5	1709
mmu-r	ENSMU	147	-20.3	147	-20.3	2065
mmu-r	ENSMU	147	-20.1	147	-20.1	612
mmu-r	ENSMU	147	-20	147	-20	1882
mmu-r	ENSMU	146	-28.1	146	-28.1	4257
mmu-r	Gm388	146	-26.8	146	-26.8	8183
mmu-r	Gm412	146	-26.6	146	-26.6	1035

mmu-r	ENSMU	146	-26.5	146	-26.5	3213
mmu-r	ENSMU	146	-25.9	146	-25.9	269
mmu-r	ENSMU	146	-25.5	146	-25.5	1212
mmu-r	ENSMU	146	-25.1	146	-25.1	506
mmu-r	ENSMU	146	-24.7	146	-24.7	2837
mmu-r	Gm388	146	-24.6	146	-24.6	7064
mmu-r	ENSMU	146	-24.3	146	-24.3	2448
mmu-r	ENSMU	146	-23.7	146	-23.7	1358
mmu-r	ENSMU	146	-23.2	146	-23.2	3890
mmu-r	Gm412	146	-23.1	146	-23.1	2574
mmu-r	ENSMU	146	-22.9	146	-22.9	5870
mmu-r	ENSMU	146	-22.9	146	-22.9	2645
mmu-r	ENSMU	146	-22.9	146	-22.9	1202
mmu-r	ENSMU	146	-22.5	146	-22.5	3934
mmu-r	Gm412	146	-22.4	146	-22.4	1308
mmu-r	Gm388	146	-22.2	146	-22.2	8440
mmu-r	ENSMU	146	-22.1	146	-22.1	6085
mmu-r	ENSMU	146	-22	146	-22	4604
mmu-r	Gm412	146	-21.8	146	-21.8	3100
mmu-r	Gm388	146	-21.8	146	-21.8	2441
mmu-r	Gm412	146	-21.8	146	-21.8	4408
mmu-r	Gm388	146	-21.6	146	-21.6	4010
mmu-r	ENSMU	146	-21.6	146	-21.6	2600
mmu-r	ENSMU	146	-21.5	146	-21.5	10611
mmu-r	ENSMU	146	-21.4	146	-21.4	5485
mmu-r	ENSMU	146	-21.4	146	-21.4	2652
mmu-r	ENSMU	146	-21.2	146	-21.2	5815
mmu-r	ENSMU	146	-21.2	146	-21.2	9102
mmu-r	ENSMU	146	-21.1	146	-21.1	2391
mmu-r	ENSMU	146	-21	146	-21	3356
mmu-r	ENSMU	146	-20.9	146	-20.9	581
mmu-r	ENSMU	146	-20.9	146	-20.9	10066
mmu-r	Gm412	146	-20.9	146	-20.9	947
mmu-r	ENSMU	146	-20.8	146	-20.8	3892
mmu-r	ENSMU	146	-20.8	146	-20.8	1351
mmu-r	ENSMU	146	-20.7	146	-20.7	5743
mmu-r	ENSMU	146	-20.5	146	-20.5	5282
mmu-r	ENSMU	146	-20.5	146	-20.5	2247
mmu-r	Gm388	146	-20.4	146	-20.4	8849
mmu-r	Gm388	146	-20.2	146	-20.2	6401
mmu-r	Gm388	146	-20.2	146	-20.2	2183
mmu-r	ENSMU	146	-20	146	-20	5819
mmu-r	ENSMU	145	-27.1	145	-27.1	12115
mmu-r	ENSMU	145	-25.6	145	-25.6	690
mmu-r	Gm388	145	-25.1	145	-25.1	5415
mmu-r	Gm412	145	-25	145	-25	410
mmu-r	ENSMU	145	-24.5	145	-24.5	2519
mmu-r	ENSMU	145	-23.8	145	-23.8	9034
mmu-r	ENSMU	145	-23.5	145	-23.5	9019
mmu-r	ENSMU	145	-23.4	145	-23.4	8969
mmu-r	ENSMU	145	-22.8	145	-22.8	4665
mmu-r	ENSMU	145	-22.3	145	-22.3	52
mmu-r	ENSMU	145	-22.3	145	-22.3	5290
mmu-r	ENSMU	145	-22.3	145	-22.3	816
mmu-r	Gm412	145	-22.1	145	-22.1	352
mmu-r	ENSMU	145	-22	145	-22	2390
mmu-r	ENSMU	145	-22	145	-22	1336
mmu-r	ENSMU	145	-21.6	145	-21.6	2407
mmu-r	Gm388	145	-21.6	145	-21.6	2075
mmu-r	Gm412	145	-21.4	145	-21.4	1305
mmu-r	ENSMU	145	-21.4	145	-21.4	1883
mmu-r	ENSMU	145	-21.2	145	-21.2	13737
mmu-r	ENSMU	145	-20.8	145	-20.8	2160
mmu-r	ENSMU	145	-20.8	145	-20.8	5999

mmu-r	ENSMU	145	-20.7	145	-20.7	9001
mmu-r	ENSMU	145	-20.7	145	-20.7	1121
mmu-r	Gm412	145	-20.6	145	-20.6	12
mmu-r	ENSMU	145	-20.6	145	-20.6	1351
mmu-r	ENSMU	145	-20.6	145	-20.6	2695
mmu-r	ENSMU	145	-20.3	145	-20.3	9154
mmu-r	Gm388	145	-20.2	145	-20.2	1792
mmu-r	ENSMU	145	-20.1	145	-20.1	1162
mmu-r	Gm412	145	-20.1	145	-20.1	4058
mmu-r	ENSMU	145	-20.1	145	-20.1	9038
mmu-r	Gm412	144	-27.5	144	-27.5	2685
mmu-r	ENSMU	144	-23.6	144	-23.6	629
mmu-r	ENSMU	144	-23.2	144	-23.2	5386
mmu-r	ENSMU	144	-23.1	144	-23.1	3016
mmu-r	ENSMU	144	-23	144	-23	521
mmu-r	ENSMU	144	-22.6	144	-22.6	6715
mmu-r	Gm388	144	-22.6	144	-22.6	8023
mmu-r	Gm388	144	-22.2	144	-22.2	1188
mmu-r	ENSMU	144	-22	144	-22	5986
rco-mil	ENSMU	144	-21.9	144	-21.9	2268
mmu-r	Gm388	144	-21.9	144	-21.9	8387
rco-mil	ENSMU	144	-21.5	144	-21.5	2268
rco-mil	ENSMU	144	-21.5	144	-21.5	2268
mmu-r	Gm388	144	-21.3	144	-21.3	6628
mmu-r	ENSMU	144	-21.2	144	-21.2	1711
mmu-r	Gm412	144	-21	144	-21	713
mmu-r	ENSMU	144	-20.9	144	-20.9	8575
mmu-r	Gm412	144	-20.8	144	-20.8	1778
mmu-r	Gm388	144	-20.7	144	-20.7	3983
mmu-r	ENSMU	144	-20.7	144	-20.7	1961
mmu-r	ENSMU	144	-20.7	144	-20.7	9738
mmu-r	ENSMU	144	-20.7	144	-20.7	6715
mmu-r	ENSMU	144	-20.6	144	-20.6	8601
mmu-r	ENSMU	144	-20.6	144	-20.6	5871
mmu-r	Gm412	144	-20.5	144	-20.5	2960
mmu-r	ENSMU	144	-20.5	144	-20.5	2298
mmu-r	ENSMU	144	-20.3	144	-20.3	10275
rco-mil	ENSMU	144	-20.2	144	-20.2	2756
mmu-r	ENSMU	143	-28.8	143	-28.8	2198
mmu-r	ENSMU	143	-25.6	143	-25.6	3090
mmu-r	ENSMU	143	-25.2	143	-25.2	5784
mmu-r	ENSMU	143	-23.8	143	-23.8	14095
mmu-r	Gm388	143	-23.5	143	-23.5	8441
mmu-r	ENSMU	143	-22.7	143	-22.7	5821
mmu-r	ENSMU	143	-22.4	143	-22.4	1551
mmu-r	ENSMU	143	-21.4	143	-21.4	2958
mmu-r	ENSMU	143	-21	143	-21	3916
mmu-r	ENSMU	143	-20.9	143	-20.9	8848
mmu-r	Gm388	143	-20.6	143	-20.6	811
mmu-r	ENSMU	143	-20.5	143	-20.5	169
mmu-r	ENSMU	143	-20.4	143	-20.4	479
mmu-r	ENSMU	143	-20.1	143	-20.1	564
mmu-r	ENSMU	142	-27	142	-27	843
mmu-r	Gm388	142	-26.7	142	-26.7	8480
mmu-r	ENSMU	142	-26.2	142	-26.2	797
mmu-r	ENSMU	142	-25.1	142	-25.1	1007
mmu-r	ENSMU	142	-24.2	142	-24.2	299
mmu-r	ENSMU	142	-24	142	-24	13507
mmu-r	ENSMU	142	-23.3	142	-23.3	2170
mmu-r	ENSMU	142	-23.2	142	-23.2	5783
mmu-r	ENSMU	142	-22.9	142	-22.9	3996
mmu-r	ENSMU	142	-22.3	142	-22.3	3891
mmu-r	Gm412	142	-22.2	142	-22.2	1311
mmu-r	ENSMU	142	-22.2	142	-22.2	54

mmu-r	ENSMU	142	-22.1	142	-22.1	2795
mmu-r	ENSMU	142	-22	142	-22	2823
mmu-r	ENSMU	142	-21.8	142	-21.8	3832
mmu-r	Gm388	142	-21.6	142	-21.6	5428
mmu-r	ENSMU	142	-21.6	142	-21.6	2122
mmu-r	Gm412	142	-21.5	142	-21.5	1318
mmu-r	ENSMU	142	-21.4	142	-21.4	618
mmu-r	ENSMU	142	-21.2	142	-21.2	13436
mmu-r	ENSMU	142	-21.2	142	-21.2	2450
mmu-r	ENSMU	142	-21.1	142	-21.1	4304
mmu-r	ENSMU	142	-20.9	142	-20.9	12332
mmu-r	Gm388	142	-20.9	142	-20.9	2551
mmu-r	Gm412	142	-20.5	142	-20.5	1712
mmu-r	ENSMU	142	-20.1	142	-20.1	4223
mmu-r	ENSMU	142	-20.1	142	-20.1	785
mmu-r	ENSMU	142	-20.1	142	-20.1	514
mmu-r	Gm388	142	-20	142	-20	183
mmu-r	ENSMU	141	-24.2	141	-24.2	3871
mmu-r	ENSMU	141	-23.7	141	-23.7	1988
mmu-r	ENSMU	141	-21.8	141	-21.8	3410
mmu-r	ENSMU	141	-21.7	141	-21.7	10423
mmu-r	ENSMU	141	-21.6	141	-21.6	1237
mmu-r	ENSMU	141	-21.1	141	-21.1	179
mmu-r	ENSMU	141	-20.9	141	-20.9	3723
mmu-r	ENSMU	141	-20.8	141	-20.8	3215
mmu-r	ENSMU	141	-20.1	141	-20.1	1360
mmu-r	ENSMU	140	-26.2	140	-26.2	11809
mmu-r	ENSMU	140	-24.4	140	-24.4	9037
mmu-r	ENSMU	140	-23.8	140	-23.8	582
mmu-r	ENSMU	140	-23.7	140	-23.7	1102
mmu-r	ENSMU	140	-23.2	140	-23.2	3790
mmu-r	Gm412	140	-23.1	140	-23.1	28
mmu-r	ENSMU	140	-21.9	140	-21.9	8883
mmu-r	ENSMU	140	-21.9	140	-21.9	8225
mmu-r	ENSMU	140	-21.8	140	-21.8	10514
mmu-r	Gm388	140	-21.1	140	-21.1	6664
mmu-r	ENSMU	140	-20.8	140	-20.8	2509
mmu-r	ENSMU	140	-20.8	140	-20.8	10423
mmu-r	ENSMU	140	-20.8	140	-20.8	5300
mmu-r	ENSMU	140	-20.7	140	-20.7	1175
mmu-r	ENSMU	140	-20.7	140	-20.7	1176
mmu-r	ENSMU	140	-20.6	140	-20.6	10086
mmu-r	ENSMU	140	-20.4	140	-20.4	13455
mmu-r	ENSMU	140	-20.4	140	-20.4	9040
mmu-r	ENSMU	140	-20.3	140	-20.3	9178
mmu-r	Gm412	140	-20	140	-20	4552
