# Supplementary material for: Comparative transcription profiling of mRNA and lncRNA in pulmonary arterial hypertension after C75 treatment
Source: BMC Pulm Med. 2023 Jan 31;23:46. doi: 10.1186/s12890-023-02334-6 (PMC9887911; doi:10.1186/s12890-023-02334-6)
Supplement: Supplementary file 3 — Additional file 3. Table S3. List of filtered 259 lncRNA-miRNA relationship pairs (in the attachment of supporting information). [file 12890_2023_2334_MOESM3_ESM.pdf]

miRNA	lncRNA	Tot Score	Tot Energy
mmu-miR	ENSMUSG	2261	-254.86
mmu-miR	Gm38850	2016	-263.97
mmu-miR	Gm38850	2000	-254.68
mmu-miR	ENSMUSG	1894	-226.48
mmu-miR	ENSMUSG	1844	-82.11
mmu-miR	Gm38850	1812	-257.76
mmu-miR	Gm38850	1810	-203.56
mmu-miR	Gm38850	1730	-254.57
mmu-miR	Gm38850	1721	-299.48
mmu-miR	ENSMUSG	1692	-144.08
mmu-miR	Gm38850	1686	-295.53
mmu-miR	Gm38850	1641	-244.23
mmu-miR	Gm38850	1637	-230.11
mmu-miR	Gm38850	1627	-139.48
mmu-miR	Gm38850	1625	-191.33
mmu-miR	Gm38850	1592	-154.53
mmu-miR	ENSMUSG	1571	-245.72
mmu-miR	ENSMUSG	1554	-142.18
mmu-miR	Gm38850	1545	-171.15
mmu-miR	Gm38850	1533	-233.24
mmu-miR	ENSMUSG	1519	-294.02
mmu-miR	ENSMUSG	1490	-136.91
mmu-miR	ENSMUSG	1489	-144.63
mmu-miR	ENSMUSG	1482	-109.14
mmu-miR	ENSMUSG	1466	-167.41
mmu-miR	ENSMUSG	1464	-210.78
mmu-miR	Gm38850	1434	-187.17
mmu-miR	ENSMUSG	1424	-115.79
mmu-miR	ENSMUSG	1418	-98.58
mmu-miR	ENSMUSG	1417	-128.46
mmu-miR	ENSMUSG	1385	-168.48
mmu-miR	ENSMUSG	1384	-190.15
mmu-miR	Gm38850	1383	-195.33
mmu-miR	ENSMUSG	1382	-125.36
mmu-miR	ENSMUSG	1363	-160.94
mmu-miR	ENSMUSG	1324	-112.9
mmu-miR	ENSMUSG	1322	-127.53
mmu-miR	ENSMUSG	1317	-160.4
mmu-miR	ENSMUSG	1311	-124.81
mmu-miR	ENSMUSG	1297	-197.95
mmu-miR	Gm38850	1281	-193.8
mmu-miR	ENSMUSG	1281	-106.6
mmu-miR	ENSMUSG	1262	-151.82
mmu-miR	ENSMUSG	1251	-139.95
mmu-miR	ENSMUSG	1246	-219.48
mmu-miR	ENSMUSG	1239	-106.28
mmu-miR	Gm38850	1228	-130.91
mmu-miR	Gm38850	1217	-94.39
mmu-miR	ENSMUSG	1216	-104.82
mmu-miR	ENSMUSG	1203	-166.89
mmu-miR	ENSMUSG	1201	-136.48
mmu-miR	ENSMUSG	1196	-98.23
mmu-miR	ENSMUSG	1175	-213.32
mmu-miR	ENSMUSG	1171	-135.79
mmu-miR	ENSMUSG	1161	-150.77
mmu-miR	ENSMUSG	1151	-134.62
mmu-miR	ENSMUSG	1129	-142.17

mmu-miR	ENSMUSG	1116	-217.65
mmu-miR	ENSMUSG	1088	-138.12
mmu-miR	ENSMUSG	1074	-167.64
mmu-miR	ENSMUSG	1072	-130.03
mmu-miR	ENSMUSG	1066	-133.36
mmu-miR	ENSMUSG	1066	-115.1
mmu-miR	ENSMUSG	1062	-95.06
mmu-miR	ENSMUSG	1056	-151.85
mmu-miR	ENSMUSG	1055	-92.41
mmu-miR	ENSMUSG	1054	-156.94
mmu-miR	ENSMUSG	1054	-151.43
mmu-miR	ENSMUSG	1032	-90.07
mmu-miR	ENSMUSG	1032	-90.07
rco-miR39	ENSMUSG	995	-103.22
rco-miR39	ENSMUSG	992	-90.05
mmu-miR	ENSMUSG	962	-170.35
mmu-miR	ENSMUSG	956	-126.08
mmu-miR	ENSMUSG	956	-111.12
mmu-miR	Gm41235	952	-153.07
mmu-miR	ENSMUSG	952	-28.75
mmu-miR	ENSMUSG	946	-143.18
mmu-miR	ENSMUSG	946	-95.54
mmu-miR	ENSMUSG	942	-64.37
mmu-miR	ENSMUSG	942	-64.37
mmu-miR	ENSMUSG	937	-109.28
mmu-miR	ENSMUSG	933	-63.04
mmu-miR	ENSMUSG	933	-63.04
mmu-miR	ENSMUSG	933	-63.04
mmu-miR	ENSMUSG	932	-59.96
mmu-miR	ENSMUSG	932	-36.29
mmu-miR	ENSMUSG	929	-125.49
mmu-miR	ENSMUSG	927	-146.72
mmu-miR	ENSMUSG	925	-156.55
mmu-miR	ENSMUSG	920	-148.7
mmu-miR	ENSMUSG	916	-37.55
mmu-miR	ENSMUSG	915	-61.62
mmu-miR	ENSMUSG	914	-117.79
mmu-miR	ENSMUSG	911	-57.01
mmu-miR	ENSMUSG	910	-136.05
mmu-miR	ENSMUSG	909	-132.77
mmu-miR	ENSMUSG	909	-117.5
mmu-miR	ENSMUSG	907	-145.29
mmu-miR	ENSMUSG	906	-142.11
mmu-miR	ENSMUSG	904	-102.41
mmu-miR	ENSMUSG	902	-82.11
mmu-miR	ENSMUSG	898	-98.54
mmu-miR	ENSMUSG	890	-127.73
mmu-miR	ENSMUSG	890	-93.44
mmu-miR	Gm38850	888	-142.02
mmu-miR	ENSMUSG	888	-127.44
mmu-miR	Gm38850	887	-104.44
mmu-miR	ENSMUSG	884	-60.12
mmu-miR	ENSMUSG	883	-64.53
mmu-miR	ENSMUSG	881	-135.04
mmu-miR	ENSMUSG	880	-82.05
mmu-miR	ENSMUSG	878	-127.11
mmu-miR	ENSMUSG	875	-91.98
mmu-miR	ENSMUSG	874	-77.62

mmu-miR	ENSMUSG	868	-86.04
mmu-miR	ENSMUSG	868	-53.44
mmu-miR	ENSMUSG	867	-98.41
mmu-miR	ENSMUSG	860	-116.01
mmu-miR	ENSMUSG	854	-126.36
mmu-miR	ENSMUSG	840	-138.84
mmu-miR	ENSMUSG	826	-133.99
mmu-miR	ENSMUSG	820	-160.15
mmu-miR	ENSMUSG	811	-168.83
mmu-miR	ENSMUSG	810	-112.7
mmu-miR	ENSMUSG	803	-100.39
mmu-miR	ENSMUSG	801	-113.01
mmu-miR	ENSMUSG	798	-79.49
mmu-miR	ENSMUSG	795	-130.98
mmu-miR	Gm41235	795	-81.65
mmu-miR	ENSMUSG	792	-94.94
mmu-miR	ENSMUSG	792	-90.38
mmu-miR	ENSMUSG	791	-118.01
mmu-miR	Gm38850	789	-108.19
mmu-miR	ENSMUSG	786	-89.67
mmu-miR	ENSMUSG	784	-89.51
mmu-miR	ENSMUSG	783	-103.18
mmu-miR	ENSMUSG	781	-132.07
mmu-miR	ENSMUSG	781	-109.82
mmu-miR	ENSMUSG	780	-112.78
mmu-miR	Gm38850	779	-130.89
mmu-miR	ENSMUSG	779	-110.46
mmu-miR	ENSMUSG	778	-61.26
mmu-miR	ENSMUSG	777	-112.77
mmu-miR	ENSMUSG	776	-109.35
mmu-miR	ENSMUSG	776	-79.69
mmu-miR	ENSMUSG	776	-79.69
mmu-miR	ENSMUSG	776	-77.01
mmu-miR	Gm38850	775	-97.71
mmu-miR	ENSMUSG	773	-111.92
mmu-miR	ENSMUSG	771	-139.37
mmu-miR	ENSMUSG	769	-110.38
mmu-miR	ENSMUSG	769	-102.25
mmu-miR	Gm38850	768	-95.2
mmu-miR	ENSMUSG	768	-67.38
mmu-miR	ENSMUSG	767	-110.65
mmu-miR	ENSMUSG	767	-87.29
mmu-miR	ENSMUSG	767	-83.6
mmu-miR	ENSMUSG	767	-80.69
mmu-miR	ENSMUSG	766	-142.18
mmu-miR	ENSMUSG	765	-117.32
mmu-miR	Gm38850	765	-116.38
mmu-miR	ENSMUSG	764	-75.07
mmu-miR	ENSMUSG	764	-74.37
mmu-let-	ENSMUSG	763	-98.97
mmu-miR	ENSMUSG	763	-69.22
mmu-miR	Gm38850	761	-130.22
mmu-miR	Gm38850	761	-106.81
mmu-miR	ENSMUSG	761	-59.35
mmu-miR	ENSMUSG	760	-80.64
mmu-miR	ENSMUSG	760	-63.35
mmu-miR	Gm38850	759	-108.94
mmu-miR	ENSMUSG	759	-59.92

mmu-miR	ENSMUSG	758	-122.92
mmu-miR	ENSMUSG	758	-110.78
mmu-miR	ENSMUSG	757	-102.78
mmu-miR	ENSMUSG	757	-99.04
mmu-miR	Gm38850	757	-95.76
mmu-miR	Gm38850	756	-123.12
mmu-let-	ENSMUSG	756	-97.06
mmu-miR	ENSMUSG	755	-87.95
mmu-miR	ENSMUSG	755	-86.71
mmu-miR	Gm38850	754	-91.52
mmu-miR	ENSMUSG	753	-113.38
mmu-miR	ENSMUSG	753	-59.27
mmu-miR	ENSMUSG	752	-93.08
mmu-miR	ENSMUSG	751	-94.6
mmu-miR	Gm38850	750	-96.56
mmu-miR	ENSMUSG	750	-83.14
mmu-miR	ENSMUSG	750	-72.55
mmu-miR	Gm41235	749	-88.34
mmu-miR	Gm38850	749	-87.02
mmu-miR	ENSMUSG	747	-103.4
mmu-miR	ENSMUSG	747	-77.6
mmu-miR	ENSMUSG	746	-94.4
mmu-miR	ENSMUSG	745	-107.84
mmu-miR	Gm38850	745	-94.12
mmu-miR	Gm38850	745	-92.06
mmu-miR	ENSMUSG	745	-91.83
mmu-miR	ENSMUSG	745	-83.91
mmu-miR	ENSMUSG	744	-106.27
mmu-miR	Gm38850	744	-91.78
mmu-miR	Gm38850	743	-108.04
mmu-miR	Gm38850	743	-90.1
mmu-miR	Gm38850	743	-81.83
mmu-miR	ENSMUSG	742	-100.44
mmu-miR	Gm38850	742	-98.67
mmu-miR	ENSMUSG	742	-93.82
mmu-miR	ENSMUSG	742	-93.27
mmu-miR	Gm38850	741	-98.01
mmu-miR	ENSMUSG	741	-83.48
mmu-miR	ENSMUSG	741	-75.07
mmu-let-	ENSMUSG	740	-86.54
mmu-miR	ENSMUSG	740	-78.38
mmu-miR	ENSMUSG	740	-59.1
mmu-miR	ENSMUSG	740	-51.98
mmu-miR	Gm38850	739	-103.85
mmu-miR	ENSMUSG	739	-91.22
mmu-miR	ENSMUSG	739	-89.5
mmu-miR	ENSMUSG	739	-72.54
mmu-miR	ENSMUSG	739	-66.44
mmu-miR	ENSMUSG	738	-98.55
mmu-miR	ENSMUSG	738	-93.16
mmu-let-	ENSMUSG	738	-71.96
mmu-miR	ENSMUSG	737	-86.81
mmu-miR	ENSMUSG	737	-85.62
mmu-miR	ENSMUSG	735	-93.82
mmu-let-	ENSMUSG	735	-86.09
mmu-let-	ENSMUSG	735	-81.53
mmu-miR	ENSMUSG	735	-60.98
mmu-miR	Gm38850	733	-113.11

mmu-miR	ENSMUSG	733	-83.28
mmu-miR	Gm38850	732	-83.63
mmu-let-	ENSMUSG	732	-81.81
mmu-let-	ENSMUSG	732	-72.64
mmu-miR	ENSMUSG	732	-56.87
mmu-miR	ENSMUSG	731	-80.48
mmu-miR	ENSMUSG	731	-61.76
mmu-miR	ENSMUSG	730	-87.57
mmu-miR	ENSMUSG	730	-68.24
mmu-miR	ENSMUSG	729	-85.21
mmu-miR	ENSMUSG	728	-67.08
mmu-miR	ENSMUSG	728	-47.8
mmu-miR	ENSMUSG	726	-90.41
mmu-let-	ENSMUSG	724	-74.06
mmu-miR	ENSMUSG	724	-57.48
mmu-miR	ENSMUSG	723	-80.54
rco-miR15	ENSMUSG	722	-67.6
rco-miR15	ENSMUSG	722	-67.6
rco-miR15	ENSMUSG	722	-67.6
mmu-miR	ENSMUSG	722	-66.54
mmu-miR	ENSMUSG	722	-56.16
mmu-miR	Gm38850	718	-78.44
mmu-miR	ENSMUSG	717	-92.66
mmu-miR	ENSMUSG	715	-94.23
mmu-miR	ENSMUSG	713	-63.1
mmu-miR	ENSMUSG	712	-78.6
mmu-miR	ENSMUSG	708	-68.27
mmu-miR	ENSMUSG	707	-61.04
