# Supplementary material for: Comparative transcription profiling of mRNA and lncRNA in pulmonary arterial hypertension after C75 treatment
Source: BMC Pulm Med. 2023 Jan 31;23:46. doi: 10.1186/s12890-023-02334-6 (PMC9887911; doi:10.1186/s12890-023-02334-6)
Supplement: Supplementary file 4 — Additional file 4. Figure S1. Co-expression networks of lncRNAs and protein-coding genes. [file 12890_2023_2334_MOESM4_ESM.docx]

**
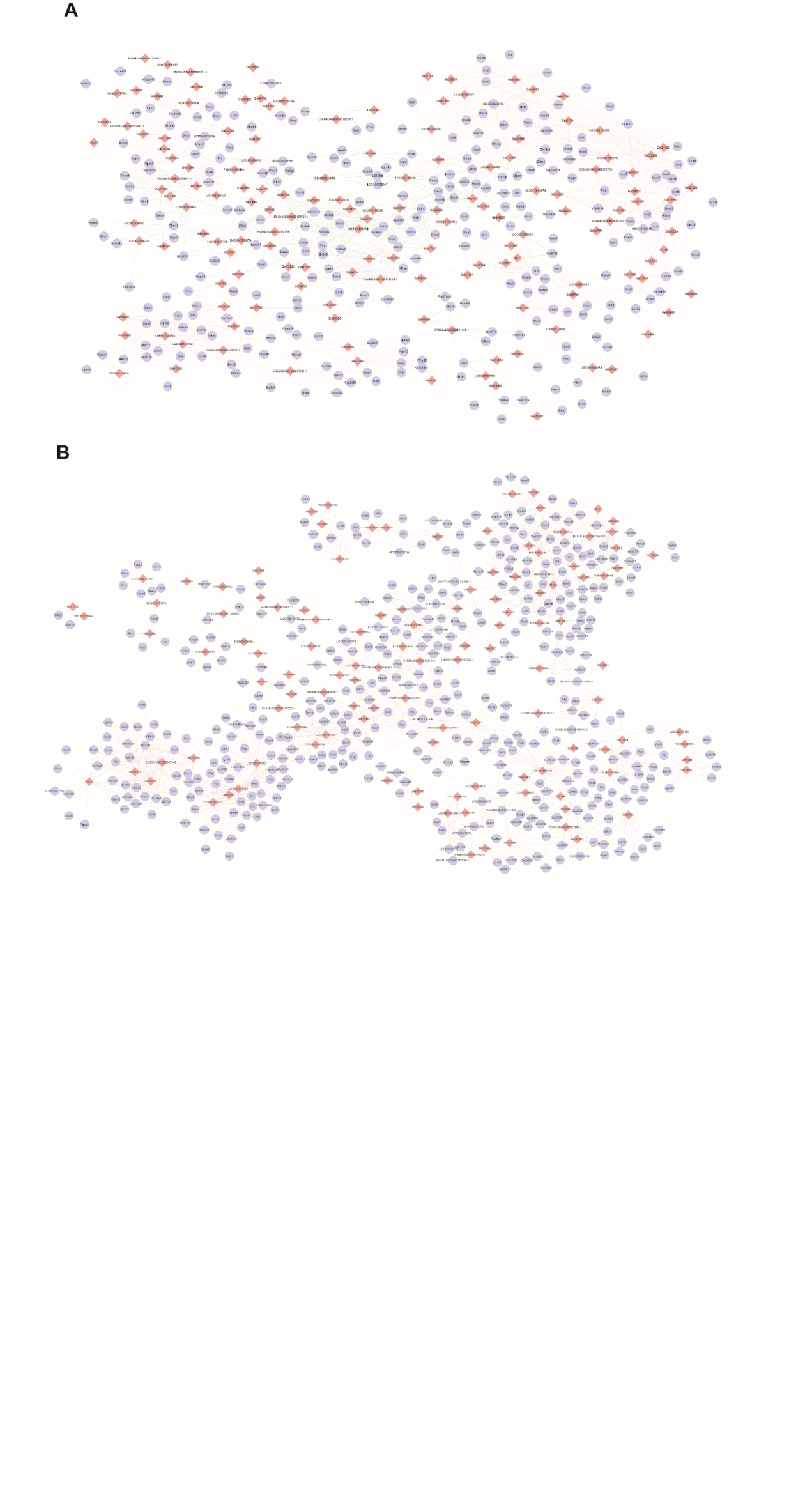
**

**Supplementary Figure 1**

**Supplementary Figure 1.** Co-expression networks of lncRNAs and protein-coding genes.

**Supplementary Table 1**. The sequences of the six lncRNAs (in the attachment of supporting information).

**Supplementary Table 2**. Lists of six lncRNAs and 1623 miRNAs (in the attachment of supporting information).

**Supplementary Table 3**. List of filtered 259 lncRNA-miRNA relationship pairs (in the attachment of supporting information).
